# Development of a Series of Tanshinone Derivatives Through Scaffold Hopping for Treating Non-Small-Cell Lung Cancer (NSCLC)

**DOI:** 10.3390/molecules31030446

**Published:** 2026-01-27

**Authors:** Lan-Xin Zhou, Zheng-Yu Shu, Heng Li, Hui Zhong, Dou-Nan Xu, Lei Tang, Chu-Jiao Hu, Cheng Luo, Huan Xiong

**Affiliations:** 1 State Key Laboratory of Discovery and Utilization of Functional Components in Traditional Chinese Medicine, Guizhou Provincial Key Laboratory of Innovation and Manufacturing for Pharmaceuticals, Guizhou Medical University, Guiyang 550004, China; zhoulanxin607@zidd.ac.cn (L.-X.Z.); 2024120030698@stu.gmc.edu.cn (Z.-Y.S.); tlei1974@163.com (L.T.); 2 Zhongshan Institute for Drug Discovery, Shanghai Institute of Materia Medica, Chinese Academy of Sciences, Zhongshan 528400, China; zhonghui1900@zidd.ac.cn (H.Z.); xudounan@zidd.ac.cn (D.-N.X.); 3 School of Chinese Materia Medica, Nanjing University of Chinese Medicine, Nanjing 210023, China; liheng2022@foxmail.com; 4 Guangzhou University of Chinese Medicine, Guangzhou 510006, China; 5 Key Laboratory for Cancer Prevention and Treatment of Guizhou Province, Guiyang 550004, China

**Keywords:** tanshinone derivatives, scaffold hopping, NSCLC, cytotoxic activity

## Abstract

Non-small-cell lung cancer (NSCLC) is one of the most prevalent cancer types and accounts for the majority of cancer-related deaths worldwide. Tanshinone and its derivatives exhibit diverse biological activities, and their prominent antitumor potential has been well documented. In this study, we rationally designed a series of tanshinone derivatives with a scaffold-hopping strategy. Thirty-five tanshinone derivatives were synthesized, and their cytotoxic activities against the NSCLC cell lines A549 and H838 were investigated. Concurrently, their safety profile was assessed in BEAS-2B cells. The results showed that compounds **S2-1**, **S2-4**, and **S2-8** exhibited superior inhibitory activity against A549 cells compared with the positive control, β-lapachone. Meanwhile, compounds **S2-1**, **S2-3**, **S2-4**, **S2-8**, **S2-13**, and **S2-14** exhibited similar or increased antiproliferation activity against H838 cells. Compounds **S2-4** (0.58 ± 0.07 μM) and **S2-8** (0.42 ± 0.04 μM) demonstrated the greatest potency towards H838 cells; compounds **S2-13** (1.28 ± 0.13 μM) and **S2-14** (1.80 ± 0.24 μM) exhibited potent and selective activity towards H838 cells. Molecular docking studies of S2-4/NLRP3 and S2-14/STAT3, combined with the structure–activity relationship (SAR) analysis, indicated that the benzofuran core containing an *ortho*-quinone, along with an amide linkage and a 1,2,3-triazole group introduced at the C-2 position of the furan ring, is an effective chemical scaffold for enhancing the anti-NSCLC activity of tanshinone derivatives.

## 1. Introduction

**Figure 1:**
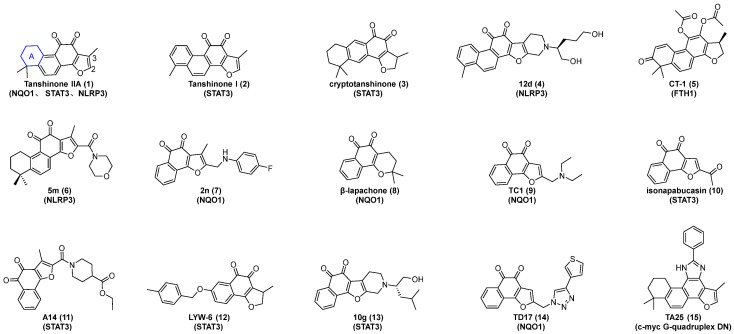
The structures of representative tanshinone derivatives and their corresponding targets.

Tanshinones, including **tanshinone IIA** (Figure 1, compound **1**), **tanshinone I** (Figure 1, compound **2**), and **cryptotanshinone** (Figure 1, compound **3**), are a class of bioactive constituents extensively employed in traditional Chinese medicine (TCM) for the treatment of diverse diseases [1,2]. Structurally, this class of compounds typically contains four rings consisting of naphthalene or tetrahydronaphthalene rings, an *ortho*- or *para*-quinone or lactone ring, and a furan or dihydrofuran ring (Figure 1) [3]. At present, accumulating evidence has demonstrated that natural and synthetic derivatives bearing the tanshinone scaffold exhibit diverse biological activities, with their validated molecular targets encompassing NAD(P)H: quinone oxidoreductase 1 (NQO1) [4,5], signal transducer and activator of transcription 3 (STAT3) [6,7], NOD-like receptor family pyrin domain-containing 3 (NLRP3) [1], and ferritin heavy chain 1 (FTH1) [8], among others (Figure 1). In 2023, Li et al. synthesized a new type of NLRP3 inhibitor via hybridizing the natural product **tanshinone I** with a piperidine scaffold. Compared with **tanshinone I**, these new derivatives showed significant improvements in potency, selectivity, and drug-likeness. Notably, compound **12d** (Figure 1, compound **4**) exerted a potent inhibitory effect on interleukin-1β (IL-1β) secretion, with a half-maximal inhibitory concentration (IC_50_) value of 0.065 μM, and it abrogated apoptosis-associated speck-like protein containing CARD (ASC) oligomerization by blocking NLRP3 inflammasome activation [1]. In 2025, Liu et al. developed **CT-1** (Figure 1, compound **5**), a novel derivative of **cryptotanshinone**, and further identified FTH1—a key protein in cellular iron metabolism—as its direct molecular target with a Kd value of 17.15 μM [8]. In the same year, Zhu et al. discovered compound **5m** (Figure 1, compound **6**), a potent and selective NLRP3 inflammasome inhibitor with a Kd value of 1.34 μM [9].

Recent studies have demonstrated that certain tanshinone analogs retain or even enhance antitumor activity upon the removal of ring A while retaining the furan ring [10,11,12]. In 2014, Bian et al. designed and synthesized compound **2n** (Figure 1, compound **7**) based on **tanshinone IIA** and **β-lapachone** (Figure 1, compound **8**). This compound exhibited a higher metabolic rate by NQO1 (1302 ± 180 µmol NADPH/min/µmol NQO1) compared to **β-lapachone** (1146 ± 23 µmol NADPH/min/µmol NQO1) [13]. Similarly, Li et al. synthesized twenty-seven *L*-shaped *ortho*-quinone analogs via the removal of ring A from **tanshinone IIA** and the C-3 position methyl group from the furan ring and the introduction of diverse side chains at the C-2 position. Approximately half of these compounds exhibited potent broad-spectrum cytotoxicity against human leukemia cells K562, prostate cancer cells PC3, and melanoma cells WM9. Especially, compound **TC1** (Figure 1, compound **9**, IC_50_ = 0.347 μM) exerted a high inhibitory effect on prostate cancer cells PC3 [12]. In 2018, Locken et al. synthesized **isonapabucasin** (Figure 1, compound **10**) and identified it as a STAT3 inhibitor with antiproliferative activity against cancer. This compound exhibited potent inhibitory effects against MDA-MB-231 and K562 cells, with IC_50_ values of 0.74 ± 0.01 μM and 1.1 ± 0.16 μM, respectively [6,14]. Subsequently, Chen et al. utilized **isonapabucasin** as the lead compound to design and synthesize compound **A14** (Figure 1, compound **11**), which showed potent inhibitory activity against HepG2 and K562 cells, with IC_50_ values of 0.88 μM and 2.16 μM, respectively [15,16]. **Cryptotanshinone**, a bioactive component isolated from Salvia miltiorrhiza Bunge, moderately inhibits STAT3 but exhibits limited selectivity [7]. In light of this, Wang et al. modified **cryptotanshinone** using a structure-based drug design strategy and synthesized isonapabucasin derivatives, such as **LYW-6** (Figure 1, compound **12**), which exhibited a high STAT3 binding affinity with a dissociation constant (Kd) value of 6.6 μM [7]. Through the scaffold-hopping design of naphthoquinone natural products (**napabucasin**, **isonapabucasin**, and **cryptotanshinone**) and a multicomponent synthetic approach, Li et al. discovered a novel naphthoquinone–furan–piperidine scaffold, which is analogous to tanshinone without ring A. Derivatives of this scaffold exhibit inhibitory effects on STAT3. Among them, compound **10g** (Figure 1, compound **13**) can inhibit the phosphorylation of STAT3 with a Kd value of 8.30 μM [17].

**Scheme 1:**
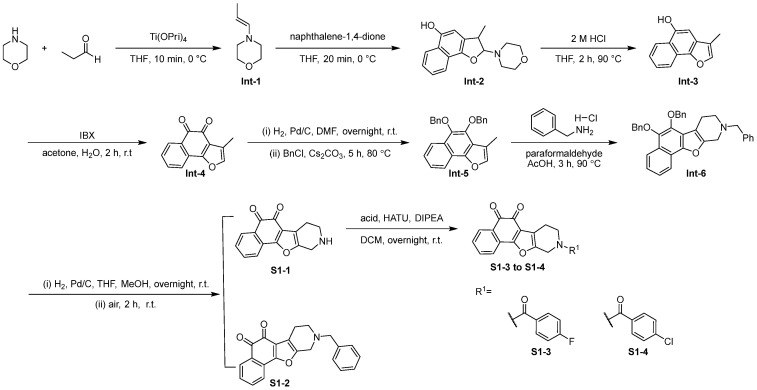
Synthesis of Series 1 compounds.

Furthermore, previous studies have found that the broad-spectrum anticancer activity of *L*-shaped *ortho*-quinone analogs, namely 3-methylnaphtho[1,2-*b*]furan-4,5-dione (Scheme 1, **Int-4**), can be enhanced by removing the methyl group at the C-3 position and introducing 1,2,3-triazole at the C-2 terminal of the furan ring [18]. In 2021, Yu et al. designed, synthesized, and evaluated the proliferation inhibitory activity of a novel *L*-shaped *ortho*-quinone analog **TD17** (Figure 1, compound **14**). The IC_50_ values of **TD17** against PC3, Hela, A549, MDA-231, and K562 cells were 1.190 ± 0.180, 0.862 ± 0.047, 2.430 ± 1.257, 0.991 ± 0.091, and 1.216 ± 0.366 μM, respectively. Moreover, it was found to specifically bind to the NQO1 protein in prostate cancer cells PC3 and leukemia cells K562 [18].

Additionally, certain studies have demonstrated that compounds formed by combining the *ortho*-quinone part of the tanshinone scaffold with aldehydes to construct an imidazole ring still exhibit anticancer activity [19]. In 2021, Wang et al. designed and synthesized a tanshinone IIA imidazole derivative **TA25** (Figure 1, compound **15**). They further verified that **TA25** could act as a potential inhibitor against the proliferation, migration, and invasion of lung cancer A549 cells, with an IC_50_ value of about 17.9 μM [19].

**Figure 2:**
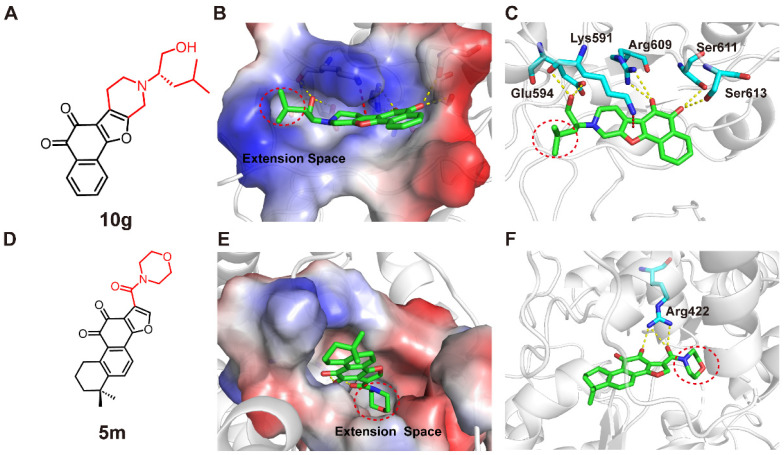
Reported docking modes of tanshinone derivatives with corresponding targeting proteins. (**A**) Structure of compound **10g**; (**B**) electrostatic potential surface of the predicted binding pose of compound **10g** in the STAT3 pocket. (PDB ID: 1BG1); and (**C**) the predicted binding poses between compound **10g** and STAT3 residues. Red dashed lines indicate π–cation interactions, and yellow dashed lines represent hydrogen bonds; (**D**) structure of compound **5m**; (**E**) electrostatic potential surface of the predicted binding pose of compound **5m** in the NLRP3 pocket (PDB ID: 7ALV); and (**F)** the predicted binding pose between compound **5m** and NLRP3 residues. Yellow dashed lines represent hydrogen bonds.

**Figure 3:**
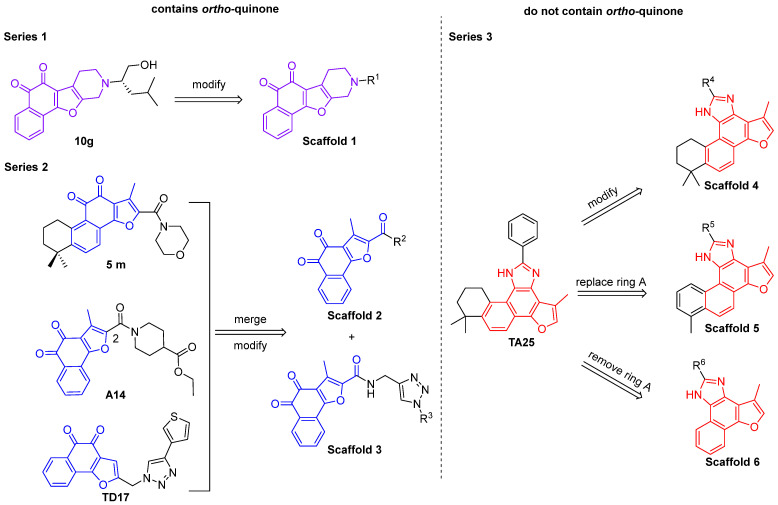
Design rationale for the compounds in this study.

Our analysis of the reported binding modes of tanshinone derivatives and their corresponding targeting proteins revealed that the *ortho*-quinone group on the tanshinone scaffold commonly participates in hydrogen bonding with residues in the protein binding pocket. Moreover, in the docking structure of compound **10g** (Figure 1, compound **13**) and STAT3 [17], it forms a stabilizing π–cation interaction between the furan ring of compound **10g** and Lys591 of STAT3 (Figure 2C). Notably, there is accessible extension space around the alkyl group (Figure 2B). In the docking structure of compound **5m** (Figure 1, compound **6**) and NLRP3 [9], the carbonyl side chain of compound **5m** formed a hydrogen bond with the Arg422 of NLRP3, while the morpholine ring of compound **5m** was fitted into a hydrophobic pocket defined by Ile74, Ile255, Met505, and Tyr476 (Figure 2F). This pocket provides ample extension space for structural modification (Figure 2E). These interactions account for the high binding affinity of **5m** toward NLRP3. Based on these observations, we retained the tanshinone scaffold and designed three series of compounds by introducing different side chains or fused rings, with the aim of obtaining compounds exhibiting enhanced biological activity (Figure 3).

## 2. Discussion and Results

### 2.1. Design of the Tanshinone Derivatives

From a structure-based design perspective, the selection of R substituents in the compound series was guided by the binding pocket characteristics of the target proteins, as suggested by molecular docking analysis. For STAT3, the binding site contains positively charged residues such as Lys and Arg, which favor ligands capable of forming hydrogen-bonding and π-related interactions. Accordingly, aromatic/heteroaromatic substituents and amide bonds were introduced to provide π systems and hydrogen-bonding moieties, thereby enhancing binding stability. In this case, we designed Series 1 (Figure 3) compounds based on the binding mode of compound **10g**. While retaining the naphthoquinone–furo–piperidine scaffold of compound **10g**, these compounds introduced additional modifications to the furan ring to promote more interactions with surrounding amino acid residues.

In contrast, the NLRP3 binding pocket contains a well-defined hydrophobic region formed by residues such as Ile74, Ile255, Met505, and Tyr476. Accordingly, aromatic and heteroaromatic substituents were selected as R substituents to fit this hydrophobic pocket and enhance van der Waals interactions. Moreover, pocket geometry allows for further structural extension at the modification site, supporting the introduction of substituents with suitable size and polarity. Therefore, in Series 2 of the compound design (Figure 3), we analyzed the binding mode of compound **5m** with the NLRP3 protein and found that introducing an amide bond and cyclic structures on the furan ring side chain could enhance the binding stability. Combining these findings with the scaffold of compound **A14**, we attached an amide group to the C-2 position of the furan ring of compound **A14** to obtain Scaffold 2. Furthermore, by analyzing the structure of compound **TD17**, we introduced a 1,2,3-triazole group based on Scaffold 2, thereby yielding Scaffold 3 (Figure 3).

As reported in the literature, tanshinone scaffolds retain anticancer activity even in the absence of the *ortho*-quinone group [19]. Therefore, unlike the chemical scaffolds of Series 1 and Series 2, we also attempted to synthesize a subset of compounds devoid of the *ortho*-quinone. In Series 3 of the compound design (Figure 3), we took the scaffold of **TA25** as the lead structure and performed structural modifications following three strategies. Firstly, we structurally modified the benzene ring of **TA25** to obtain Scaffold 4. Secondly, we replaced the tanshinone IIA scaffold of **TA25** with **tanshinone I**, that is, we replaced ring A, while simultaneously modifying the benzene ring to obtain Scaffold 5. Finally, we removed ring A from the tanshinone IIA scaffold of **TA25** and further modified the benzene ring to obtain Scaffold 6.

Based on these design considerations, through structural modifications of the above-mentioned six scaffolds (Figure 3), we designed and synthesized a series of tanshinone derivatives and evaluated their corresponding in vitro inhibitory activities via cellular assays, with the aim of applying these derivatives to the treatment of non-small-cell lung cancer.

### 2.2. Chemistry

**Scheme 2:**
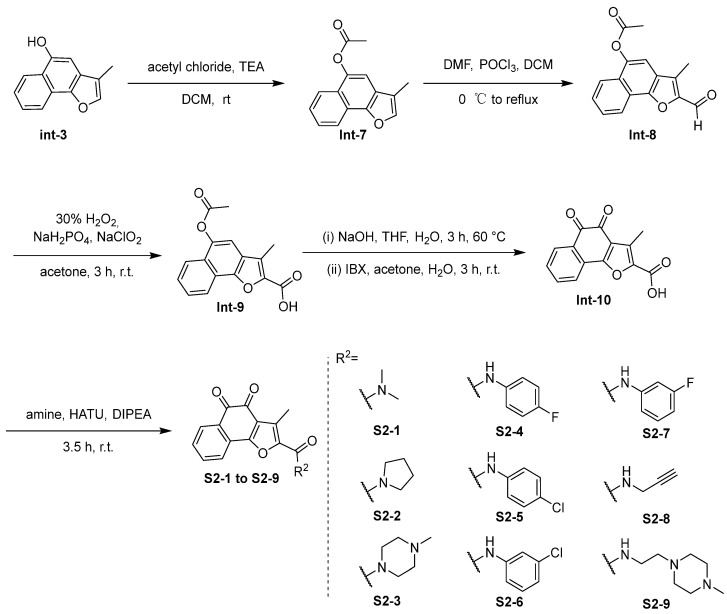
Synthesis route 1 of Series 2 compounds.

**Scheme 3:**
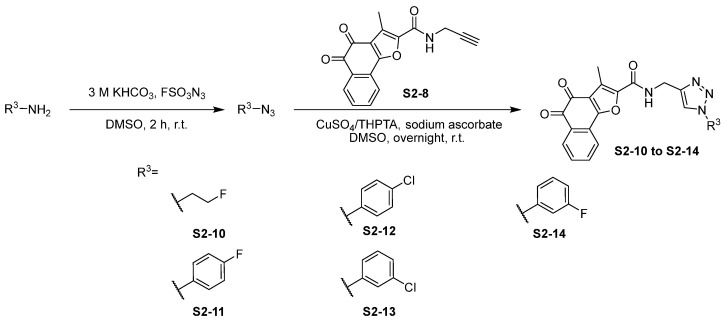
Synthesis route 2 of Series 2 compounds.

**Scheme 4:**
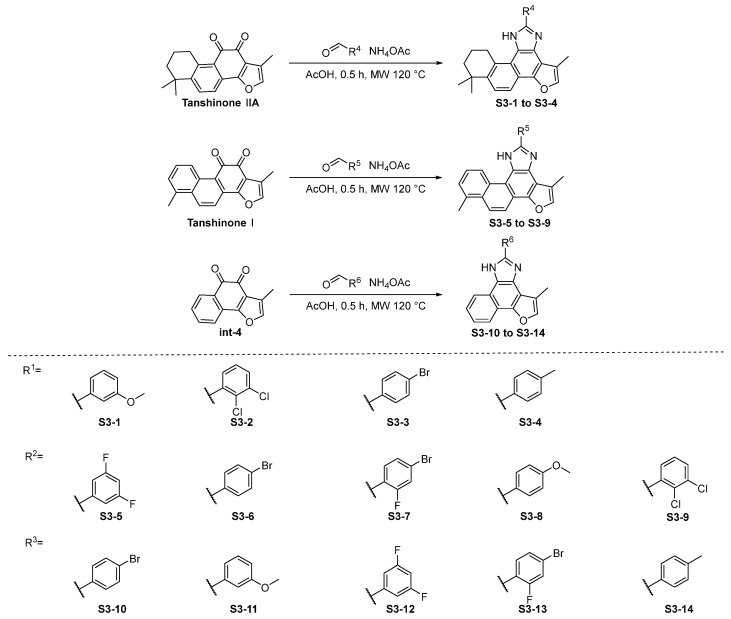
Synthesis of Series 3 compounds.

The synthesis of three series tanshinone derivatives is outlined in Scheme 1, Scheme 2, Scheme 3 and Scheme 4. In brief, we employed four synthetic routes to synthesize 35 compounds. The synthesis of compounds in Series 1 is shown in Scheme 1. The commercially available reagents morpholine and propionaldehyde were reacted via a reductive amination reaction to produce intermediate **Int-1**. **Int-2** was obtained by condensation reaction, the reaction mixture was filtered through Celite, and the filtrate was subjected to extraction, vacuum concentration, and other conventional workup procedures. The obtained residue was then treated with 2 M hydrochloric acid and heated to render morpholine release and afford intermediate **Int-3**. This process is a one-pot, three-step sequence. The yield could be improved by moderately extending the heating time. Subsequent oxidation reaction with **Int-3** in the presence of 2-iodoxybenzoic acid (IBX) gave intermediate **Int-4**. This then underwent hydrogenation in the presence of Pd/C and a sequential benzylation to obtain intermediate **Int-6**. Intermediates **Int-5** and **Int-6** exhibited extremely low polarity, necessitating purification using pure petroleum ether as the mobile phase, which rendered the isolation process rather challenging. **S1-1** and **S1-2** were generated through a debenzylation reaction involving intermediate **Int-6**. Amide coupling of **S1-1** with carboxylic acid linkers provides the desired compounds **S1-3** and **S1-4**.

The synthesis of compounds in Series 2 is shown in Scheme 2 and Scheme 3. In Scheme 2, the intermediate **Int-3** was reacted via an acylation reaction to produce intermediate **Int-7**. **Int-8** was obtained by the Vilsmeier–Haack reaction with POCl_3_, followed by an oxidation reaction to afford intermediate **Int-9**. Subsequent basic ester hydrolysis reaction with NaOH and an oxidation reaction with IBX gave intermediate **Int-10**. Amide coupling of **S1-1** with amine linkers provides the desired compounds **S2-1** to **S2-9**. The use of highly pure intermediate **Int-10** in the amide condensation step helped to improve the yields of the target compounds **S2-1** to **S2-9**. When introducing substituents to the carboxylic acid group of **Int-10**, the incorporation of hydrophobic groups, especially aromatic rings, such as **S2-4** to **S2-7**, significantly reduced the solubility of the resulting compounds, thereby increasing the difficulty of reaction and purification.

In Scheme 3, the commercially available amino reagents were reacted via an azidation reaction with FSO_3_N_3_ to produce azides, followed by click reactions to provide the desired compounds **S2-10** to **S2-14**. In the synthesis of compounds in Scheme 3, the poor solubility of the target compound resulted in a relatively low yield and made purification more challenging, particularly for compound **S2-10**.

The synthesis of compounds in Series 3 is shown in Scheme 4. The *ortho*-quinone group of **tanshinone IIA**, **tanshinone I**, and **int-4**, with different commercially available aldehydes, provide the desired compounds **S3-1** to **S3-14**.

### 2.3. In Vitro Cytotoxicity Results and Structure–Activity Trends Analysis

The cytotoxic activities of the synthesized tanshinone derivatives at concentrations of 5 μmol/L and 1 μmol/L were evaluated against two cancer cell lines, namely, A549 and H838. Concurrently, their safety profile was assessed in human bronchial epithelial (BEAS-2B) cells, which are commonly used in studies related to NSCLC (Table 1, Table 2 and Table 3).

The results of the compounds in **Series 1** are summarized in Table 1. The data demonstrated that the compounds in Series 1 and their intermediates generally displayed higher inhibitory activity against H838 cells than against A549 cells. Meanwhile, these compounds exhibited markedly attenuated inhibitory activities against the two aforementioned cell lines, and their safety profiles were comparable to those of the positive control drug **β-lapachone**. Analysis of the SAR of the compounds in Series 1 revealed that the inhibitory rates of both classes of compounds against A549 cell lines at 5 μmol/L were lower than those at 1 μmol/L. In addition, compared with the *ortho*-quinone-containing compounds (**Int-4**, **S1-1**, **S1-2**, **S1-3**, and **S1-4**), the *ortho*-quinone-free compounds **(Int-3** and **Int-5**) exhibited superior inhibitory rates against H838 cell lines at 1 μmol/L. In contrast, a converse tendency was manifested at 5 μmol/L. Additionally, experimental data from BEAS-2B cell lines indicated that the *ortho*-quinone-free compounds (**Int-3** and **Int-5**) showed significantly better safety profiles than the *ortho*-quinone-containing compounds (**Int-4**, **S1-1**, **S1-2**, **S1-3**, **S1-4**, and **β-lapachone**) at 5 μmol/L.

The results of compounds in **Series 2** are summarized in Table 2. The data demonstrated that compounds of **Series 2** exerted an overall substantially superior inhibitory activity relative to those of Series 1 against the two NSCLC cell lines, with distinct structure-dependent differences observed. At a concentration of 5 μM, most compounds exhibited an extremely potent inhibitory efficacy against H838 cells; specifically, **S2-1**, **S2-3**, **S2-4**, **S2-8**, **S2-13**, and **S2-14** all achieved inhibition rates approaching or exceeding 95%, which were comparable to or even superior to that of the positive control β-lapachone.

Notably, several of these compounds also displayed strong activity against A549 cells. In particular, **S2-1**, **S2-3**, **S2-4**, and **S2-8** showed inhibition rates greater than 80% against A549 cells at 5 μM, indicative of broad-spectrum antitumor activity. However, at a lower concentration of 1 μM, the inhibitory effects of most compounds diminished markedly in A549 cells, whereas moderate to strong inhibitory activity was still retained in H838 cells. This observation suggests that H838 cells possess a higher sensitivity to **Series 2** compounds.

In the normal lung epithelial cell line BEAS-2B, most **Series 2** compounds still maintained high inhibition rates at 5 μM. These results imply that the **Series 2** compounds, as a whole, exhibit considerable cytotoxicity, highlighting the necessity for further optimization to enhance their tumor/normal cell selectivity. Analysis of the data in Table 2 revealed that rational modification of the furan ring side chain of the benzofuran-structured compound (**Int-4**) significantly enhanced the inhibitory efficacy of the resulting derivatives (**S2-1**, **S2-3**, **S2-4**, and **S2-8**) against the two cell lines. For Scaffold 3, the side chain introduction of meta-halogen substituents yielded compounds (**S2-13**, **S2-14**) with superior inhibitory activity against the two aforementioned cell lines compared to their *para*-substituted (**S2-11**, **S2-12**) and the alkane-substituted compound (**S2-10**).

The results of the compounds in **Series 3** are summarized in Table 3. The data demonstrated that comparative analysis of tanshinone II A versus **S3-1** to **S3-4**, tanshinone I versus **S3-5** to **S3-9**, and **Int-4** versus **S3-10** to **S3-14** against A549 and H838 cell lines, respectively, revealed that the tanshinone scaffold still exerted a certain degree of inhibitory effect on both cell lines even in the absence of *ortho*-quinone groups, and the inhibitory effect was maintained to a certain extent or slightly enhanced. Although the compounds in **Series 3** showed relatively weak inhibitory activity against the tested A549 and H838 cell lines compared to the positive compound β-lapachone, their cytotoxicity towards the normal cell line BEAS-2B was significantly reduced.

By performing structural modifications on six chemical scaffolds, we successfully established a highly efficient synthetic strategy and prepared three series of tanshinone derivatives. Based on the results of cytotoxicity assays (Table 1, Table 2 and Table 3), a preliminary SAR of these compounds was constructed. Among the six scaffolds, those bearing the benzofuran moiety, namely, Scaffold 2 and Scaffold 3, exhibited superior inhibitory activity against both A549 and H838 cell lines. The *ortho*-quinone-containing compounds in **Series 2** (e.g., **S1-1**, **S2-3**, **S2-4**, **S2-8**, **S2-13,** and **S2-14**) exhibited superior inhibitory effects on the two cell lines than these *ortho*-quinone-free compounds in Series 3. Furthermore, the introduction of the amido bond and 1,2,3-triazole at the C-2 position of the furan ring in **Series 2** compounds, as exemplified by **S2-13** and **S2-14** of Scaffold 5, was found to enhance the cell line selectivity to a certain extent.

**Figure 4:**
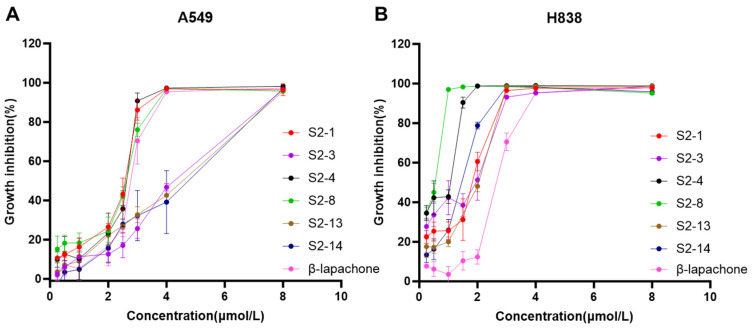
Growth inhibition induced by the tanshinone derivatives on (**A**) A549 cell line; and (**B**) H838 cell line by CCK-8 assay. The IC_50_ values (μM) of the compounds were determined according to these cures at different incubation times. The 100 μL tested compounds were added to 96-well microculture plates, and 100 μL cells (a final concentration of 6 × 10^3^/well) were incubated for 48 h at 37 °C. Cell survival was evaluated by CCK-8 assay. The inhibition ratio (%) was calculated as described in the Methods section. Data was presented as mean ± SD of three independent experiments.

To ensure the efficient use of resources and focus on the most promising candidates, IC_50_ values were determined only for relatively strong active compounds identified in the initial screening. The concentration inhibition curves (Figure 4) were analyzed to calculate the IC_50_ values of the selected active compounds. The results indicated that there was a dose-dependent trend of the inhibitory response of all active compounds on two cancer cells after 8 h of treatment. The IC_50_ values were summarized in Table 4, which shows that these Series 2 compounds exhibit μM-level inhibitory activity against both NSCLC cell lines and show an overall higher sensitivity toward H838 cells. In particular, the IC_50_ values of **S2-4** and **S2-8** against H838 cells were 0.58 ± 0.07 μM and 0.42 ± 0.04 μM, respectively, which were significantly superior to that of the positive control **β-lapachone**, demonstrating potent cytotoxic inhibition potential. In contrast, the activity differences among the tested compounds were relatively small in the A549 cell line. Nevertheless, **S2-1**, **S2-4**, and **S2-8** still maintained relatively low IC_50_ values (approximately 2.4–2.5 μM), suggesting that these compounds may possess specific cell line selectivity, with a particular preference for exhibiting H838 cells. This result provided a preliminary biological activity basis for investigating anticancer candidate agents for NSCLC.

### 2.4. Effects of Bioactive Compounds on Cell Proliferation and Migration

**Figure 5:**
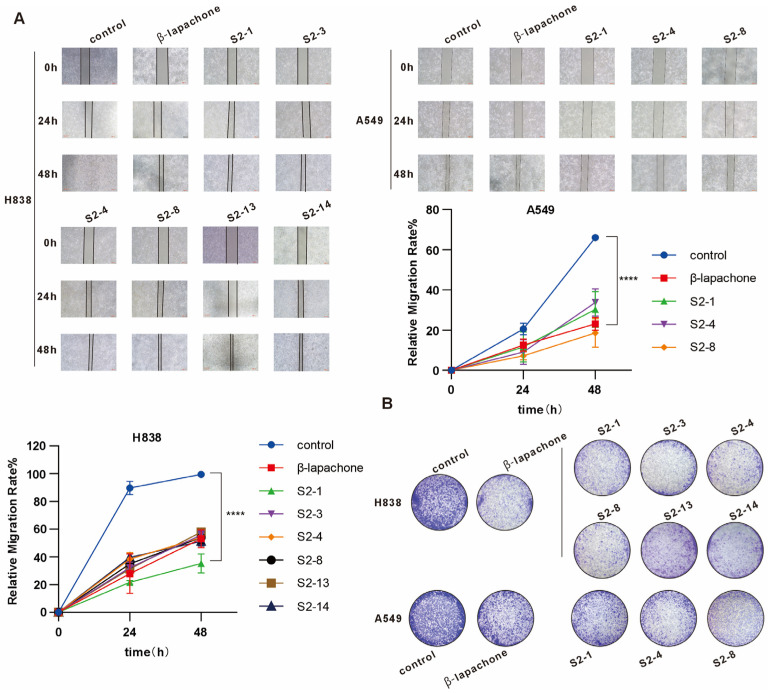
Effects of active compounds on cell proliferation and migration. (**A**) Shows the wound healing of H838 and A549 cells at different time points (24 h and 48 h) in the control group, β-lapachone treatment group, and multiple active compound treatment groups, as well as the changes in the relative migration rate of each group over time. (**B**) Effect of active compounds on plate colony formation of A549 and H838 lung cancer cells. Note: **** represents *p* < 0.01.

Based on the half-maximal inhibitory concentration (IC_50_) values of all the aforementioned bioactive compounds, six compounds (**S2-1**, **S2-3**, **S2-4**, **S2-8**, **S2-13**, and **S2-14**) were selected for H838 cells, whereas three compounds (**S2-1**, **S2-4**, and **S2-8**) were chosen for A549 cells. All these compounds exhibited higher bioactivity than the positive control group. To further investigate the impacts of these bioactive compounds on cell proliferation and migration, a wound healing assay (Figure 5A) and colony formation assay (Figure 5B) were performed in this study. Results of the colony formation assay (Figure 5B) demonstrated that treatment with the aforementioned compounds at their respective IC_50_ concentrations significantly suppressed the colony-forming capacity of the cells. Compared with the control group, the number of cell colonies in each compound treatment group decreased markedly, with statistically significant differences (*p* < 0.05). These findings suggested that these bioactive compounds could effectively inhibit cell proliferative capacity and reduce colony formation. Results of the wound healing assay indicated that the migratory capacity of both H838 and A549 cells was significantly inhibited after treatment with the aforementioned bioactive compounds. During the wound healing process, compared with the control group, the cell migration rate in each compound treatment group slowed down distinctly, and the closure degree of the scratched area was significantly reduced at the same time point. Specifically, for H838 cells, the relative area of the scratched region in the **S2-1**, **S2-3**, **S2-4**, **S2-8**, **S2-13**, and **S2-14** treatment groups was significantly larger than that in the control group at 24 h and 48 h post-scratching. Moreover, this difference gradually increased over time, suggesting that these compounds could exert a sustained inhibitory effect on cell migration. Similarly, for A549 cells, the **S2-1**, **S2-4**, and **S2-8** treatment groups also showed analogous migration inhibition at different time points after the wound healing assay, with the relative area of the scratched region significantly larger than that in the control group. Additionally, certain differences were observed among the treatment groups of different compounds, which might be associated with their chemical structures and mechanisms of action. Furthermore, the extent of the inhibitory effect of each compound on cell migration was further quantified by calculating the cell migration rate. The results indicated that the inhibitory effects of these bioactive compounds on the migration of H838 and A549 cells were dose-dependent and time-dependent, and the inhibitory efficacy was negatively correlated with their IC_50_ values. In other words, compounds with lower IC_50_ values exerted stronger inhibitory effects on cell migration.

### 2.5. Molecular Docking Analysis of the Designed Compounds with Corresponding Target Proteins

**Figure 6:**
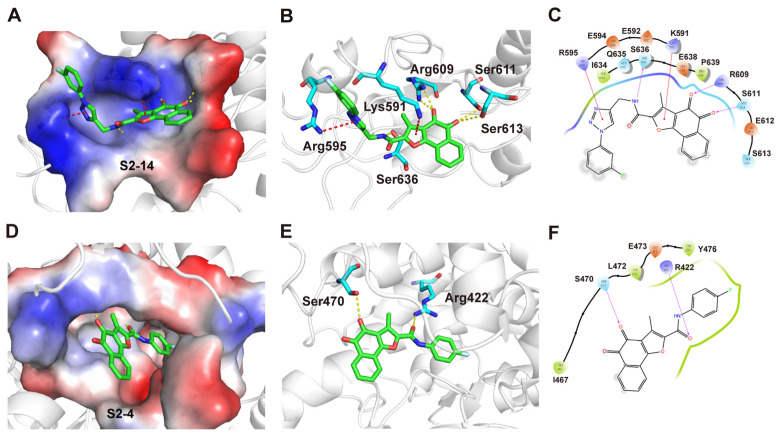
Molecular docking analysis of compound **S2-14** with STAT3 (PDB ID: 1BG1) and compound **S2-4** with NLRP3 (PDB ID: 7ALV). (**A**) Electrostatic potential surface for the predicted binding pose of compound **S2-14** in the STAT3 pocket; (**B**) the predicted binding pose between compound **S2-14** and STAT3 residues. Red dashed lines indicate π–cation interactions, and yellow dashed lines represent hydrogen bonds; (**C**) 2D interaction diagram of compound **S2-14** with STAT3. The red lines indicate π–cation interactions, and the purple lines represent hydrogen-bond interactions; (**D**) electrostatic potential surface of the predicted binding pose of compound **S2-4** in the NLRP3 pocket; (**E**) the predicted binding pose between compound **S2-4** and NLRP3 residues. Yellow dashed lines represent hydrogen bonds; and (**F**) 2D interaction diagram of compound **S2-4** with NLRP3. The purple lines represent hydrogen-bond interactions.

Bioactivity assays showed that **S2-4** and **S2-14** exhibited stronger inhibitory effects (Table 4). To further investigate the binding modes of the designed compounds, molecular docking was performed with STAT3 (PDB ID: 1BG1) and NLRP3 (PDB ID: 7ALV). The docking results indicated that both compounds adopted reasonable binding orientations within the corresponding protein targets. In the designed compounds, the *ortho*-quinone moiety of the tanshinone scaffold was retained, allowing for stable hydrogen bonding with nearby amino acid residues. In the docking structure of compound **S2-14** and STAT3, the amide bond introduced in **S2-14** enabled the formation of an additional hydrogen bond with the Ser636 of STAT3. The furan ring on the compound scaffold forms a π–cation interaction with the Lys591 of STAT3. Meanwhile, the triazole group of **S2-14** established a π–cation interaction with Arg595 of STAT3, thereby enhancing the stability of the docking mode (Figure 6A–C). These cooperative interactions helped anchor compound **S2-14** within the STAT3 binding pocket and contributed to its favorable binding orientation observed in the docking results. In the docking structure of compound **S2-4** and NLRP3, the amide bond of **S2-4** formed a hydrogen bond with the Arg422 of NLRP3. Meanwhile, its left-hand phenyl ring fitted into a hydrophobic pocket constituted by Ile74, Ile255, Met505, and Tyr476, which contributed to the stabilization of the binding mode and the high binding affinity of **S2-4** toward NLRP3 (Figure 6D–F). Therefore, the amide bond and the triazole group were essential for the designed small molecules to bind to STAT3 and NLRP3, indicating the success of this structural optimization strategy.

**Scheme 5:**
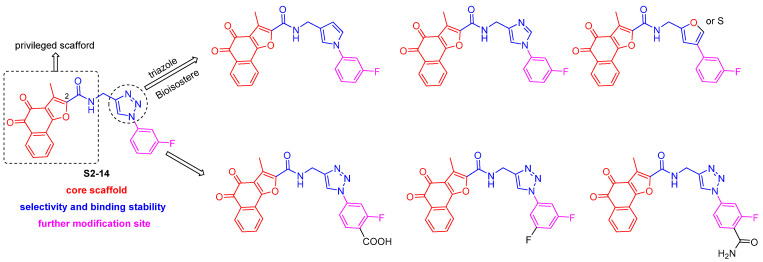
Further modifications of the core scaffold suggested by the present study.

Based on the above research findings, we make the following further recommendations regarding compound modification (take compound **S2-14** as an example). The benzofuran core scaffold bearing an *ortho*-quinone group should be retained as the core scaffold. Moving forward, an amide bond or 1,2,3-triazole groups should be introduced at the 2-position of the furan ring. Appropriate hydrophobic groups, such as alkyl or aryl rings, should then be introduced onto the triazole moiety to enhance the ligand’s binding affinity to the hydrophobic pocket of the protein through the hydrophobic effect. Alternatively, the 1,2,3-triazole group could be replaced with its bioisosteres, such as pyrrole, imidazole, and furan. On the other hand, to preserve the core structure of **S2-14**, new ways in which it interacts with proteins can be investigated, and negatively charged functional groups, such as carboxyl or amide groups, can be introduced at the ortho position of the fluorinated benzene ring (Scheme 5).

## 3. Materials and Methods

### 3.1. Instruments and Materials

High-resolution mass spectra (HRMS) were obtained on an electrospray ionization (ESI) mode on a Thermo Scientific ESI-QTOF mass spectrometer(Waltham, MA, USA). Nuclear magnetic resonance (NMR) spectra were recorded on a Bruker Avance NEO (^1^H NMR, 500 MHz, and 600 MHz; ^13^C NMR, 126 MHz, 151 MHz, and 201 MHz; and ^19^F 471 MHz, Bruker, Switzerland) with TMS as an internal standard. The purity of all tested compounds was confirmed by HPLC with an Agilent 1260 spectrometer (Waldbronn, Germany). HPLC analysis conditions: EC-C18, 2.7 μm, and 4.6 mm × 100 mm. Method: flow rate, 0.5 or 0.2 mL/min; eluent C, 0.1% formic acid in water; eluent D, acetonitrile; and gradient conditions were initially 5% B, 10% B, 20% B, 30% B, 50% B, 80% B, or 95% B, increasing linearly to 95% B over 10 min, decreasing linearly to initial eluent ratio over 1 min, and remaining at initial eluent ratio for 4 min. All compounds are >95% pure by HPLC analysis. The column chromatography was performed on silica gel (Shanghai, China, 300–400 mesh), and the thin-layer (0.25 mm) chromatography (TLC) analysis was carried out on silica gel plates (Huanghai, Yantai, China). The volumes of liquid reagents were measured using micropipettes (DLAB Scientific, Beijing, China). Other reagents were analytical grade or guaranteed reagent commercial products and used without further purification, unless otherwise noted.

### 3.2. Methods of Synthesis

#### 3.2.1. Synthesis of **S1-1** to **S1-4** [1,17,20,21,22]

##### Synthesis of 3-Methylnaphtho[1,2-b]furan-5-ol (**Int-3**)

A solution of propionaldehyde (7.205 mL, 100 mmol, 1.0 eq) and Ti(OPr^i^)_4_ (29.606 mL, 100 mmol, 1.0 eq) in THF (50 mL) was added dropwise to the solution of morpholine (8.757 mL, 100 mmol, 1.0 eq) in THF (50 mL) at 0 °C and stirred for 0.5 h. A solution of naphthalene-1,4-dione (15.815 g, 100 mmol, 1.0 eq) in THF (130 mL) was dropped into the reaction mixture with stirring for 1 h at 0 °C. Then, 2 M HCl aqueous (100 mL) was added to the reaction mixture, and the reaction mixture was heated to 90 °C and stirred for 6 h. After the reaction was complete, the resulting reaction mixture was filtered through Celite, and the filtrate was concentrated under reduced pressure to remove THF, extracted with water and EtOAc (×3), and the combined organic layers were dried over anhydrous Na_2_SO_4_, filtered, and removed in vacuo to afford the residue, which was pre-purified by flash column chromatography followed by prep and recrystallization from dichloromethane to afford **Int-3** (5.846 g, yield 29.5%) as a brown solid. ^1^H NMR (500 MHz, DMSO-*d*_6_) *δ* 9.96 (s, 1 H), 8.21 (d, *J* = 8.4 Hz, 1 H), 8.11 (d, *J* = 8.2 Hz, 1 H), 7.79 (s, 1 H), 7.60 (t, *J* = 7.5 Hz, 1 H), 7.47 (t, *J* = 7.6 Hz, 1 H), 6.95 (s, 1 H), and 2.22 (s, 3 H). ^13^C NMR (126 MHz, DMSO-*d*_6_) *δ* 149.2, 143.9, 141.4, 126.8, 124.0, 124.0, 123.3, 123.0, 121.2, 119.2, 116.1, 98.6, and 7.8. Purity: 98.9%. HRMS (ESI): calcd for C_13_H_10_O_2_ [M + H]^+^: 199.0754, found: 199.0757.

##### Synthesis of 3-Methylnaphtho[1,2-b]furan-4,5-dione (**Int-4**)

To a solution of **Int-3** (5.846 g, 29.49 mmol, 1.0 eq) in acetone (30 mL) and H_2_O (30 mL) was added slowly IBX (12.388 g, 44.24 mmol, 1.5 eq). The mixture was stirred at room temperature for 2 h. Then the resulting reaction mixture was concentrated under reduced pressure to remove THF, extracted with water and EtOAc (×3), and the combined organic layers were dried over anhydrous Na_2_SO_4_, filtered, and removed in vacuo to afford the residue. The residue was dissolved in dichloromethane, filtered, and the filtrate was collected to afford the relatively pure titled compounds, which was purified by flash column chromatography using a mixture of petroleum ether and ethyl acetate to afford **Int-4** (3.607 g, yield 57.6%) as a red solid. ^1^H NMR (500 MHz, DMSO-*d*_6_) *δ* 7.91 (d, *J* = 7.7 Hz, 1 H), 7.76–7.68 (m, 3 H), 7.55–7.50 (m, 1 H), and 2.18 (s, 3 H). ^13^C NMR (126 MHz, DMSO-*d*_6_) *δ* 179.7, 175.2, 159.3, 142.4, 135.1, 129.9, 129.3, 129.1, 127.9, 121.7, 120.8, 120.5, and 8.6. Purity: 95.8%. HRMS (ESI): calcd for C_13_H_8_O_3_ [M + H]^+^: 213.0546, found: 213.0549.

##### Synthesis of 4,5-Bis(benzyloxy)-3-methylnaphtho[1,2-b]furan (**Int-5**)

To a solution of **Int-4** (300 mg, 1.41 mmol, 1.0 eq) in DMF (5 mL), 10% Pd/C (38 mg) was added. The mixture was purged with hydrogen three times and stirred at room temperature for 9 h. Afterward, the reaction solution was transferred with a syringe to another two-neck flask, which had been previously charged with Cs_2_CO_3_ (31.382 g, 4.24 mmol, 3.0 eq) under an argon atmosphere. Benzyl chloride (488 μL, 4.24 mmol, 3.0 eq) was then added, and the mixture was stirred at 75 °C for 5 h. Upon reaction completion, the reaction solution was filtered through Celite, the filtrate was extracted with water and EtOAc (×3), and the combined organic layers were washed with water and saturated brine, dried over anhydrous Na_2_SO_4_, filtered, and removed in vacuo to afford the residue, which was purified by flash column chromatography using petroleum ether to afford **Int-5** (54 mg, yield 9.7%) as a colorless oil. ^1^H NMR (500 MHz, Chloroform-*d*) *δ* 8.27–8.20 (m, 2 H), 7.59–7.46 (m, 7 H), 7.46–7.34 (m, 6 H), 5.34 (s, 2 H), 5.20 (s, 2 H), and 2.36 (s, 3 H). Purity: 97.0%. HRMS (ESI): calcd for C_27_H_22_O_3_ [M + H]^+^: 395.1642, found: 395.1650. Other data were found in reference [21].

##### Synthesis of 9-Benzyl-5,6-bis(benzyloxy)-7,8,9,10-tetrahydronaptho[2′,1′:4,5]fur-o[2,3-c]pyridine (**Int-6**)

To a solution of **Int-5** (100 mg, 0.25 mmol, 1.0 eq) in AcOH (3 mL), phenylmethanamine hydrochloride (73 mg, 0.51 mmol, 2.0 eq) and paraformaldehyde (76 mg, 2.54 mmol, 10.0 eq) were added. The mixture was stirred at 90 °C for 3 h. Then, the resulting reaction mixture was extracted with water and EtOAc (×3), and the combined organic layers were washed with saturated NaHCO_3_ aqueous solution, dried over anhydrous Na_2_SO_4_, filtered, and removed in vacuo to afford the residue, which was purified by flash column chromatography using a mixture of petroleum ether and ethyl acetate to afford **Int-6** (38 mg, yield 59.3%) as a light yellowish brown solid. ^1^H NMR (500 MHz, Chloroform-*d*) *δ* 8.19 (d, *J* = 8.2 Hz, 1 H), 8.15 (d, *J* = 8.1 Hz, 1 H), 7.56–7.51 (m, 2 H), 7.51–7.40 (m, 7 H), 7.40–7.28 (m, 8 H), 5.28 (s, 2 H), 5.17 (s, 2 H), 3.85–3.73 (m, 4 H), and 2.93–2.82 (m, 4 H). LC-MS (ESI): *m*/*z* 526.22 [M + H]^+^.

##### Synthesis of 7,8,9,10-Tetrahydronaphtho[2′,1′:4,5]furo[2,3-c]pyridine-5,6-dione (**S1-1**)

To a solution of **Int-6** (320 mg) in THF (10 mL) and MeOH (10 mL), 10% Pd/C (160 mg) was added. The mixture was purged with hydrogen three times and stirred at room temperature overnight. LC-MS showed that most of the starting material was consumed completely. Then, the resulting reaction mixture was exposed to air for 2 h, filtered through Celite, and the filtrate was removed in vacuo to afford the residue, which was purified by flash column chromatography using a mixture of dichloromethane and methanol to afford **S1-1** (35 mg, yield 22.7%) as a crimson solid. ^1^H NMR (500 MHz, DMSO-*d*_6_) *δ* 7.90 (dd, *J* = 7.7, 1.3 Hz, 1 H), 7.73–7.68 (m, 1 H), 7.65 (dd, *J* = 7.7, 1.3 Hz, 1 H), 7.52–7.47 (m, 1 H), 3.85–3.77 (m, 2 H), 2.89 (t, *J* = 5.5 Hz, 2 H), 2.62–2.57 (m, 2 H), and 2.02–1.94 (m, 1 H). Purity: 96.1%. HRMS (ESI): calcd for C_15_H_11_NO_3_ [M + H]^+^: 254.0812, found: 254.0816. Other data were found in reference [17].

##### Synthesis of 9-Benzyl-7,8,9,10-tetrahydronaphtho[2′,1′:4,5]furo[2,3-c]pyridine-5,6-dione (**S1-2**)

To a solution of **Int-6** (320 mg) in THF (10 mL) and MeOH (10 mL), 10% Pd/C (160 mg) was added. The mixture was purged with hydrogen three times and stirred at room temperature overnight. LC-MS showed that most of the starting material was consumed completely. Then, the resulting reaction mixture was exposed to air for 2 h, filtered through Celite, and the filtrate was removed in vacuo to afford the residue, which was purified by flash column chromatography using a mixture of dichloromethane and methanol to afford **S1-2** (67 mg, yield 32.1%) as a crimson solid. ^1^H NMR (500 MHz, Chloroform-*d*) *δ* 8.00 (d, *J* = 7.7 Hz, 1 H), 7.60–7.53 (m, 2 H), 7.42–7.29 (m, 6 H), 3.85 (s, 2 H), 3.66 (s, 2 H), 2.93–2.88 (m, 2 H), and 2.87–2.83 (m, 2 H). ^13^C NMR (126 MHz, Chloroform-*d*) *δ* 180.7, 175.1, 159.7, 151.1, 136.4, 135.5, 130.6, 129.9, 129.5, 128.8, 128.7, 128.6, 128.0, 122.0, 120.8, 116.3, 61.3, 49.5, 49.0, and 21.1. Purity: 97.0%. HRMS (ESI): calcd for C_22_H_17_NO_3_ [M + H]^+^: 344.1281, found: 344.1288.

##### Synthesis of **S1-3** and **S1-4**

Under the protection of Ar atmosphere, to a solution of **S1-1** (0.09 mmol, 1.0 eq), acid (0.13 mmol, 1.5 eq), and HATU (0.17 mmol, 2.0 eq) in anhydrous DCM (3 mL), DIPEA (0.17 mmol, 2.0 eq) was added. The mixture was stirred at room temperature overnight. Then the resulting reaction mixture was concentrated under reduced pressure to remove DCM to afford the residue, which was purified by flash column chromatography using a mixture of dichloromethane and methanol to afford the desired products.

**S1-3**: 9-(4-Fluorobenzoyl)-7,8,9,10-tetrahydronaphtho[2′,1′:4,5]furo[2,3-*c*]pyridine-5,6-dione. Red solid, yield: 73.6%. ^1^H NMR (500 MHz, Chloroform-*d*) *δ* 8.04 (d, *J* = 7.7 Hz, 1 H), 7.70–7.55 (m, 2 H), 7.53–7.47 (m, 2 H), 7.47–7.42 (m, 1 H), 7.14 (t, *J* = 8.4 Hz, 2 H), 4.97–4.64 (m, 2 H), 3.92–3.45 (m, 2 H), and 2.93–2.82 (m, 2 H). Purity: 95.3%. HRMS (ESI): calcd for C_22_H_14_FNO_4_ [M + H]^+^: 376.0980, found: 376.0983. Other data were found in reference [17].

**S1-4**: 9-(4-Chlorobenzoyl)-7,8,9,10-tetrahydronaphtho[2′,1′:4,5]furo[2,3-*c*]pyridine-5,6-dione. Crimson solid, yield: 58.8%. ^1^H NMR (500 MHz, Chloroform-*d*) *δ* 8.02 (d, *J* = 7.7 Hz, 1 H), 7.67–7.55 (m, 2 H), 7.46–7.39 (m, 5 H), 4.91–4.64 (m, 2 H), 3.82–3.47 (m, 2 H), and 2.92–2.81 (m, 2 H). Purity: 96.0%. HRMS (ESI): calcd for C_22_H_14_ClNO_4_ [M + H]^+^: 392.0684, found: 392.0690. Other data was found in reference [17]

#### 3.2.2. Synthesis of **S2-1** to **S2-9** [15]

##### Synthesis of 3-Methylnaphtho[1,2-b]furan-5-yl acetate (**Int-7**)

To a 250 mL flask, **Int-3** (4.20 g, 21.21 mmol, 1.0 eq), dry DCM (50 mL), triethylamine (8.80 mL, 63.64 mmol, 3.0 eq), and 10 mL of DCM solution of acetyl chloride (2.25 mL, 31.82 mmol, 1.5 eq) were added slowly dropwise at room temperature for 10 min. After completion, the solution was concentrated under *vacuum*, washed with water, and then extracted with EtOAc. The combined organic layers were dried and concentrated. Purification of the residue was accomplished by column chromatography to afford **Int-7** (4.7 g, yield 92.3%) as a yellow solid. ^1^H NMR (500 MHz, Chloroform-*d*) *δ* 8.30 (d, *J* = 8.2 Hz, 1 H), 7.90 (d, *J* = 8.2 Hz, 1 H), 7.63–7.59 (m, 1 H), 7.57 (s, 1 H), 7.54–7.50 (m, 1 H), 7.40 (s, 1 H), 2.49 (s, 3 H), and 2.30 (s, 3 H). LC-MS (ESI): *m*/*z* 241.1 [M + H]^+^.

##### Synthesis of 2-Formyl-3-methylnaphtho[1,2-b]furan-5-yl Acetate (**Int-8**)

DMF (20 mL) and dry DCM (20 mL) were added to a 250 mL flask. Then, POCl_3_ (3.6 mL, 39.17 mmol, 2.0 eq) in 20 mL DCM solution was slowly dropped at 0 °C for 20 min under argon protection. The reaction was carried out at room temperature for 30 min. **Int-7** (4.7 g, 19.58 mmol, 1.0 eq) was added slowly dropwise at room temperature, and the temperature was raised to 70 °C for 12 h. The reaction solution was poured into 200 mL of ice water, filtered, and dried to obtain **Int-8** (4.943 g, yield 93.8%) as a yellow solid. ^1^H NMR (500 MHz, Chloroform-*d*) *δ* 10.06 (s, 1 H), 8.45 (d, *J* = 8.1 Hz, 1 H), 7.94 (d, *J* = 8.1 Hz, 1 H), 7.71–7.67 (m, 1 H), 7.67–7.63 (m, 1 H), 7.44 (s, 1 H), 2.66 (s, 3 H), and 2.50 (s, 3 H). LC-MS (ESI): *m*/*z* 269.1 [M + H]^+^.

##### Synthesis of 5-Acetoxy-3-methylnaphtho[1,2-b]furan-2-carboxylic Acid (**Int-9**)

To a solution of **Int-8** (2.016 g, 7.51 mmol, 1.0 eq) in acetone (60 mL), NaH_2_PO_4_ (2.705 g, 22.54 mmol, 3.0 eq) in H_2_O (20 mL) and 30% H_2_O_2_ (2.303 mL, 22.54 mmol, 3.0 eq) were added. Then, NaClO_2_ (2.549 g, 22.54 mmol, 3.0 eq) in H_2_O (40 mL) was added dropwise to the mixture. The mixture was stirred at room temperature for 2 h. Then, the mixture was concentrated under reduced pressure to remove acetone, followed by addition of ice water (30 mL), and the mixture was allowed to be stirred at room temperature for 1 h. The resulting reaction mixture was filtered and filter cake was collected to afford **Int-9** (1.777 g, yield 83.2%) as a pale yellow solid. LC-MS (ESI): *m*/*z* 283.1 [M − H]^−^.

##### Synthesis of 3-Methyl-4,5-dioxo-4,5-dihydronaphtho[1,2-b]furan-2-carboxylic Acid (**Int-10**)

To a solution of **Int-9** (2.860 g, 10.06 mmol, 1.0 eq) in THF (30 mL), NaOH (1.207 g, 30.18 mmol, 3.0 eq) in H_2_O (10 mL) was added dropwise. The mixture was stirred at 60 °C for 3 h. Then, the mixture was concentrated under reduced pressure to remove THF, followed by addition of acetone (20 mL), H_2_O (5 mL), and IBX (2.986 g, 10.66 mmol, 1.06 eq), and the mixture was allowed to be stirred at room temperature for 3 h. The resulting reaction mixture was extracted with water and EtOAc (×3), and the aqueous phase adjusted the pH to 1–2 with 3 M HCl, was extracted with water and EtOAc (×3), and the combined organic layers were dried over anhydrous Na_2_SO_4_, filtered, and removed in vacuo to afford the residue. To the residue, MeOH (20 mL) was added, filtered, and the filter cake was collected to afford **Int-10** (593 mg, yield 23.0%) as a deep orange solid. LC-MS (ESI): *m*/*z* 255.02 [M − H]^−^.

##### Synthesis of **S2-1** to **S2-9**

To a solution of **Int-10** (1.0 eq) in anhydrous DMF, HATU (1.2 eq) and DIPEA (3.0 eq) were added. After stirring at room temperature for 1 h, amine (1.2 eq) in anhydrous DMF was added dropwise to the mixture. The mixture was stirred at room temperature for 2.5 h. Then, the resulting reaction mixture was extracted with water and EtOAc (×3), and the combined organic layers were dried over anhydrous Na_2_SO_4_, filtered, and removed in vacuo to afford the residue, which was purified by flash column chromatography to afford the desired products.

**S2-1**: *N*,*N*,3-trimethyl-4,5-dioxo-4,5-dihydronaphtho[1,2-*b*]furan-2-carboxamide. Deep orange solid, yield: 66.3%. ^1^H NMR (500 MHz, DMSO-*d*_6_) *δ* 7.97 (d, *J* = 7.7 Hz, 1 H), 7.82–7.74 (m, 2 H), 7.62–7.56 (m, 1 H), 3.18 (s, 3 H), 3.01 (s, 3 H), 2.35 (s, 3 H). ^13^C NMR (126 MHz, DMSO-*d*_6_) *δ* 179.1, 175.1, 159.5, 158.1, 144.3, 135.0, 130.6, 129.8, 129.3, 127.2, 124.7, 122.2, 121.1, and 9.7 (The peak of methyl carbon in the ^13^C NMR spectrum could not be clearly observed due to overlap with the solvent signal of DMSO-*d*_6_). Purity: 99.4%. HRMS (ESI): calcd for C_16_H_13_NO_4_ [M + H]^+^: 284.0917; found: 284.0918.

**S2-2**: 3-Methyl-2-(pyrrolidine-1-carbonyl)naphtho[1,2-*b*]furan-4,5-dione. Orange-red solid, yield: 38.6%. ^1^H NMR (500 MHz, DMSO-*d*_6_) *δ* 7.97 (d, *J* = 7.6 Hz, 1 H), 7.83 (d, *J* = 7.6 Hz, 1 H), 7.80–7.75 (m, 1 H), 7.63–7.58 (m, 1 H), 3.86 (t, *J* = 6.8 Hz, 2 H), 3.50 (t, *J* = 6.8 Hz, 2 H), 2.47 (s, 3 H), 1.98–1.93 (m, 2 H), and 1.89–1.83 (m, 2 H). ^13^C NMR (151 MHz, DMSO-*d*_6_) *δ* 179.1, 175.1, 157.9, 157.6, 144.3, 135.0, 130.7, 130.0, 129.3, 127.2, 126.5, 122.4, 121.2, 47.4, 46.7, 26.1, 23.3, and 10.0. Purity: 97.4%. HRMS (ESI): calcd for C_18_H_15_NO_4_ [M + H]^+^: 310.1074; found: 310.1078.

**S2-3**: 3-Methyl-2-(4-methylpiperazine-1-carbonyl)naphtho[1,2-*b*]furan-4,5-dione. Pinkish orange solid, yield: 83.3%. ^1^H NMR (500 MHz, DMSO-*d*_6_) *δ* 7.97 (d, *J* = 7.7 Hz, 1 H), 7.81–7.75 (m, 2 H), 7.62–7.58 (m, 1 H), 3.63 (t, *J* = 4.9 Hz, 4 H), 2.49–2.38 (m, 4 H), 2.34 (s, 3 H), and 2.25 (s, 3 H). ^13^C NMR (151 MHz, DMSO-*d*_6_) *δ* 179.1, 175.1, 158.4, 158.2, 143.9, 135.1, 130.7, 129.8, 129.4, 127.1, 124.6, 122.3, 121.1, 45.4, and 9.7 (One peak of aliphatic carbon in the ^13^C NMR spectrum could not be clearly resolved due to overlap with the solvent peak of DMSO-*d*_6_). Purity: 96.1%. HRMS (ESI): calcd for C_19_H_18_N_2_O_4_ [M + H]^+^: 339.1339, found: 339.1343.

**S2-4**: *N*-(4-fluorophenyl)-3-methyl-4,5-dioxo-4,5-dihydronaphtho[1,2-*b*]furan-2-carboxamide. Crimson solid, yield: 90.5% as a crimson solid. ^1^H NMR (500 MHz, DMSO-*d*_6_) *δ* 10.33 (s, 1 H), 8.18 (dd, *J* = 7.7, 1.2 Hz, 1 H), 7.99 (dd, *J* = 7.7, 1.2 Hz, 1 H), 7.88–7.84 (m, 1 H), 7.80–7.74 (m, 2 H), 7.66–7.62 (m, 1 H), 7.28–7.22 (m, 2 H), and 2.58 (s, 3 H). ^13^C NMR (126 MHz, DMSO-*d*_6_) *δ* 179.0, 175.1, 158.7 (d, *J* = 241.5 Hz), 158.4, 156.6, 142.6, 134.7, 134.2 (d, *J* = 2.8 Hz), 130.9, 130.1, 129.2, 127.5, 127.1, 123.0 (d, *J* = 7.9 Hz), 123.0, 121.9, 115.3 (d, *J* = 22.3 Hz), and 9.8. ^19^F NMR (471 MHz, DMSO-*d*_6_) *δ* −118.08. Purity: 98.6%. HRMS (ESI): calcd for C_20_H_12_FNO_4_ [M + H]^+^: 350.0823, found: 350.0826.

**S2-5**: *N*-(4-chlorophenyl)-3-methyl-4,5-dioxo-4,5-dihydronaphtho[1,2-*b*]furan-2-carboxamide. Orange solid, yield: 63.0%. ^1^H NMR (500 MHz, DMSO-*d*_6_) *δ* 10.38 (s, 1 H), 8.18 (dd, *J* = 7.7, 1.2 Hz, 1 H), 7.99 (dd, *J* = 7.7, 1.2 Hz, 1 H), 7.89–7.84 (m, 1 H), 7.83–7.79 (m, 2 H), 7.67–7.62 (m, 1 H), 7.48–7.44 (m, 2 H), and 2.58 (s, 3 H). ^13^C NMR (151 MHz, DMSO-*d*_6_) *δ* 179.0, 175.1, 158.5, 156.7, 142.5, 137.0, 134.7, 131.0, 130.2, 129.2, 128.7, 127.9, 127.8, 127.1, 123.2, 122.5, 121.9, and 9.9. Purity: 99.8%. HRMS (ESI): calcd for C_20_H_12_ClNO_4_ [M + H]^+^: 366.0528; found: 366.0533.

**S2-6**: *N*-(3-chlorophenyl)-3-methyl-4,5-dioxo-4,5-dihydronaphtho[1,2-*b*]furan-2-carboxamide. Orange solid, yield: 63.0%. ^1^H NMR (500 MHz, DMSO-*d*_6_) *δ* 10.39 (s, 1 H), 8.19 (d, *J* = 7.7 Hz, 1 H), 8.00 (d, *J* = 7.7 Hz, 1 H), 7.94 (t, *J* = 2.1 Hz, 1 H), 7.89–7.84 (m, 1 H), 7.74 (dd, *J* = 8.1, 2.1 Hz, 1 H), 7.67–7.63 (m, 1 H), 7.43 (t, *J* = 8.1 Hz, 1 H), 7.22 (dd, *J* = 8.1, 2.1 Hz, 1 H), and 2.59 (s, 3 H). ^13^C NMR (151 MHz, DMSO) *δ* 178.9, 175.1, 158.5, 156.8, 142.3, 139.5, 134.7, 133.0, 131.0, 130.5, 130.3, 129.2, 128.1, 127.0, 123.9, 123.2, 121.9, 120.3, 119.2, and 9.9. Purity: 95.1%. HRMS (ESI): calcd for C_20_H_12_ClNO_4_ [M + H]^+^: 366.0528, found: 366.0533.

**S2-7**: *N*-(3-fluorophenyl)-3-methyl-4,5-dioxo-4,5-dihydronaphtho[1,2-*b*]furan-2-carboxamide. Crimson solid, yield: 19.6%. ^1^H NMR (500 MHz, DMSO-*d*_6_) *δ* 10.42 (s, 1 H), 8.19 (d, *J* = 7.6 Hz, 1 H), 8.00 (d, *J* = 7.6 Hz, 1 H), 7.86 (t, *J* = 7.6 Hz, 1 H), 7.73 (d, *J* = 11.6 Hz, 1 H), 7.65 (t, *J* = 7.6 Hz, 1 H), 7.58 (d, *J* = 8.2 Hz, 1 H), 7.48–7.40 (m, 1 H), 7.03–6.96 (m, 1 H), and 2.59 (s, 3 H). ^13^C NMR (201 MHz, DMSO-*d*_6_) *δ* 179.0, 175.1, 162.0 (d, *J* = 241.5 Hz), 158.5, 156.8, 142.4, 139.8 (d, *J* = 10.9 Hz), 134.7, 131.0, 130.4 (d, *J* = 9.3 Hz), 130.3, 129.2, 128.1, 127.0, 123.3, 121.9, 116.5, 110.7 (d, *J* = 20.9 Hz), 107.6 (d, *J* = 26.2 Hz), and 9.9. ^19^F NMR (471 MHz, DMSO-*d*_6_) *δ* −112.11. Purity: 95.2%. HRMS (ESI): calcd for C_20_H_12_FNO_4_ [M + H]^+^: 350.0823, found: 350.0826.

**S2-8**: 3-Methyl-4,5-dioxo-*N*-(prop-2-yn-1-yl)-4,5-dihydronaphtho[1,2-*b*]furan-2-carboxamide. Crimson solid, yield: 87.7%. ^1^H NMR (500 MHz, DMSO-*d*_6_) *δ* 9.11 (t, *J* = 5.7 Hz, 1 H), 8.07 (dd, *J* = 7.6, 1.2 Hz, 1 H), 7.97 (dd, *J* = 7.6, 1.2 Hz, 1 H), 7.85–7.80 (m, 1 H), 7.64–7.58 (m, 1 H), 4.08 (dd, *J* = 5.7, 2.5 Hz, 2 H), 3.18 (t, *J* = 2.5 Hz, 1 H), and 2.52 (s, 3 H). ^13^C NMR (126 MHz, DMSO-*d*_6_) *δ* 179.0, 175.1, 158.3, 157.9, 142.6, 134.7, 130.8, 130.1, 129.2, 127.1, 126.4, 122.9, 121.6, 81.0, 73.1, 27.6, and 9.6. Purity: 95.8%. HRMS (ESI): calcd for C_17_H_11_NO_4_ [M + H]^+^: 294.0761, found: 294.0765.

**S2-9**: 3-Methyl-*N*-(2-(4-methylpiperazin-1-yl)ethyl)-4,5-dioxo-4,5-dihydronaphtho[1,2-*b*]furan-2-carboxamide. Orange-red solid, yield: 43.7%. ^1^H NMR (500 MHz, DMSO-*d*_6_) *δ* 8.55 (t, *J* = 5.8 Hz, 1 H), 8.03 (d, *J* = 7.6 Hz, 1 H), 7.97 (d, *J* = 7.6 Hz, 1 H), 7.83 (t, *J* = 7.6 Hz, 1 H), 7.61 (t, *J* = 7.6 Hz, 1 H), 3.42–3.36 (m, 2 H), 2.53–2.51 (m, 5 H), 2.49–2.44 (m, 4 H), 2.41–2.26 (m, 4 H), and 2.16 (s, 3 H). ^13^C NMR (126 MHz, DMSO-*d*_6_) *δ* 179.1, 175.2, 158.1, 158.0, 143.2, 134.7, 130.7, 130.1, 129.2, 127.2, 125.6, 122.8, 121.7, 56.9, 54.7, 52.6, 45.7, 36.0, and 9.6. Purity: 99.4%. HRMS (ESI): calcd for C_21_H_23_N_3_O_4_ [M + H]^+^: 382.1761 found: 382.1768.

#### 3.2.3. Synthesis of **S2-10** to **S2-14**

To a solution of amide (1.2 eq) in DMSO (3 mL), 3 M KHCO_3_ (4.0 eq) and FSO_3_N_3_ (1.0 eq) were added at room temperature for 2 h. Then, sodium ascorbate (1.0 eq) in H_2_O (0.25 mL), **S2-8** (1.0 eq), and 5 mM CuSO_4_/THPTA (0.7 mL) were added to the mixture. Then, the resulting reaction mixture was extracted with water and EtOAc (×3), and the combined organic layers were dried over anhydrous Na_2_SO_4_, filtered, and removed in vacuo to afford the residue, which was purified to afford the desired products.

**S2-10**: *N*-((1-(2-fluoroethyl)-1*H*-1,2,3-triazol-4-yl)methyl)-3-methyl-4,5-dioxo-4,5-dihydronaph-tho[1,2-*b*]furan-2-carboxamide. Reddish brown solid, yield: 5.1%. ^1^H NMR (500 MHz, DMSO-*d*_6_) *δ* 9.24 (t, *J* = 5.9 Hz, 1 H), 8.08–8.04 (m, 2 H), 7.96 (dd, *J* = 7.7, 1.2 Hz, 1 H), 7.83–7.79 (m, 1 H), 7.62–7.58 (m, 1 H), 4.88–4.84 (m, 1 H), 4.78–4.75 (m, 1 H), 4.72 (t, *J* = 4.7 Hz, 1 H), 4.68–4.65 (m, 1 H), 4.54 (d, *J* = 5.9 Hz, 2 H), and 2.53 (s, 3 H). ^13^C NMR (126 MHz, DMSO-*d*_6_) *δ* 179.0, 175.3, 158.2, 145.0, 142.9, 134.8, 130.8, 130.1, 129.7, 129.2, 127.2, 126.2, 123.5, 123.0, 121.7, 82.0 (d, *J* = 168.8 Hz), 50.1 (d, *J* = 20.2 Hz), 33.9, and 9.7. ^19^F NMR (471 MHz, DMSO-*d*_6_) *δ* −222.00. Purity: 96.6%. HRMS (ESI): calcd for C_19_H_15_FN_4_O_4_ [M + H]^+^: 383.1850, found: 383.1156.

**S2-11**: *N*-((1-(4-fluorophenyl)-1*H*-1,2,3-triazol-4-yl)methyl)-3-methyl-4,5-dioxo-4,5-dihydronaphtho[1,2-*b*]furan-2-carboxamide. Orange solid, yield: 20.4%. ^1^H NMR (500 MHz, DMSO-*d*_6_) *δ* 9.27 (t, *J* = 5.8 Hz, 1 H), 8.71 (s, 1 H), 8.06 (d, *J* = 7.6 Hz, 1 H), 8.01–7.91 (m, 3 H), 7.80 (t, *J* = 7.6 Hz, 1 H), 7.60 (t, *J* = 7.6 Hz, 1 H), 7.43 (t, *J* = 8.6 Hz, 2 H), 4.62 (d, *J* = 5.8 Hz, 2 H), and 2.53 (s, 3 H). ^13^C NMR (151 MHz, DMSO-*d*_6_) *δ* 179.1, 175.2, 161.6 (d, *J* = 245.8 Hz), 158.2, 158.2, 145.9, 142.9, 134.7, 133.2, 130.8, 130.1, 129.2, 127.2, 126.2, 122.9, *δ* 122.3 (d, *J* = 8.8 Hz), 121.7, 121.6, 116.7 (d, *J* = 23.5 Hz), 34.1, and 9.7. ^19^F NMR (471 MHz, DMSO-*d*_6_) *δ* −113.32. Purity: 98.7%. HRMS (ESI): calcd for C_23_H_15_FN_4_O_4_ [M + H]^+^: 431.1150, found: 431.1157.

**S2-12**: *N*-((1-(4-chlorophenyl)-1*H*-1,2,3-triazol-4-yl)methyl)-3-methyl-4,5-dioxo-4,5-dihydronaphtho[1,2-*b*]furan-2-carboxamide. Orange solid, yield: 13.1%. ^1^H NMR (500 MHz, DMSO-*d*_6_) *δ* 9.28 (t, *J* = 5.8 Hz, 1 H), 8.77 (s, 1 H), 8.06 (d, *J* = 7.6 Hz, 1 H), 8.00–7.93 (m, 3 H), 7.80 (t, *J* = 7.6 Hz, 1 H), 7.65 (d, *J* = 8.4 Hz, 2 H), 7.60 (t, *J* = 7.6 Hz, 1 H), 4.62 (d, *J* = 5.8 Hz, 2 H), and 2.53 (s, 3 H). ^13^C NMR (201 MHz, DMSO-*d*_6_) *δ* 179.1, 175.2, 158.3, 158.2, 146.1, 142.9, 135.5, 134.8, 132.9, 130.8, 130.1, 129.9, 129.2, 127.2, 126.2, 123.0, 121.7, 121.4, 33.9, and 9.7. Purity: 97.9%. HRMS (ESI): calcd for C_23_H_15_ClN_4_O_4_ [M + H]^+^: 447.0855, found: 447.0861.

**S2-13**: *N*-((1-(3-chlorophenyl)-1*H*-1,2,3-triazol-4-yl)methyl)-3-methyl-4,5-dioxo-4,5-dihydronaphtho[1,2-*b*]furan-2-carboxamide. Orange solid, yield: 48.1%. ^1^H NMR (500 MHz, DMSO-*d*_6_) *δ* 9.28 (t, *J* = 5.8 Hz, 1 H), 8.83 (s, 1 H), 8.09–8.03 (m, 2 H), 7.98–7.92 (m, 2 H), 7.80 (t, *J* = 7.6 Hz, 1 H), 7.60 (td, *J* = 7.8, 4.3 Hz, 2 H), 7.54 (d, *J* = 8.0 Hz, 1 H), 4.63 (d, *J* = 5.8 Hz, 2 H), and 2.54 (s, 3 H). ^13^C NMR (126 MHz, DMSO-*d*_6_) *δ* 179.1, 175.1, 158.2, 158.2, 146.2, 142.9, 137.7, 134.7, 134.2, 131.6, 130.8, 130.1, 129.2, 128.4, 127.2, 126.2, 122.9, 121.7, 121.5, 119.7, 118.5, 33.9, and 9.7. Purity: 98.1%. HRMS (ESI): calcd for C_23_H_15_ClN_4_O_4_ [M + H]^+^: 447.0855, found: 447.0862.

**S2-14**: *N*-((1-(3-fluorophenyl)-1*H*-1,2,3-triazol-4-yl)methyl)-3-methyl-4,5-dioxo-4,5-dihydronaphtho[1,2-*b*]furan-2-carboxamide. Orange solid, yield: 56.8%. ^1^H NMR (500 MHz, DMSO-*d*_6_) *δ* 9.31–9.23 (m, 1 H), 8.80 (s, 1 H), 8.08–8.02 (m, 1 H), 7.95 (d, *J* = 7.7 Hz, 1 H), 7.89–7.83 (m, 1 H), 7.85–7.76 (m, 2 H), 7.67–7.56 (m, 2 H), 7.36–7.29 (m, 1 H), 4.63 (d, *J* = 5.8 Hz, 2 H), and 2.53 (s, 3 H). ^13^C NMR (126 MHz, DMSO-*d*_6_) *δ* 179.1, 175.1, 162.5 (d, *J* = 245.1 Hz), 158.3, 158.2, 146.1, 142.9, 137.9 (d, *J* = 10.6 Hz), 134.8, 131.9 (d, *J* = 9.1 Hz), 130.8, 130.1, 129.2, 127.2, 126.2, 123.0, 121.7, 121.5, 115.9 (d, *J* = 3.0 Hz), 115.3 (d, *J* = 20.9 Hz), 107.4 (d, *J* = 26.7 Hz), 33.9, and 9.7. ^19^F NMR (471 MHz, DMSO-*d*_6_) *δ* −110.60. Purity: 97.4%. HRMS (ESI): calcd for C_23_H_15_FN_4_O_4_ [M + H]^+^: 431.1150, found: 431.1157.

#### 3.2.4. Synthesis of **S3-1** to **S3-14** [19,23,24]

To a solution of diketone compounds (1.0 eq), NH_4_OAc (26.0 eq) in AcOH (12 mL) was added to aromatic aldehyde compounds (1.5 eq). The reaction mixture was stirred at 120 °C for 0.5 h in a microwave. Then, the resulting reaction mixture was quenched with saturated aqueous sodium bicarbonate, extracted with EtOAc (×3), and the combined organic layers were washed with saturated aqueous sodium bicarbonate, dried over anhydrous MgSO_4_, filtered, and removed in vacuo to afford the residue, which was purified to afford the titled compound.

**S3-1**: 11-(3-Methoxyphenyl)-1,6,6-trimethyl-7,8,9,10-tetrahydro-6*H*-furo[2′,3′:1,2]phenanthro[3,4-*d*]imidazole. Yellow solid, yield: 52.6%. ^1^H NMR (500 MHz, Chloroform-*d*) *δ* 10.11 (s, 1 H), 8.23 (d, *J* = 8.6 Hz, 1 H), 7.70 (t, *J* = 2.0 Hz, 1 H), 7.63–7.59 (m, 2 H), 7.56 (s, 1 H), 7.43 (t, *J* = 7.9 Hz, 1 H), 6.98 (dd, *J* = 8.2, 2.0 Hz, 1 H), 3.94 (s, 3 H), 3.65–3.27 (m, 2 H), 2.69 (s, 3 H), 2.14–2.07 (m, 2 H), 1.85–1.81 (m, 2 H), and 1.43 (s, 6 H). Purity: 97.9%. HRMS (ESI): calcd for C_27_H_26_N_2_O_2_ [M + H]^+^: 411.2067, found: 411.2076. Other data were found in reference [25].

**S3-2**: 11-(2,3-Dichlorophenyl)-1,6,6-trimethyl-7,8,9,10-tetrahydro-6*H*-furo[2′,3′:1,2]phenanthro[3,4-*d*]imidazole. Yellow solid, yield: 57.0%. ^1^H NMR (500 MHz, DMSO-*d*_6_) *δ* 13.22 (s, 1 H), 8.10 (d, *J* = 8.6 Hz, 1 H), 7.91 (s, 1 H), 7.86–7.82 (m, 2 H), 7.64 (d, *J* = 8.6 Hz, 1 H), 7.58 (d, *J* = 8.0 Hz, 1 H), 3.83 (t, *J* = 6.4 Hz, 2 H), 2.52 (s, 3 H), 1.94–1.87 (m, 2 H), 1.76–1.72 (m, 2 H), and 1.37 (s, 6 H). ^13^C NMR (126 MHz, DMSO) *δ* 148.9, 144.4, 142.7, 141.3, 135.8, 133.2, 132.6, 131.2, 131.1, 131.0, 130.0, 129.7, 128.2, 128.2, 125.0, 123.9, 122.7, 117.8, 115.2, 38.5, 34.3, 31.9, 31.7, 26.6, and 9.4. Purity: 98.6%. HRMS (ESI): calcd for C_26_H_22_Cl_2_N_2_O [M + H]^+^: 449.1182 found: 449.1194.

**S3-3**: 11-(4-Bromophenyl)-1,6,6-trimethyl-7,8,9,10-tetrahydro-6*H*-furo[2′,3′:1,2]phenanthro[3,4-*d*]imidazole. Dark brown solid, yield: 66.2%. ^1^H NMR (500 MHz, Chloroform-*d*) *δ* 10.06 (s, 1 H), 8.22 (d, *J* = 8.6 Hz, 1 H), 7.96–7.92 (m, 2 H), 7.64–7.59 (m, 3 H), 7.55 (s, 1 H), 3.68–3.36 (m, 2 H), 2.67 (s, 3 H), 2.13–2.07 (m, 2 H), 1.85–1.80 (m, 2 H), and 1.43 (s, 6 H). Purity: 99.4%. HRMS (ESI): calcd for C_26_H_23_BrN_2_O [M + H]^+^: 459.1067 found: 459.1080. Other data were found in reference [25].

**S3-4**: 1,6,6-Trimethyl-11-(p-tolyl)-7,8,9,10-tetrahydro-6*H*-furo[2′,3′:1,2]phenanthro[3,4-*d*]imid-azole. Brownish black solid, yield: 59.7%. ^1^H NMR (500 MHz, Chloroform-*d*) *δ* 10.09 (s, 1 H), 8.23 (d, *J* = 8.6 Hz, 1 H), 7.99 (d, *J* = 7.7 Hz, 2 H), 7.60 (d, *J* = 8.6 Hz, 1 H), 7.56 (s, 1 H), 7.33 (d, *J* = 7.7 Hz, 2 H), 3.68–3.33 (m, 2 H), 2.70 (s, 3 H), 2.43 (s, 3 H), 2.15–2.07 (m, 2 H), 1.85–1.80 (m, 2 H), and 1.43 (s, 6 H). Purity: 98.7%. HRMS (ESI): calcd for C_27_H_26_BrN_2_O [M + H]^+^: 395.2118 found: 395.2127. Other data were found in reference [25].

**S3-5**: 11-(3,5-Difluorophenyl)-1,6-dimethyl-10*H*-furo[2′,3′:1,2]phenanthro[3,4-*d*]imidazole. Off-white solid, yield: 60.1%. ^1^H NMR (500 MHz, DMSO-*d*_6_) *δ* 13.02 (s, 1 H), 10.74 (d, *J* = 8.5 Hz, 1 H), 8.33 (d, *J* = 9.1 Hz, 1 H), 8.20–8.09 (m, 3 H), 8.00 (s, 1 H), 7.75–7.69 (m, 1 H), 7.52 (d, *J* = 7.0 Hz, 1 H), 7.43–7.36 (m, 1 H), 2.77 (s, 3 H), and 2.61 (s, 3 H). ^13^C NMR (126 MHz, DMSO) *δ* 162.8 (dd, *J* = 13.9, 245.7 Hz), 149.2, 146.4, 142.1, 136.4, 133.9, 133.7 (t, 9.6 Hz), 130.5, 130.1, 127.1, 126.6, 126.5, 126.4, 122.1, 119.1, 118.8, 116.0, 115.4, 112.6, 109.7 (dd, 7.6, 21.4 Hz), 104.5 (t, 25.2 Hz), 19.9, and 9.7. ^19^F NMR (471 MHz, DMSO-*d*_6_) *δ* -109.19. Purity: 98.5%. HRMS (ESI): calcd for C_25_H_16_F_2_N_2_O [M + H]^+^: 399.1303 found: 399.1313.

**S3-6**: 11-(4-Bromophenyl)-1,6-dimethyl-10*H*-furo[2′,3′:1,2]phenanthro[3,4-*d*]imidazole. Cream-colored solid, yield: 71.0%. ^1^H NMR (500 MHz, DMSO-*d*_6_) *δ* 13.11 (s, 1 H), 10.82 (d, *J* = 8.6 Hz, 1 H), 8.42 (d, *J* = 8.5 Hz, 2 H), 8.38 (d, *J* = 9.1 Hz, 1 H), 8.15 (d, *J* = 9.1 Hz, 1 H), 8.04 (s, 1 H), 7.86 (d, *J* = 8.5 Hz, 2 H), 7.73–7.69 (m, 1 H), 7.54 (d, *J* = 6.9 Hz, 1 H), 2.79 (s, 3 H), and 2.66 (s, 3 H). ^13^C NMR (126 MHz, DMSO-*d*_6_) *δ* 148.9, 147.9, 142.1, 136.5, 134.0, 131.8, 130.5, 130.2, 129.6, 128.9, 127.1, 126.6, 126.3, 122.7, 122.0, 119.1, 118.9, 115.8, 115.4, 112.7, 19.9, and 9.7. Purity: 99.1%. HRMS (ESI): calcd for C_25_H_17_BrN_2_O [M + H]^+^: 441.0597 found: 441.0611.

**S3-7**: 11-(4-Bromo-2-fluorophenyl)-1,6-dimethyl-10*H*-furo[2′,3′:1,2]phenanthro[3,4-*d*]imidazole. Off-white solid, yield: 46.1%. ^1^H NMR (500 MHz, DMSO-*d*_6_) *δ* 13.20 (s, 1 H), 10.75 (d, *J* = 8.6 Hz, 1 H), 8.38 (d, *J* = 9.1 Hz, 1 H), 8.21–8.14 (m, 2 H), 8.05 (s, 1 H), 7.89 (d, *J* = 10.2 Hz, 1 H), 7.72 (d, *J* = 8.4 Hz, 1 H), 7.70–7.65 (m, 1 H), 7.53 (d, *J* = 7.0 Hz, 1 H), 2.79 (s, 3 H), and 2.62 (s, 3 H). ^13^C NMR (126 MHz, DMSO) *δ* 159.3 (d, *J* = 255.6 Hz), 149.0, 143.4, 142.0, 136.4, 133.9, 132.3 (d, *J* = 3.2 Hz), 130.5, 130.2, 128.1 (d, *J* = 3.5 Hz), 127.1, 126.6, 126.2, 126.1, 122.9 (d, *J* = 9.4 Hz), 121.9, 119.9 (d, *J* = 25.0 Hz), 119.2, 118.8, 118.5 (d, *J* = 12.3 Hz), 115.8, 115.3, 112.6, 19.9, and 9.5. ^19^F NMR (471 MHz, DMSO-*d*_6_) *δ* −110.89. Purity: 98.6%. HRMS (ESI): calcd for C_25_H_16_BrFN_2_O [M + H]^+^: 459.0503 found: 459.0516.

**S3-8**: 11-(4-Methoxyphenyl)-1,6-dimethyl-10*H*-furo[2′,3′:1,2]phenanthro[3,4-*d*]imidazole. Cream-colored solid, yield: 79.8%. ^1^H NMR (500 MHz, DMSO-*d*_6_) *δ* 12.90 (s, 1 H), 10.87 (d, *J* = 8.6 Hz, 1 H), 8.39 (d, *J* = 8.8 Hz, 2 H), 8.37 (d, *J* = 9.1 Hz, 1 H), 8.12 (d, *J* = 9.1 Hz, 1 H), 8.02 (s, 1 H), 7.73–7.68 (m, 1 H), 7.52 (d, *J* = 7.0 Hz, 1 H), 7.20 (d, *J* = 8.6 Hz, 2 H), 3.89 (s, 3 H), 2.79 (s, 3 H), and 2.66 (s, 3 H). ^13^C NMR (126 MHz, DMSO-*d*_6_) *δ* 160.3, 149.2, 148.6, 141.9, 136.5, 133.8, 130.4, 130.4, 128.6, 127.0, 126.8, 126.2, 126.0, 123.1, 121.6, 119.0, 118.9, 115.5, 115.3, 114.2, 112.8, 55.4, 19.9, and 9.8. Purity: 99.0%. HRMS (ESI): calcd for C_26_H_20_N_2_O_2_ [M + H]^+^: 393.1598 found: 393.1607.

**S3-9**: 11-(2,3-Dichlorophenyl)-1,6-dimethyl-10*H*-furo[2′,3′:1,2]phenanthro[3,4-*d*]imidazole. Off-white solid, yield: 34.2%. ^1^H NMR (500 MHz, DMSO-*d*_6_) *δ* 13.49 (s, 1 H), 10.69 (d, *J* = 8.6 Hz, 1 H), 8.39 (d, *J* = 9.1 Hz, 1 H), 8.15 (d, *J* = 9.1 Hz, 1 H), 8.05 (s, 1 H), 7.93 (dd, *J* = 7.6, 1.5 Hz, 1 H), 7.90 (dd, *J* = 8.1, 1.5 Hz, 1 H), 7.66–7.60 (m, 2 H), 7.51 (d, *J* = 7.0 Hz, 1 H), 2.78 (s, 3 H), and 2.59 (s, 3 H). ^13^C NMR (126 MHz, DMSO-*d*_6_) *δ* 148.8, 146.2, 142.1, 136.0, 133.9, 133.0, 132.6, 131.5, 131.3, 131.0, 130.4, 130.1, 128.4, 127.1, 126.6, 126.3, 125.6, 121.9, 119.4, 118.9, 115.7, 115.3, 112.7, 19.9, and 9.4. Purity: 95.8%. HRMS (ESI): calcd for C_25_H_16_Cl_2_N_2_O [M + H]^+^: 431.0712 found: 431.0724.

**S3-10**: 2-(4-Bromophenyl)-4-methyl-1*H*-furo[3′,2′:3,4]naphtho[1,2-*d*]imidazole. Dark brown solid, yield: 36.6%. ^1^H NMR (500 MHz, DMSO-*d*_6_) *δ* 13.53 (s, 1 H), 8.57 (d, *J* = 8.0 Hz, 1 H), 8.28 (d, *J* = 8.5 Hz, 1 H), 8.26–8.23 (m, 2 H), 7.94 (s, 1 H), 7.84–7.80 (m, 2 H), 7.68–7.63 (m, 1 H), 7.63–7.58 (m, 1 H), and 2.61 (s, 3 H). ^13^C NMR (126 MHz, DMSO-*d*_6_) *δ* 148.4, 147.4, 141.6, 135.3, 131.9, 131.7, 129.7, 128.5, 128.0, 126.6, 125.2, 125.0, 122.5, 122.2, 120.5, 119.1, 118.6, 117.4, 116.4, and 9.4. Purity: 98.8%. HRMS (ESI): calcd for C_20_H_13_BrN_2_O [M + H]^+^: 377.0284 found: 377.0293.

**S3-11**: 2-(3-Methoxyphenyl)-4-methyl-1*H*-furo[3′,2′:3,4]naphtho[1,2-*d*]imidazole. Brown solid, yield: 27.5%. ^1^H NMR (500 MHz, DMSO-*d*_6_) *δ* 13.40 (s, 1 H), 8.58 (d, *J* = 8.1 Hz, 1 H), 8.27 (d, *J* = 8.3 Hz, 1 H), 7.93 (s, 1 H), 7.91–7.86 (m, 2 H), 7.85 (t, *J* = 2.0 Hz, 1 H), 7.67–7.62 (m, 1 H), 7.61–7.57 (m, 1 H), 7.51 (t, *J* = 7.9 Hz, 1 H), 7.07 (dd, *J* = 8.2, 2.0 Hz, 1 H), 3.90 (s, 3 H), and 2.62 (s, 3 H). ^13^C NMR (126 MHz, DMSO-*d*_6_) *δ* 148.4, 147.4, 141.6, 135.3, 131.9, 131.7, 129.7, 128.5, 128.0, 126.6, 125.2, 125.0, 122.5, 122.2, 120.5, 119.1, 118.6, 117.4, 116.4, and 9.4. Purity: 97.6%. HRMS (ESI): calcd for C_21_H_16_N_2_O_2_ [M + H]^+^: 329.1285 found: 329.1288.

**S3-12**: 2-(3,5-Difluorophenyl)-4-methyl-3a,10b-dihydro-1*H*-furo[3′,2′:3,4]naphtho[1,2-*d*]imidazole. Off-white solid, yield: 52.1%. ^1^H NMR (500 MHz, DMSO-*d*_6_) *δ* 13.58 (s, 1 H), 8.52 (d, *J* = 8.1 Hz, 1 H), 8.28 (d, *J* = 7.9 Hz, 1 H), 8.07–7.92 (m, 3 H), 7.69–7.65 (m, 1 H), 7.64–7.60 (m, 1 H), 7.40–7.35 (m, 1 H), and 2.60 (s, 3 H). ^13^C NMR (126 MHz, DMSO) *δ* 162.8 (dd, *J* = 12.6, 245.7 Hz), 147.5, 141.6, 135.2 (t, 19.5 Hz), 133.7, 126.8, 125.3, 123.9, 122.3, 122.2, 120.5, 119.0, 118.9, 117.3, 116.4, 109.0 (dd, 4.8, 25.4 Hz), 104.3 (t, *J* = 21.4), and 9.3. ^19^F NMR (471 MHz, DMSO-*d*_6_) *δ* -109.07. Purity: 97.1%. HRMS (ESI): calcd for C_20_H_14_F_2_N_2_O [M + H]^+^: 337.1147 found: 337.1149.

**S3-13**: 2-(4-Bromo-2-fluorophenyl)-4-methyl-3a,10b-dihydro-1*H*-furo[3′,2′:3,4]naphtho[1,2-*d*]imidazole. Light yellowish brown solid, yield: 52.1%. ^1^H NMR (500 MHz, DMSO-*d*_6_) *δ* 13.40 (s, 1 H), 8.69–8.64 (m, 1 H), 8.30–8.27 (m, 1 H), 8.14 (t, *J* = 8.2 Hz, 1 H), 7.94 (s, 1 H), 7.86 (dd, *J* = 10.4, 1.8 Hz, 1 H), 7.67 (dd, *J* = 8.4, 1.8 Hz, 1 H), 7.64–7.60 (m, 2 H), and 2.59 (s, 3 H). ^13^C NMR (126 MHz, DMSO-*d*_6_) *δ* 158.9 (d, *J* = 256.4 Hz), 147.5, 143.9, 141.5, 135.1, 131.8, 128.2, 126.7, 125.2, 158.9 (d, *J* = 256.4 Hz), 122.6, 120.4, 119.8 (d, *J* = 25.1 Hz), 119.2, 118.8, 118.32, 118.2, 117.3, 116.4, and 9.4. ^19^F NMR (471 MHz, DMSO-*d*_6_) *δ* −111.30. Purity: 96.3%. LC-MS (ESI): *m*/*z* 396.88 [M + H]^+^.

**S3-14**: 4-Methyl-2-(*p*-tolyl)-1*H*-furo[3′,2′:3,4]naphtho[1,2-*d*]imidazole. Brownish solid, yield: 41.8%. ^1^H NMR (500 MHz, DMSO-*d*_6_) *δ* 13.35 (s, 1 H), 8.58 (d, *J* = 8.2 Hz, 1 H), 8.27 (d, *J* = 8.4 Hz, 1 H), 8.19 (d, *J* = 8.1 Hz, 2 H), 7.92 (s, 1 H), 7.66–7.61 (m, 1 H), 7.60–7.55 (m, 1 H), 7.40 (d, *J* = 8.1 Hz, 2 H), 2.61 (s, 3 H), and 2.40 (s, 3 H). ^13^C NMR (126 MHz, DMSO-*d*_6_) *δ* 149.7, 147.3, 141.5, 138.8, 135.3, 129.5, 129.3, 127.8, 126.6, 126.3, 126.1, 125.1, 124.7, 122.2, 120.4, 119.1, 118.3, 117.5, 116.4, 21.0, and 9.4. Purity: 96.8%. HRMS (ESI): calcd for C_21_H_16_N_2_O [M + H]^+^: 313.1335 found: 313.1339.

### 3.3. In Vitro Cytotoxicity Assay

Human normal bronchial epithelial cells (BEAS-2B) and human lung adenocarcinoma cell lines (A549 and H838) were purchased from the Cell Bank of the Chinese Academy of Sciences (Shanghai, China). The cells were cultured in RPMI-1640 medium (for BEAS-2B) or DMEM (for A549 and H838), supplemented with 10% fetal bovine serum (Vazyme, Nanjing, China) and 1% penicillin/streptomycin (NCM Biotech, Suzhou, China) at 37 °C in a humidified incubator with 5% CO_2_. Cytotoxicity was evaluated using the CCK-8 assay. Briefly, A549, H838, and BEAS-2B cells were seeded into 96-well plates at a density of 6000 cells per well and allowed to adhere overnight. The following day, the culture medium was replaced with fresh medium containing various concentrations of the test compounds. After 24 h of incubation, 10% (*v*/*v*) CCK-8 solution (GLPBIO, Montclair, CA, USA) was added to each well. The optical density (OD) at 450 nm was measured using a microplate reader. The cell viability curve was plotted, and the half-maximal inhibitory concentration (IC_50_) was calculated using GraphPad Prism software (version 10.4.0; GraphPad Software, San Diego, CA, USA).

#### 3.3.1. Colony Formation Assay

A549 and H838 cells were plated in 6-well plates at a low density of 2000 cells per well and cultured for 14 days. The compounds were administered every three days. Subsequently, the formed cell colonies were fixed with 4% paraformaldehyde for 30 min and stained with 0.1% crystal violet solution. The number of visible colonies was then recorded.

#### 3.3.2. Wound Healing Assay

A549 and H838 cells were grown in 6-well plates to approximately 90% confluence under standard conditions. The cells were pretreated with 5 μg/mL mitomycin C (Selleck, Shanghai, China) for 2 h to inhibit proliferation. Subsequently, a sterile 10 μL pipette tip was used to create a straight scratch wound on the monolayer. After being gently washed three times with phosphate-buffered saline (PBS) to remove detached cells, serum-free medium was added. Cell migration into the wound area was observed and photographed under a microscope at 0, 24, and 48 h. The migration rate was quantified by measuring the change in the wound width over time.

### 3.4. Statistical Analysis

All experiments were independently repeated three times. IC_50_ values were obtained by fitting semi-logarithmic dose–response curves. All data were processed using GraphPad Prism 10.4, and results are expressed as mean ± SD. Differences among groups were first analyzed by one-way analysis of variance (ANOVA), followed by pairwise comparisons using the LSD-*t* test; Student’s *t*-test was used for direct comparisons between two groups. The significance level was set at two-sided *p* < 0.05.

### 3.5. Molecular Docking

The X-ray crystal structures of STAT3 and NLRP3 were downloaded from the RCSB Protein Data Bank (PDB IDs: 1BG1 and 7ALV). First, the Protein Preparation Wizard was used to obtain the optimal structures of the STAT3 and NLRP3 in Schrödinger 2021-2, including removal of water molecules, hydrogenation, minimization of energy, and treatment of metal ions and disulfide bonds. Then, the optimal conformation of a small molecule, including the formation of a three-dimensional (3D) structure and low-energy conformations, was created with the Lig Prep module. The pocket grid points were generated by the Receptor Grid Generation module in the software. Finally, we used the Ligand Docking module to mimic the interaction between the STAT3 and NLRP3 proteins and the small molecules. The docked poses were visualized using PyMOL(version 2.6.0a0) and the 2D Sketcher module of the Schrödinger software (version 2021-2).

## 4. Conclusions

Based on the analysis of the interactions between the reported tanshinone derivatives and their corresponding target proteins and the scaffold-hopping strategy, 35 novel tanshinone derivatives were designed and synthesized in this study. Through in vitro activity evaluation, our work provides promising candidate compounds and insights into structural optimization for the treatment of NSCLC. Firstly, the activity screening results indicated that Series 2 compounds, featuring a benzofuran scaffold with a vicinal quinone group, exhibited superior inhibitory activity against A549 and H838 cell lines compared to Series 1 (naphthoquinone–furan–piperidine scaffold) and Series 3 (lacking the *ortho*-quinone group). Among them, **S2-4** and **S2-8** exhibited IC_50_ values of 0.58 ± 0.07 μM and 0.42 ± 0.04 μM against H838 cells, respectively, outperforming the positive control **β-lapachone** and showing potent cytotoxicity. In A549 cells, **S2-1**, **S2-4**, and **S2-8** still maintained low IC_50_ values (~2.4–2.5 μM), indicating specific selectivity for H838 cells. In addition, the selected active compounds significantly suppressed the colony formation and migration abilities of both A549 and H838 cells, and their in vitro antitumor activity was comparable to that of the positive control. Secondly, the structure–activity trends analysis identified the key pharmacophoric moieties: the benzofuran core bearing an *ortho*-quinone group served as the essential scaffold for enhancing the anti-NSCLC activity of tanshinone derivatives. The introduction of amide bonds and 1,2,3-triazole groups at the C-2 position of the furan ring further improved the cellular selectivity and binding stability of the compounds. However, the structure–activity trend conclusions presented in this study are based, to some extent, on limited quantitative data and thus cannot establish definitive activity–structure relationships. The current work may provide guidance for the future design of analogs with improved potency and selectivity. It is worth noting that, although compounds in Series 3 (without the *ortho*-quinone moiety) showed weak inhibitory activity against cancer cells, their cytotoxicity toward normal bronchial epithelial cells (BEAS-2B) was significantly reduced, providing a valuable direction for the subsequent development of low-toxicity tanshinone derivatives. Molecular docking predicted the binding modes of the active compounds with potential targets, which may help explain their favorable biological activities. The docking results showed that the introduction of an amide bond enabled additional hydrogen-bond interactions with key residues, while the heteroaromatic moieties participated in favorable π–cation and hydrophobic interactions within the binding pockets of STAT3 and NLRP3. These cooperative, noncovalent interactions facilitated the stable binding orientations of **S2-14** and **S2-4**, which may account for their enhanced biological activities. Overall, the docking results support the rational design strategy and provide a reasonable explanation for the activity evaluation.

In conclusion, through rational structural design and systematic activity evaluation, this study clarifies the optimization direction of tanshinone derivatives, laying a solid experimental foundation for the development of highly efficient and selective anti-NSCLC drugs. Future work should include in vivo pharmacodynamic evaluation, pharmacokinetic studies, and more in-depth exploration of the mechanism of action, which might be conducted to promote the translation of this class of compounds into clinical applications.

## Figures and Tables

**Table 1:** The structures and inhibitory rates after treating cancer cell lines with 5 μmol/L and 1 μmol/L of Series 1-related compounds, respectively. Data is presented as the mean ± standard deviation (SD) of three independent experiments.

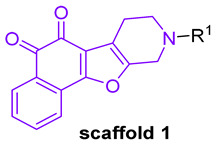							
Cpd	R^1^	Inhibition Rate (%)					
		A549 *^a^*		H838 *^b^*		BEAS-2B *^c^*	
		C = 5 μM	C = 1 μM	C = 5 μM	C = 1 μM	C = 5 μM	C = 1 μM
**S1-1**	H	15.66 ± 5.85	11.01 ± 1.13	32.24 ± 3.48	24.33 ± 8.16	76.32 ± 4.28	23.45 ± 8.68
**S1-2**	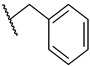	23.32 ± 7.41	10.14 ± 0.14	29.87 ± 12.19	25.44 ± 18.27	71.97 ± 2.71	23.31 ± 9.78
**S1-3**	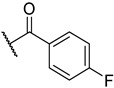	13.75 ± 0.09	10.37 ± 2.16	23.13 ± 4.05	27.9 ± 4.51	69.66 ± 7.36	20.64 ± 8.74
**S1-4**	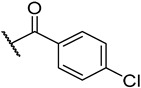	17.48 ± 11.17	10.27 ± 0.54	25.47 ± 6.79	27.44 ± 6.66	77.26 ± 6.31	22.21 ± 13.71
**Int-3**	-	16.73 ± 7.39	9.8 ± 1.74	22.87 ± 9.81	30.35 ± 8.9	24.12 ± 7.09	18.83 ± 5.71
**Int-4**	-	17.19 ± 3.58	5.78 ± 0.43	42.92 ± 10.81	26.33 ± 6.85	73.01 ± 6.2	20.55 ± 2.62
**Int-5**	-	13.04 ± 4.31	10.12 ± 4.39	22.16 ± 2.52	33.29 ± 8.86	15.86 ± 7.23	19.52 ± 5.73
**β-lapachone**	-	82.18 ± 1.82	10.19 ± 1.26	97.92 ± 0.08	36.96 ± 3.92	70.81 ± 15.09	78.16 ± 2.04

*^a^* The data represent the average of two independent experiments; *^b^*^,*c*^ the data represent the average of three independent experiments.

**Table 2:** The structures and inhibitory rates after treating cancer cell lines with 5 μmol/L and 1 μmol/L of Series 2-related compounds, respectively. Data is presented as the mean ± SD of three independent experiments.

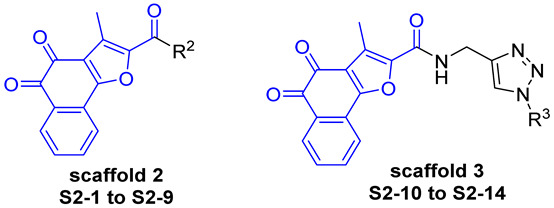							
Cpd	R^2^/R^3^	Inhibition Rate (%)					
		A549 *^a^*		H838 *^b^*		BEAS-2B *^c^*	
		C = 5 μM	C = 1 μM	C = 5 μM	C = 1 μM	C = 5 μM	C = 1 μM
**S2-1**	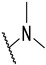	84.25 ± 1.63	1.37 ± 3.27	98.72 ± 0.13	37.82 ± 2.27	85.22 ± 6.78	77.2 ± 3.53
**S2-2**	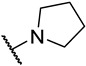	1.71 ± 6.43	1.57 ± 1.98	11.07 ± 4.63	28.67 ± 1.36	84.85 ± 7.74	47.23 ± 34.77
**S2-3**	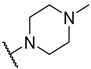	70.2 ± 2.86	0.93 ± 0.73	98.78 ± 0.15	39.94 ± 1.38	82.39 ± 8.97	79.56 ± 2.97
**S2-4**	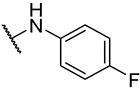	85.39 ± 1.44	8.14 ± 1.98	98.63 ± 0.06	45.95 ± 6.09	91.04 ± 4.86	79.38 ± 1.46
**S2-5**	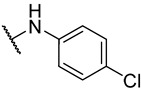	−1.95 ± 6.6	5.57 ± 2.09	17.6 ± 0.85	27.95 ± 3.62	82.11 ± 4.99	30.62 ± 10.27
**S2-6**	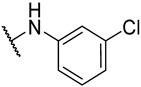	3.6 ± 1.33	6.78 ± 1.73	19.52 ± 1.44	31.04 ± 2.35	46.45 ± 35.14	24.61 ± 0.28
**S2-7**	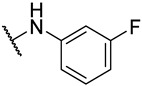	2.58 ± 1	8.61 ± 1.55	16.39 ± 2.97	30.18 ± 5.09	29.11 ± 56.5	29.33 ± 7.43
**S2-8**	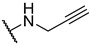	83.79 ± 1.3	6.79 ± 2.04	98.24 ± 0.08	62.7 ± 6.12	86.4 ± 0.51	74.69 ± 2.23
**S2-9**	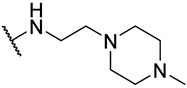	33.75 ± 0.51	7.01 ± 4.69	46.26 ± 8.14	32.05 ± 3.39	77.07 ± 5.59	24.54 ± 3.59
**S2-10**	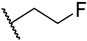	5.28 ± 5.13	1.28 ± 1.26	31.06 ± 3.9	33.84 ± 6.81	79.55 ± 2.37	28.22 ± 9.44
**S2-11**	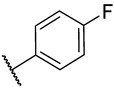	6.08 ± 3.34	1.3 ± 2.35	15.1 ± 3.9	30.73 ± 7.01	78.77 ± 1.3	25.87 ± 0.94
**S2-12**	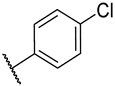	6.66 ± 3.5	2.02 ± 1.68	16.57 ± 4.49	27.5 ± 3.23	78.77 ± 4.3	29.63 ± 4.76
**S2-13**	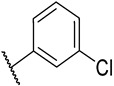	52.62 ± 0.29	7.32 ± 3.33	98.83 ± 0.3	35.85 ± 7.26	76.54 ± 3.38	63.07 ± 32.86
**S2-14**	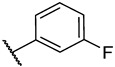	13.47 ± 9.17	4.26 ± 3.24	96.39 ± 3.14	35.01 ± 6.9	78.14 ± 11.3	61.08 ± 38.49
**Int-4**	-	17.19 ± 3.58	5.78 ± 0.43	42.92 ± 10.81	26.33 ± 6.85	73.01 ± 6.2	20.55 ± 2.62
**β-lapachone**	-	82.18 ± 1.82	10.19 ± 1.26	97.92 ± 0.08	36.96 ± 3.92	70.81 ± 15.09	78.16 ± 2.04

*^a^*^,^*^b^* The data represent the average of two independent experiments; *^c^* the data represent the average of three independent experiments.

**Table 3:** The structures and inhibitory rates after treating cancer cell lines with 5 μmol/L and 1 μmol/L of Series 3-related compounds, respectively. Data is presented as the mean ± SD of three independent experiments.

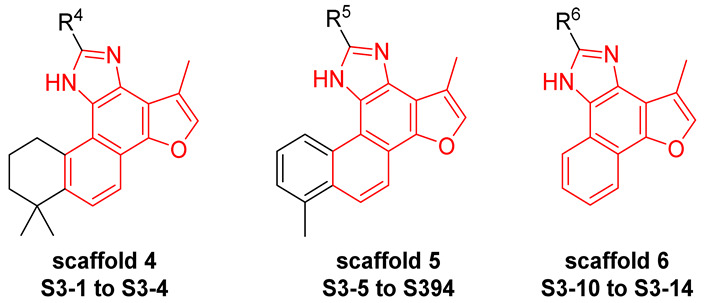							
Cpd	R^4^/R^5^/R^6^	Inhibition Rate (%)					
		A549 *^a^*		H838 *^b^*		BEAS-2B *^c^*	
		C = 5 μM	C = 1 μM	C = 5 μM	C = 1 μM	C = 5 μM	C = 1 μM
**S3-1**	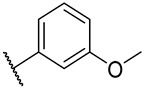	21.58 ± 1.77	6.17 ± 3.68	24.36 ± 4.88	19.32 ± 8.84	27.3 ± 19.42	14.77 ± 12.54
**S3-2**	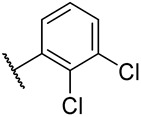	16.62 ± 3.61	4.7 ± 3.7	33.64 ± 15.86	29.05 ± 7.32	22.7 ± 1.92	21.28 ± 4.52
**S3-3**	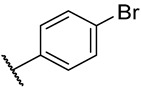	4.58 ± 4.15	7 ± 4.67	27.75 ± 5.76	29.51 ± 3.4	36.81 ± 14.77	24.09 ± 7.61
**S3-4**	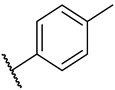	5.4 ± 0.52	7.83 ± 5.72	27.22 ± 6.6	25.31 ± 6.48	34.97 ± 11.31	20.36 ± 1.42
**S3-5**	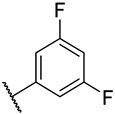	14.27 ± 5.33	5.78 ± 2.24	17.98 ± 10.49	23.14 ± 11.44	23.11 ± 7.17	19.6 ± 4.47
**S3-6**	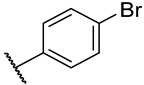	11.08 ± 8.46	5.32 ± 6.71	13.36 ± 9.33	28.07 ± 11.73	25.54 ± 4.12	15 ± 4.51
**S3-7**	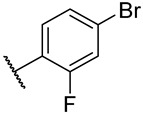	15.3 ± 0.83	2.88 ± 3.52	18.59 ± 5.77	19.72 ± 10.22	19.52 ± 1.73	16.2 ± 2.78
**S3-8**	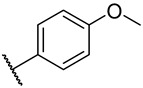	14.14 ± 0.07	4.27 ± 9.34	−4.12 ± 4.65	25.93 ± 4.2	21.89 ± 3.51	14.53 ± 1.4
**S3-9**	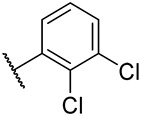	17.39 ± 6.45	5.7 ± 10.54	19.68 ± 4.72	26.61 ± 2.9	26.31 ± 6.05	17.26 ± 5.36
**S3-10**	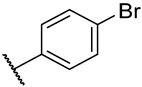	25.9 ± 1.96	7.93 ± 8.29	22.92 ± 4.35	25.54 ± 4.37	38.55 ± 9.07	23.65 ± 4.71
**S3-11**	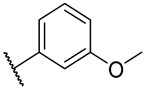	22.32 ± 8.87	7.42 ± 10.68	20.61 ± 5.79	27.29 ± 1.8	35.91 ± 7.34	21.96 ± 2.78
**S3-12**	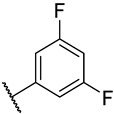	15.27 ± 16.83	8.69 ± 9.12	17.86 ± 0.45	22.5 ± 1.33	35.48 ± 11.83	22.52 ± 3.99
**S3-13**	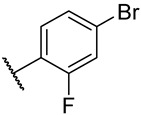	23.73 ± 4.46	8.05 ± 15.13	19.24 ± 1.48	21.15 ± 3.64	38.72 ± 13.86	19.09 ± 3.8
**S3-14**	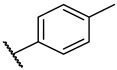	27 ± 6.19	6.98 ± 0.42	23.65 ± 5.88	30.27 ± 4.04	27.75 ± 3.38	17.07 ± 5.26
**Tanshinone** **II** **A**	-	−0.17 ± 0.63	7.41 ± 3.36	30.61 ± 5.17	29.3 ± 5.01	56.71 ± 8.48	26.53 ± 6.14
**Tanshinone** **I**	-	21.05 ± 13.77	5.52 ± 1.43	35.46 ± 4.02	28.04 ± 9.13	73.53 ± 4.24	22.02 ± 7.23
**Int-4**	-	17.19 ± 3.58	5.78 ± 0.43	42.92 ± 10.81	26.33 ± 6.85	73.01 ± 6.2	20.55 ± 2.62
**β-lapachone**	-	82.18 ± 1.82	10.19 ± 1.26	97.92 ± 0.08	36.96 ± 3.92	70.81 ± 15.09	78.16 ± 2.04

*^a^* The data represent the average of two independent experiments; *^b^*^,*c*^ the data represent the average of three independent experiments.

**Table 4:** IC_50_ values of selected compounds in vitro.

Compounds	IC_50_/μM *^a^*	
	A549	H838
**S2-1**	2.4 ± 0.14	1.59 ± 0.29 **
**S2-3**	3.82 ± 0.07	1.14 ± 0.18 **
**S2-4**	2.48 ± 0.20	0.58 ± 0.07 **
**S2-8**	2.39 ± 0.32	0.42 ± 0.04 **
**S2-13**	3.66 ± 0.12	1.28 ± 0.13 **
**S2-14**	3.78 ± 0.75	1.80 ± 0.24 **
**β-lapachone**	2.74 ± 0.14	2.62 ± 0.05

Note: ** represents *p* < 0.01 vs. the inhibition of the positive control to the cancer cell lines. *^a^* The data represented the average of three independent experiments.

## Data Availability

Data is contained within the article or Supplementary Materials.

## Supplementary Materials

The following supporting information can be downloaded at: https://www.mdpi.com/article/10.3390/molecules31030446/s1. ^1^H/^13^C/^19^F NMR, HPLC, and HRMS spectra of the synthesized compounds in this study.
