# Supplementary material for: Development of a Series of Tanshinone Derivatives Through Scaffold Hopping for Treating Non-Small-Cell Lung Cancer (NSCLC)
Source: Molecules. 2026 Jan 27;31(3):446. doi: 10.3390/molecules31030446 (PMC12900021; doi:10.3390/molecules31030446)
Supplement: Supplementary file 1 [file molecules-31-00446-s001.zip › molecules-4097889-supplementary.pdf]

Supplementary file

Development of a Series of Tanshinone Derivatives Through Scaffold Hopping for Treating Non-Small-Cell Lung Cancer (NSCLC)

Lan-Xin Zhou [1,2,†], Zheng-Yu Shu [1,†], Heng Li [3], Hui Zhong [2,4], Dou-Nan Xu [2], Lei Tang [1,5], Chu-Jiao Hu [1,5,*], Cheng Luo [1,2,3,4,5,*], Huan Xiong [2,*]

1 State Key Laboratory of Discovery and Utilization of Functional Components in Traditional Chinese Medicine, Guizhou Provincial Key Laboratory of Innovation and Manufacturing for Pharmaceuticals, Guizhou Medical University, Guiyang 550004, China; zhoulanxin607@zidd.ac.cn (L.-X.Z.); 2024120030698@stu.gmc.edu.cn (Z.-Y.S.); tlei1974@163.com (L.T.)

2 Zhongshan Institute for Drug Discovery, Shanghai Institute of Materia Medica, Chinese Academy of Sciences, Zhongshan 528400, China; zhonghui1900@zidd.ac.cn (H.Z.); xudounan@zidd.ac.cn (D.-N.X.)

3 School of Chinese Materia Medica, Nanjing University of Chinese Medicine, Nanjing 210023, China liheng2022@foxmail.com (H.L.);

4 Guangzhou University of Chinese Medicine, Guangdong 510006, China

5 Key Laboratory for Cancer Prevention and Treatment of Guizhou Province, Guiyang 550004, China

* Correspondence: huchujiao2021@gmc.edu.cn (C.-J.H.); luocheng@zidd.ac.cn (C.L.); xionghuan@zidd.ac.cn (H.X.)

† These authors contributed equally to this work

This file contains the $^{1}H/^{13}C/^{19}F$ NMR, HPLC and HRMS spectra of the synthesized compounds

1. ^{1}H/^{13}C/^{19}F NMR, HPLC and HRMS spectra of the synthesized compounds

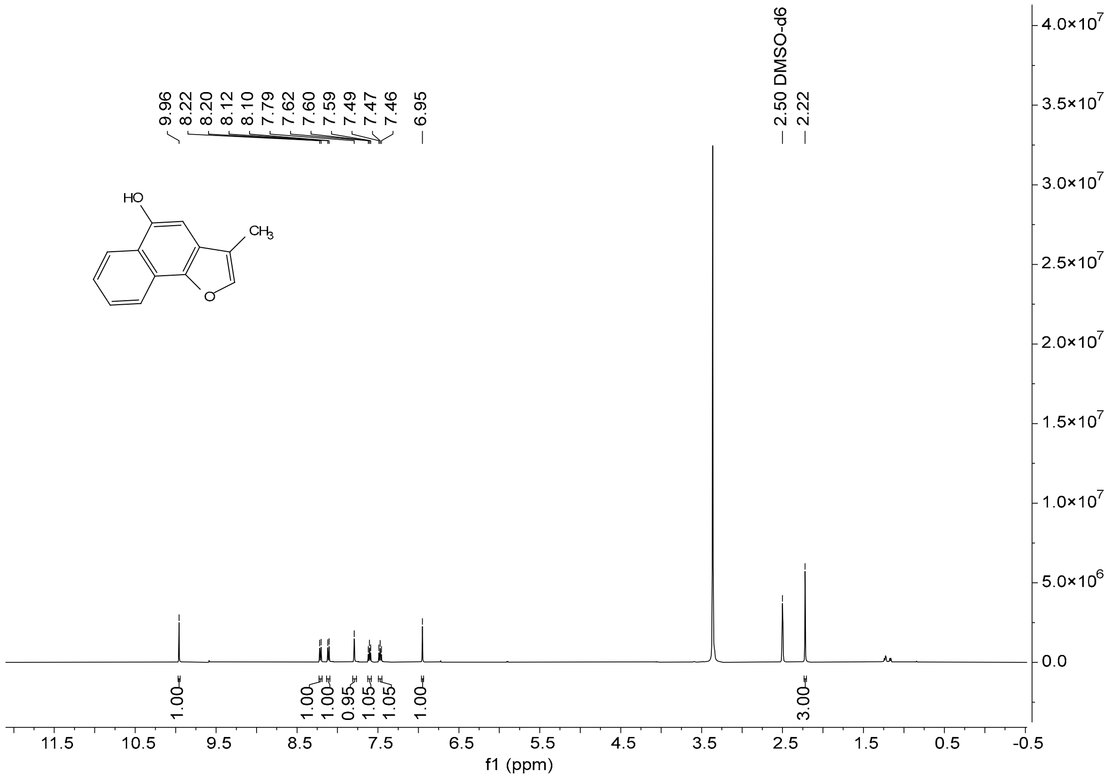

Figure S1. ^{1}H NMR spectrum (500 MHz, DMSO-d_6) of compound **Int-3**.

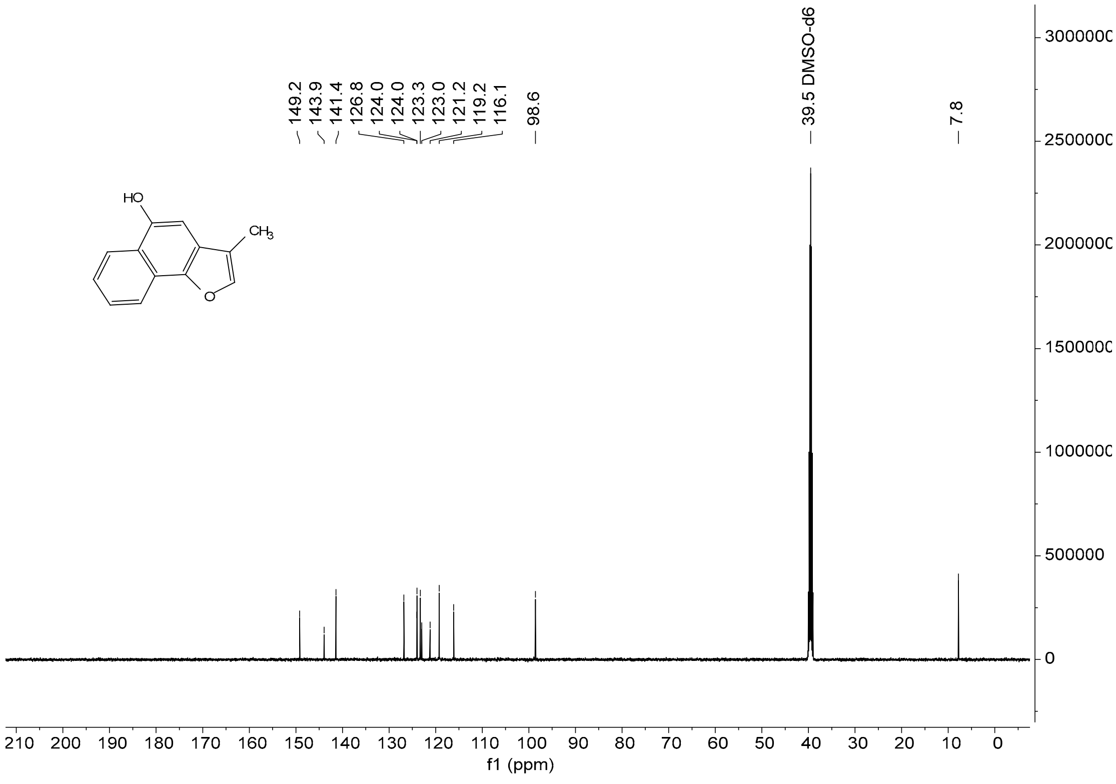

Figure S2. ^{13}C NMR spectrum (500 MHz, DMSO-d_6) of compound **Int-3**.

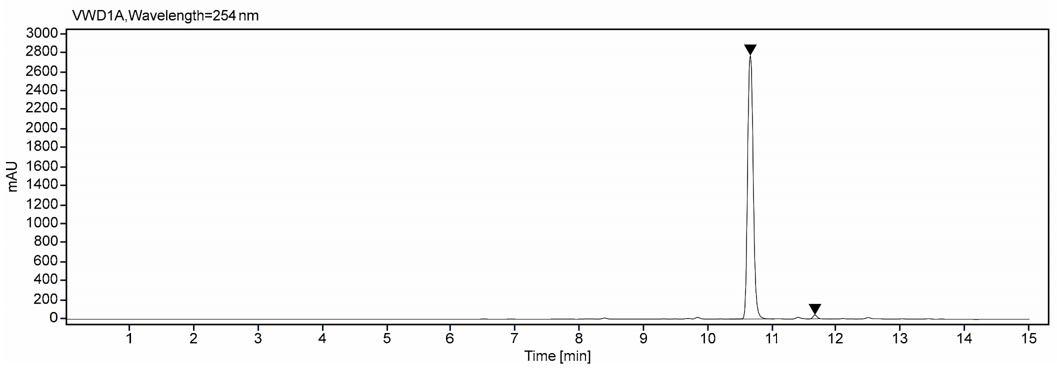

Signal: VWD1A,Wavelength=254 nm

RT [min]	Width [min]	Area	Height	Area%
10.653	0.69	16902.58	2772.77	98.93
11.660	0.23	182.63	40.56	1.07
	**Sum**	**17085.21**		

Figure S3. HPLC spectrum of compound **Int-3**.

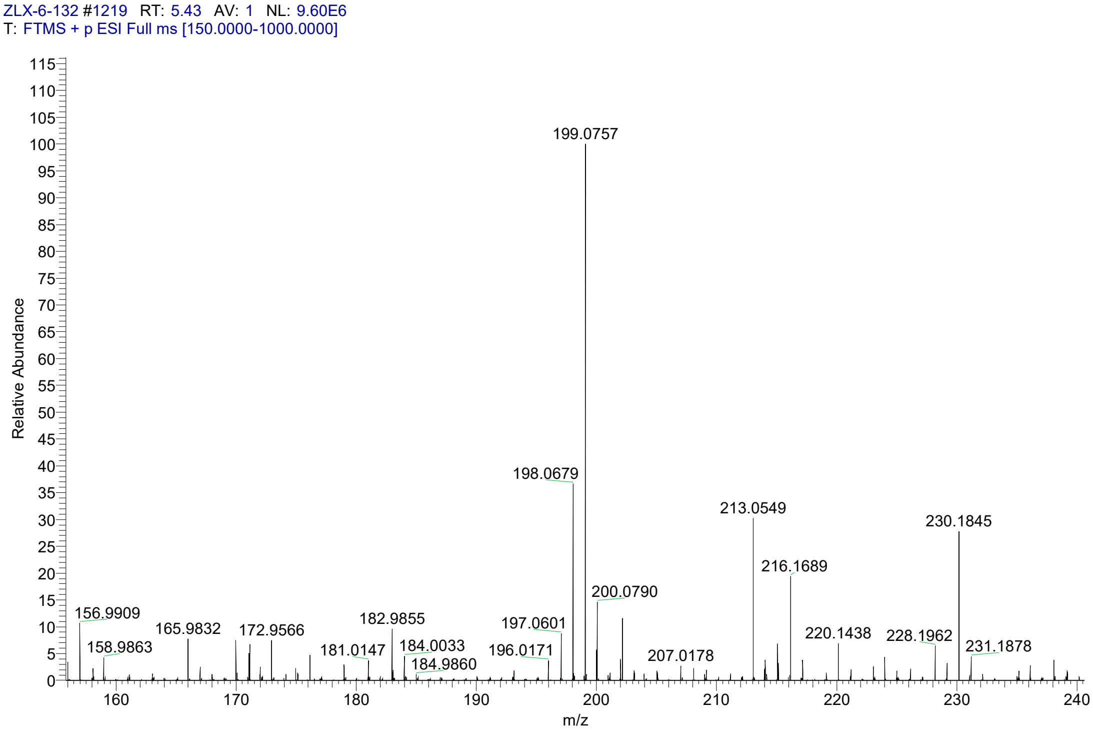

Figure S4. HRMS of compound **Int-3**.

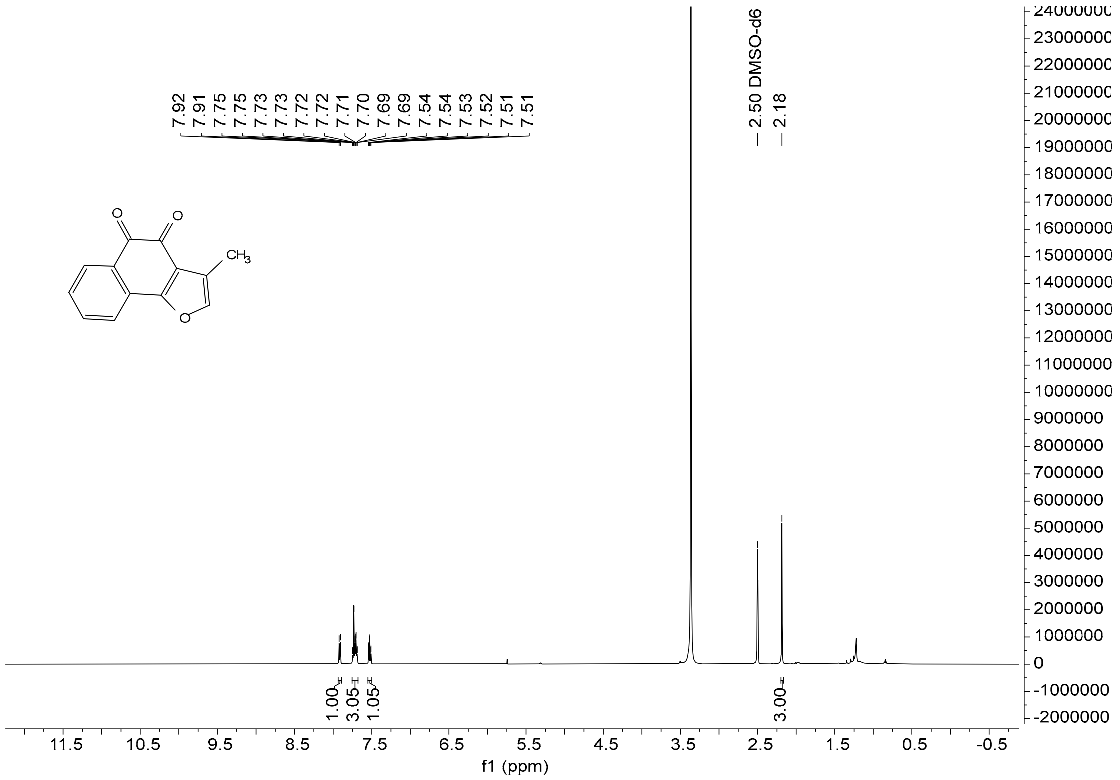

Figure S5. ^{1}H NMR spectrum (500 MHz, DMSO-d_6) of compound **Int-4**.

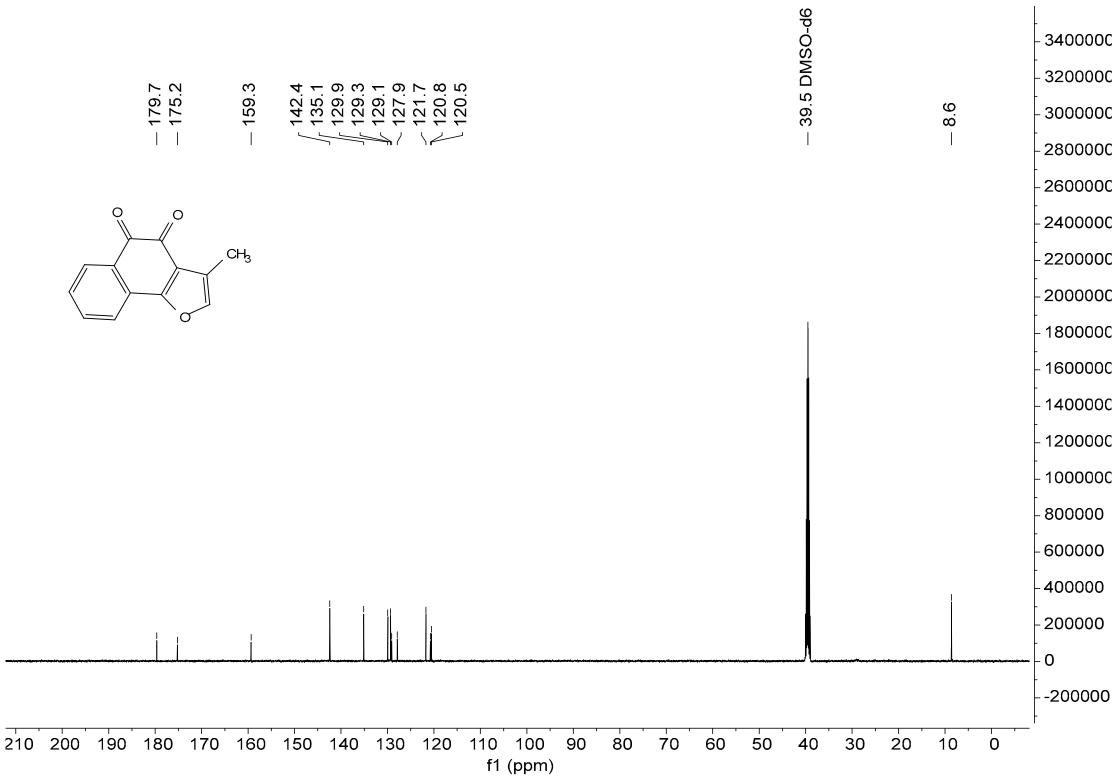

Figure S6. ^{13}C NMR spectrum (500 MHz, DMSO-d_6) of compound **Int-4**.

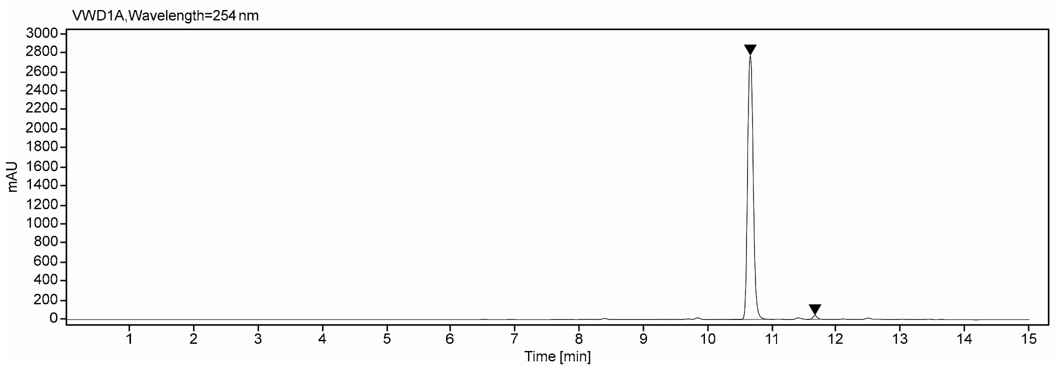

Signal: VWD1A,Wavelength=254 nm

RT [min]	Width [min]	Area	Height	Area%
10.653	0.69	16902.58	2772.77	98.93
11.660	0.23	182.63	40.56	1.07
	**Sum**	**17085.21**		

Figure S7. HPLC spectrum of compound **Int-4**.

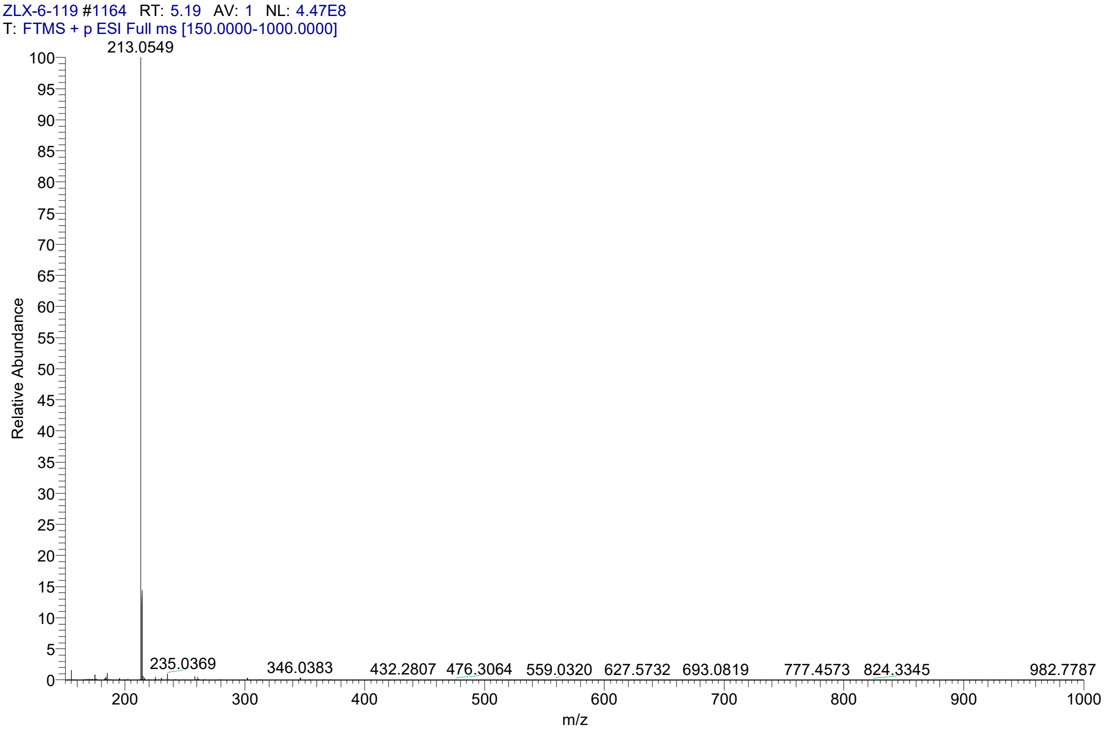

Figure S8. HRMS of compound **Int-4**.

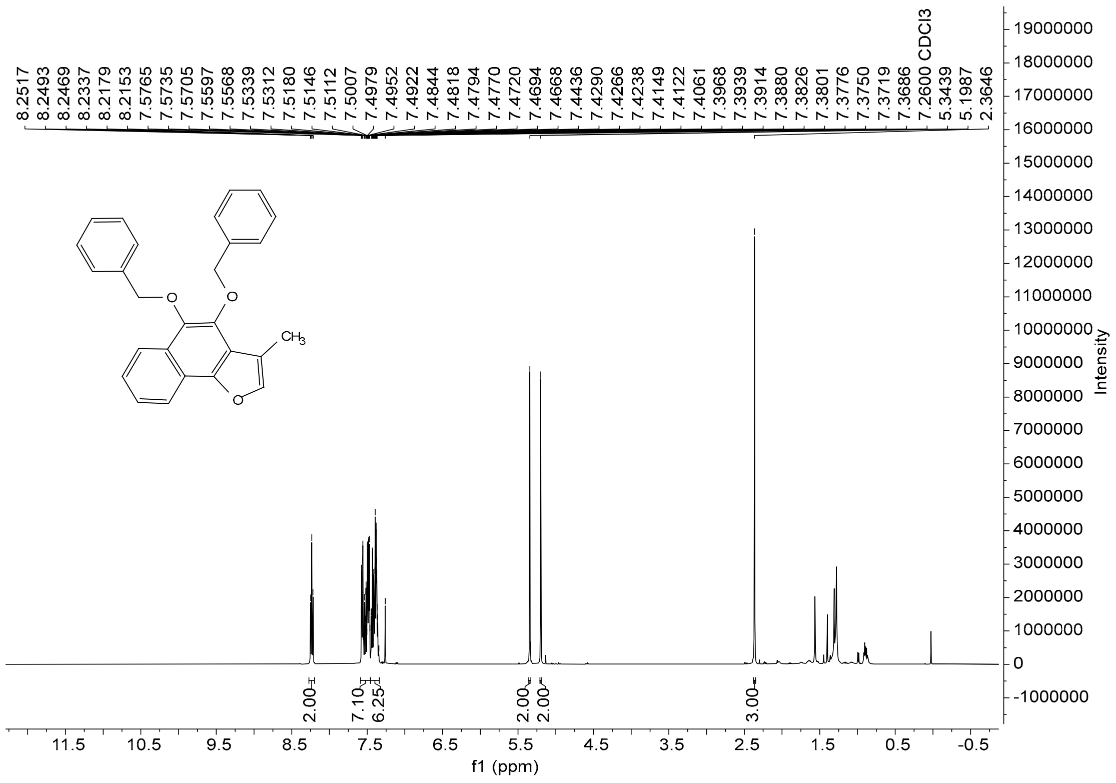

Figure S9. ^{1}H NMR spectrum (500 MHz, Chloroform-*d*) of compound **Int-5**.

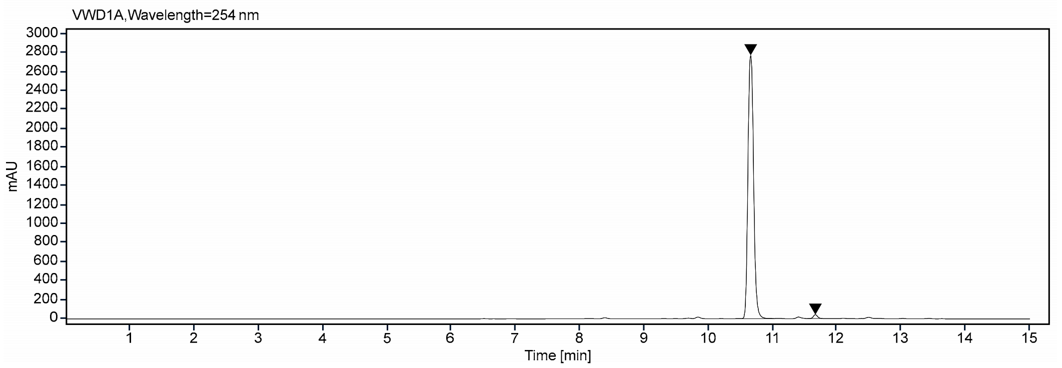

Signal:	VWD1A,Wavelength=254 nm			
**RT [min]**	**Width [min]**	**Area**	**Height**	**Area%**
10.653	0.69	16902.58	2772.77	98.93
11.660	0.23	182.63	40.56	1.07
	**Sum**	**17085.21**		

Figure S10. HPLC spectrum of compound **Int-5**.

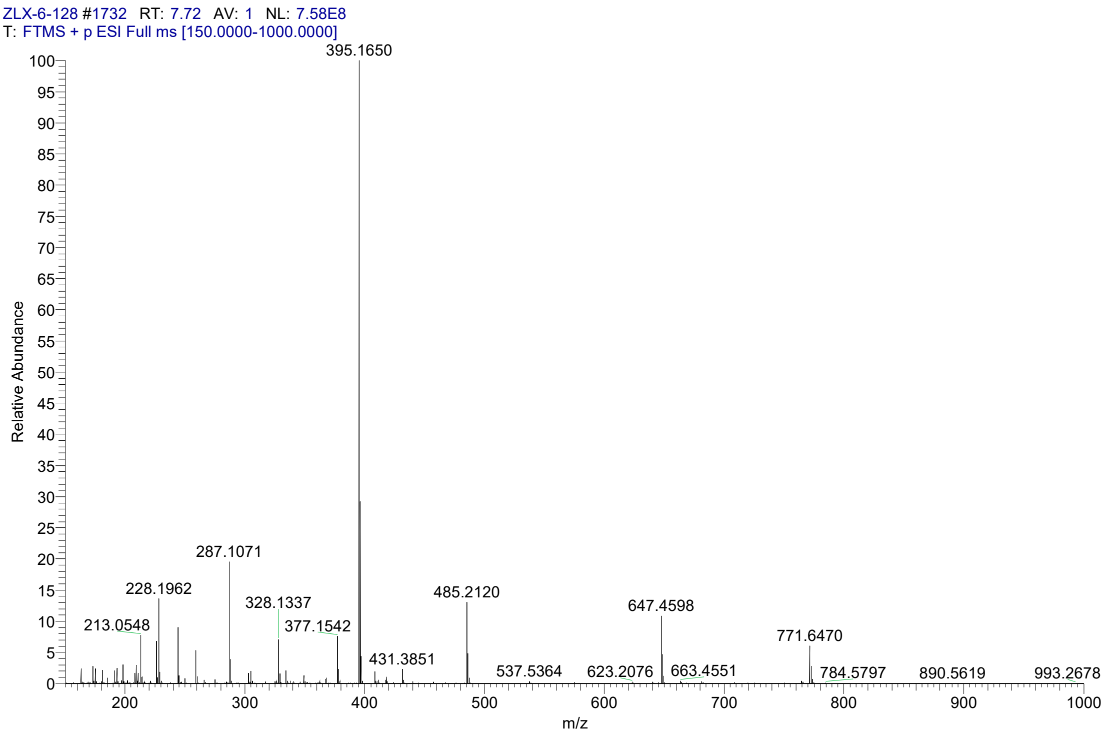

Figure S11. HRMS of compound **Int-5**.

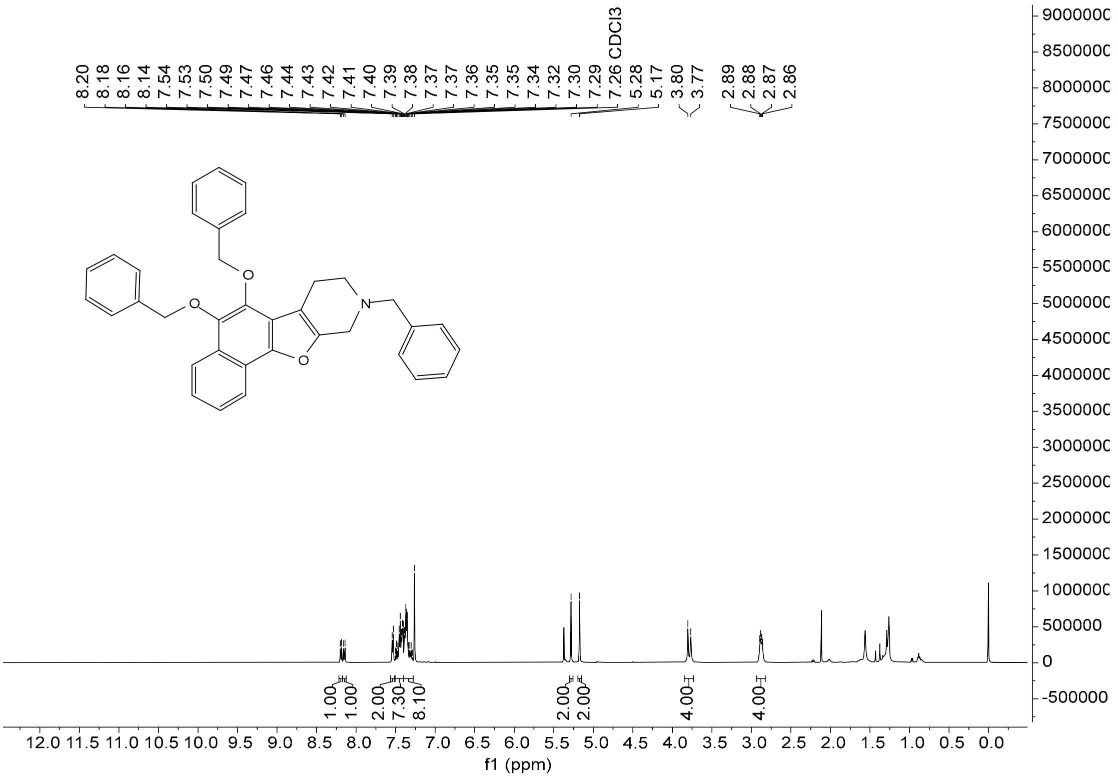

Figure S12. ^{1}H NMR spectrum (500 MHz, Chloroform-*d*) of compound **Int-6**.

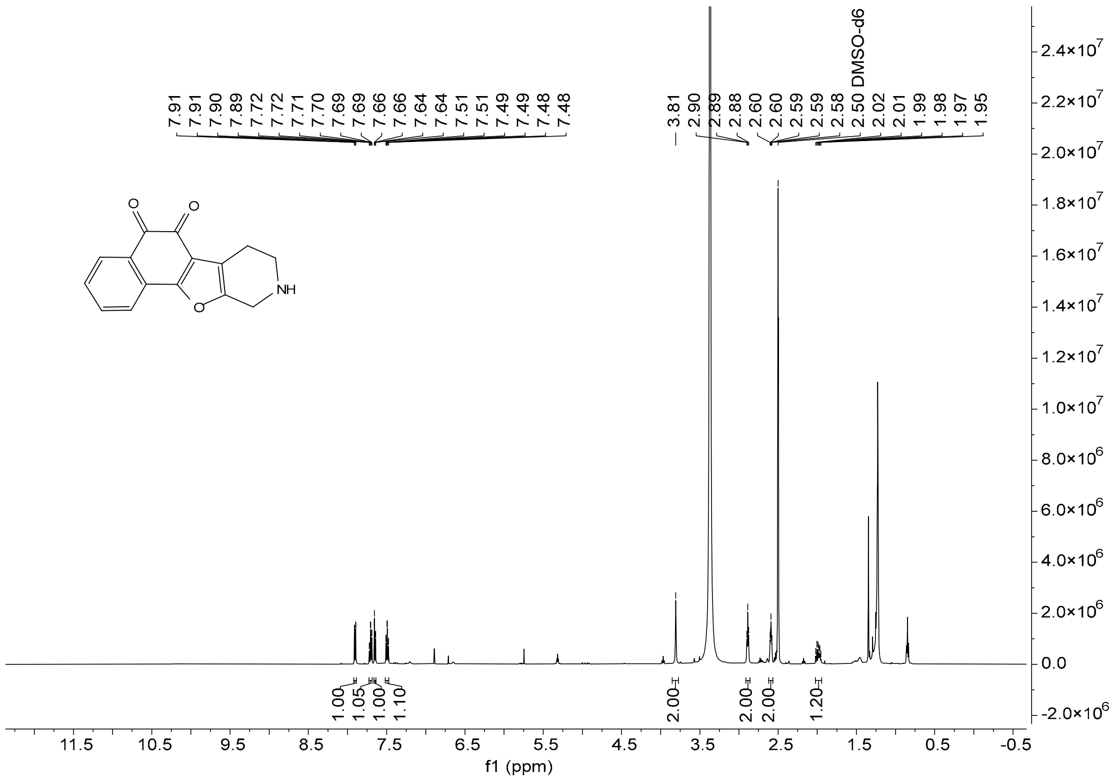

Figure S13. ^{1}H NMR spectrum (500 MHz, DMSO-*d*$_6$)) of compound **S1-1**.

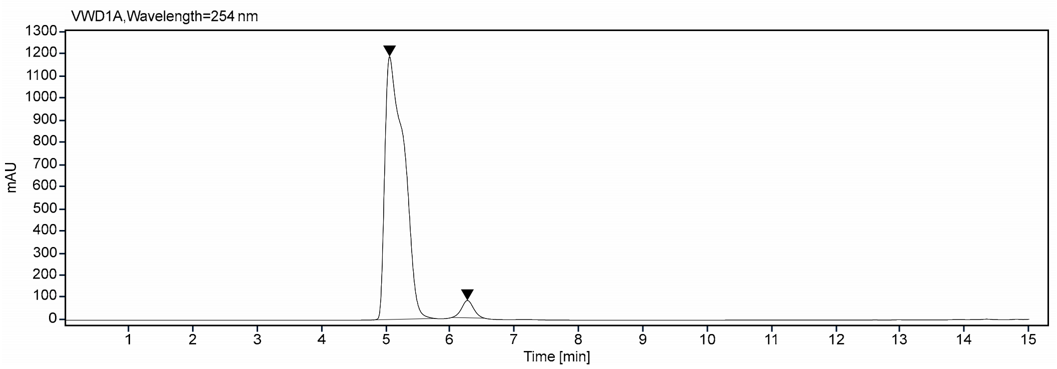

Signal: VWD1A,Wavelength=254 nm

RT [min]	Width [min]	Area	Height	Area%
5.046	0.96	24510.53	1186.24	96.05
6.258	0.50	1008.71	77.93	3.95
	**Sum**	**25519.24**		

Figure S14. HPLC spectrum of compound **S1-1**.

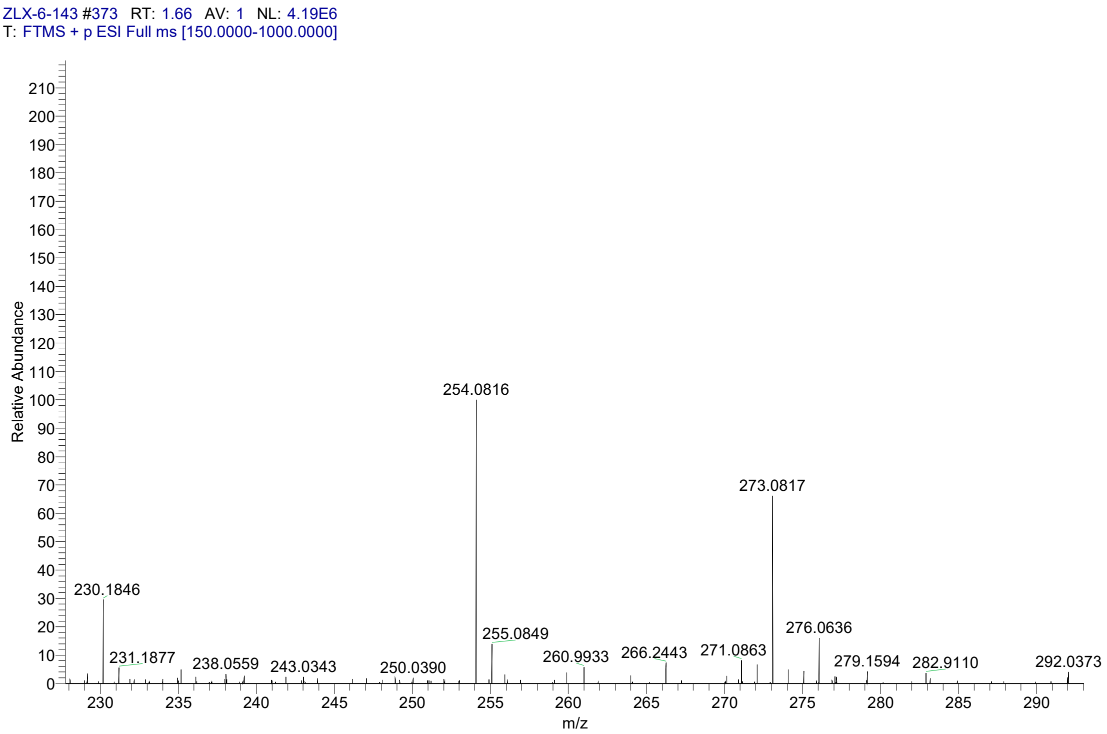

Figure S15. HRMS of compound **S1-1**.

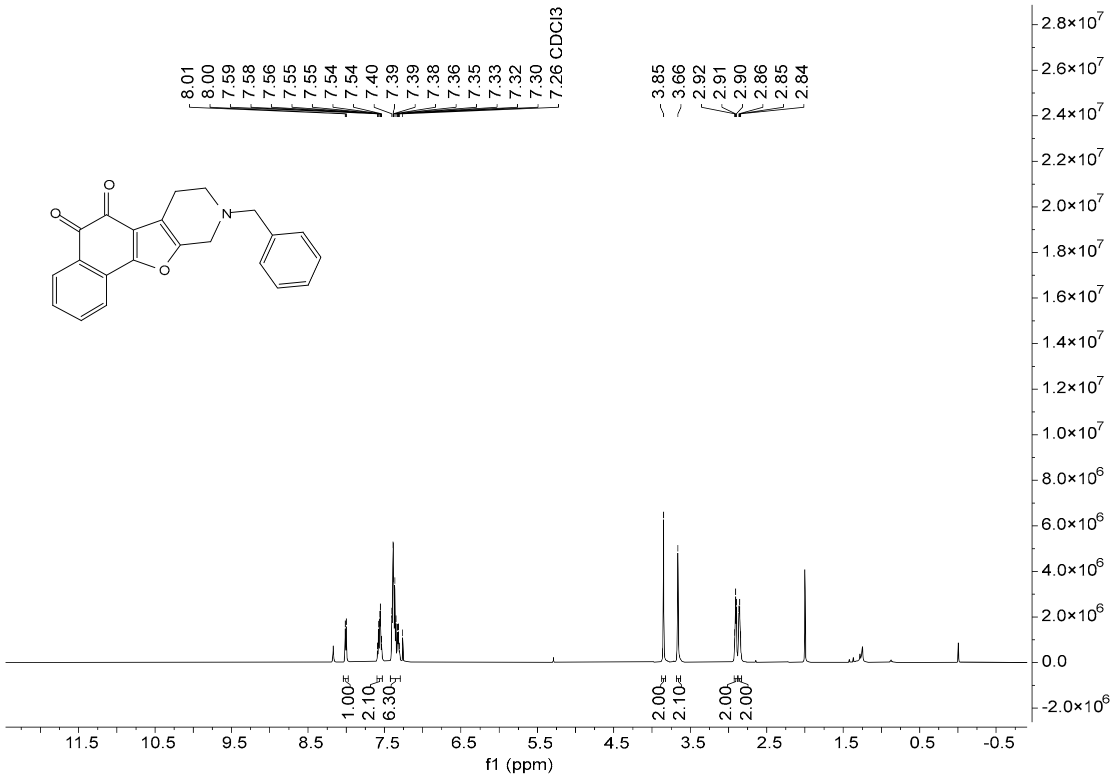

Figure S16. ^{1}H NMR spectrum (500 MHz, Chloroform-*d*) of compound **S1-2**.

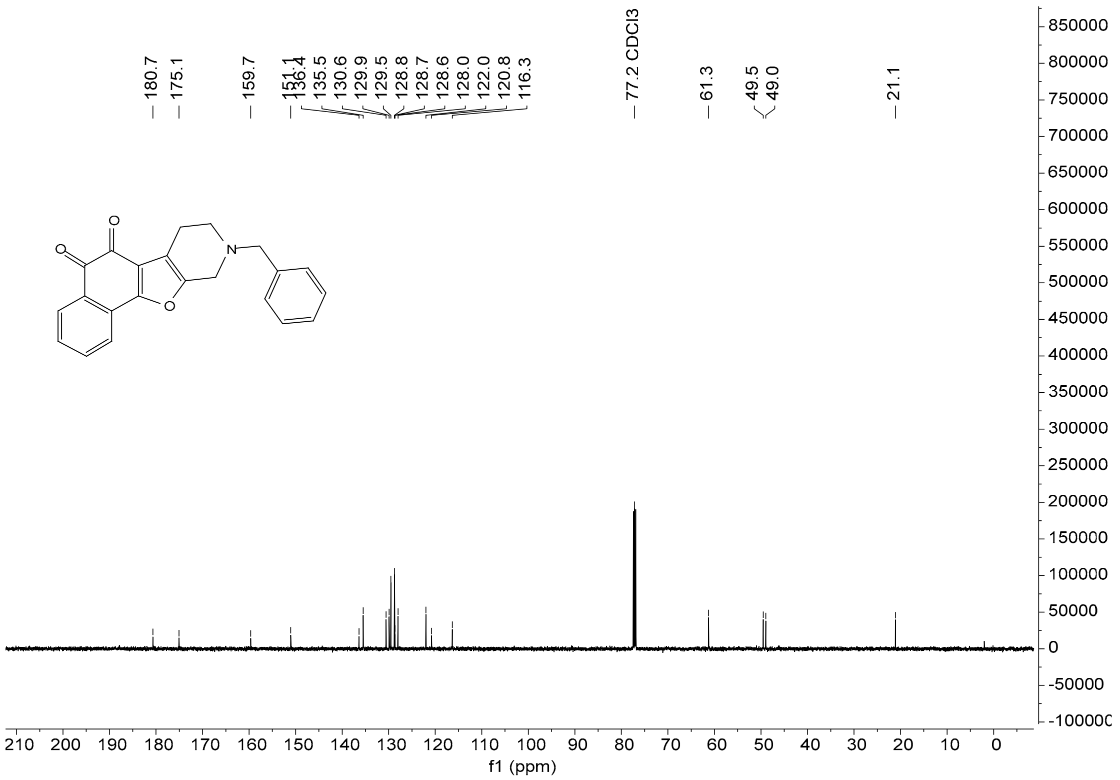

Figure S17. ^{13}C NMR spectrum (500 MHz, Chloroform-*d*) of compound **S1-2**.

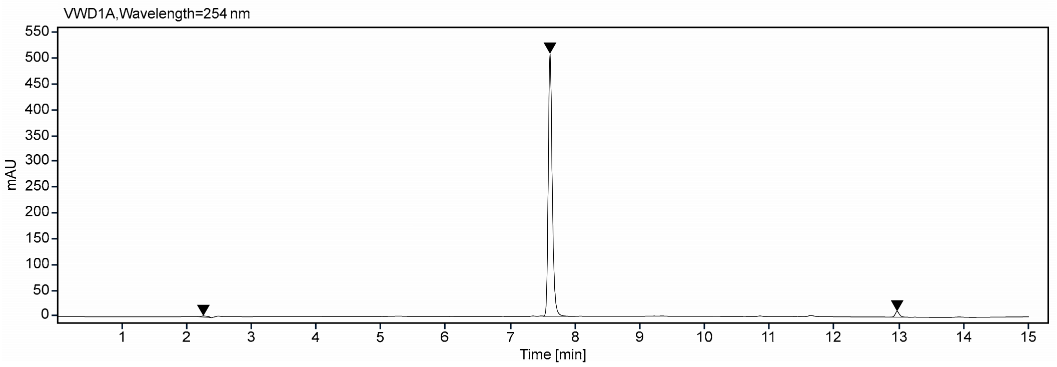

Signal:	VWD1A,Wavelength=254 nm			
**RT [min]**	**Width [min]**	**Area**	**Height**	**Area%**
2.249	0.22	17.81	2.30	0.79
7.604	0.49	2197.48	509.86	96.95
12.964	0.32	51.31	11.18	2.26
	**Sum**	**2266.60**		

Figure S18. HPLC spectrum of compound **S1-2**.

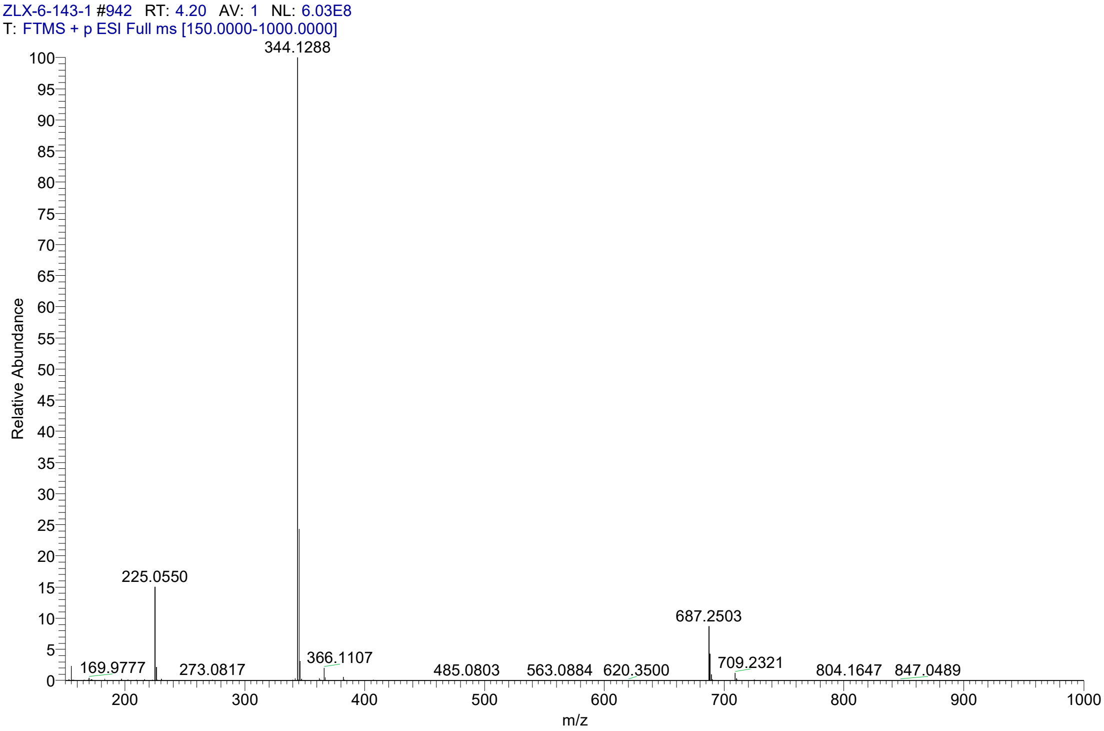

Figure S19. HRMS of compound **S1-2**.

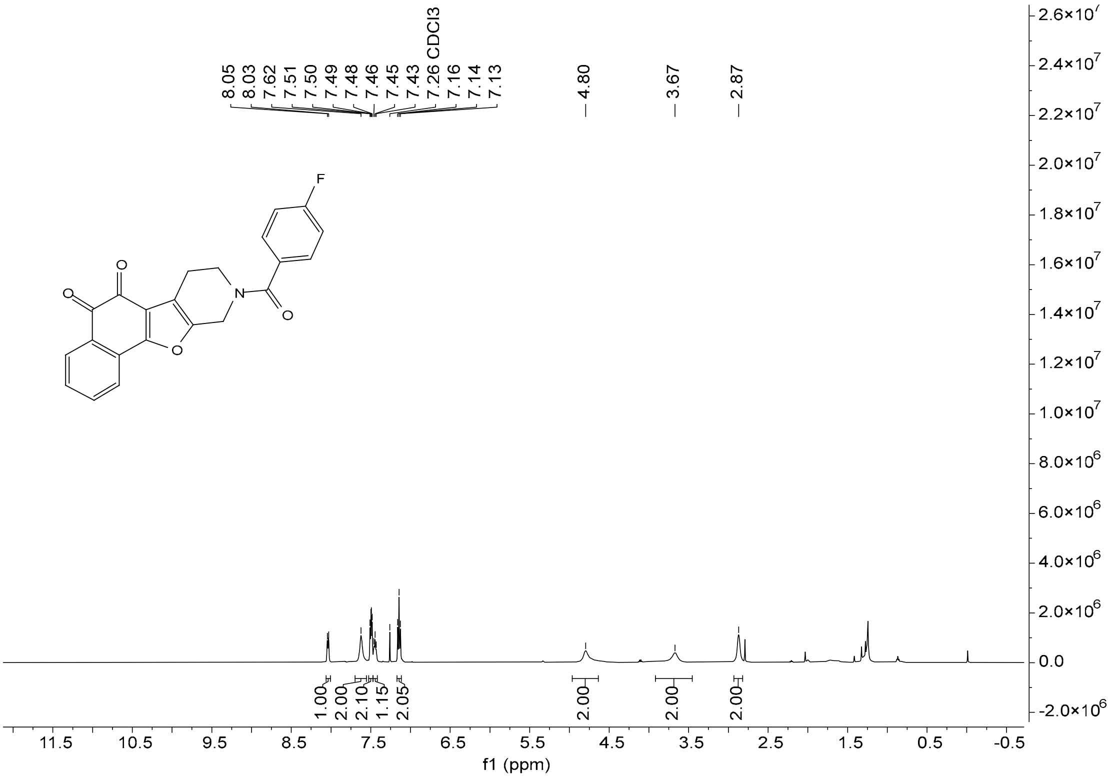

Figure S20. ^{1}H NMR spectrum (500 MHz, Chloroform-*d*) of compound **S1-3**.

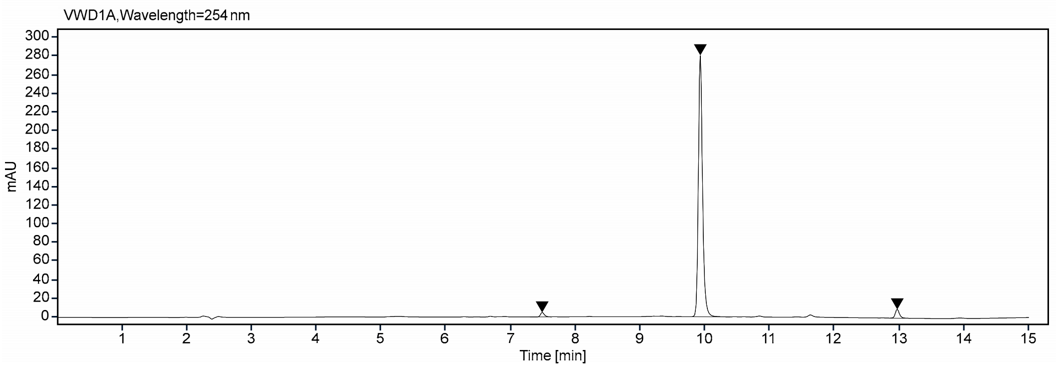

Signal: VWD1A,Wavelength=254 nm

RT [min]	Width [min]	Area	Height	Area%
7.484	0.33	20.30	4.70	1.53
9.925	0.61	1263.25	280.66	95.29
12.966	0.32	42.12	9.38	3.18
	**Sum**	**1325.66**		

Figure S21. HPLC spectrum of compound **S1-3**.

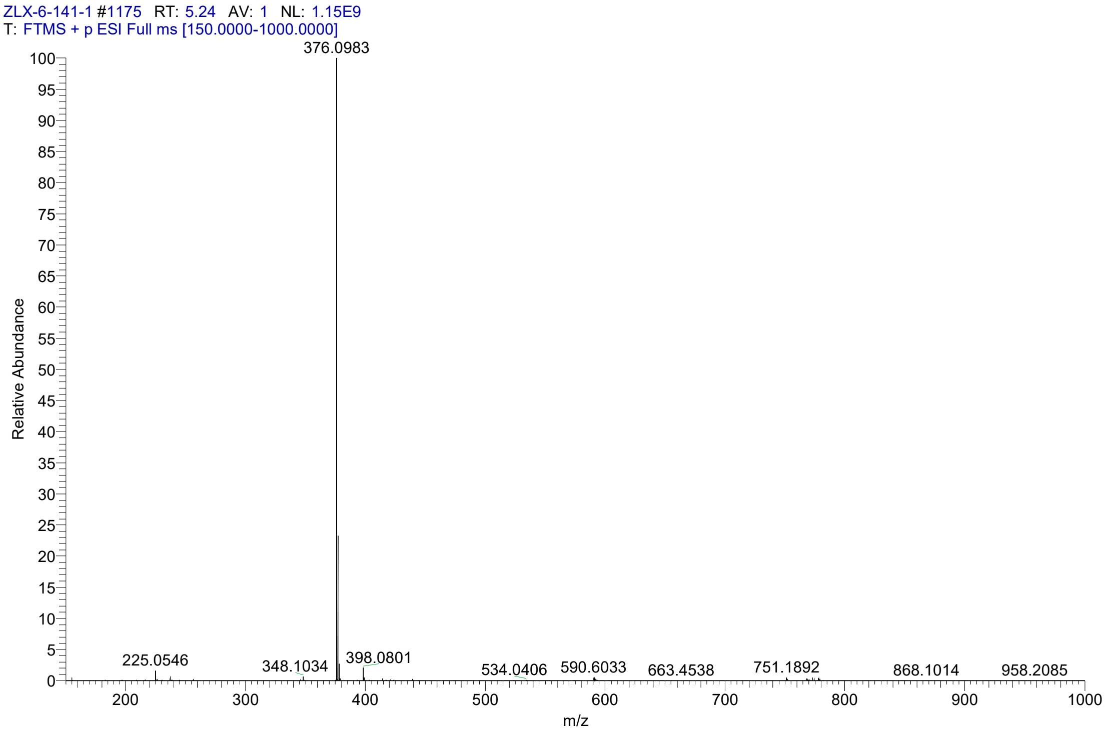

Figure S22. HRMS of compound **S1-3**.

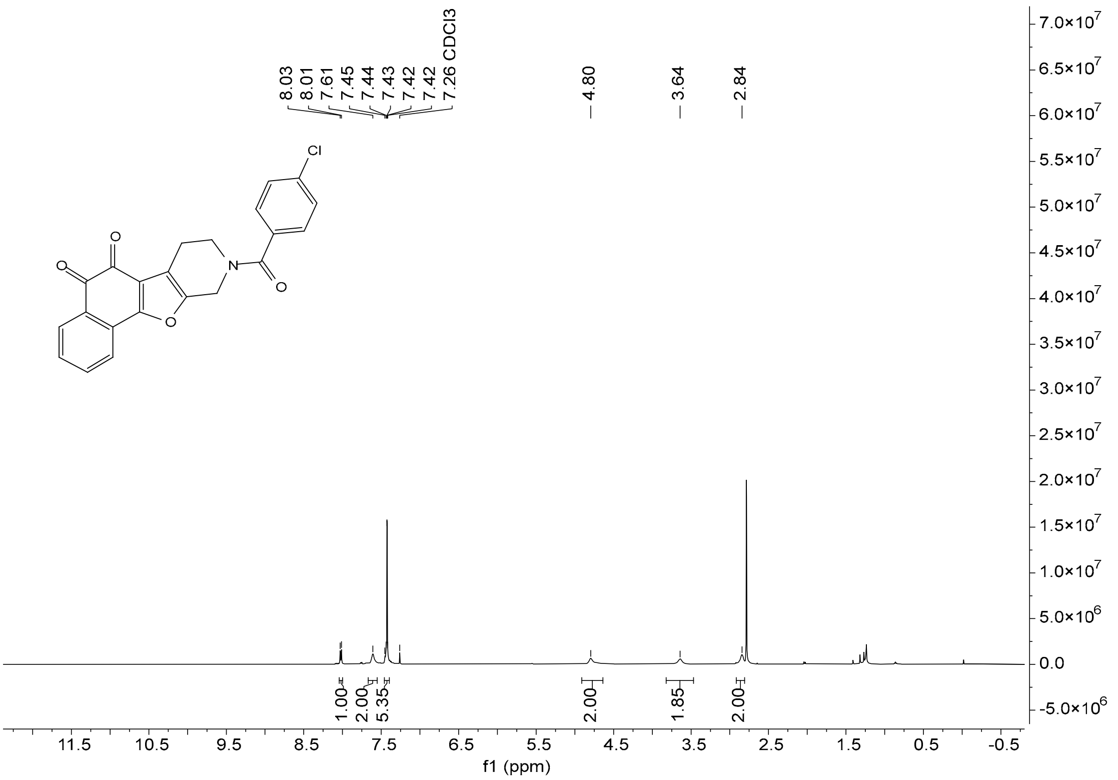

Figure S23. ^{1}H NMR spectrum (500 MHz, Chloroform-*d*) of compound **S1-4**.

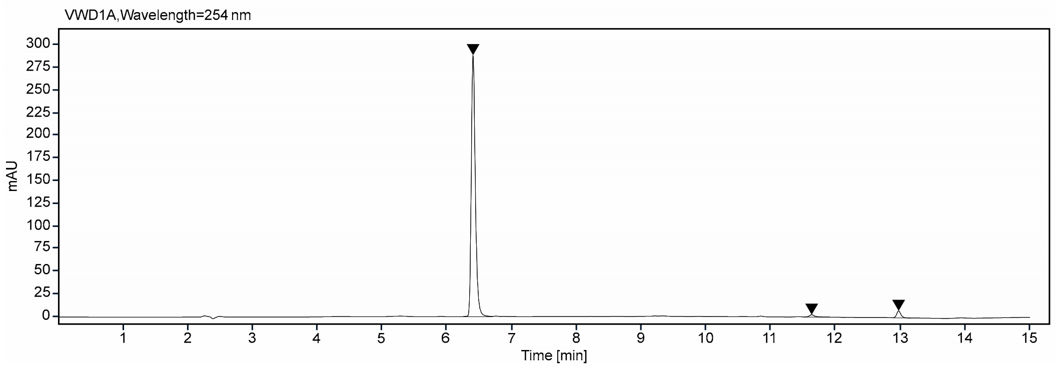

Signal: VWD1A,Wavelength=254 nm

RT [min]	Width [min]	Area	Height	Area%
6.398	0.51	1262.58	288.24	96.00
11.628	0.64	17.87	2.57	1.36
12.970	0.30	34.80	7.67	2.65
	**Sum**	**1315.25**		

Figure S24. HPLC spectrum of compound **S1-4**.

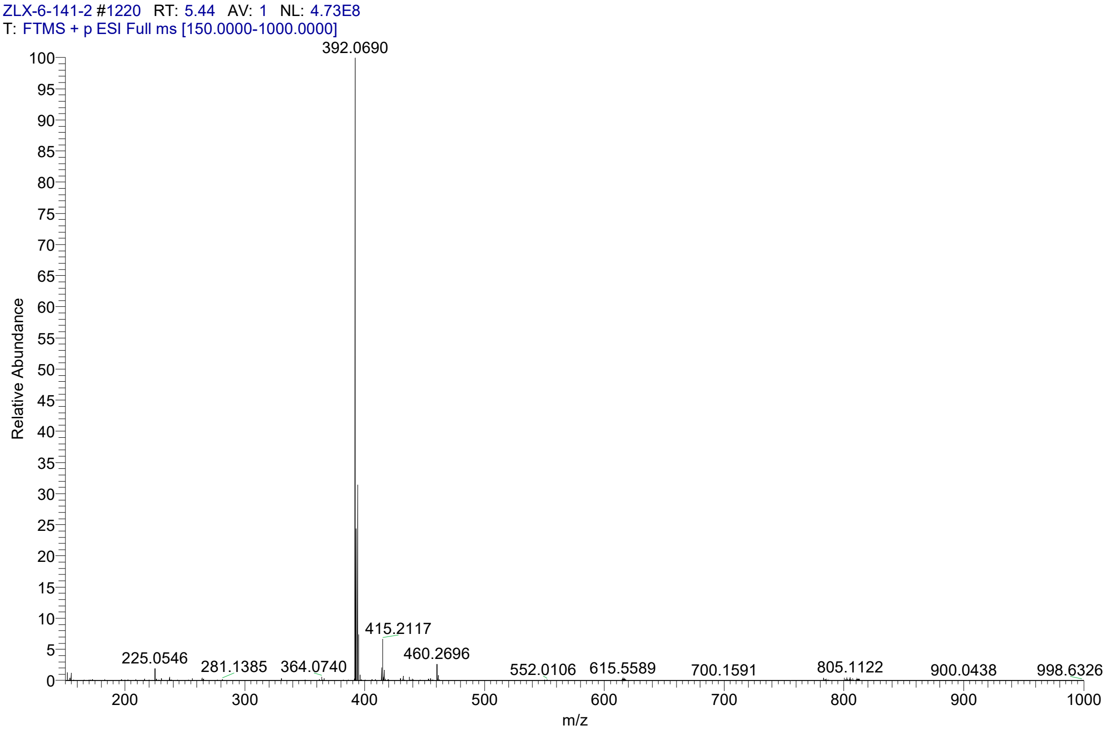

Figure S25. HRMS of compound **S1-4**.

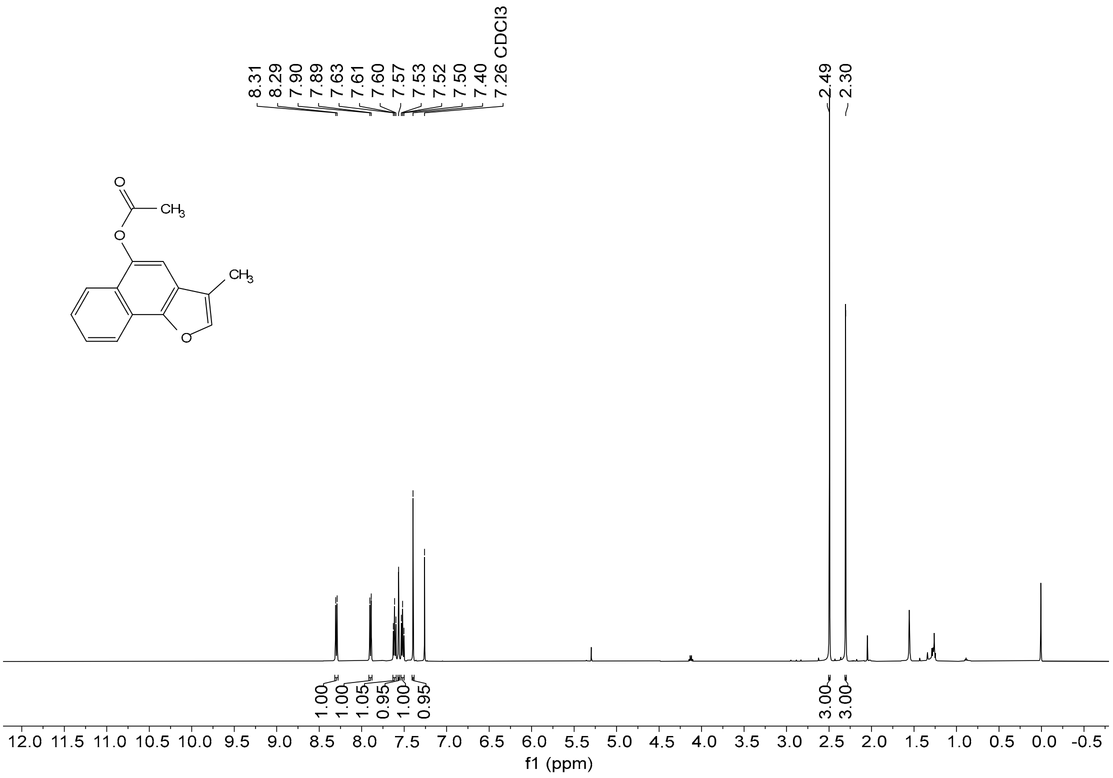

Figure S26. ^{1}H NMR spectrum (500 MHz, Chloroform-*d*) of compound **Int-7**.

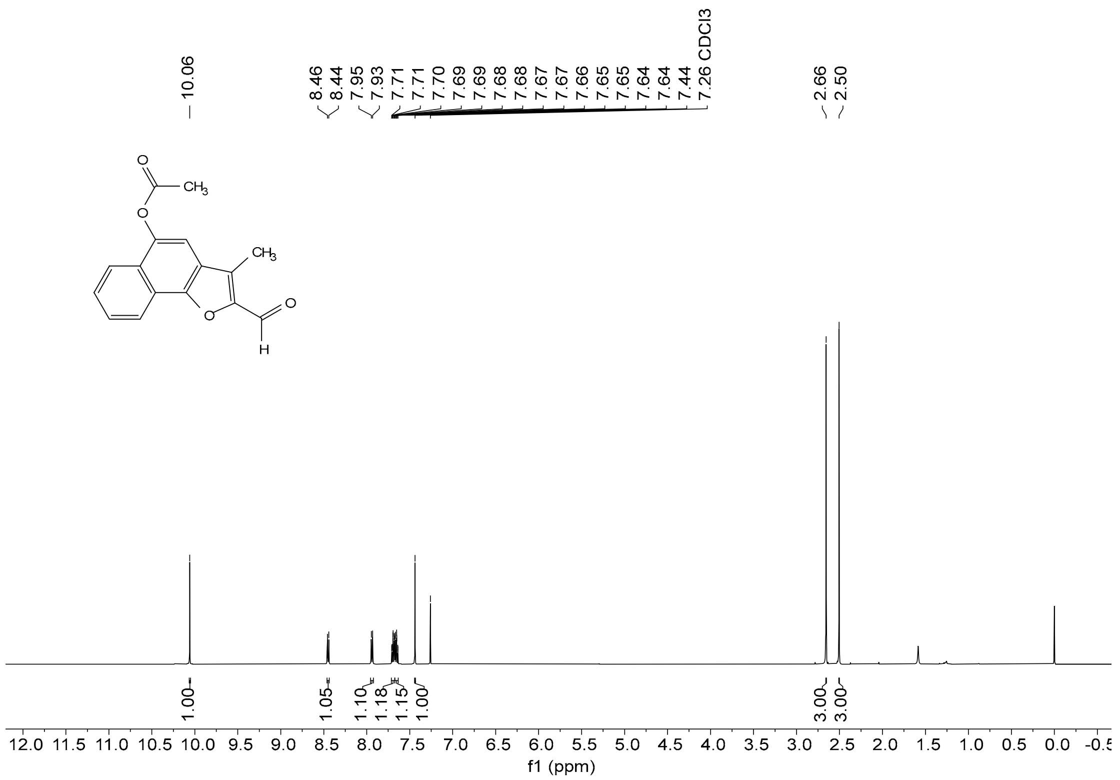

Figure S27. ^{1}H NMR spectrum (500 MHz, Chloroform-*d*) of compound **Int-8**.

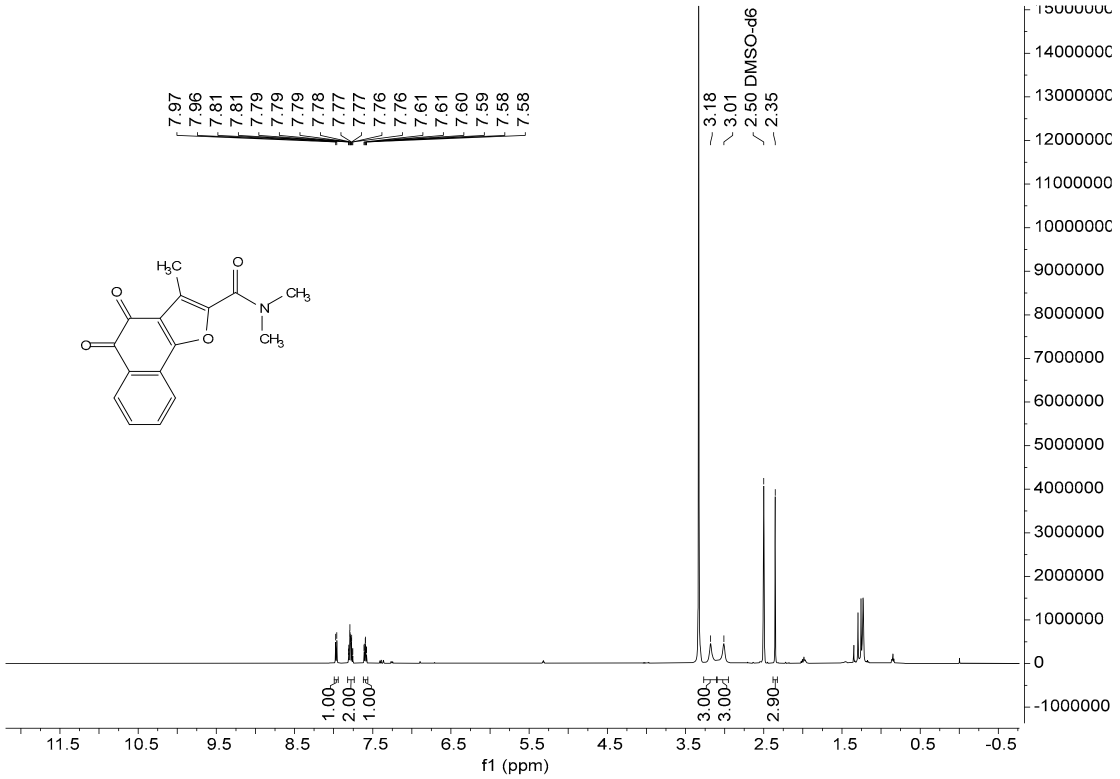

Figure S28. ^{1}H NMR spectrum (500 MHz, DMSO-*d*$_6$) of compound **S2-1**.

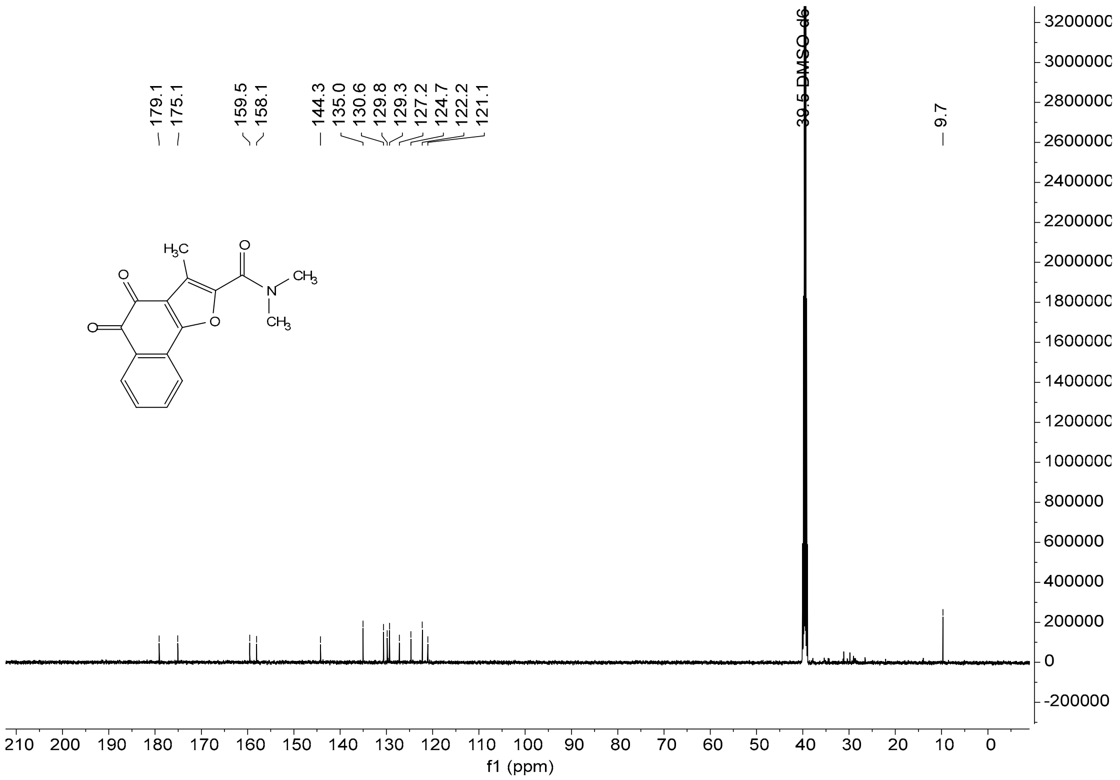

Figure S29. ^{13}C NMR spectrum (500 MHz, DMSO-d_6) of compound **S2-1**.

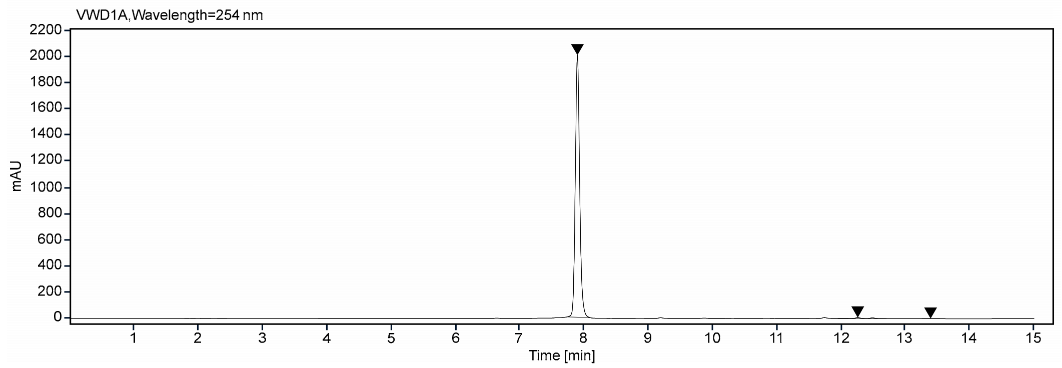

Signal: VWD1A,Wavelength=254 nm

RT [min]	Width [min]	Area	Height	Area%
7.891	0.36	9393.00	2006.82	99.42
12.253	0.76	49.68	7.45	0.53
13.388	0.28	4.78	0.67	0.05
	**Sum**	**9447.47**		

Figure S30. HPLC spectrum of compound **S2-1**.

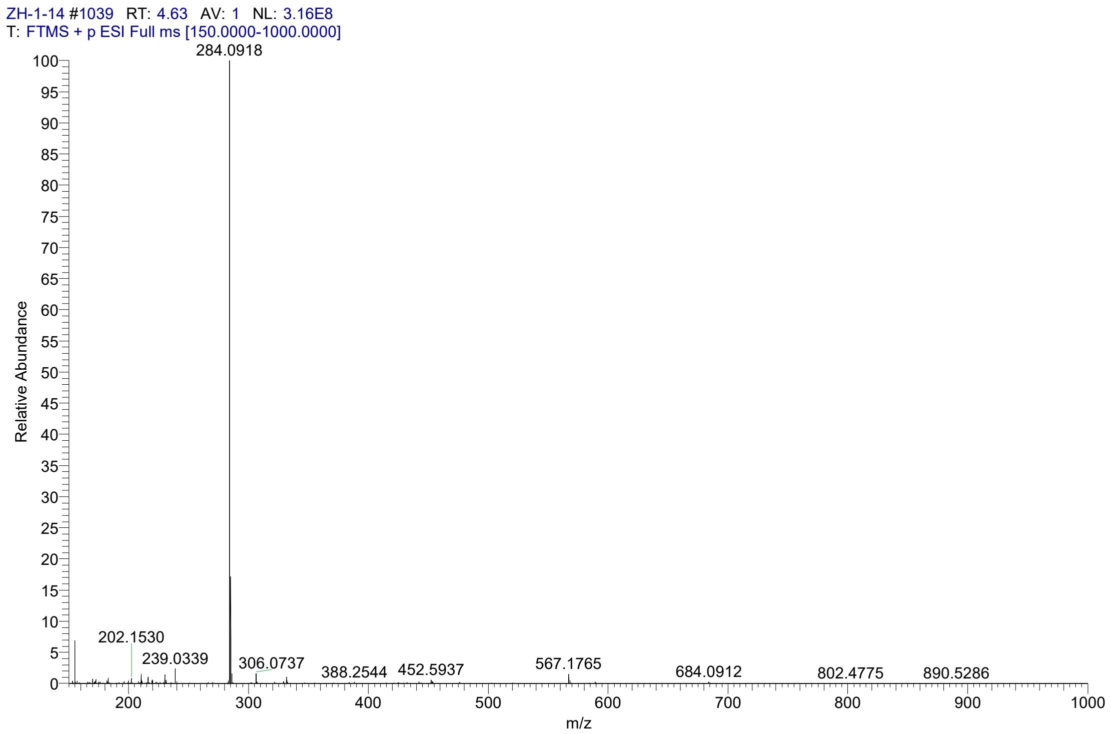

Figure S31. HRMS of compound **S2-1**.

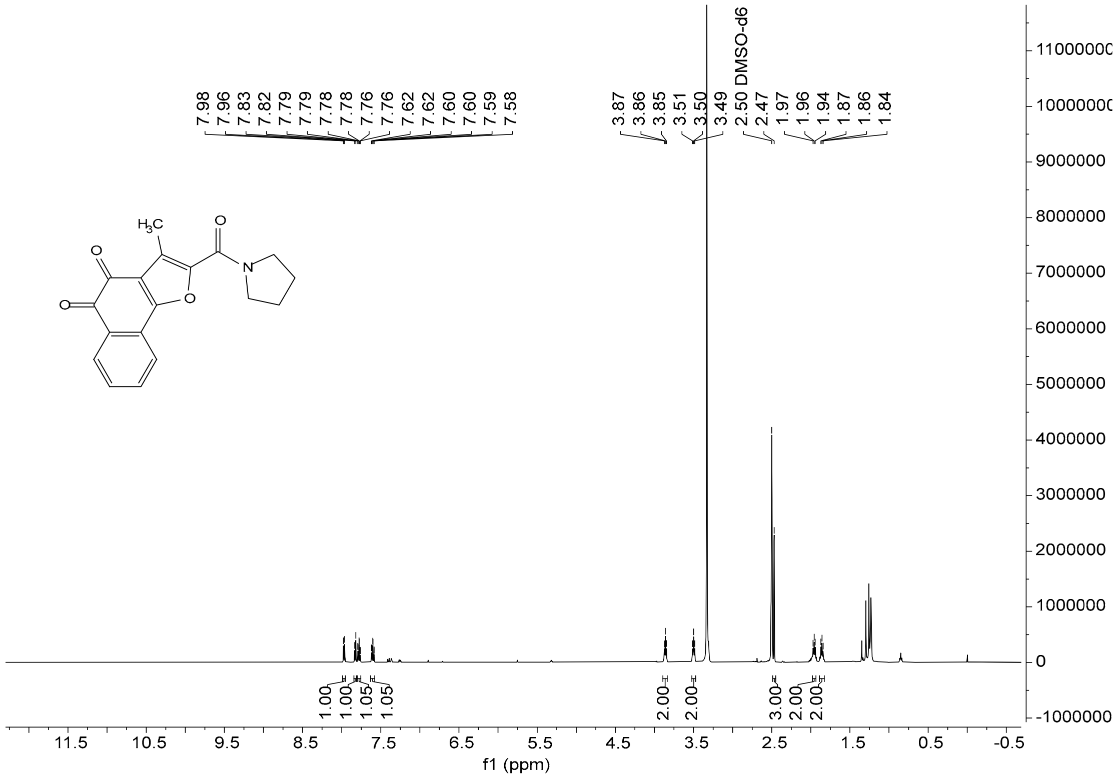

Figure S32. ^{1}H NMR spectrum (500 MHz, DMSO-d_6) of compound **S2-2**.

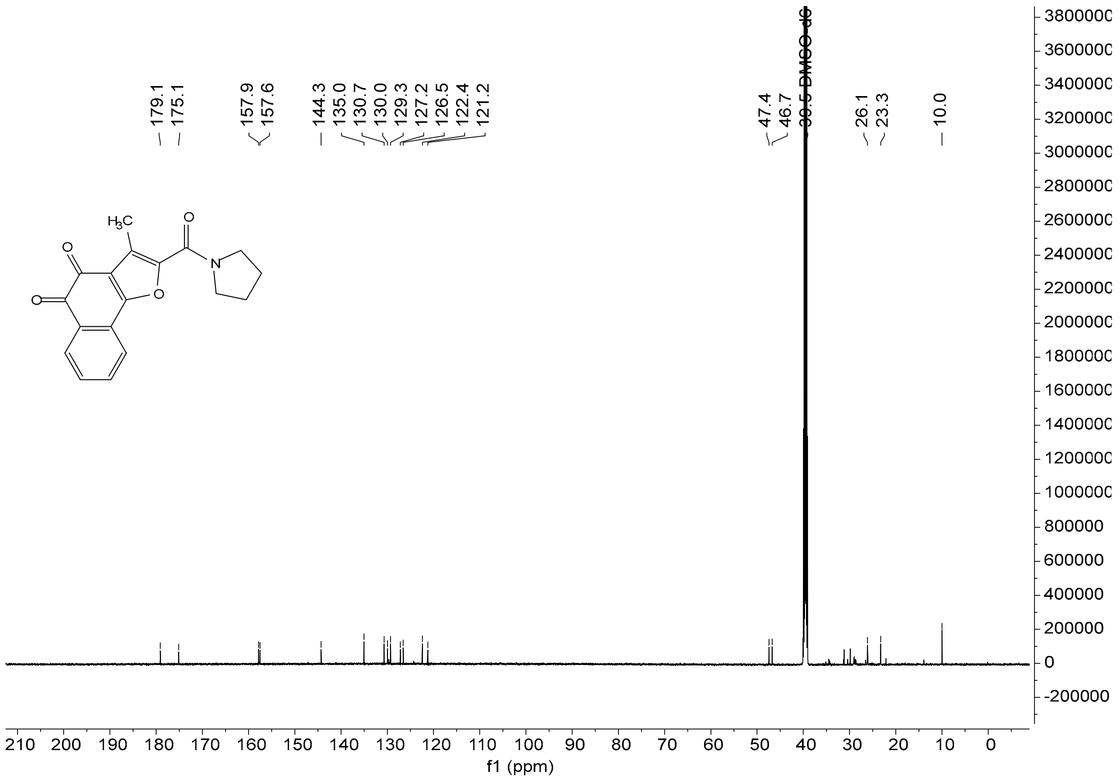

Figure S33. ^{13}C NMR spectrum (500 MHz, DMSO-d_6) of compound **S2-2**.

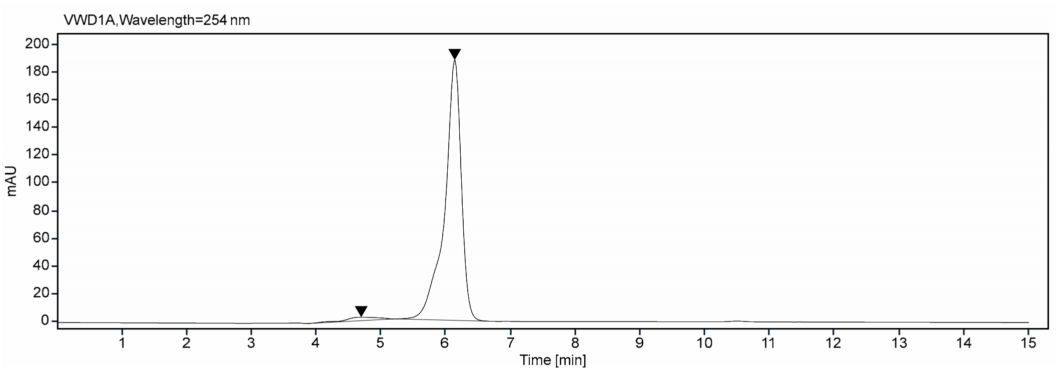

Signal: VWD1A,Wavelength=254 nm

RT [min]	Width [min]	Area	Height	Area%
4.688	1.34	90.01	2.53	2.61
6.132	1.49	3363.13	187.73	97.39
	**Sum**	**3453.14**		

Figure S34. HPLC spectrum of compound **S2-2**.

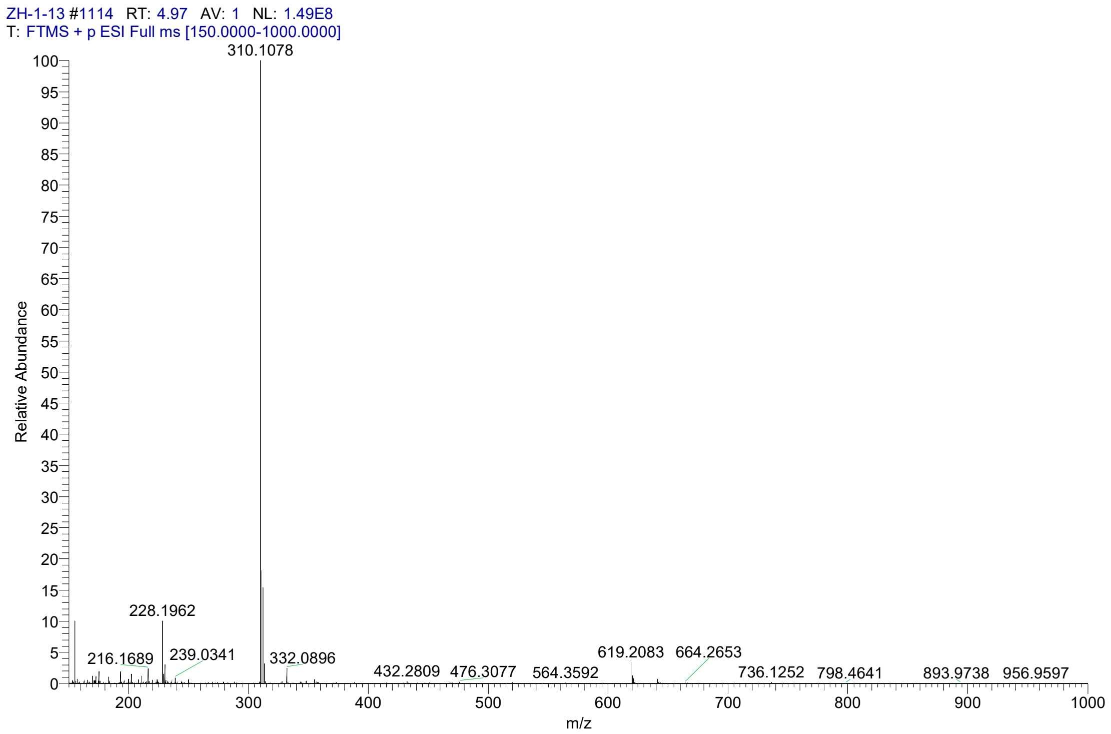

Figure S35. HRMS of compound **S2-2**.

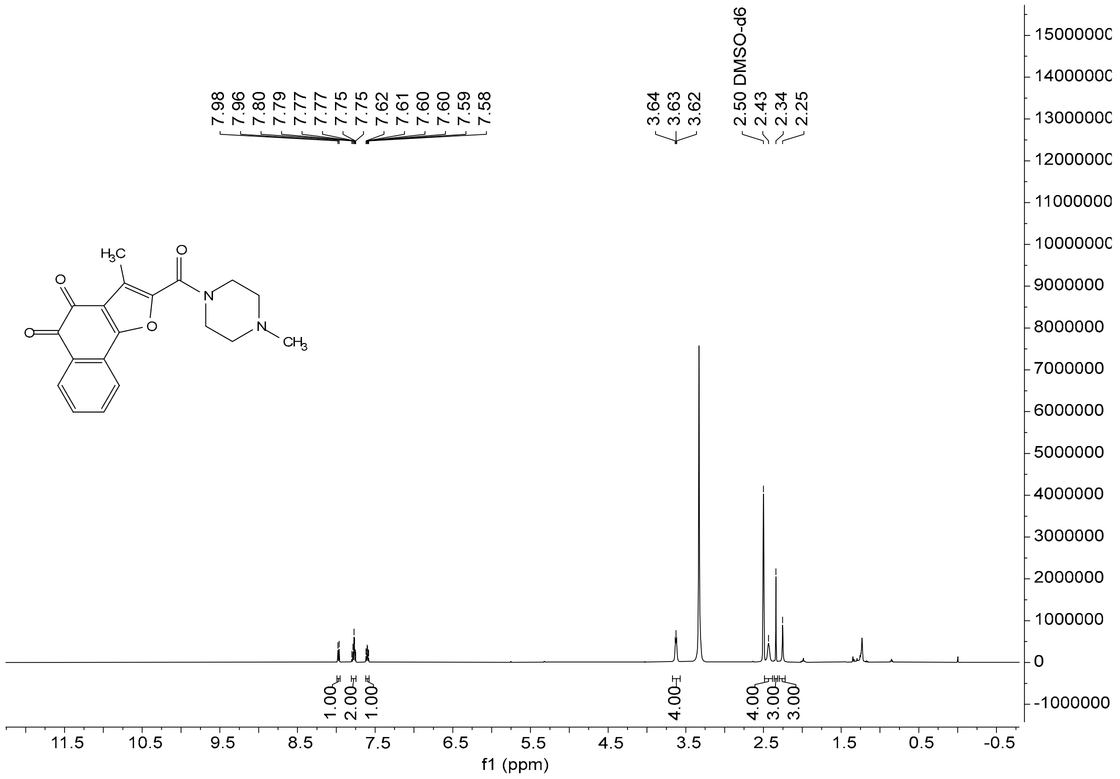

Figure S36. ^{1}H NMR spectrum (500 MHz, DMSO-d_6) of compound **S2-3**.

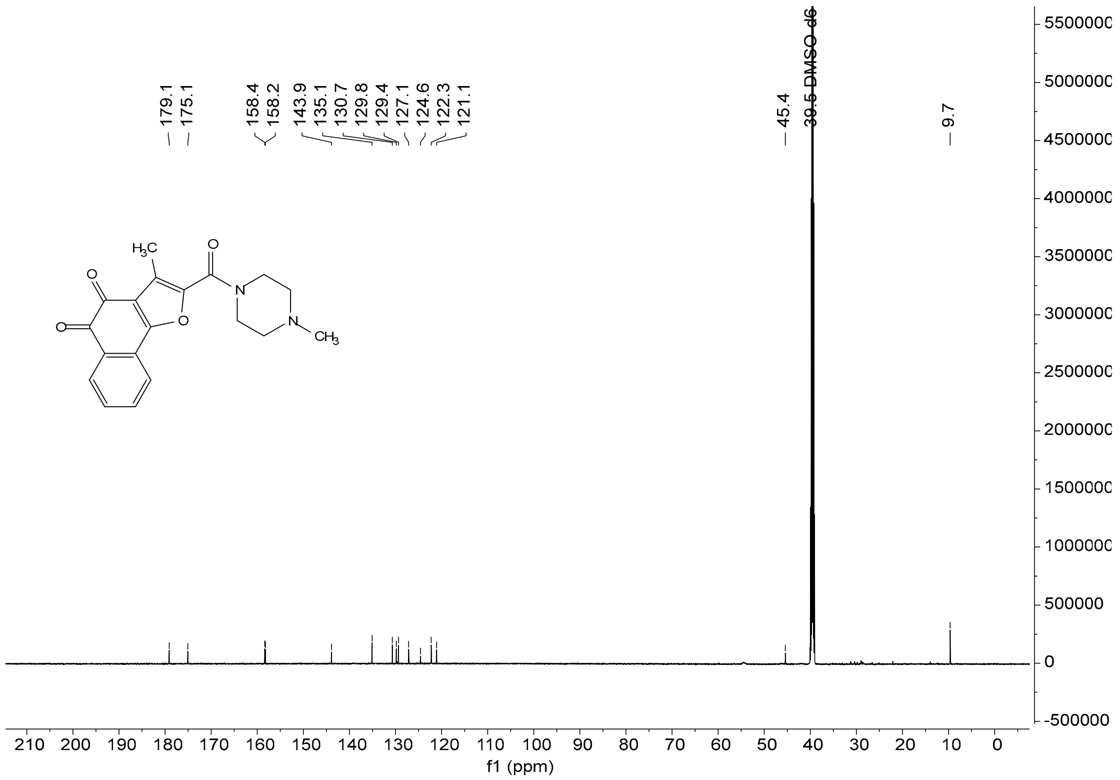

Figure S37. ^{13}C NMR spectrum (500 MHz, DMSO-d_6) of compound **S2-3**.

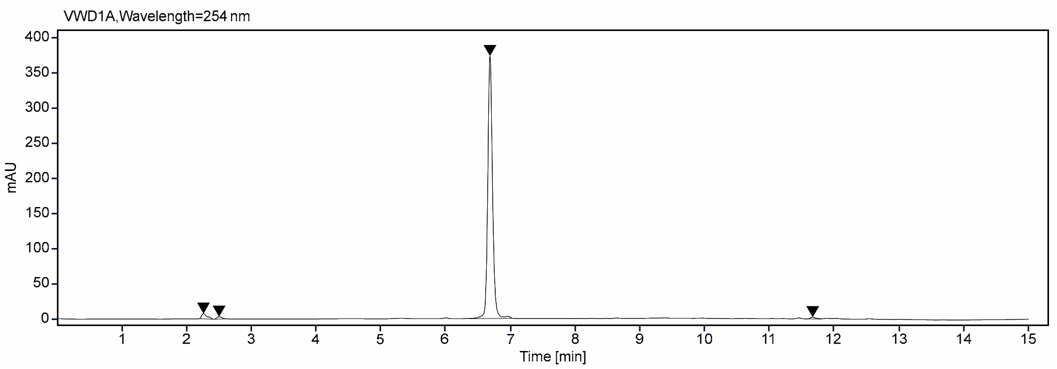

Signal: VWD1A,Wavelength=254 nm

RT [min]	Width [min]	Area	Height	Area%
2.253	0.22	44.36	7.45	2.23
2.494	0.19	17.92	3.32	0.90
6.678	0.67	1912.21	373.39	96.11
11.661	0.25	15.19	2.47	0.76
	**Sum**	**1989.68**		

Figure S38. HPLC spectrum of compound **S2-3**.

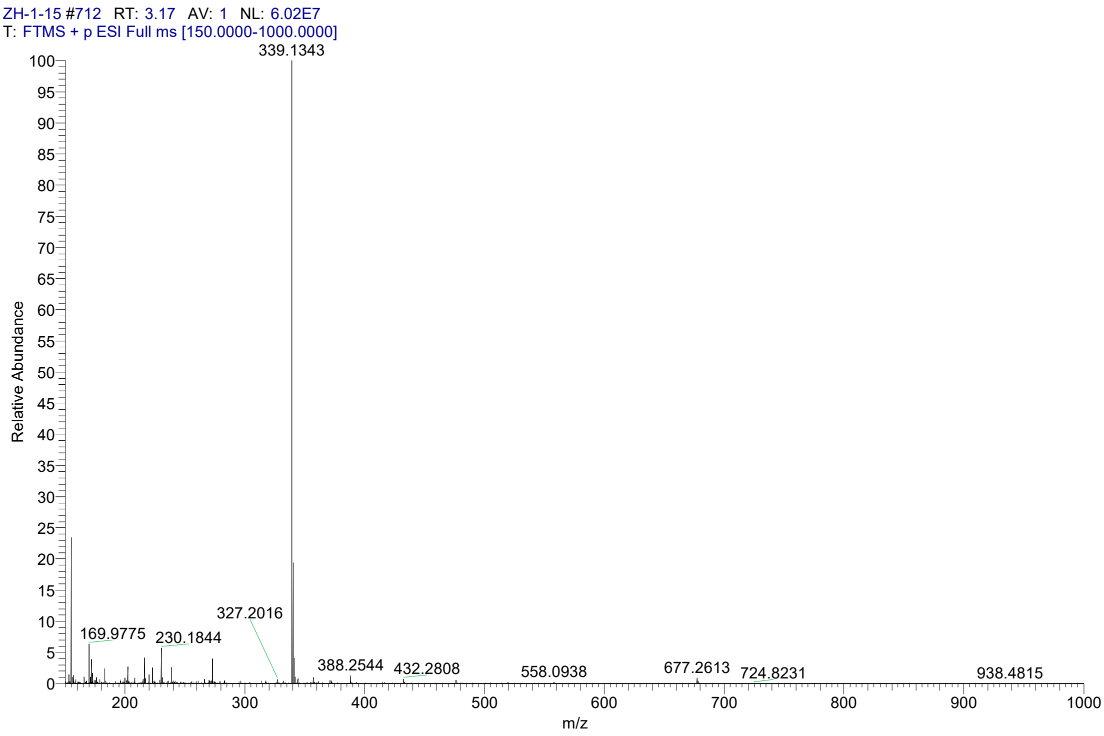

Figure S39. HRMS of compound **S2-3**.

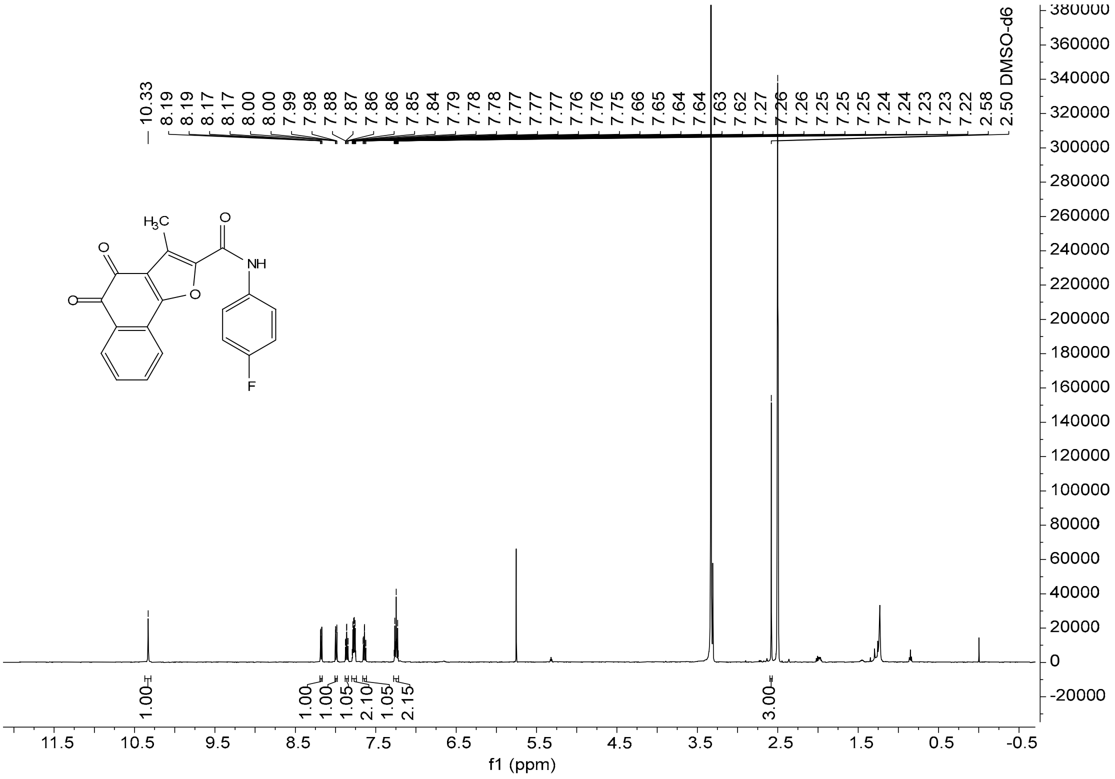

Figure S40. ^{1}H NMR spectrum (500 MHz, DMSO-d_6) of compound **S2-4**.

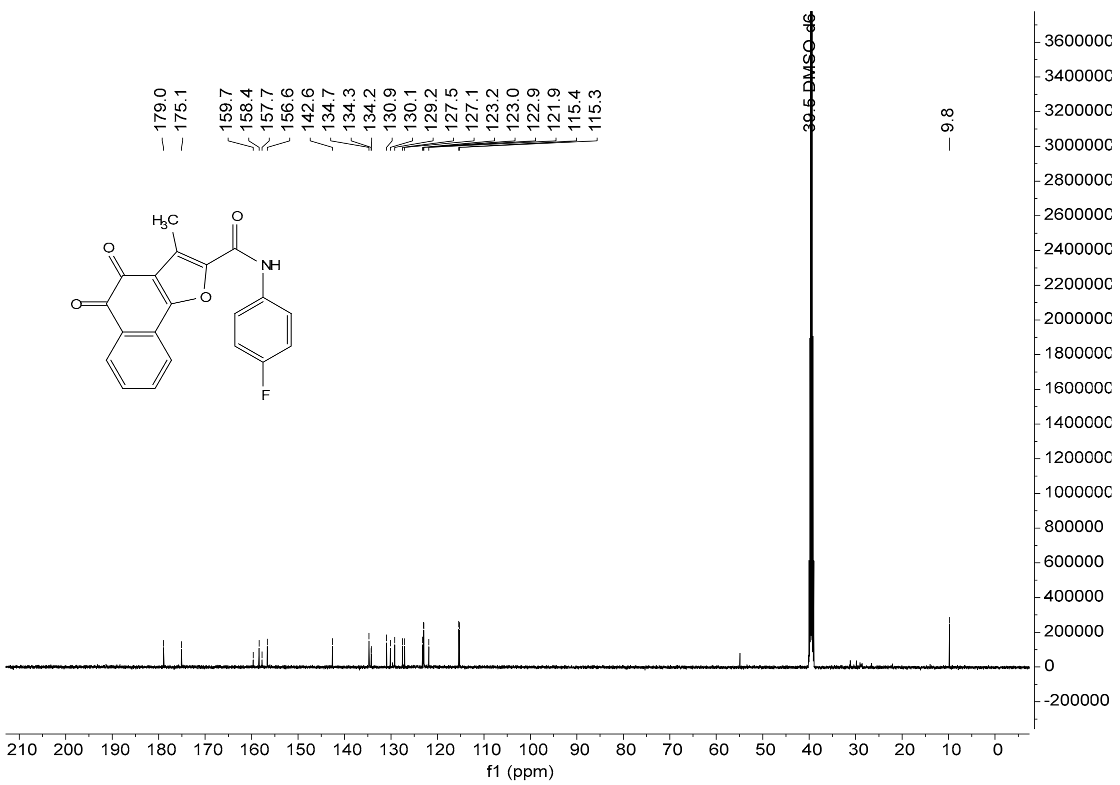

Figure S41. ^{13}C NMR spectrum (500 MHz, DMSO-*d*6) of compound **S2-4**.

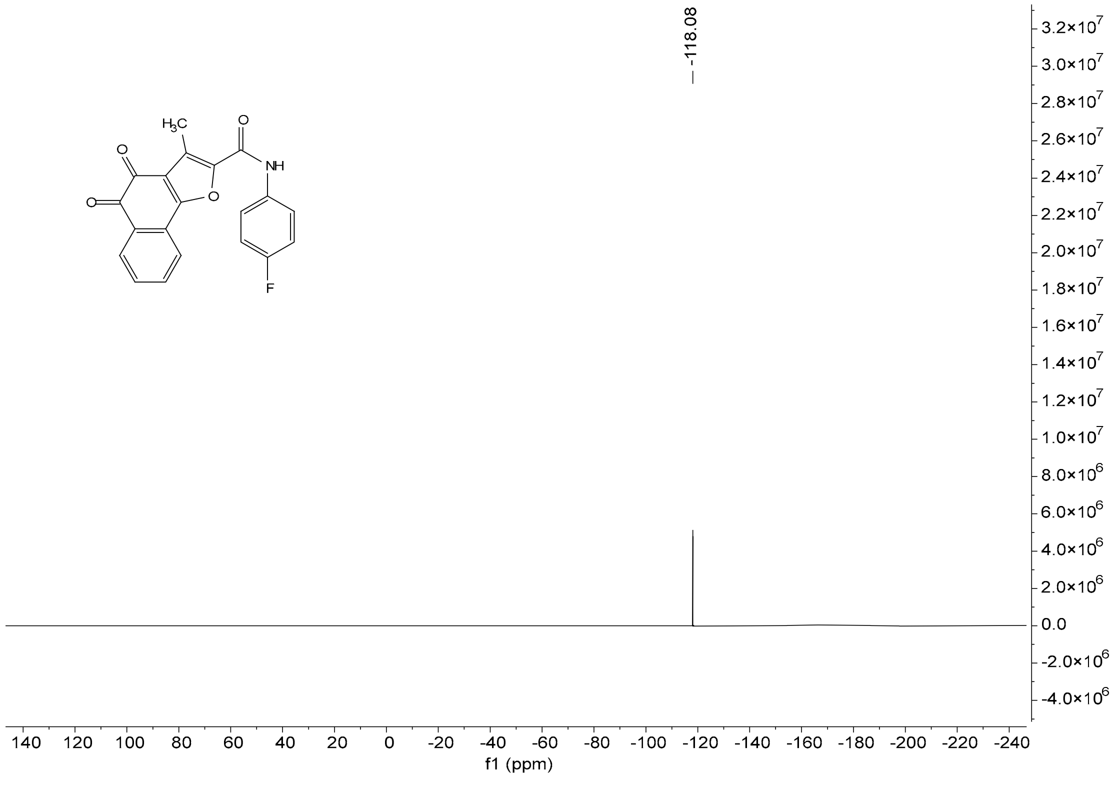

Figure S42. ^{19}F NMR spectrum (500 MHz, DMSO-*d*6) of compound **S2-4**.

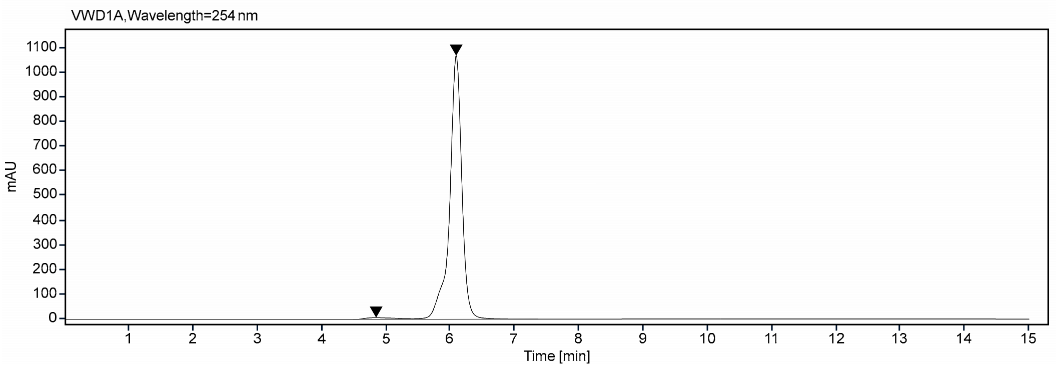

Signal: VWD1A,Wavelength=254 nm

RT [min]	Width [min]	Area	Height	Area%
4.836	0.93	208.36	6.46	1.45
6.083	1.96	14167.33	1068.86	98.55
	**Sum**	**14375.69**		

Figure S43. HPLC spectrum of compound **S2-4**.

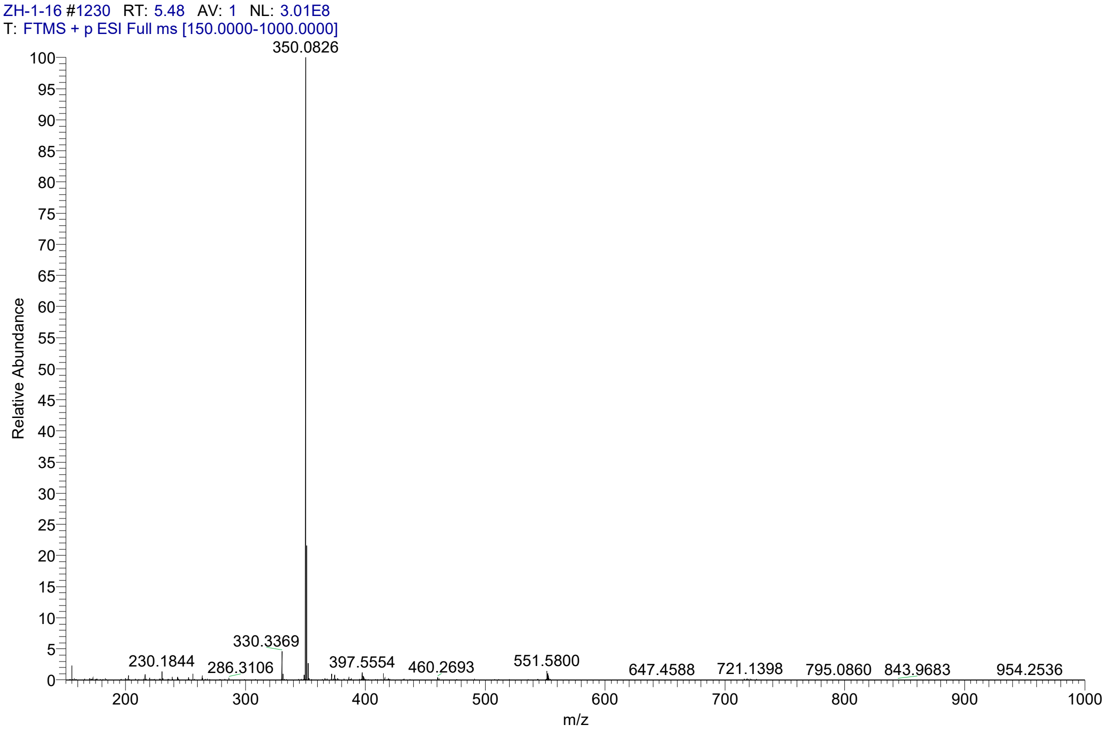

Figure S44. HRMS of compound **S2-4**.

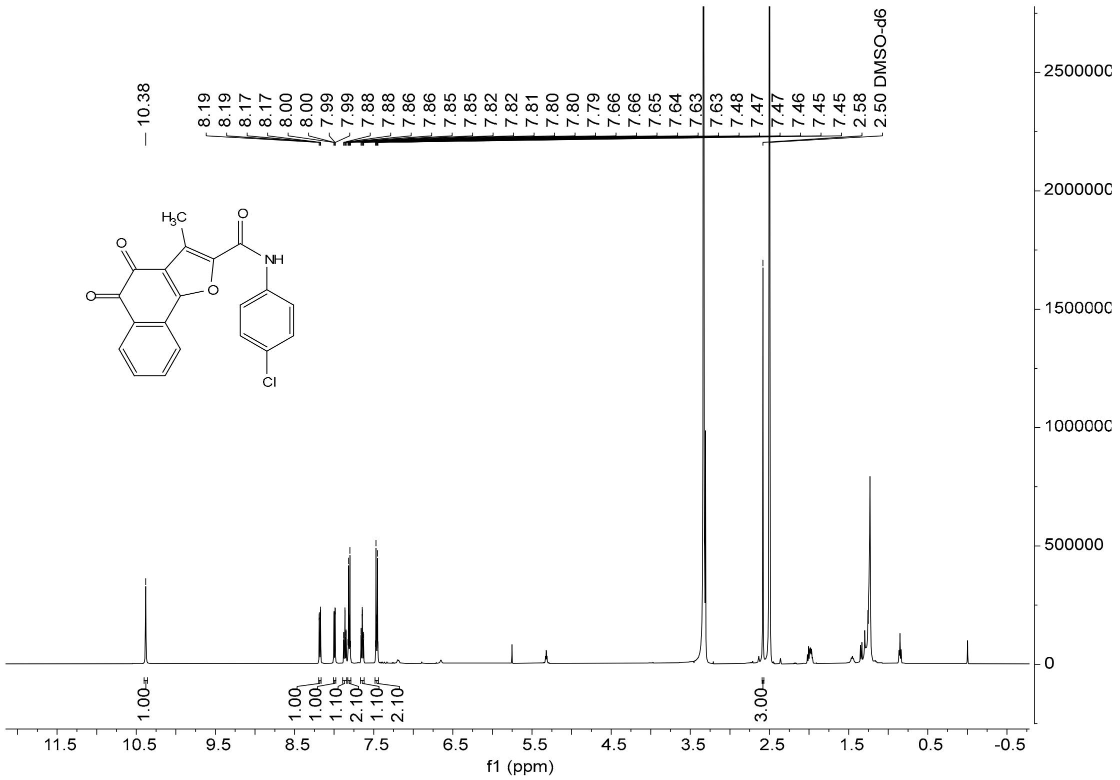

Figure S45. ¹H NMR spectrum (500 MHz, DMSO-*d*₆) of compound **S2-5**.

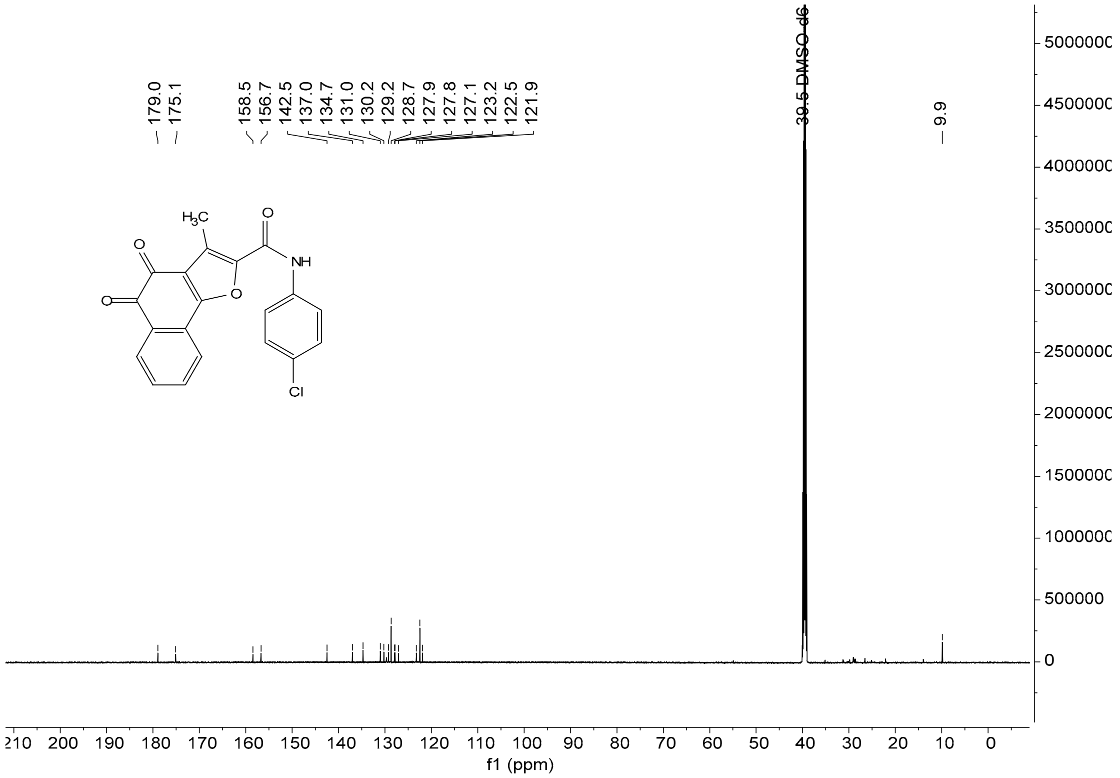

Figure S46. ¹³C NMR spectrum (500 MHz, DMSO-*d*₆) of compound **S2-5**.

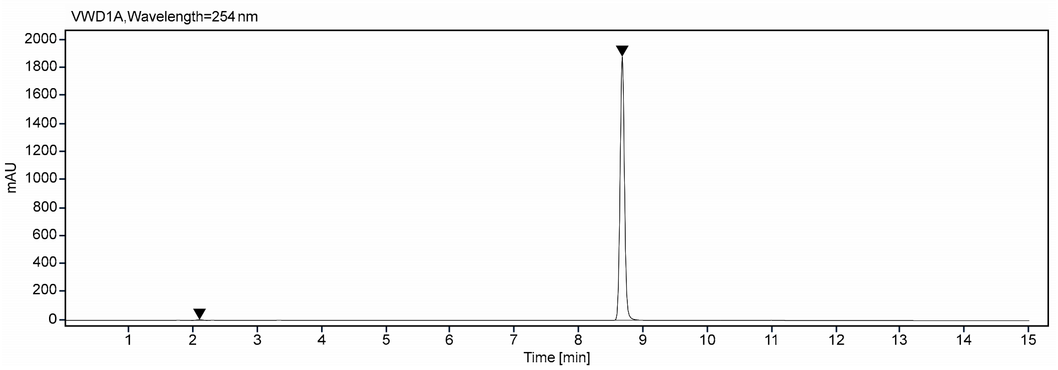

Signal: VWD1A,Wavelength=254 nm

RT [min]	Width [min]	Area	Height	Area%
2.089	0.25	21.24	3.10	0.24
8.667	0.39	8892.41	1878.77	99.76
	**Sum**	**8913.66**		

Figure S47. HPLC spectrum of compound **S2-5**.

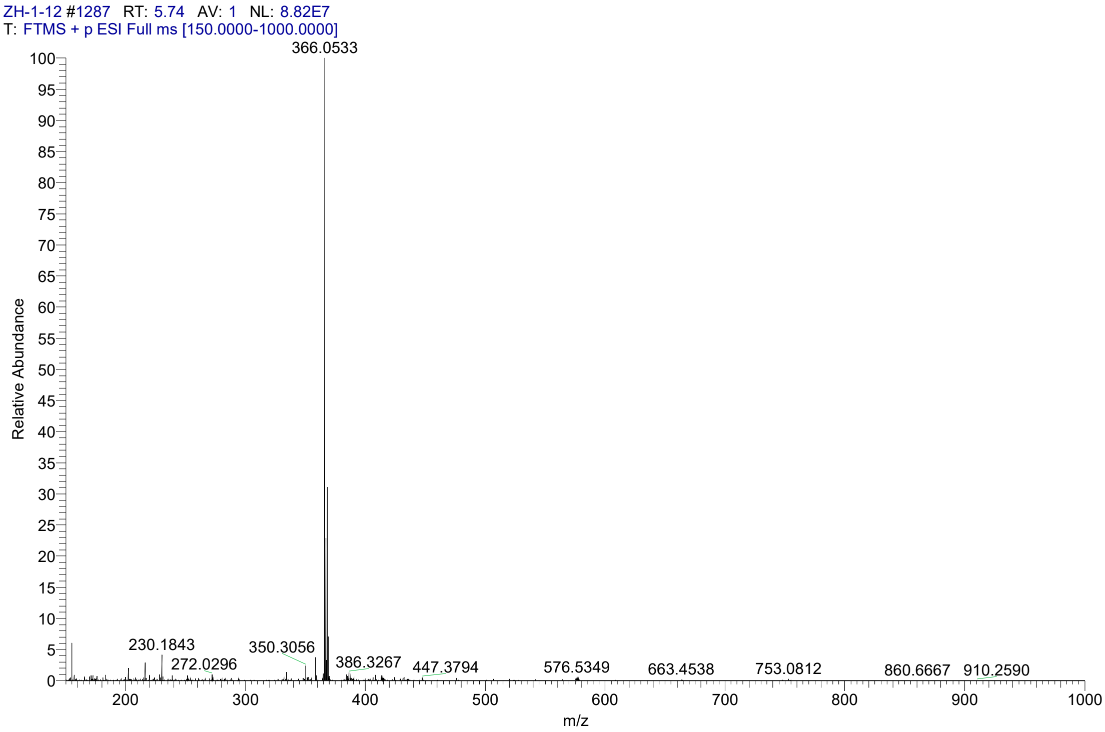

Figure S48. HRMS of compound **S2-5**.

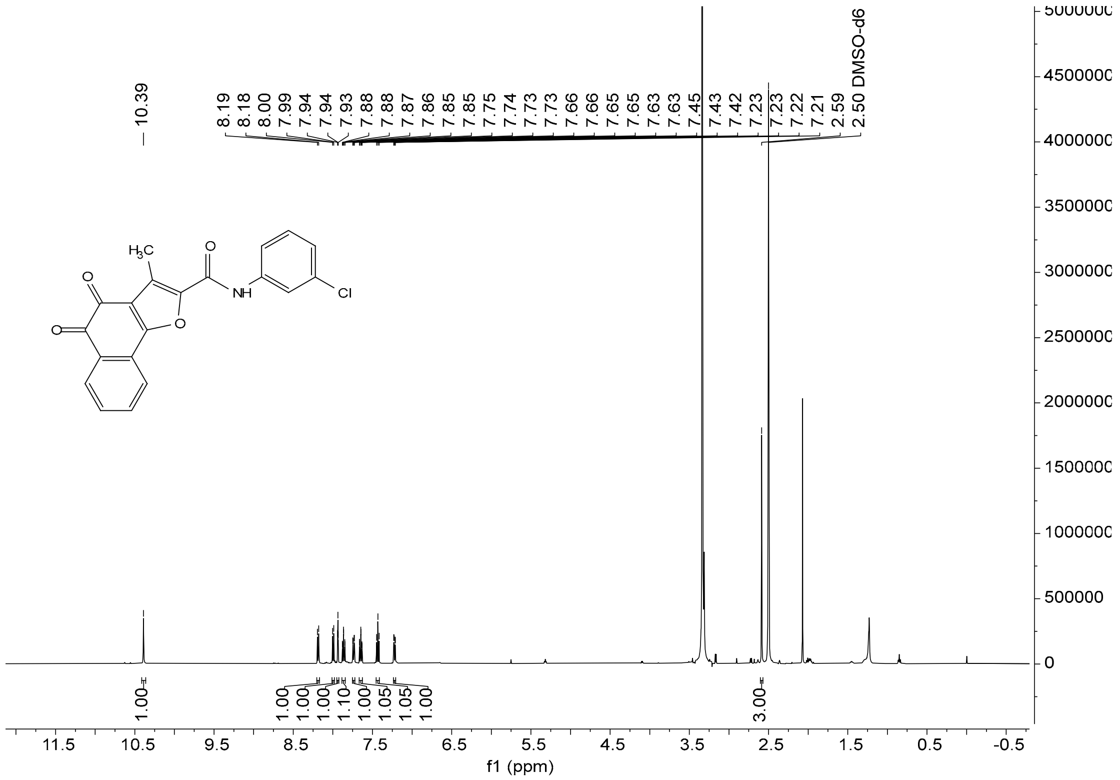

Figure S49. ^{1}H NMR spectrum (500 MHz, DMSO-d_6) of compound **S2-6**.

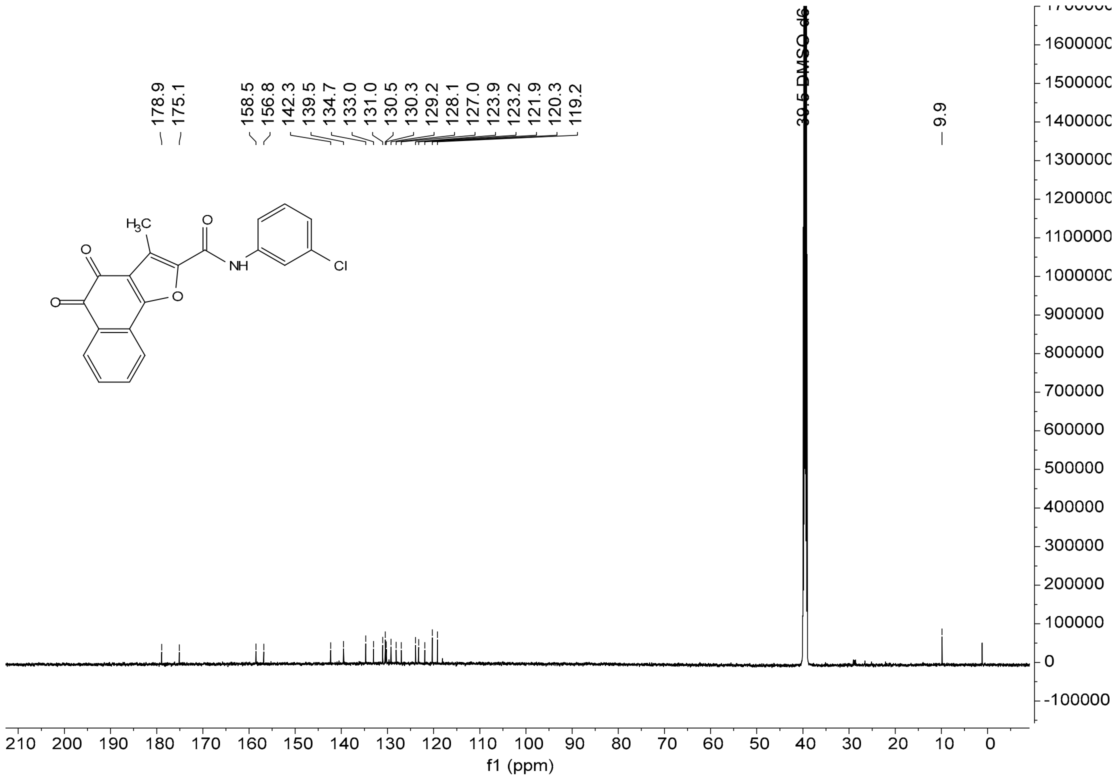

Figure S50. ^{13}C NMR spectrum (500 MHz, DMSO-d_6) of compound **S2-6**.

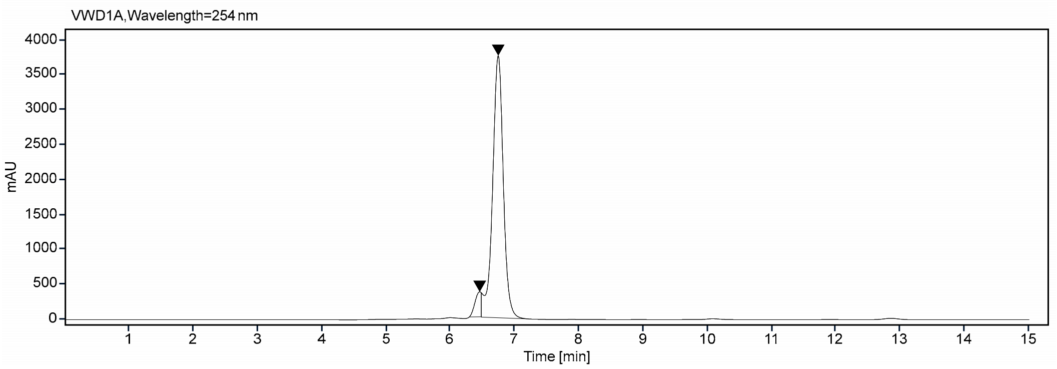

Signal: VWD1A,Wavelength=254 nm

RT [min]	Width [min]	Area	Height	Area%
6.450	0.18	2228.86	357.23	4.86
6.738	0.71	43626.75	3751.81	95.14
	**Sum**	**45855.61**		

Figure S51. HPLC spectrum of compound **S2-6**.

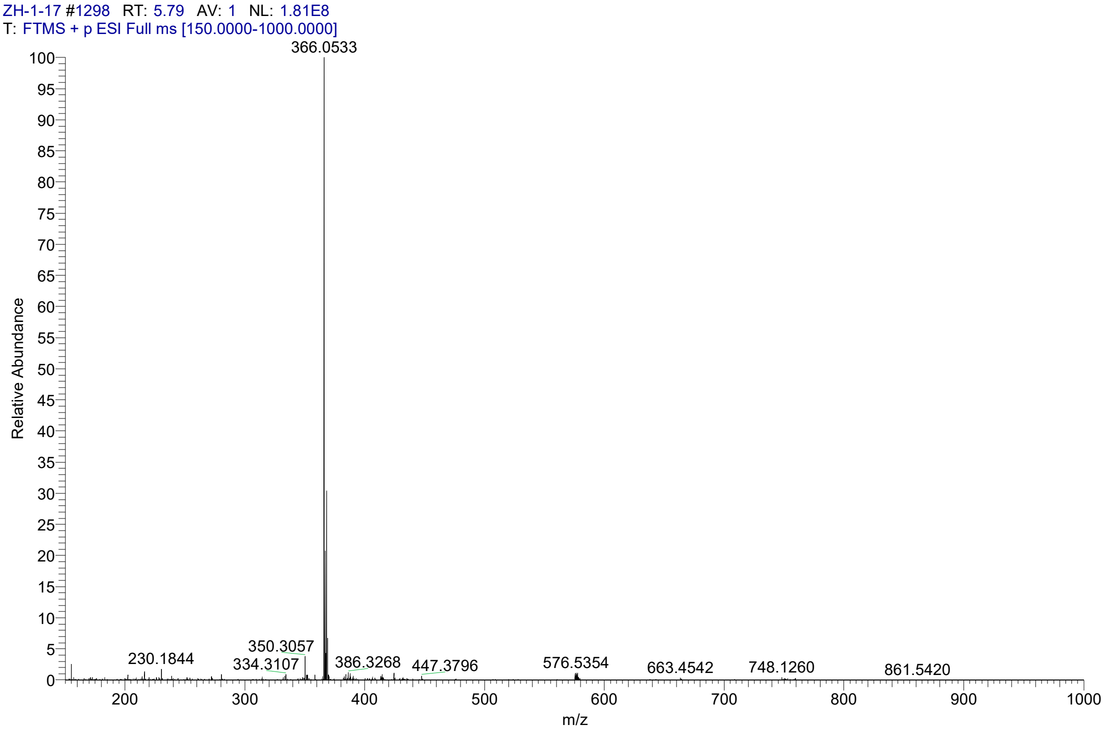

Figure S52. HRMS of compound **S2-6**.

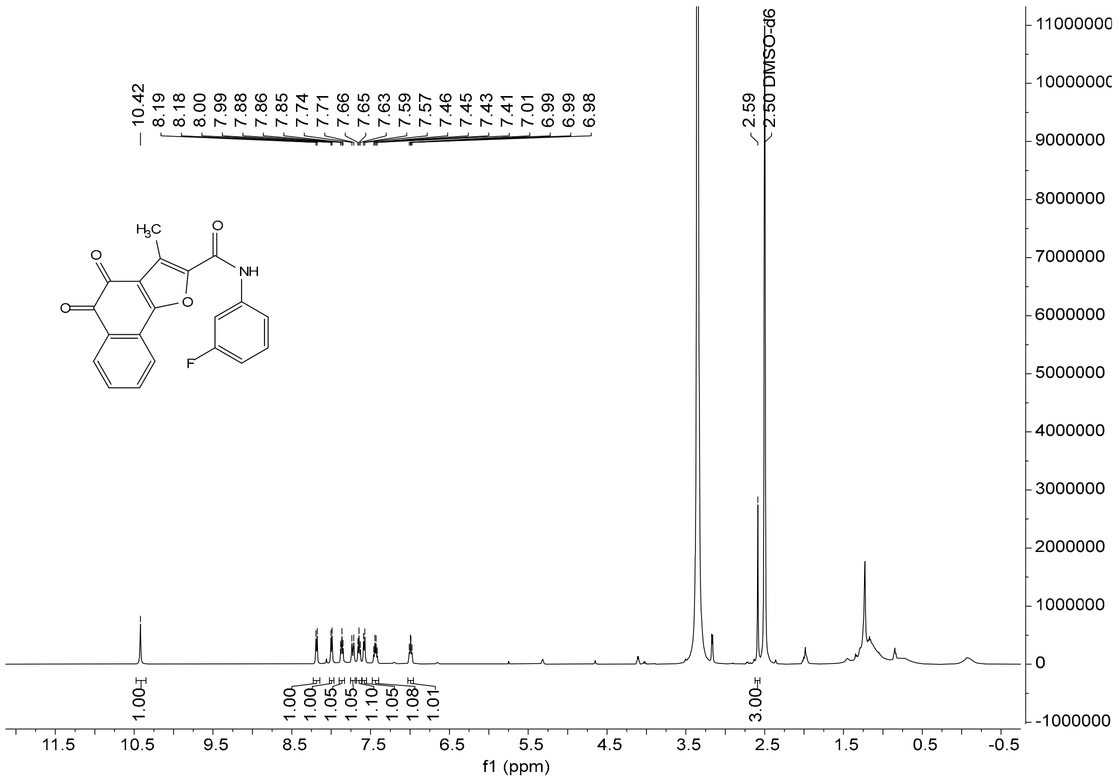

Figure S53. ^{1}H NMR spectrum (500 MHz, DMSO-d_6) of compound **S2-7**.

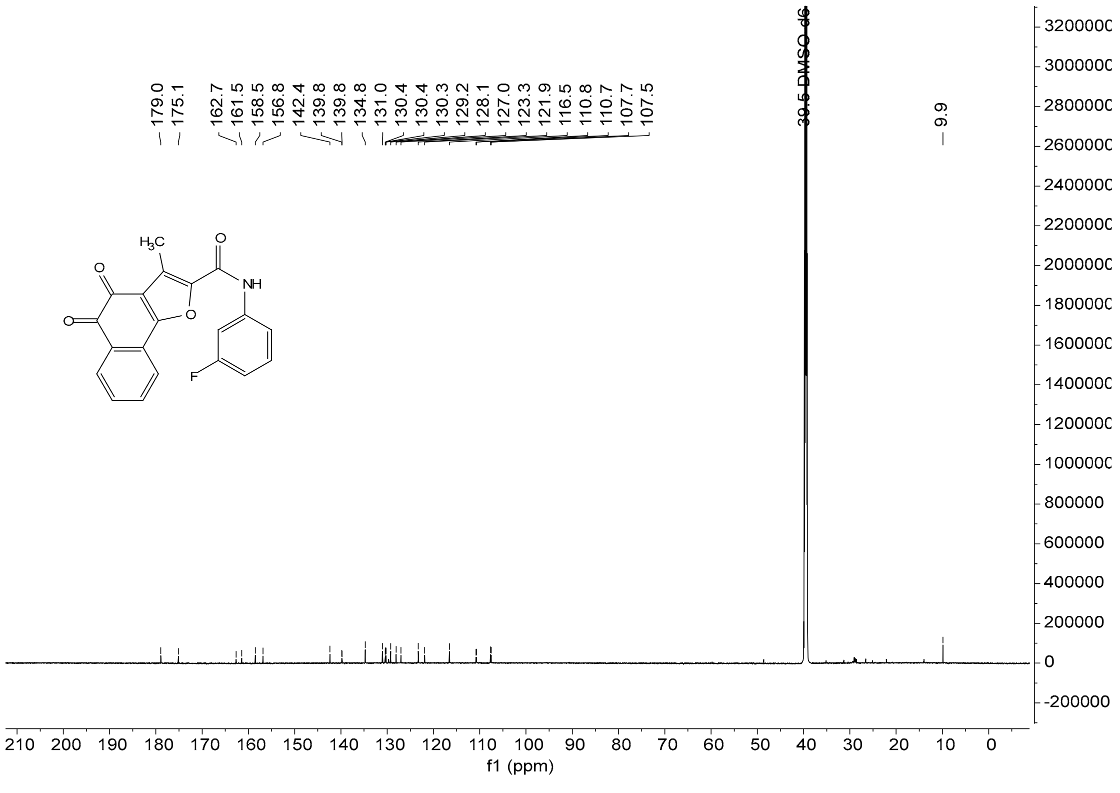

Figure S54. ^{13}C NMR spectrum (500 MHz, DMSO-d_6) of compound **S2-7**.

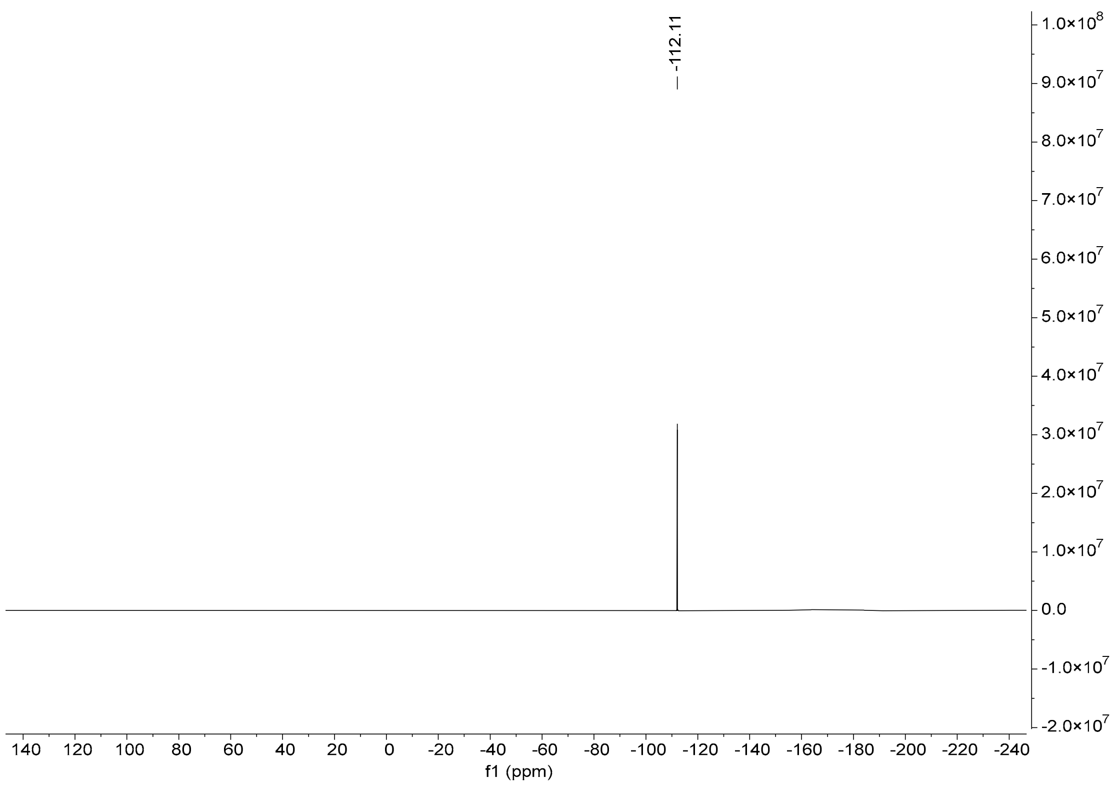

Figure S55. ^{19}F NMR spectrum (500 MHz, DMSO-d_6) of compound **S2-7**.

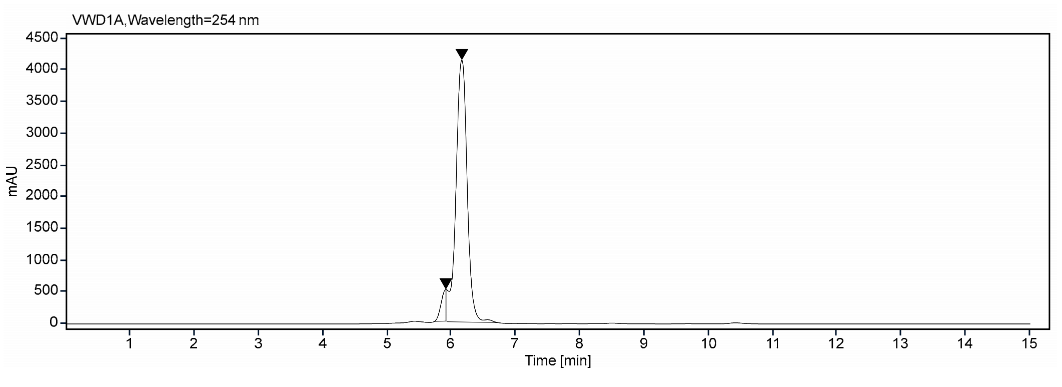

Signal: VWD1A,Wavelength=254 nm

RT [min]	Width [min]	Area	Height	Area%
5.906	0.18	2531.98	497.09	4.78
6.155	0.80	50384.83	4130.70	95.22
	**Sum**	**52916.81**		

Figure S56. HPLC spectrum of compound **S2-7**.

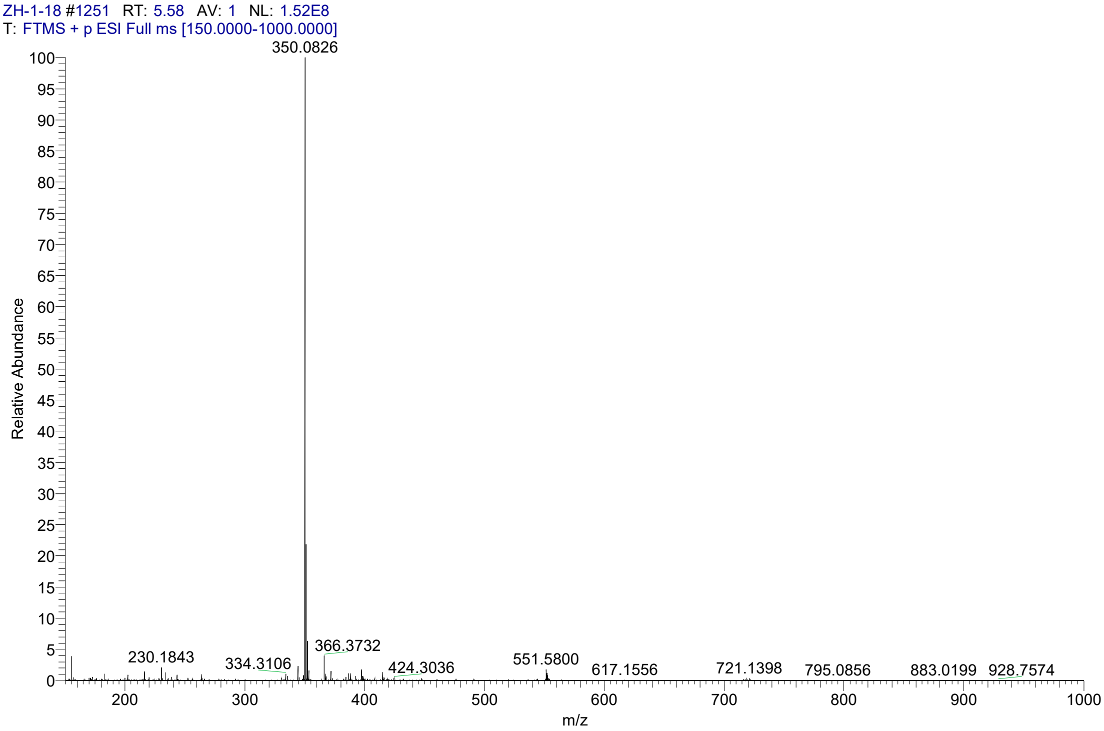

Figure S57. HRMS of compound **S2-7**.

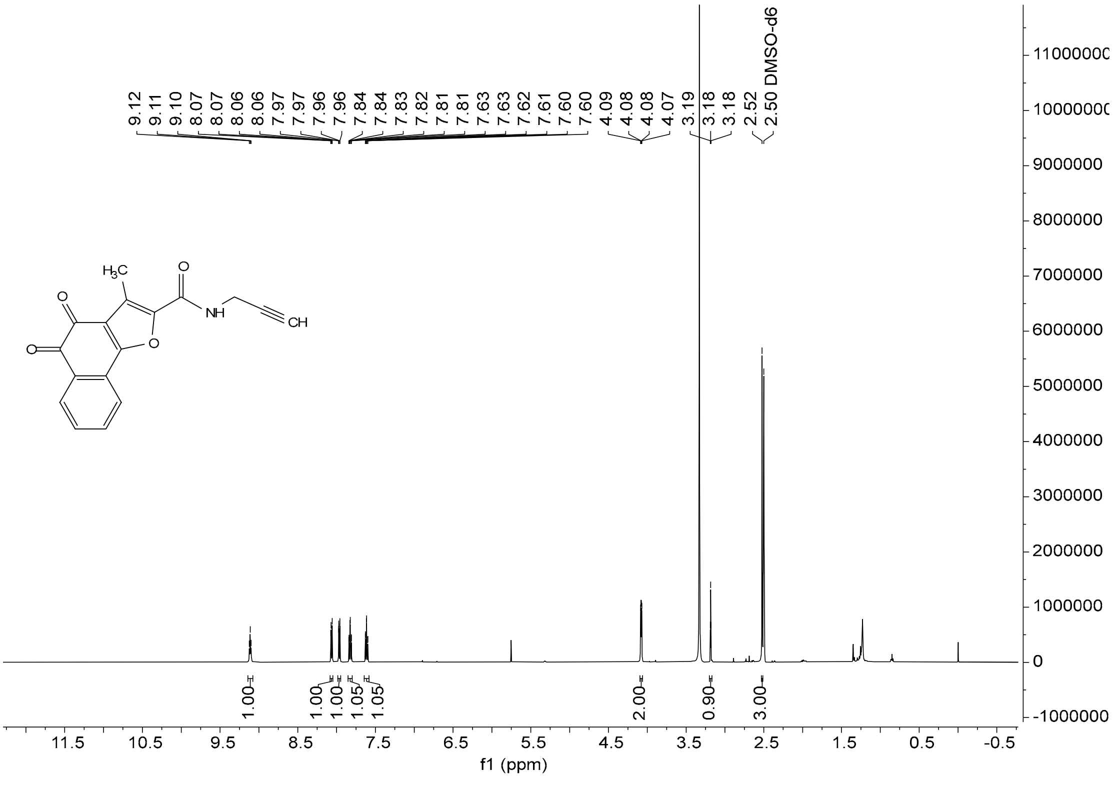

Figure S58. ¹H NMR spectrum (500 MHz, DMSO-d_6) of compound **S2-8**.

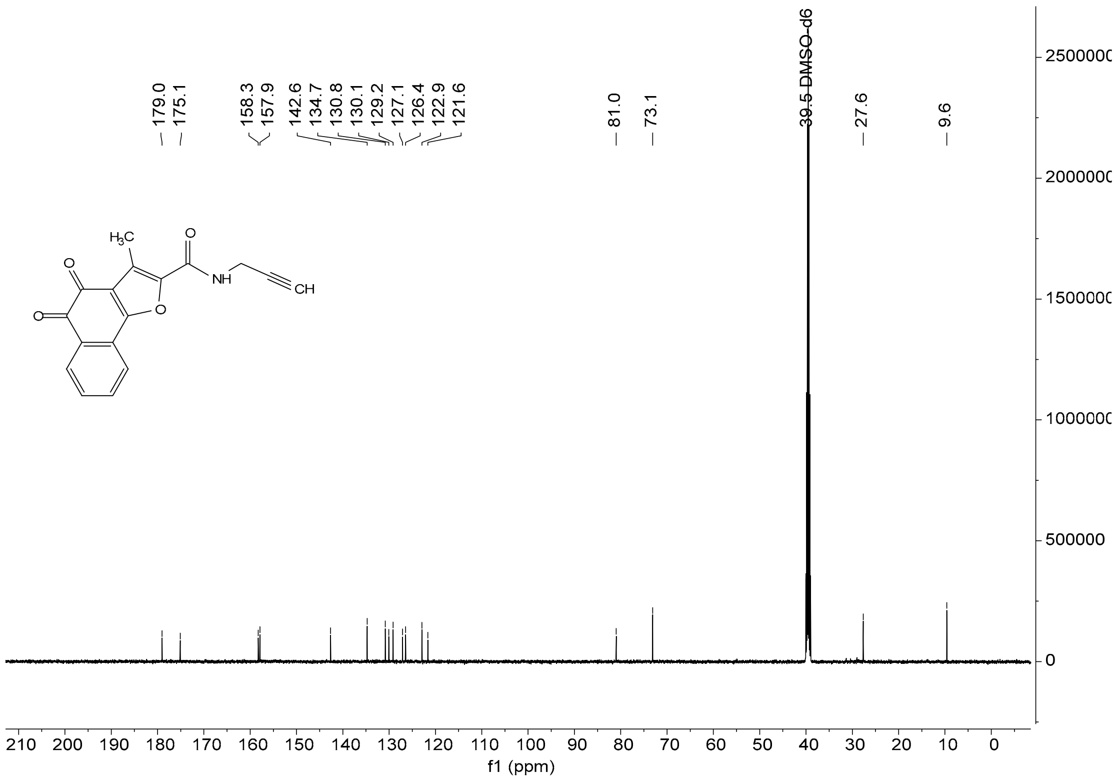

Figure S59. ^{13}C NMR spectrum (500 MHz, DMSO-d_6) of compound **S2-8**.

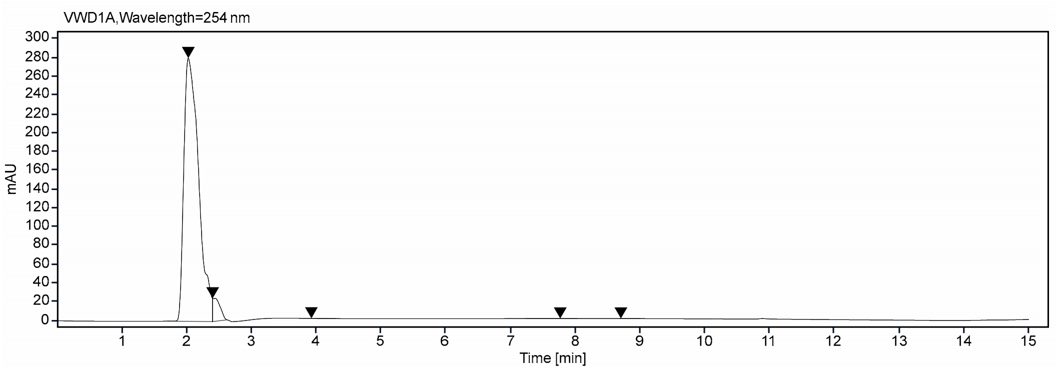

Signal: VWD1A,Wavelength=254 nm

RT [min]	Width [min]	Area	Height	Area%
2.019	0.70	4422.80	280.23	95.80
2.396	0.22	192.69	24.69	4.17
3.919	0.58	0.45	0.02	0.01
7.763	0.80	0.46	0.02	0.01
8.699	0.32	0.08	0.01	0.00
	**Sum**	**4616.48**		

Figure S60. HPLC spectrum of compound **S2-8**.

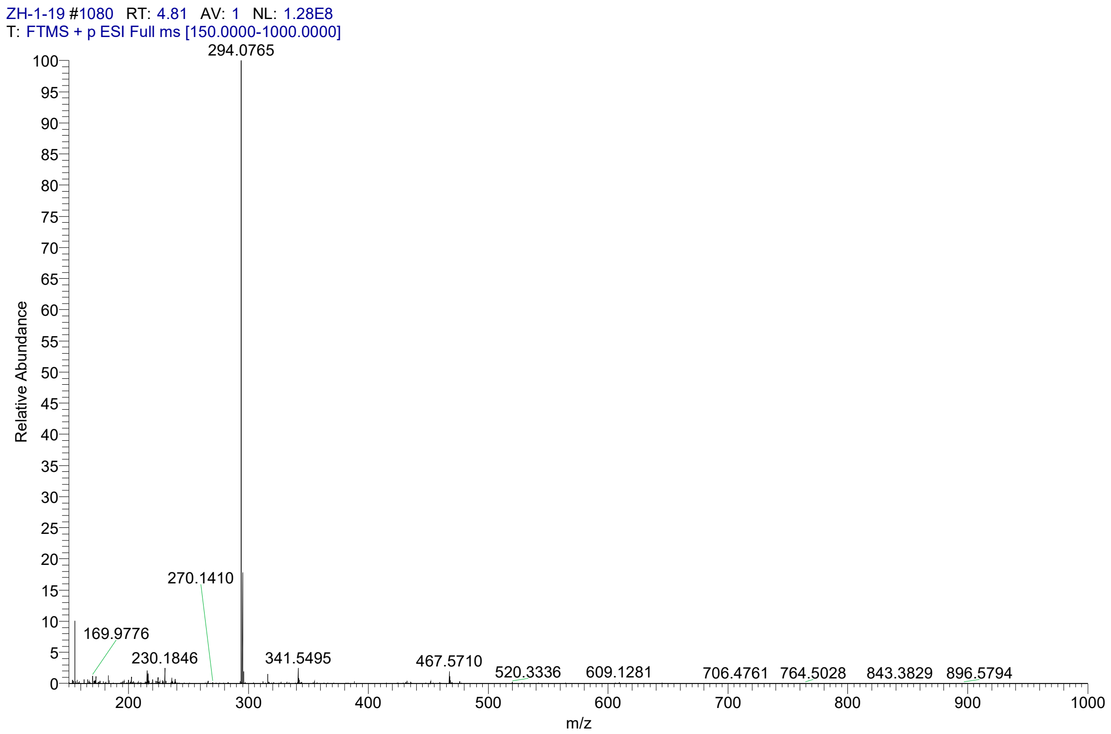

Figure S61. HRMS of compound **S2-8**.

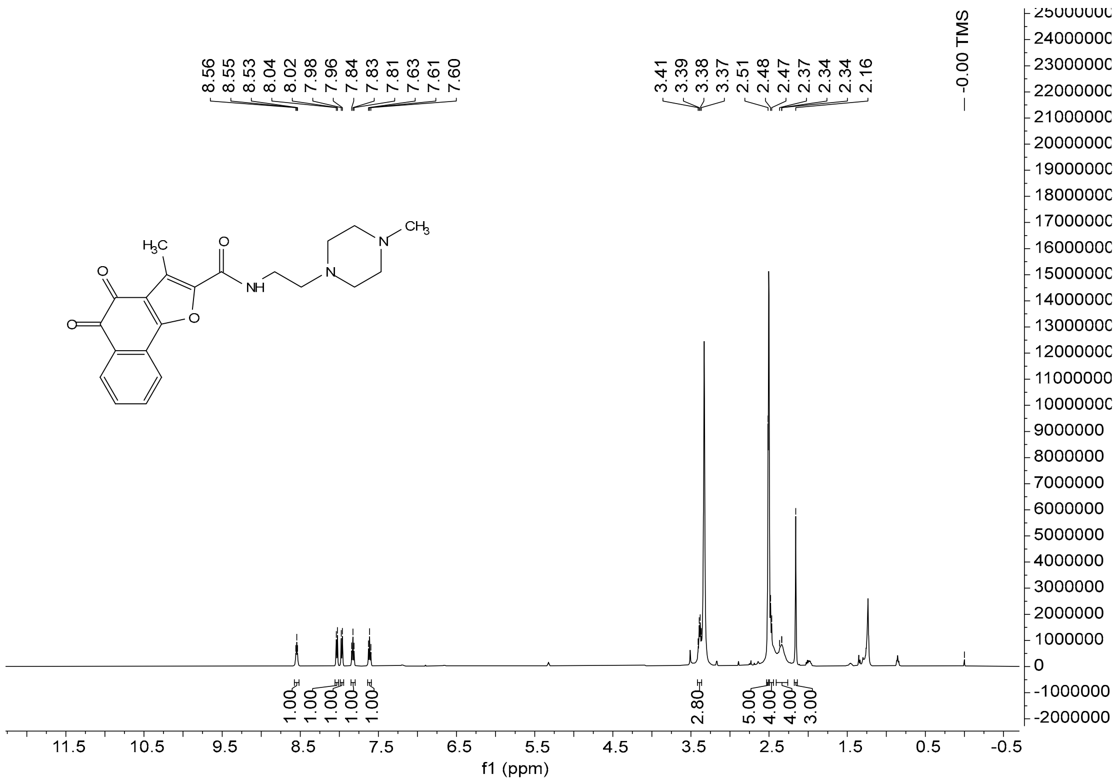

Figure S62. ^{1}H NMR spectrum (500 MHz, DMSO-d_6) of compound **S2-9**.

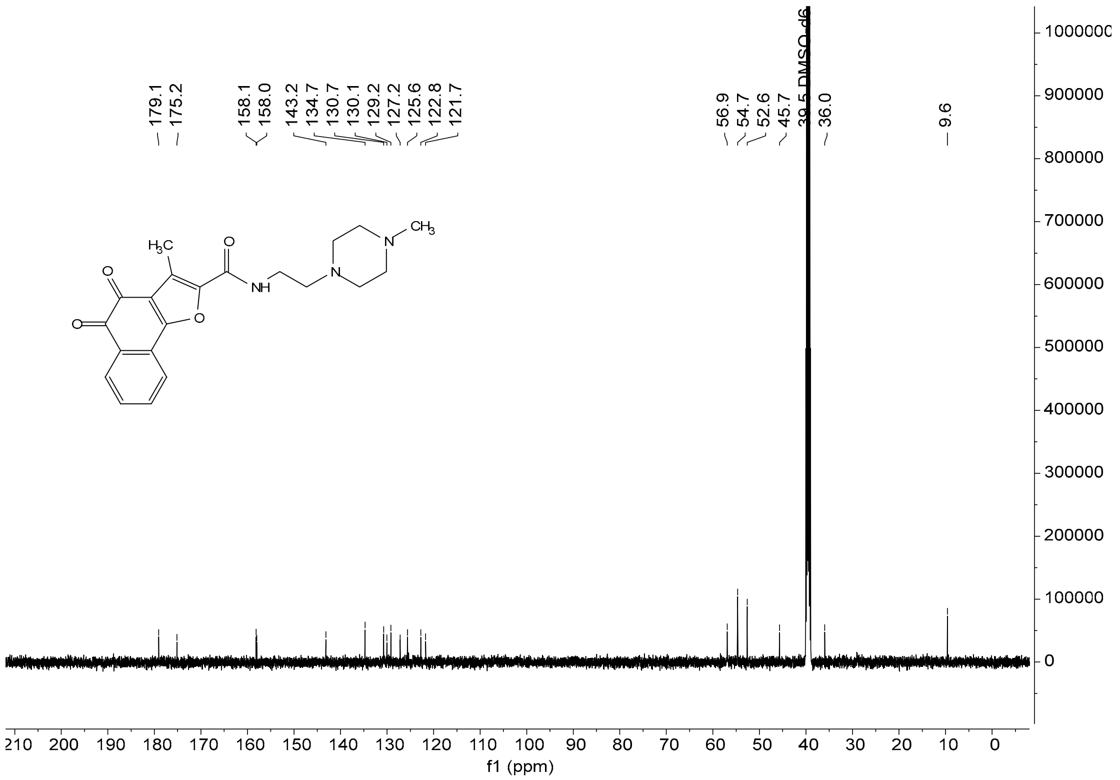

Figure S63. ^{13}C NMR spectrum (500 MHz, DMSO-d_6) of compound **S2-9**.

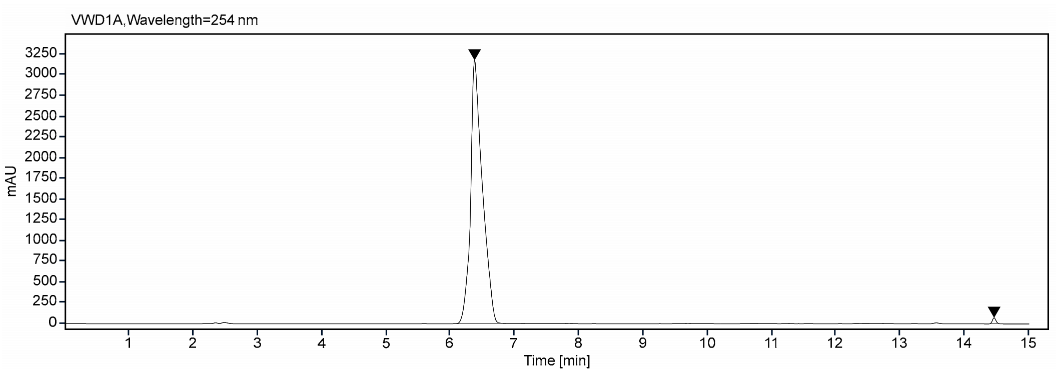

Signal: VWD1A,Wavelength=254 nm

RT [min]	Width [min]	Area	Height	Area%
6.371	0.70	40716.16	3178.01	99.42
14.455	0.12	237.90	67.14	0.58
	**Sum**	**40954.06**		

Figure S64. HPLC spectrum of compound **S2-9**.

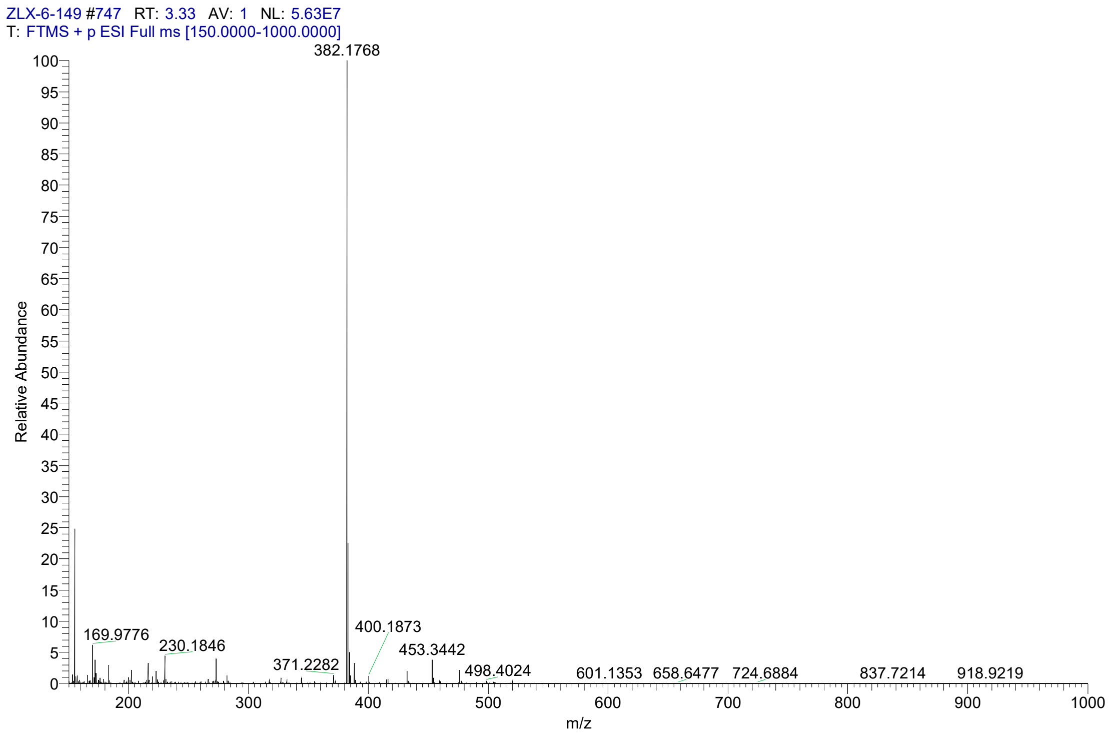

Figure S65. HRMS of compound **S2-9**.

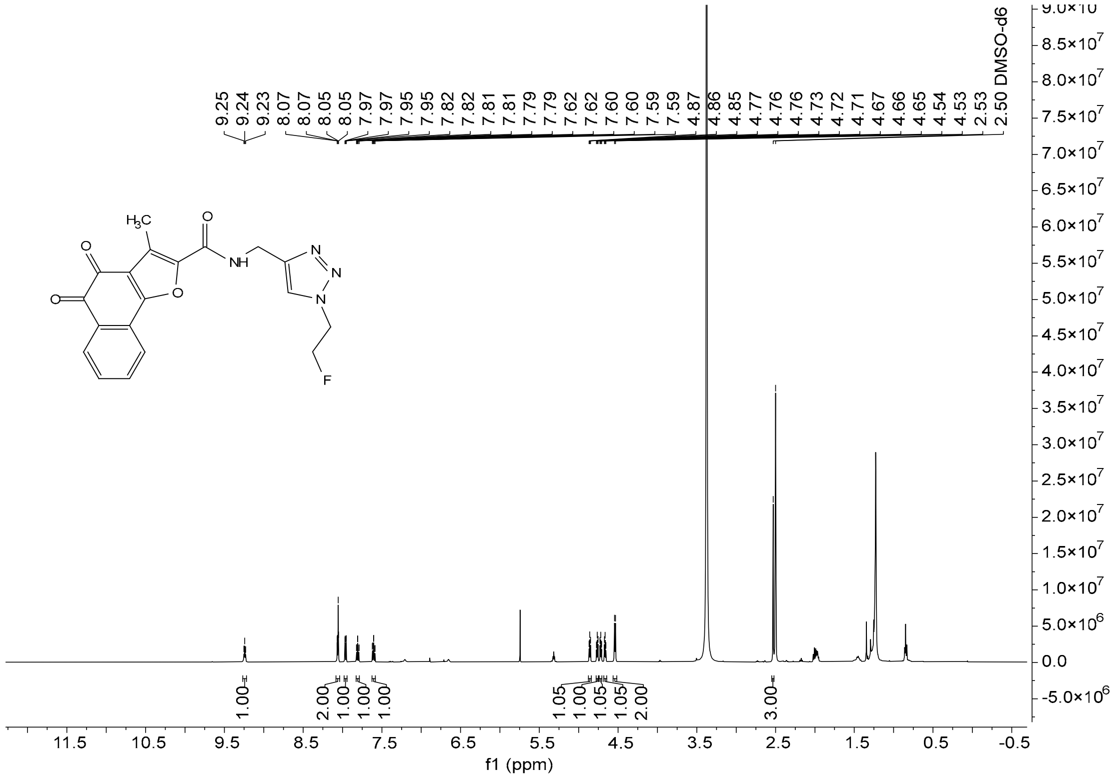

Figure S66. ^{1}H NMR spectrum (500 MHz, DMSO-*d*6) of compound **S2-10**.

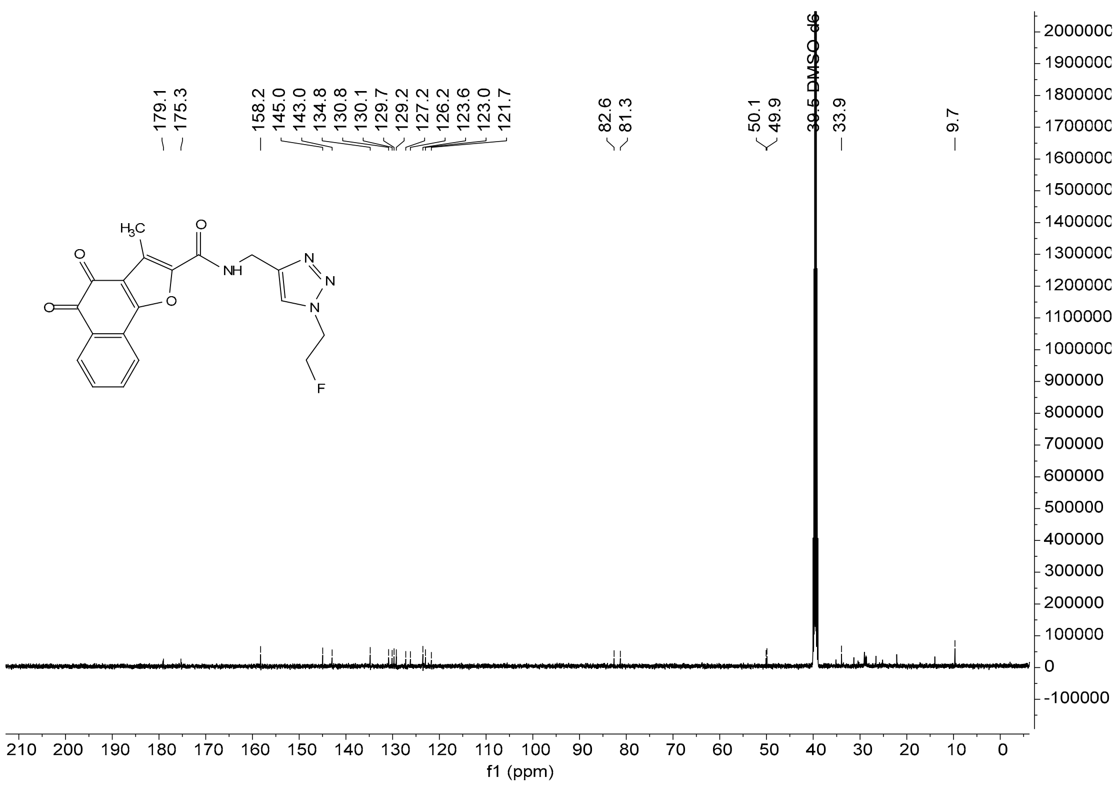

Figure S67. ^{13}C NMR spectrum (500 MHz, DMSO-d_6) of compound **S2-10**.

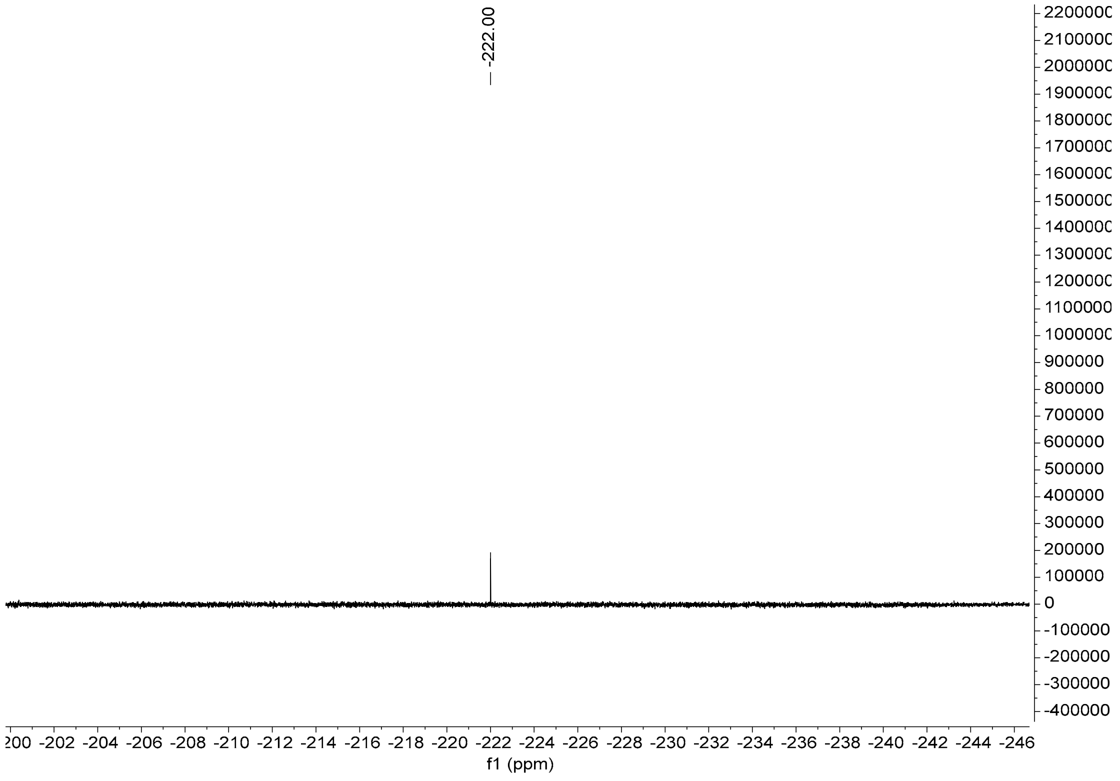

Figure S68. ^{19}F NMR spectrum (500 MHz, DMSO-d_6) of compound **S2-10**.

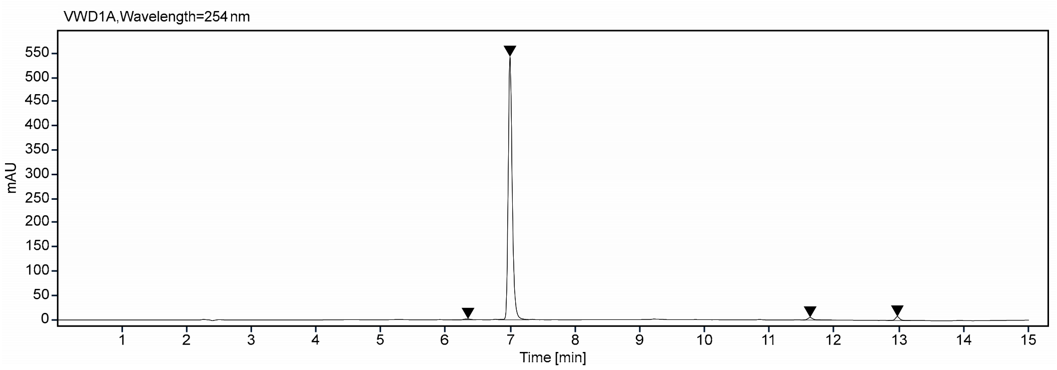

Signal: VWD1A,Wavelength=254 nm

RT [min]	Width [min]	Area	Height	Area%
6.338	0.37	13.43	1.76	0.55
6.987	0.48	2358.15	543.97	96.56
11.621	0.52	32.06	5.22	1.31
12.968	0.36	38.53	7.99	1.58
	**Sum**	**2442.17**		

Figure S69. HPLC spectrum of compound **S2-10**.

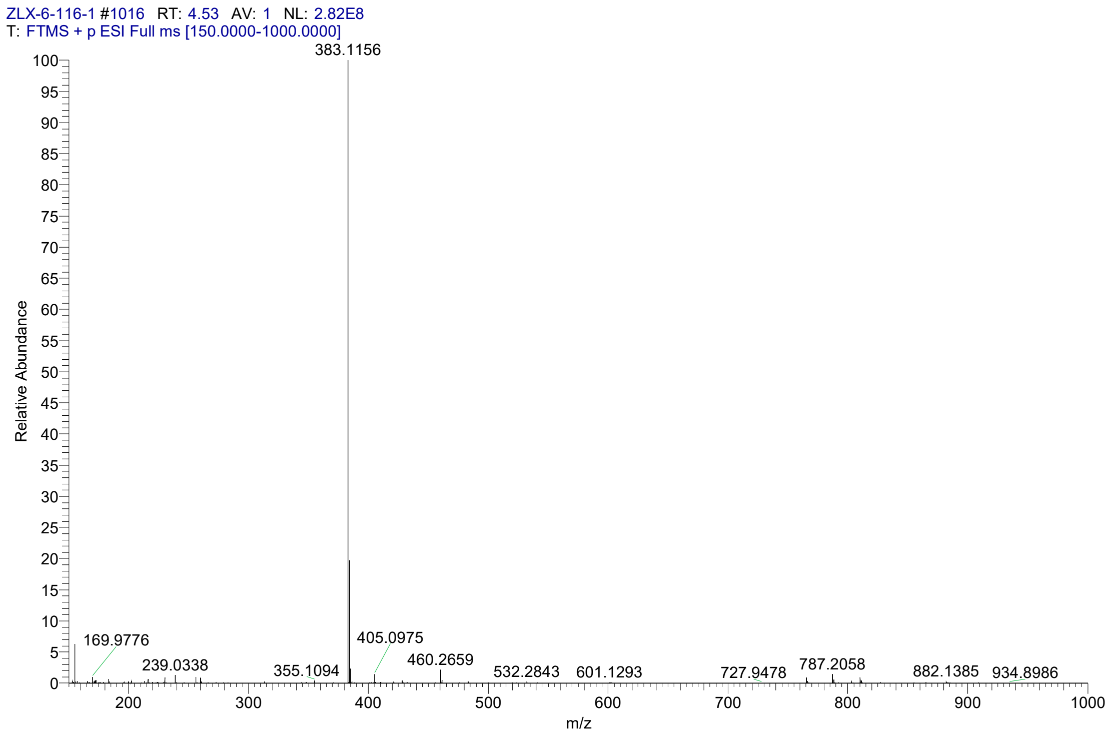

Figure S70. HRMS of compound **S2-10**.

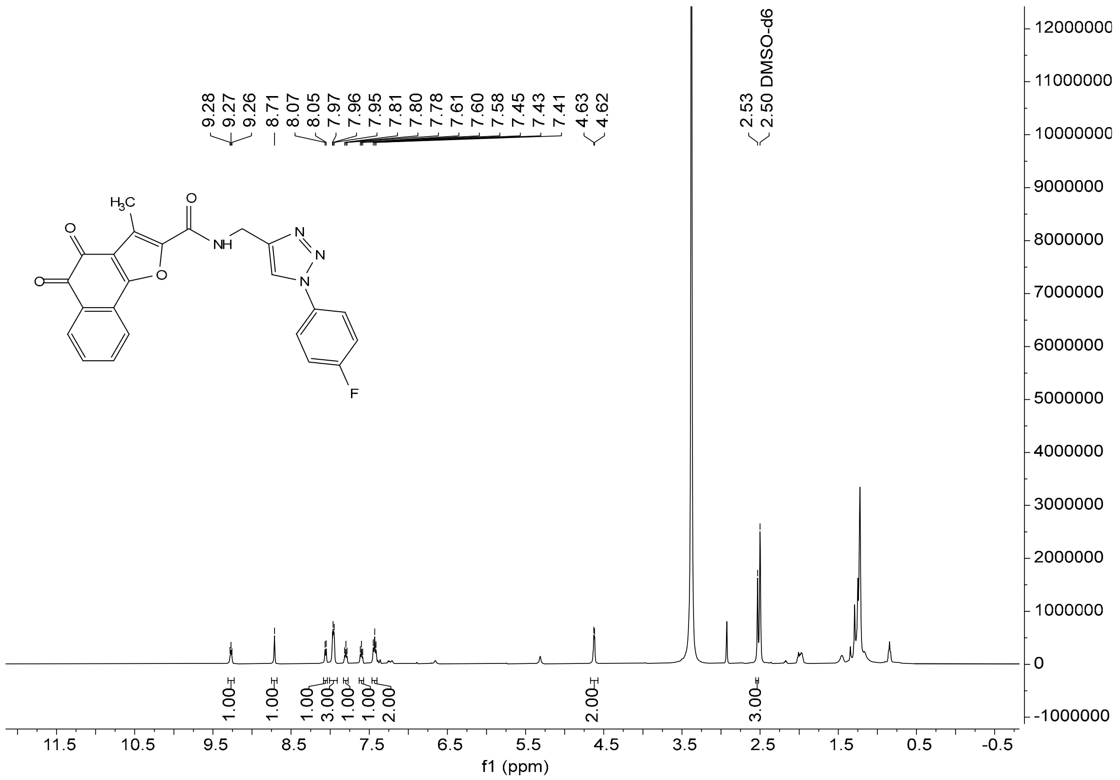

Figure S71. ^{1}H NMR spectrum (500 MHz, DMSO-*d*6) of compound **S2-11**.

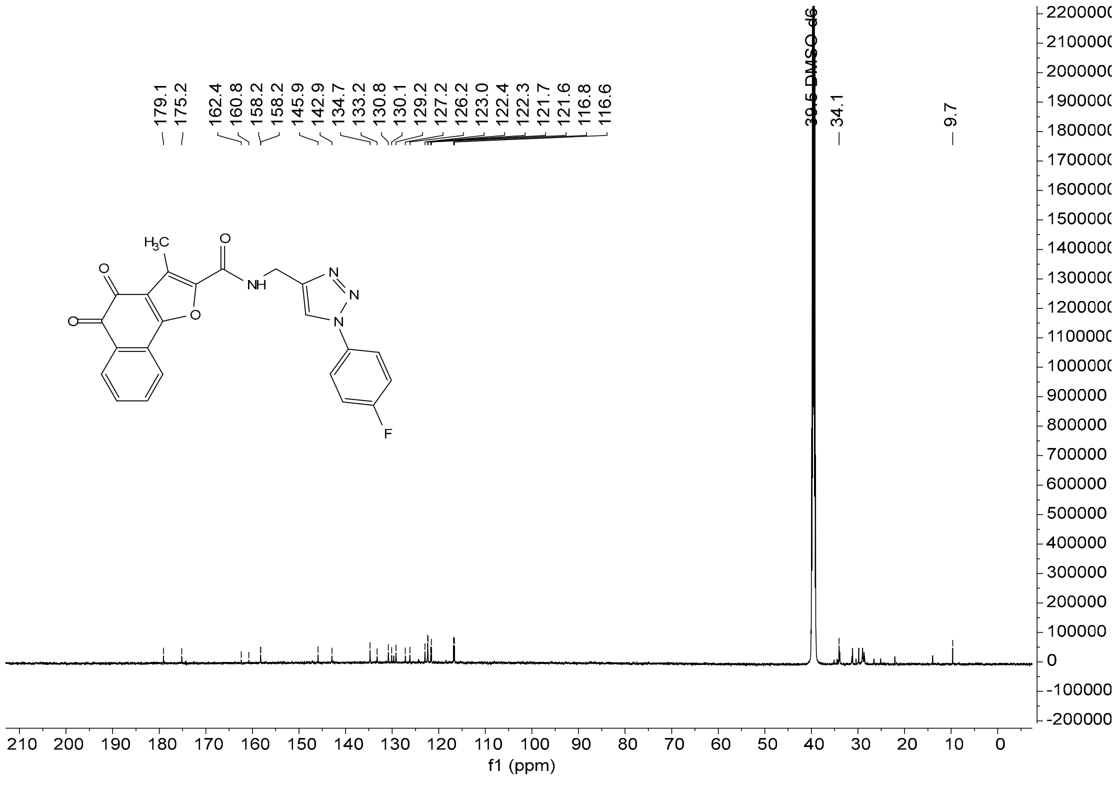

Figure S72. ^{13}C NMR spectrum (500 MHz, DMSO-*d*6) of compound **S2-11**.

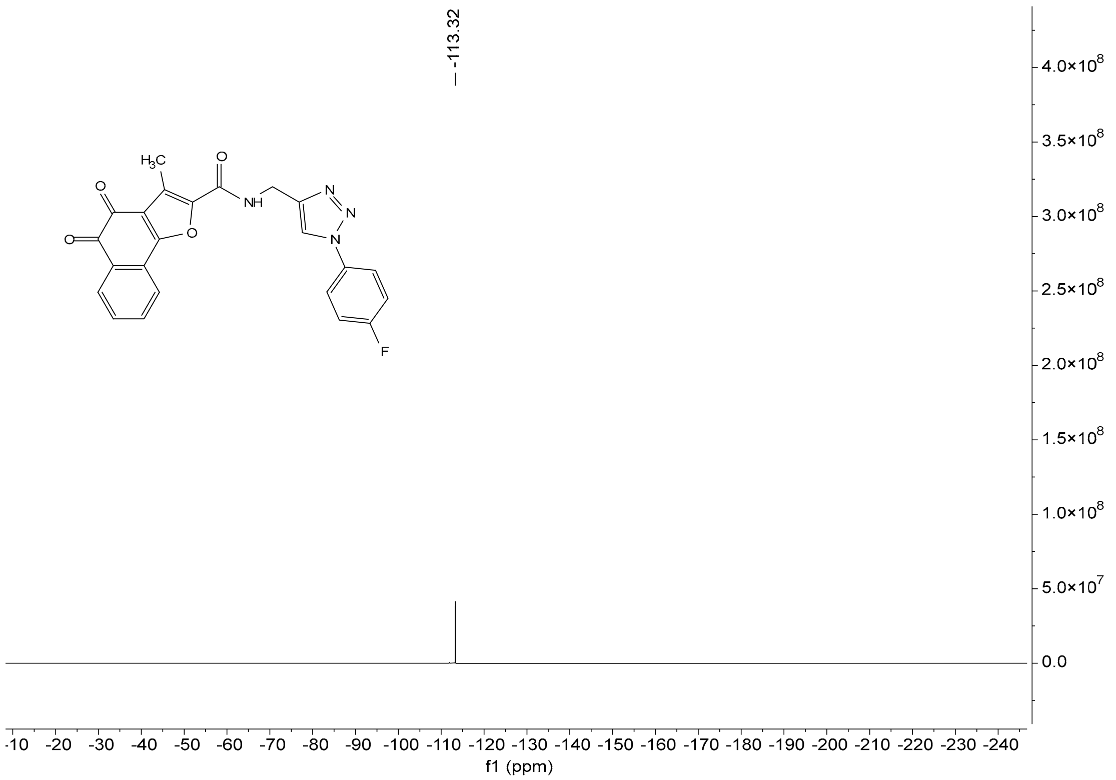

Figure S73. ^{19}F NMR spectrum (500 MHz, DMSO-d_6) of compound **S2-11**.

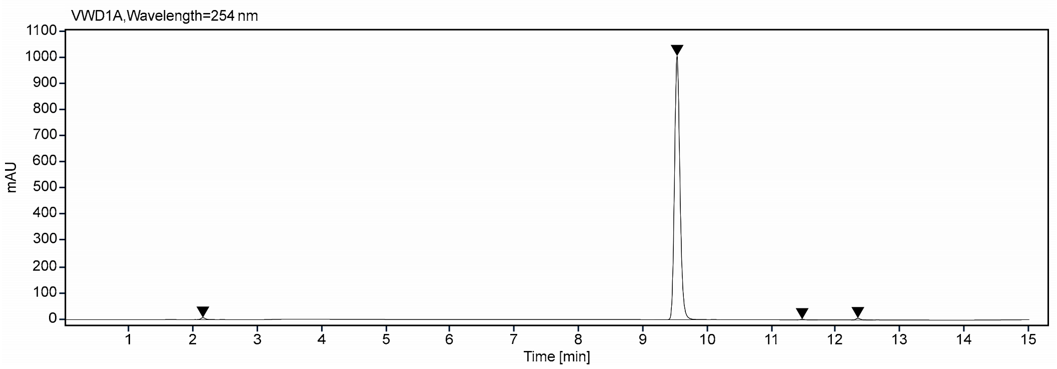

Signal: VWD1A,Wavelength=254 nm

RT [min]	Width [min]	Area	Height	Area%
2.143	0.45	39.47	7.59	0.66
9.522	0.85	5915.36	1005.06	98.70
11.469	0.33	7.97	1.73	0.13
12.337	0.33	30.22	6.46	0.50
	**Sum**	**5993.02**		

Figure S74. HPLC spectrum of compound **S2-11**.

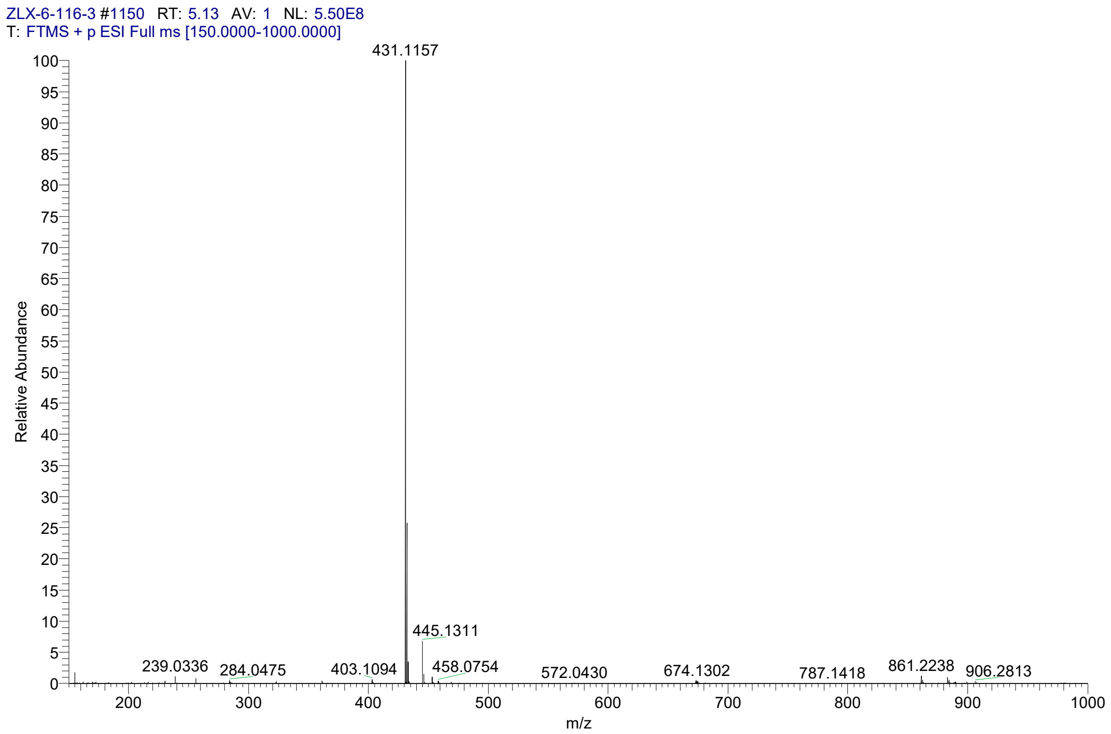

Figure S75. HRMS of compound **S2-11**.

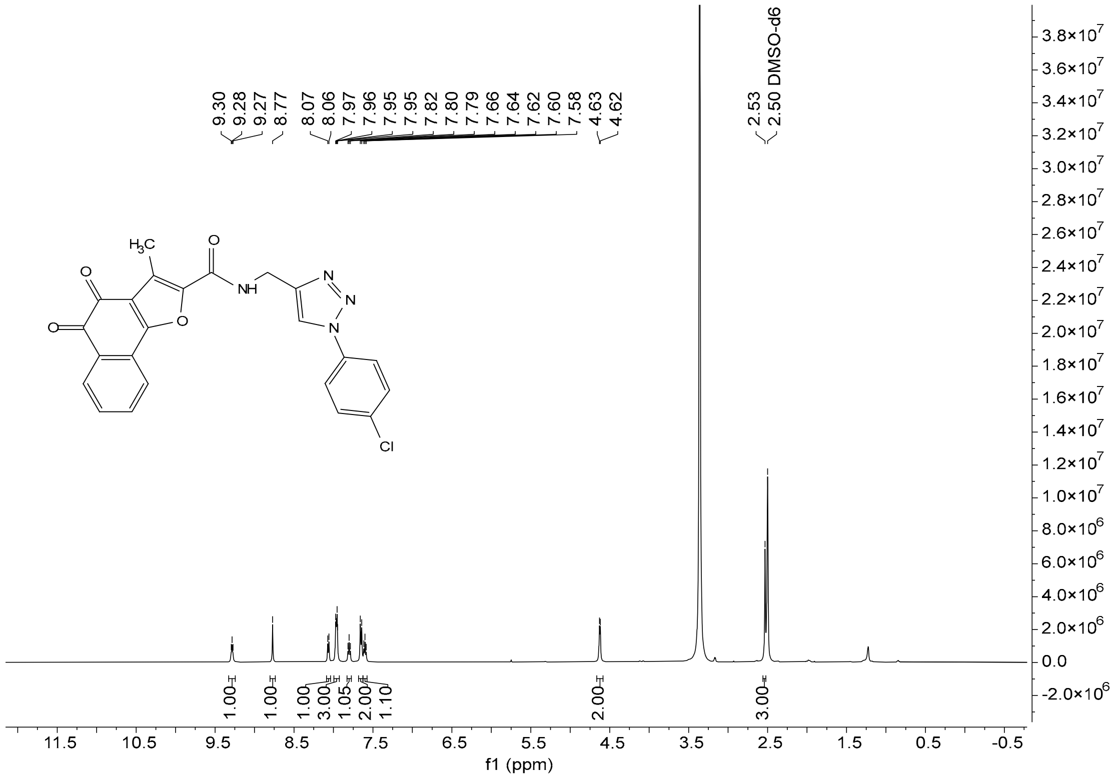

Figure S76. ^{1}H NMR spectrum (500 MHz, DMSO-d_6) of compound **S2-12**.

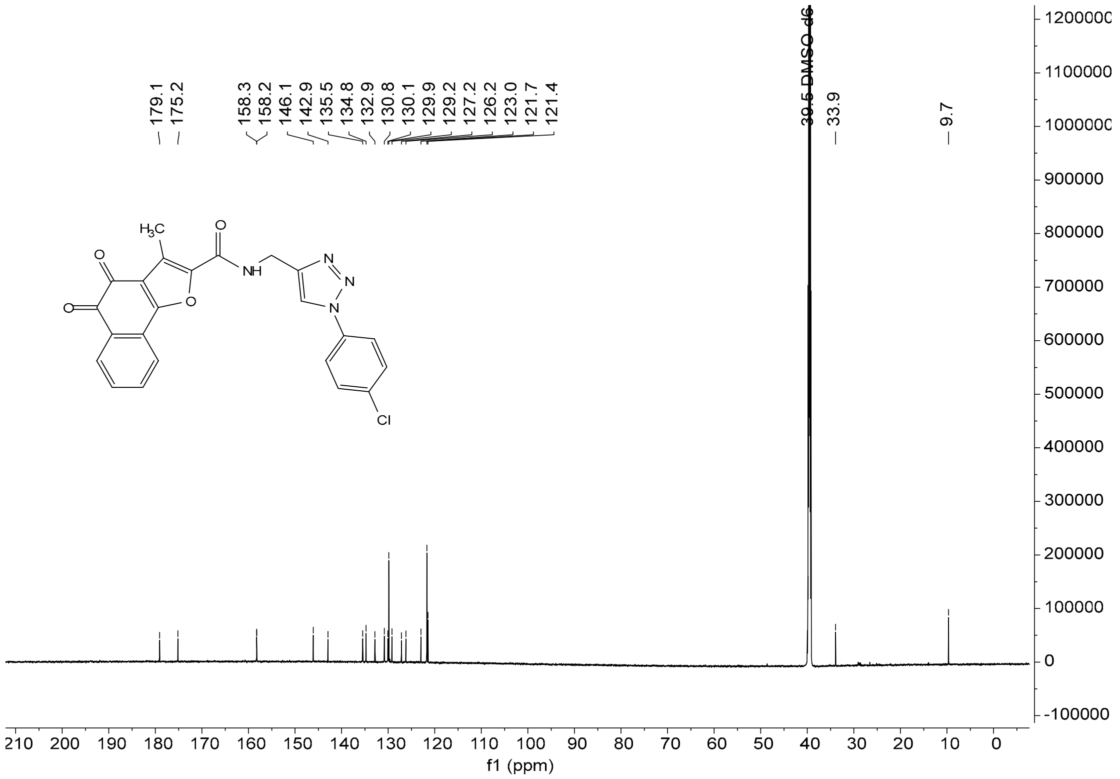

Figure S77. ^{13}C NMR spectrum (500 MHz, DMSO-d_6) of compound **S2-12**.

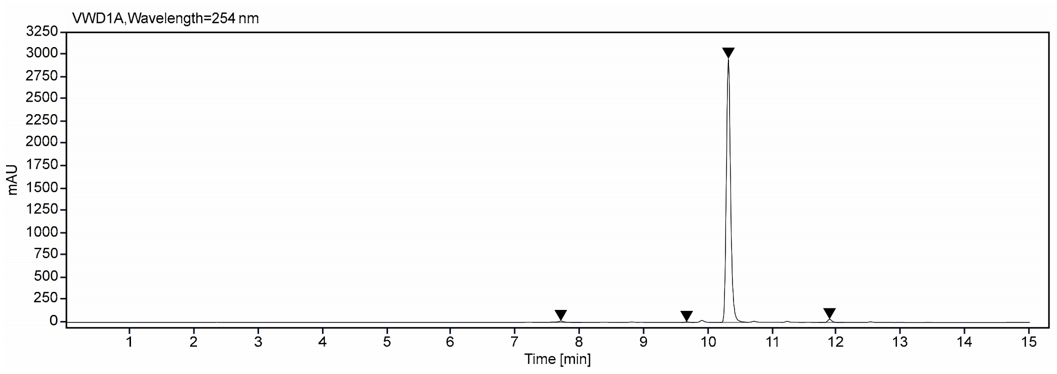

Signal: VWD1A,Wavelength=254 nm

RT [min]	Width [min]	Area	Height	Area%
7.700	0.77	110.65	13.91	0.79
9.660	0.20	19.72	4.45	0.14
10.309	0.44	13638.89	2955.63	97.93
11.885	0.39	157.64	35.48	1.13
	**Sum**	**13926.90**		

Figure S78. HPLC spectrum of compound **S2-12**.

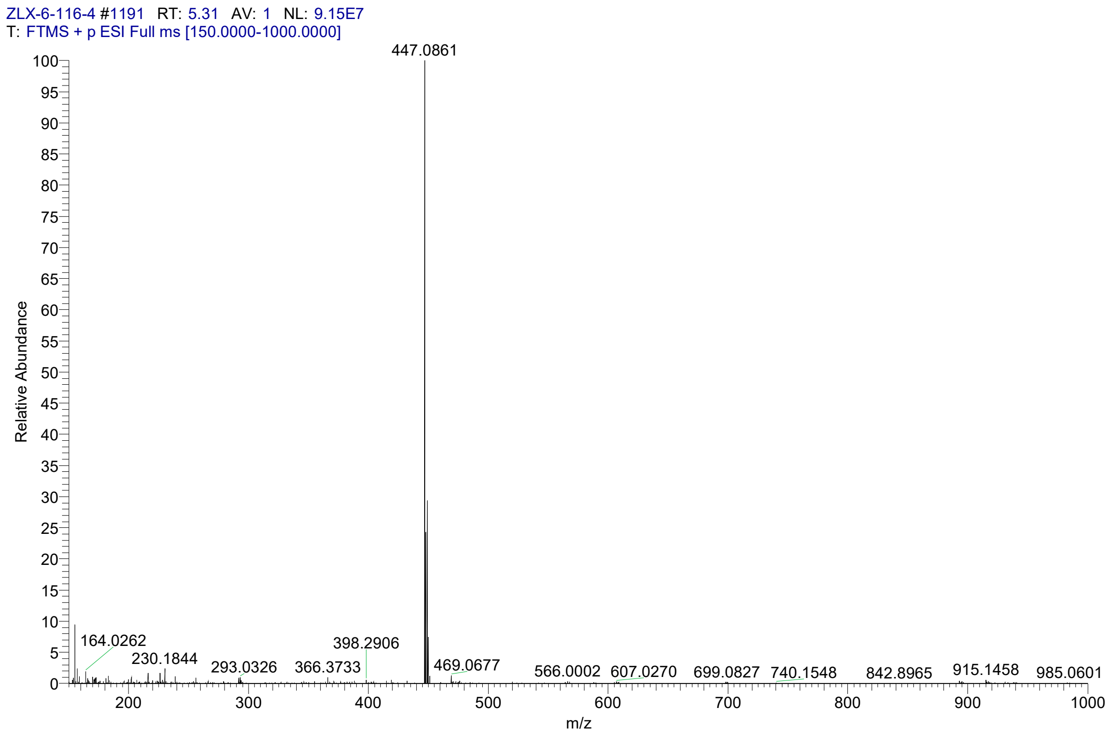

Figure S79. HRMS of compound **S2-12**.

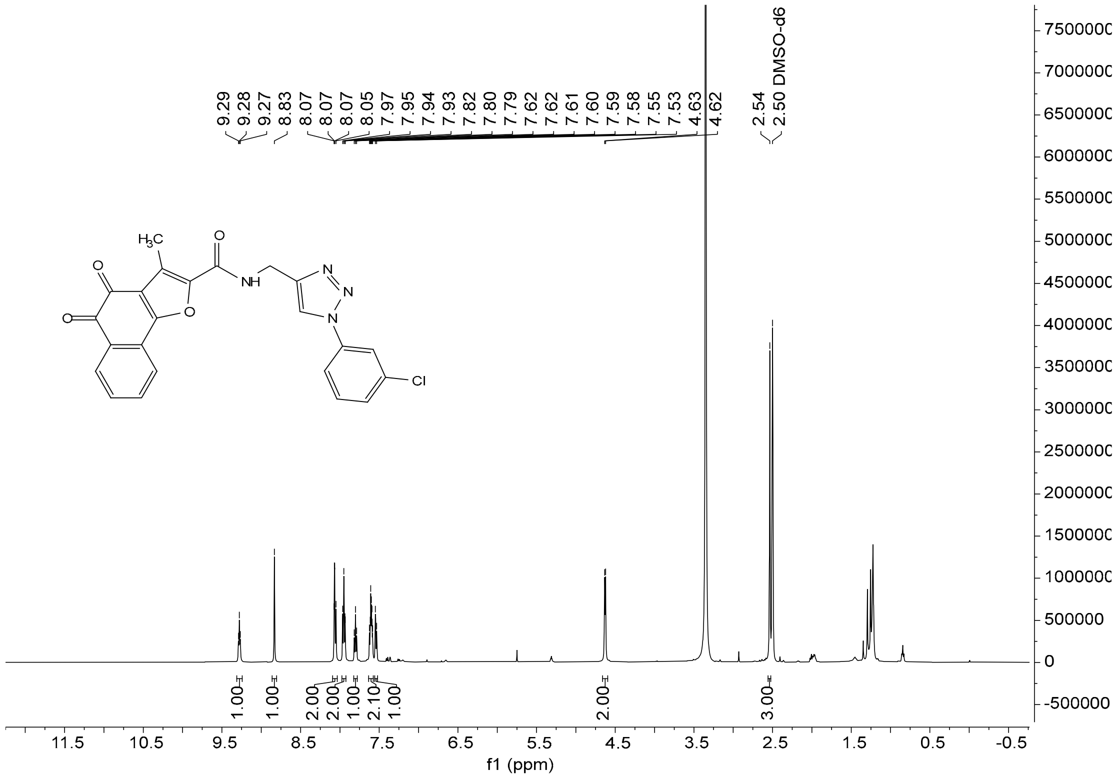

Figure S80. ^{1}H NMR spectrum (500 MHz, DMSO-d_6) of compound **S2-13**.

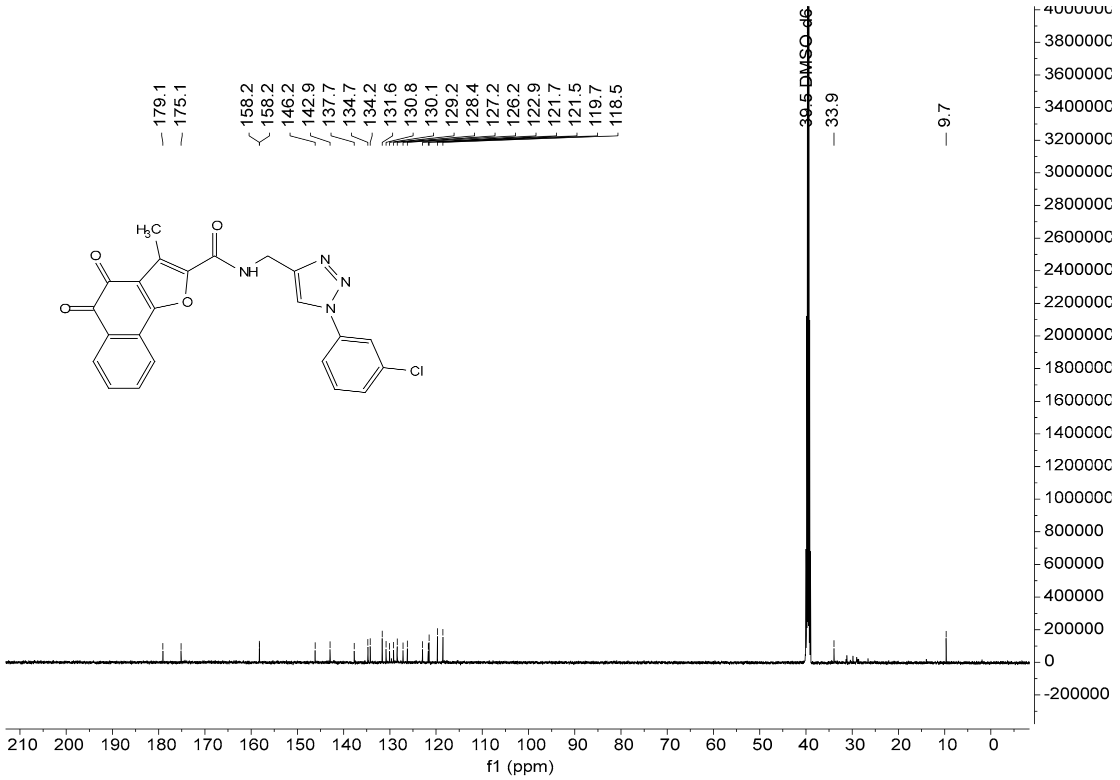

Figure S81. ^{13}C NMR spectrum (500 MHz, DMSO-d_6) of compound **S2-13**.

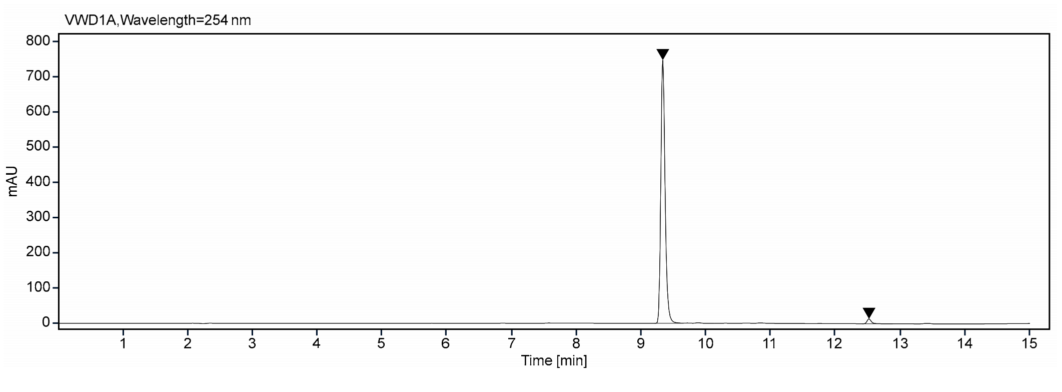

Signal: VWD1A,Wavelength=254 nm

RT [min]	Width [min]	Area	Height	Area%
9.328	0.45	3445.40	747.51	98.13
12.511	0.47	65.80	13.69	1.87
	**Sum**	**3511.20**		

Figure S82. HPLC spectrum of compound **S2-13**.

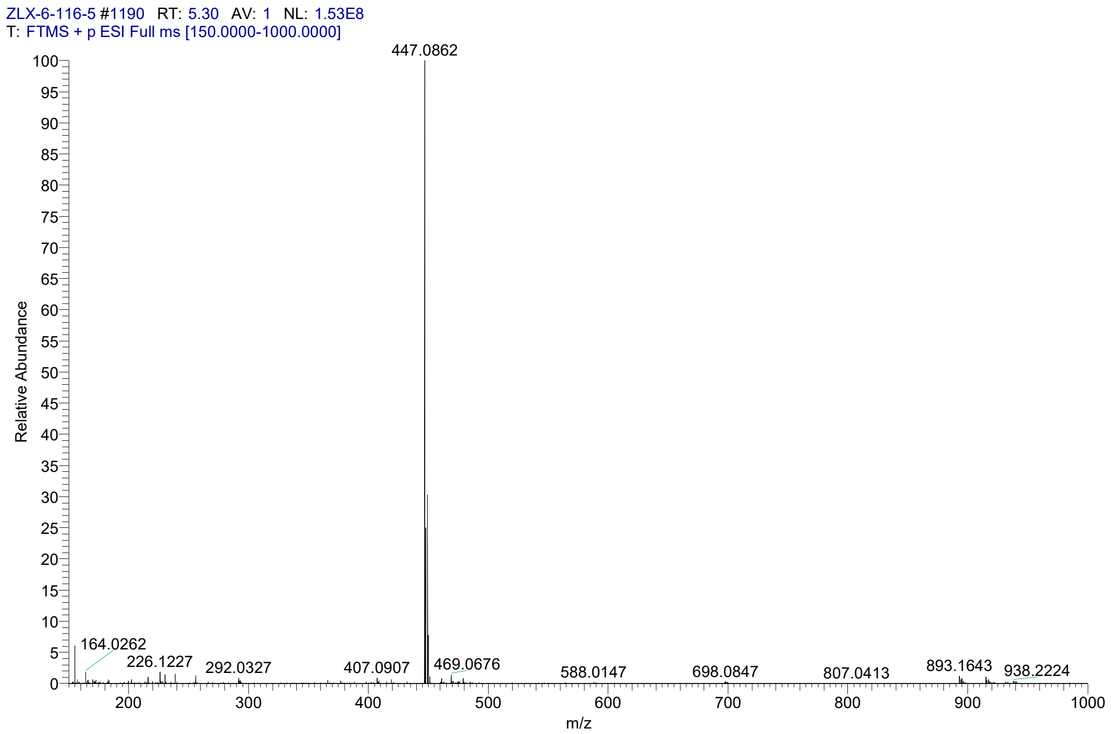

Figure S83. HRMS of compound **S2-13**.

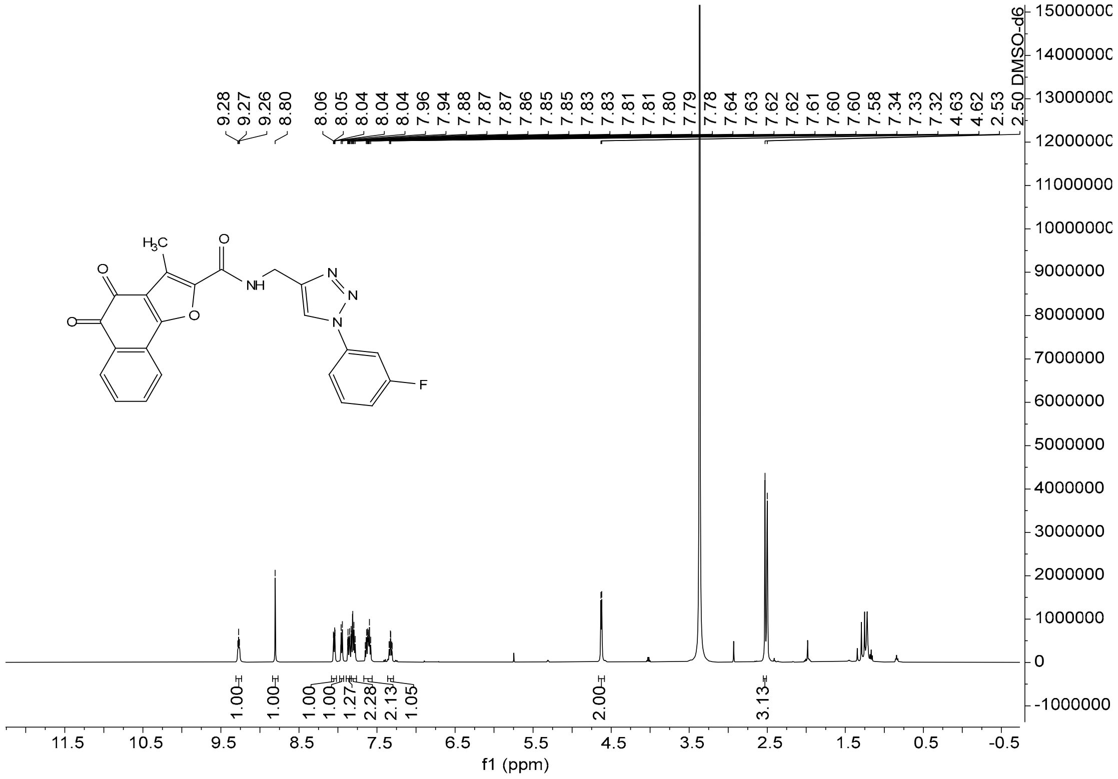

Figure S84. ^{1}H NMR spectrum (500 MHz, DMSO-*d*₆) of compound **S2-14**.

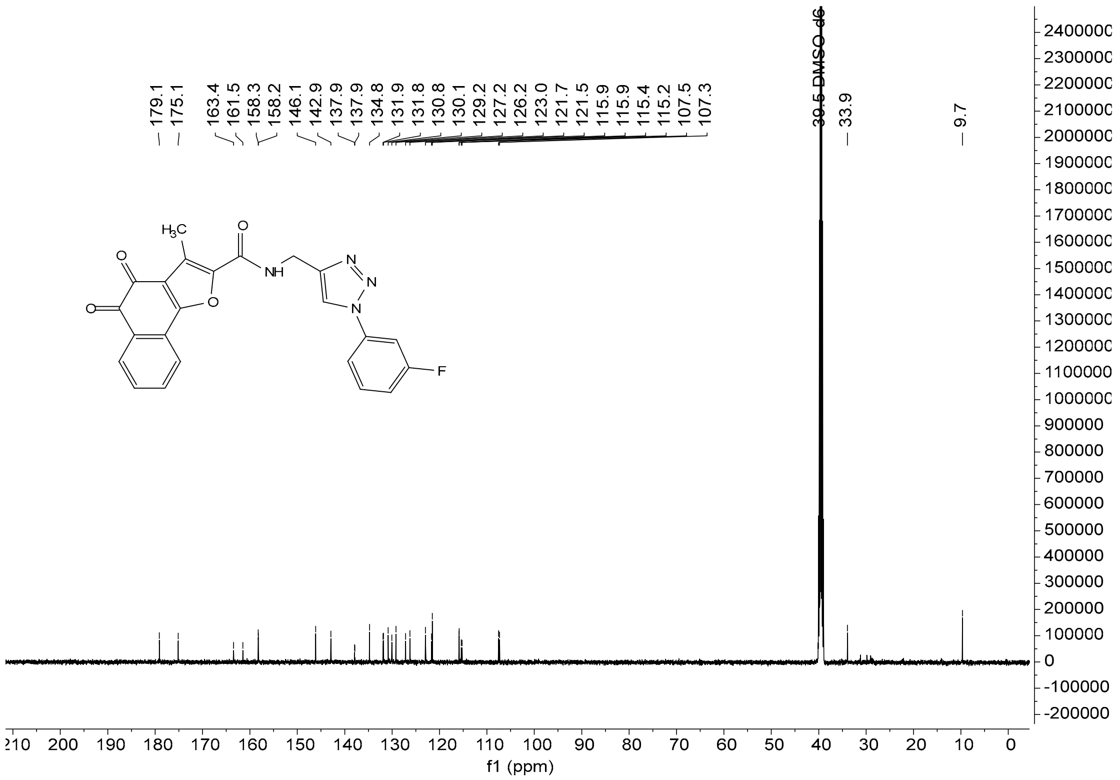

Figure S85. ^{13}C NMR spectrum (500 MHz, DMSO-*d*6) of compound **S2-14**.

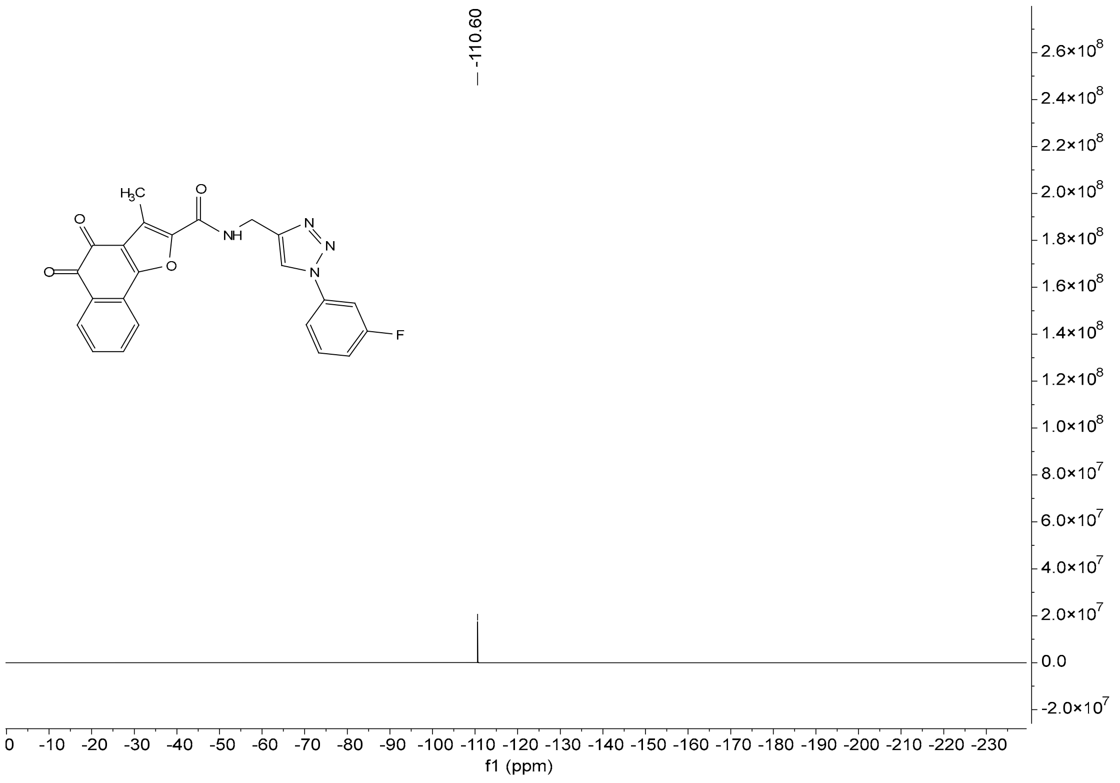

Figure S86. ^{19}F NMR spectrum (500 MHz, DMSO-*d*6) of compound **S2-14**.

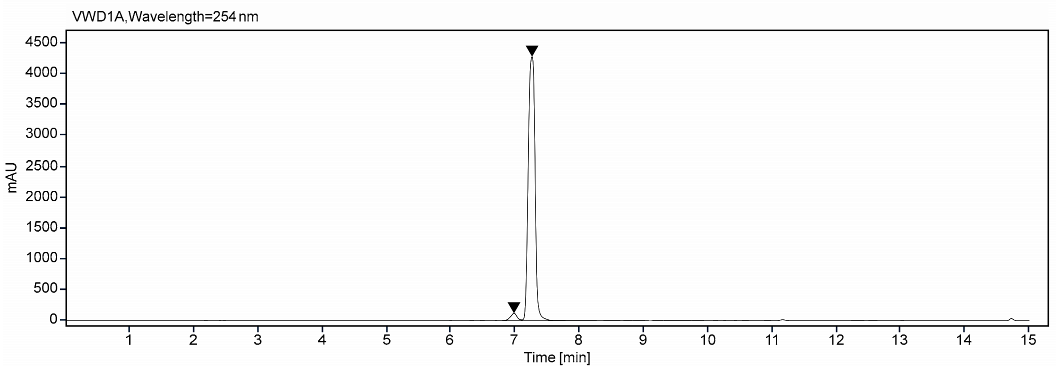

Signal: VWD1A,Wavelength=254 nm

RT [min]	Width [min]	Area	Height	Area%
6.976	0.34	841.84	116.80	2.62
7.256	0.68	31275.68	4286.79	97.38
	**Sum**	**32117.52**		

Figure S87. HPLC spectrum of compound **S2-14**.

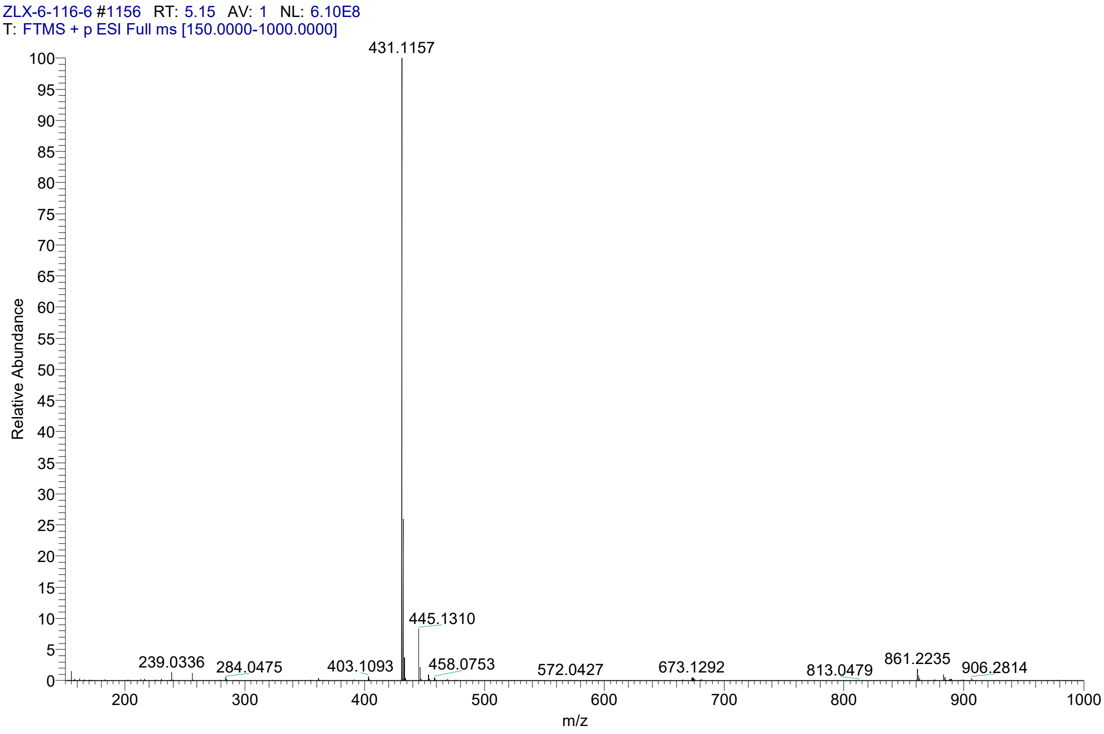

Figure S88. HRMS of compound **S2-14**.

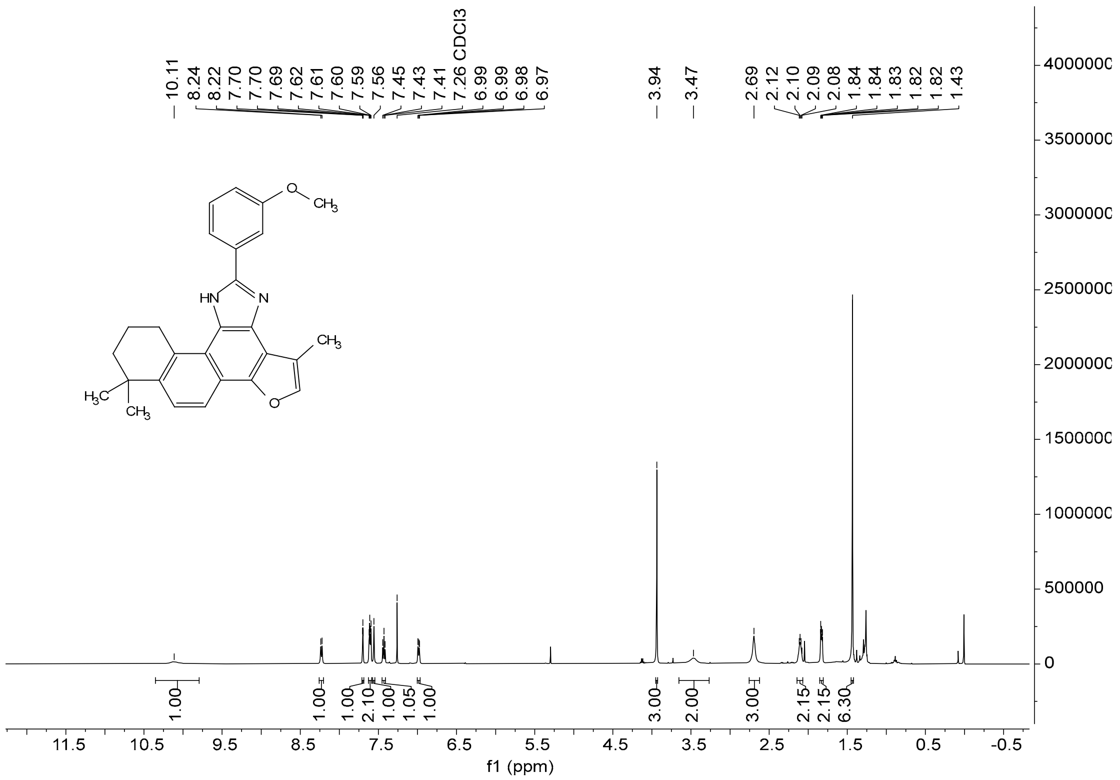

Figure S89. ^{1}H NMR spectrum (500 MHz, Chloroform-*d*) of compound **S3-1**.

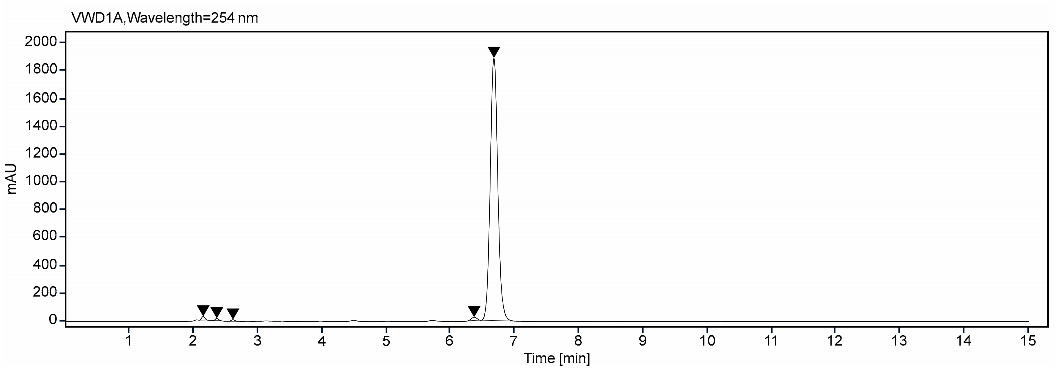

Signal: VWD1A,Wavelength=254 nm

RT [min]	Width [min]	Area	Height	Area%
2.143	0.12	92.74	26.46	0.59
2.356	0.11	55.01	16.37	0.35
2.606	0.14	43.64	11.39	0.28
6.363	0.18	135.25	23.27	0.86
6.670	0.54	15469.76	1888.35	97.93
	**Sum**	**15796.41**		

Figure S90. HPLC spectrum of compound **S3-1**.

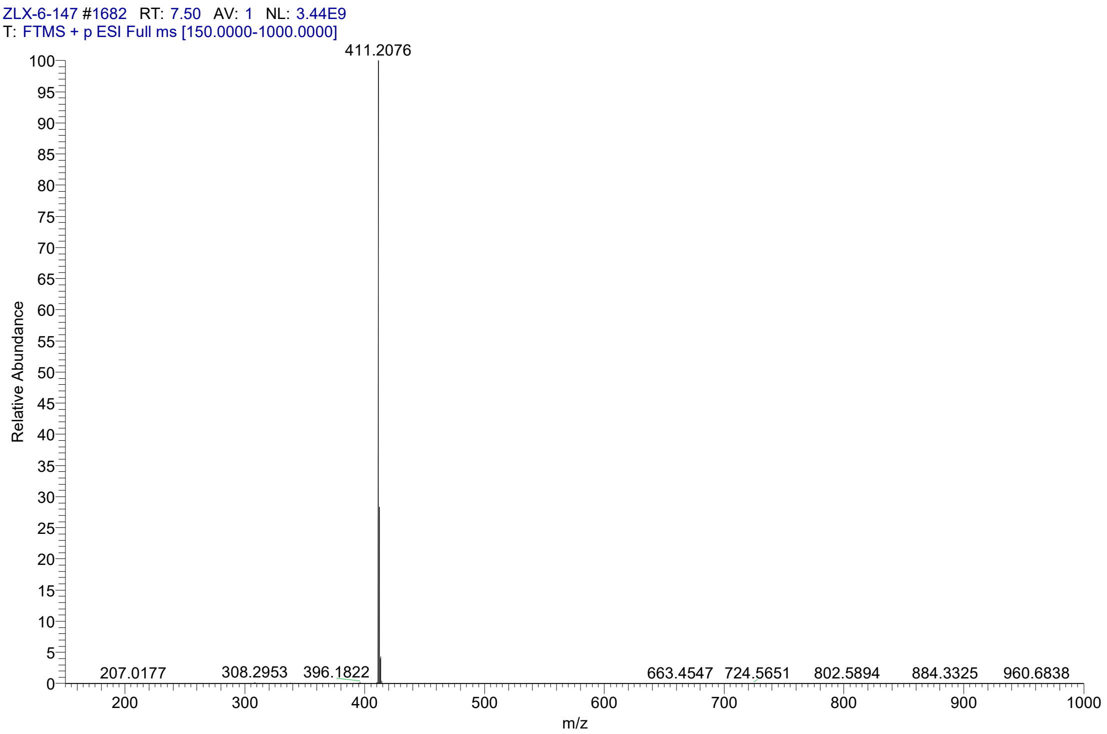

Figure S91. HRMS of compound **S3-1**.

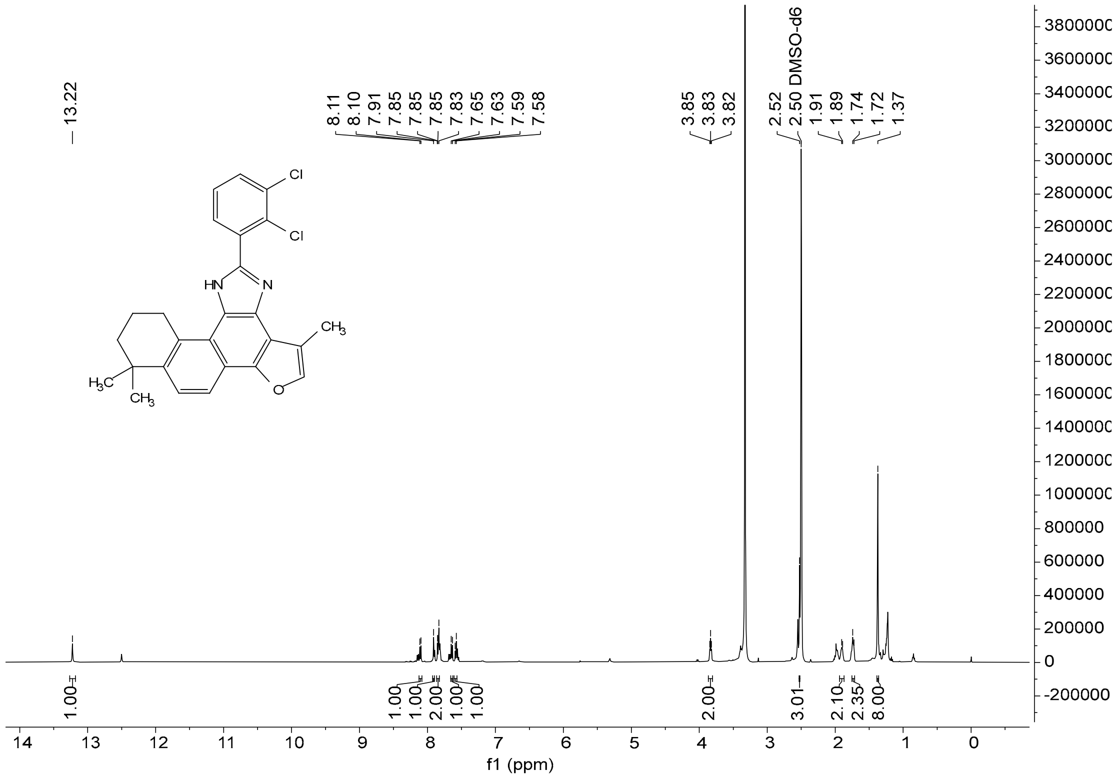

Figure S92. ^{1}H NMR spectrum (500 MHz, DMSO-d_6) of compound **S3-2**.

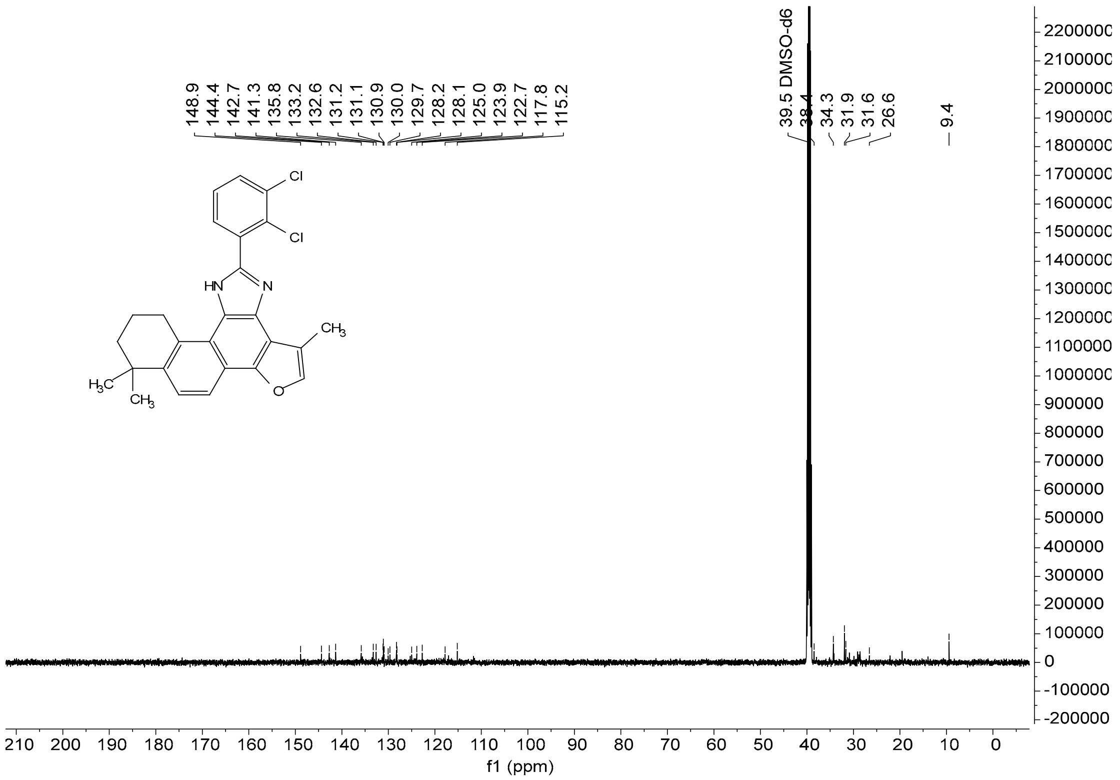

Figure S93. ^{13}C NMR spectrum (500 MHz, DMSO-d_6) of compound **S3-2**.

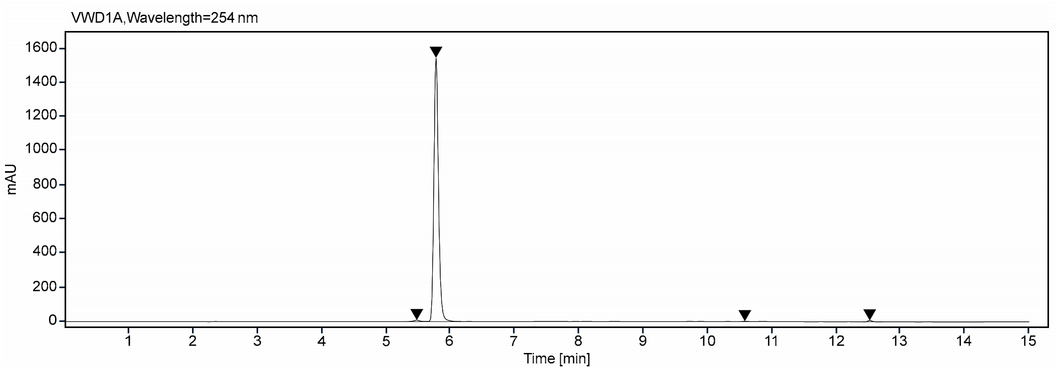

Signal: VWD1A,Wavelength=254 nm

RT [min]	Width [min]	Area	Height	Area%
5.469	0.36	58.35	8.53	0.75
5.770	0.56	7652.55	1545.38	98.62
10.574	0.26	12.43	2.11	0.16
12.518	0.31	36.62	8.07	0.47
	**Sum**	**7759.94**		

Figure S94. HPLC spectrum of compound **S3-2**.

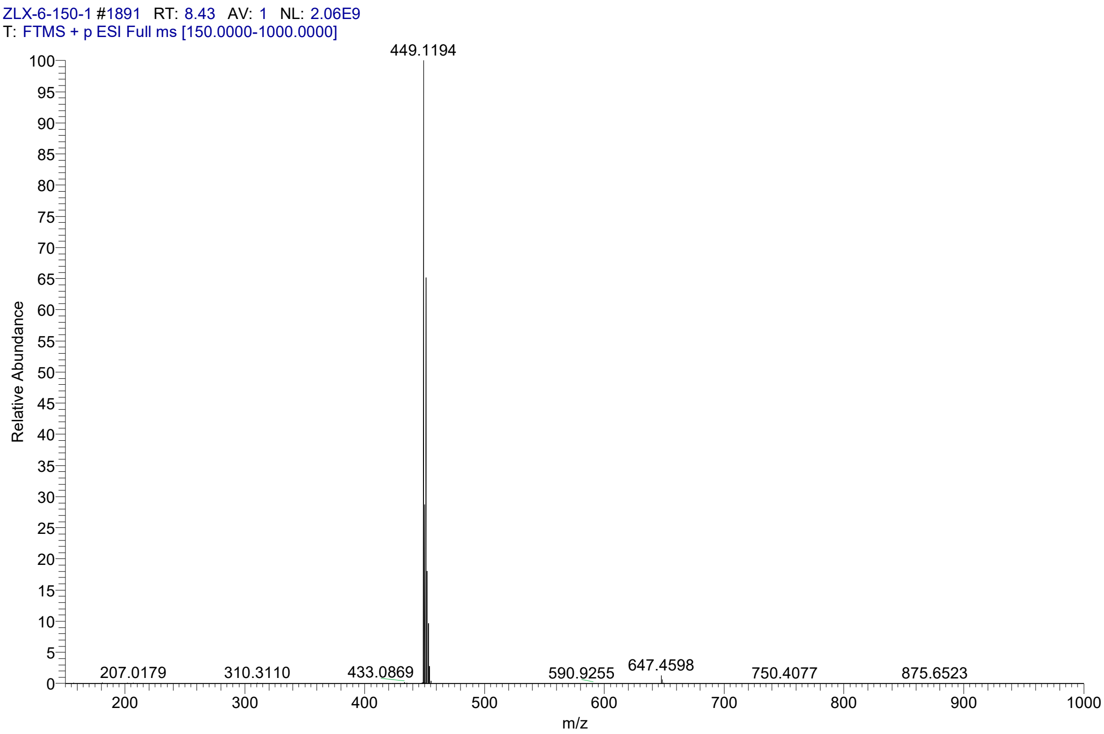

Figure S95. HRMS of compound **S3-2**.

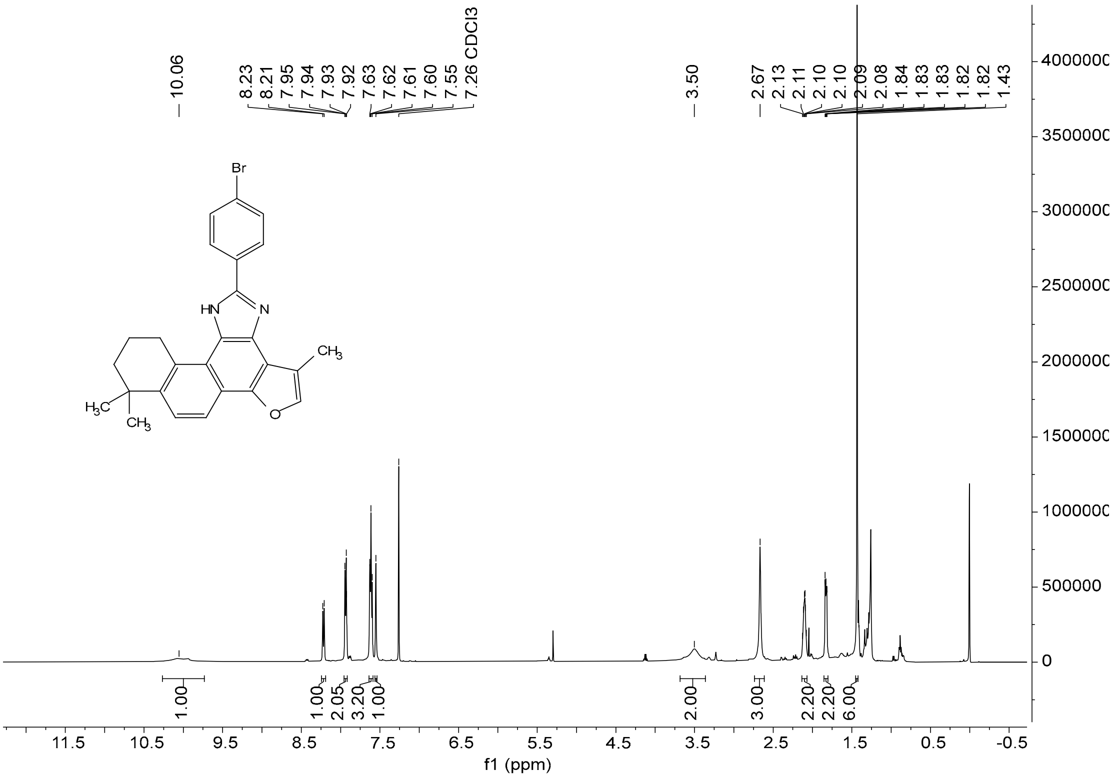

Figure S96. ^{1}H NMR spectrum (500 MHz, Chloroform-*d*) of compound **S3-3**.

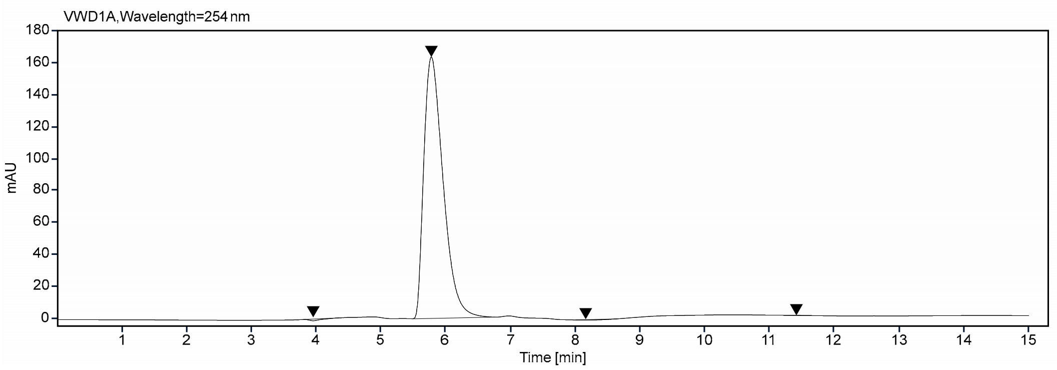

Signal: VWD1A,Wavelength=254 nm

RT [min]	Width [min]	Area	Height	Area%
3.947	0.65	13.02	1.06	0.38
5.772	1.29	3382.06	163.15	99.37
8.156	0.64	8.00	0.21	0.24
11.410	0.45	0.45	0.03	0.01
	**Sum**	**3403.53**		

Figure S97. HPLC spectrum of compound **S3-3**.

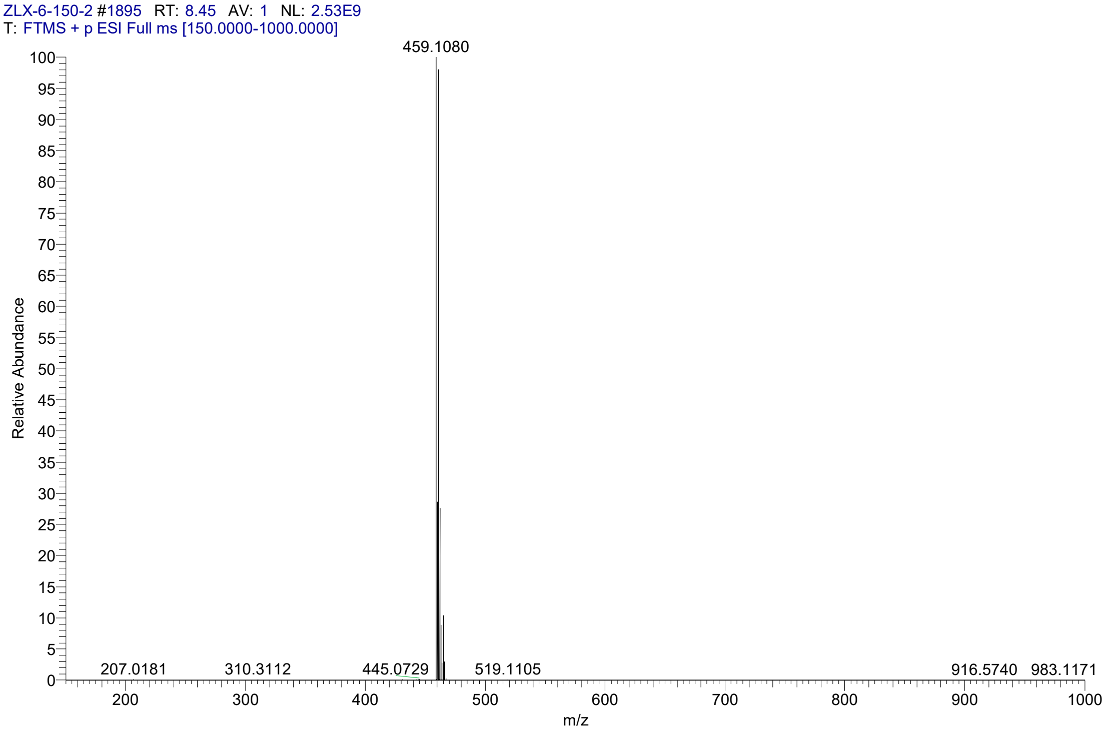

Figure S98. HRMS of compound **S3-3**.

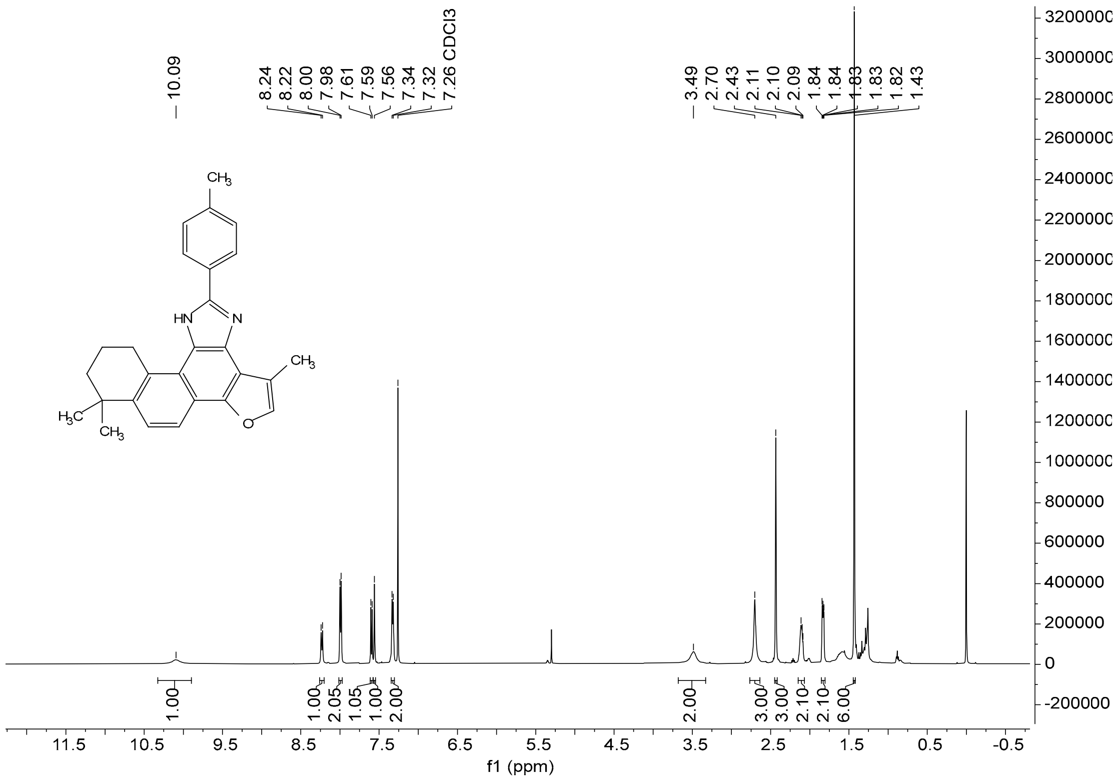

Figure S99. ^{1}H NMR spectrum (500 MHz, Chloroform-*d*) of compound **S3-4**.

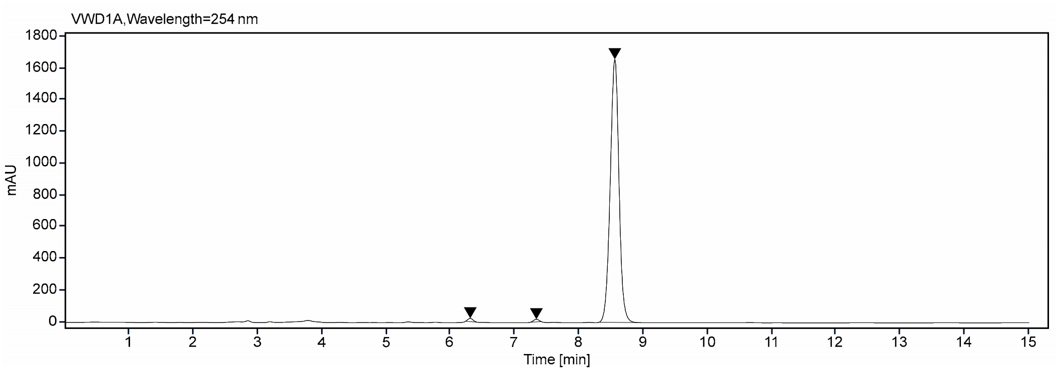

Signal: VWD1A,Wavelength=254 nm

RT [min]	Width [min]	Area	Height	Area%
6.302	0.19	115.74	20.58	0.73
7.332	0.17	92.88	16.34	0.59
8.552	0.59	15660.68	1654.86	98.69
	**Sum**	**15869.30**		

Figure S100. HPLC spectrum of compound **S3-4**.

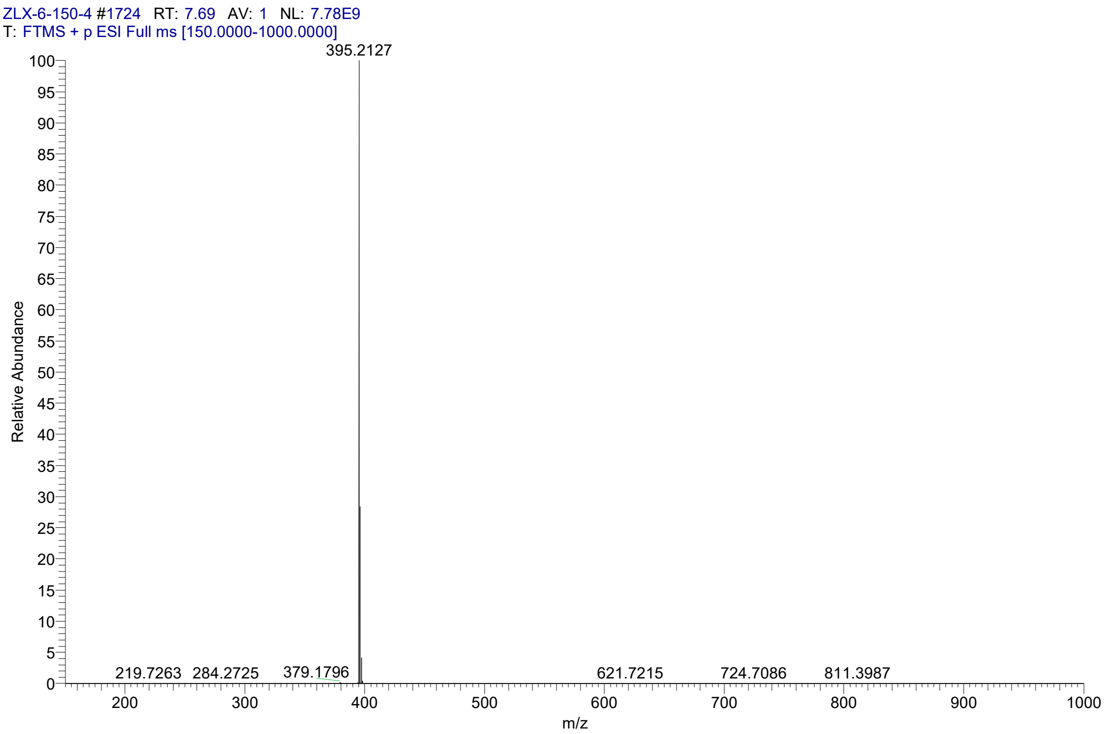

Figure S101. HRMS of compound **S3-4**.

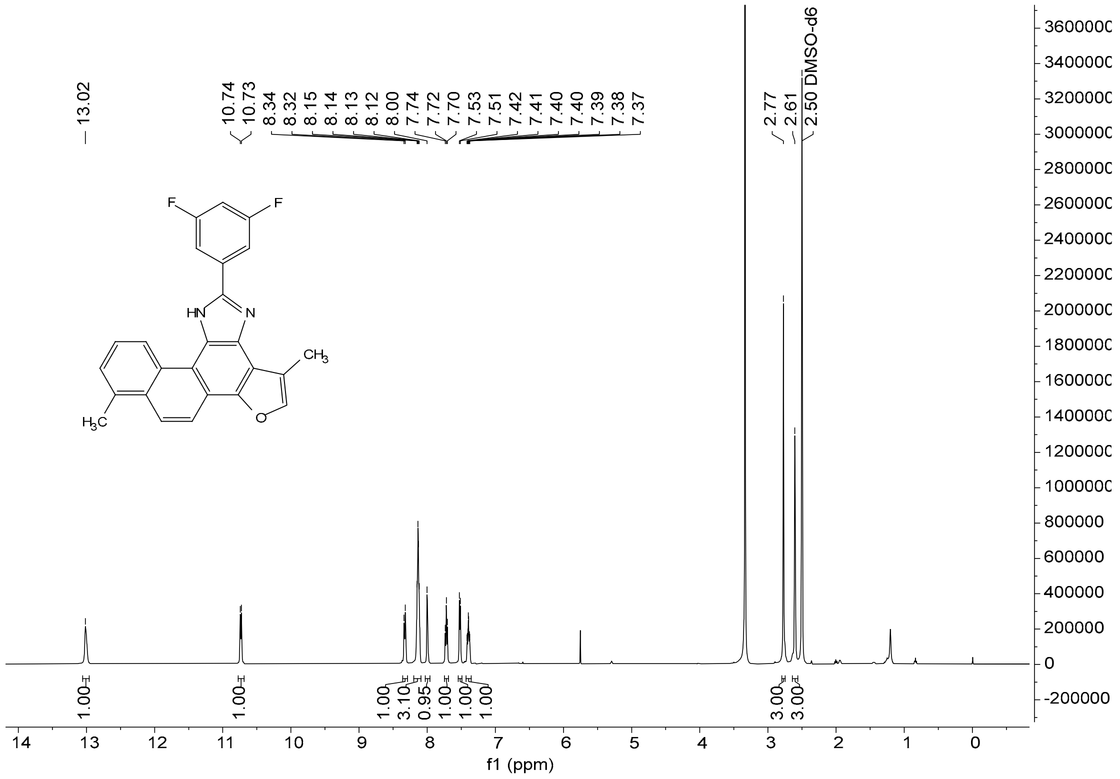

Figure S102. ^{1}H NMR spectrum (500 MHz, DMSO-d_6) of compound **S3-5**.

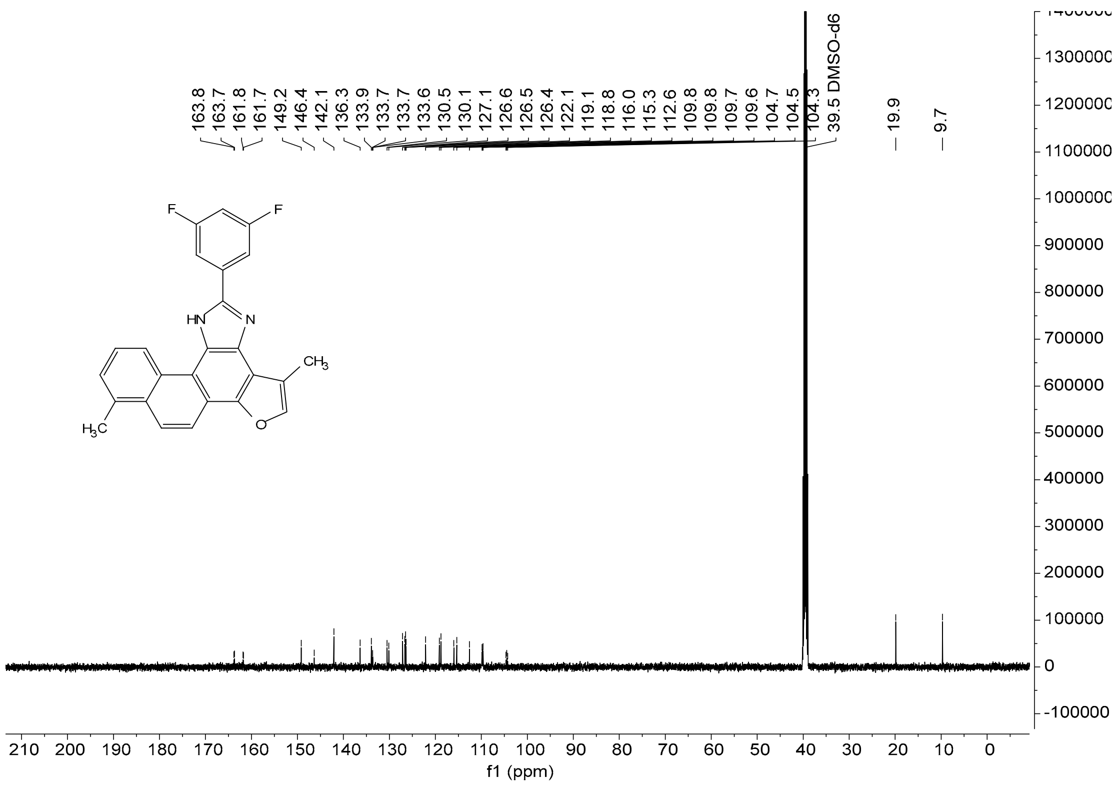

Figure S103. ^{13}C NMR spectrum (500 MHz, DMSO-d_6) of compound **S3-5**.

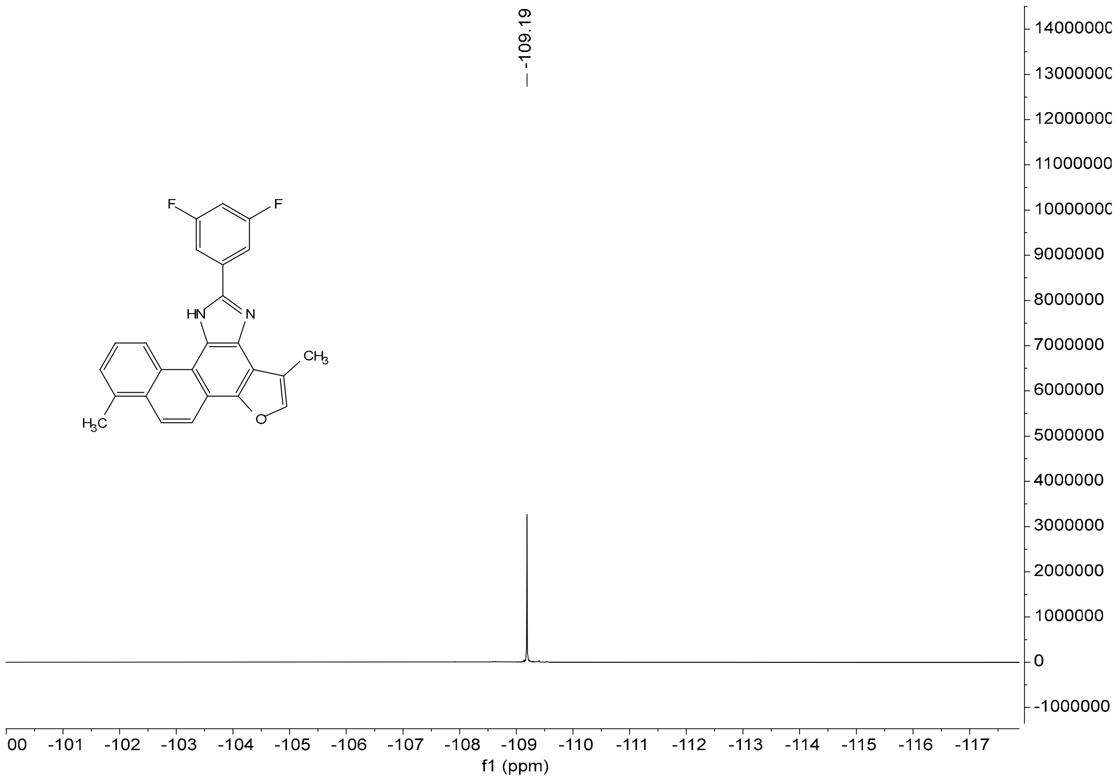

Figure S104. ^{19}F NMR spectrum (500 MHz, DMSO-d_6) of compound **S3-5**.

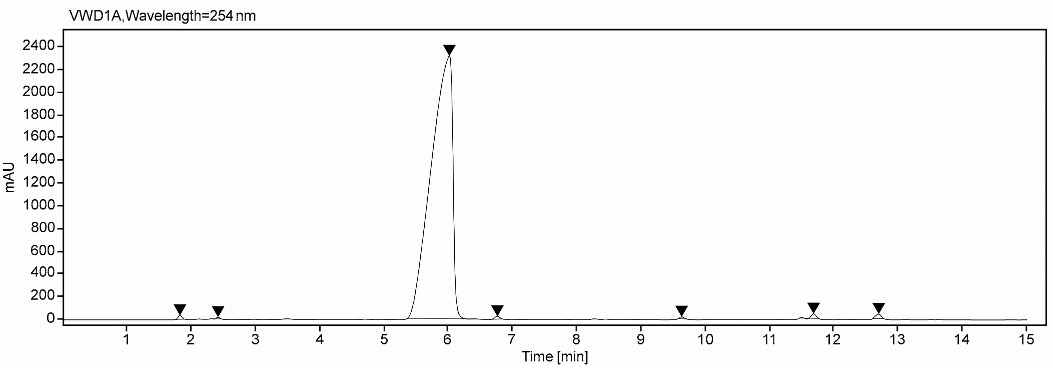

Signal: VWD1A,Wavelength=254 nm

RT [min]	Width [min]	Area	Height	Area%
1.814	0.19	143.93	34.51	0.26
2.408	0.13	41.97	13.18	0.08
6.008	1.05	53735.73	2312.86	98.49
6.755	0.20	125.59	23.94	0.23
9.624	0.13	68.59	15.99	0.13
11.678	0.30	208.59	41.33	0.38
12.693	0.17	235.94	39.25	0.43
	**Sum**	**54560.33**		

Figure S105. HPLC spectrum of compound **S3-5**.

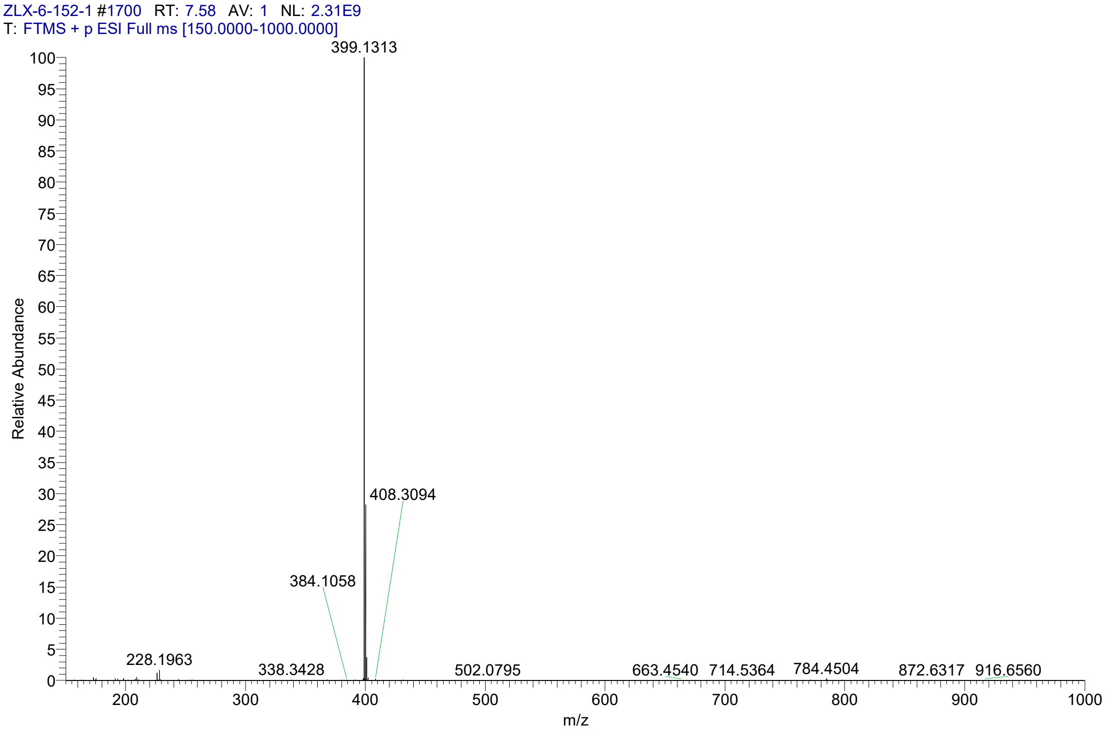

Figure S106. HRMS of compound **S3-5**.

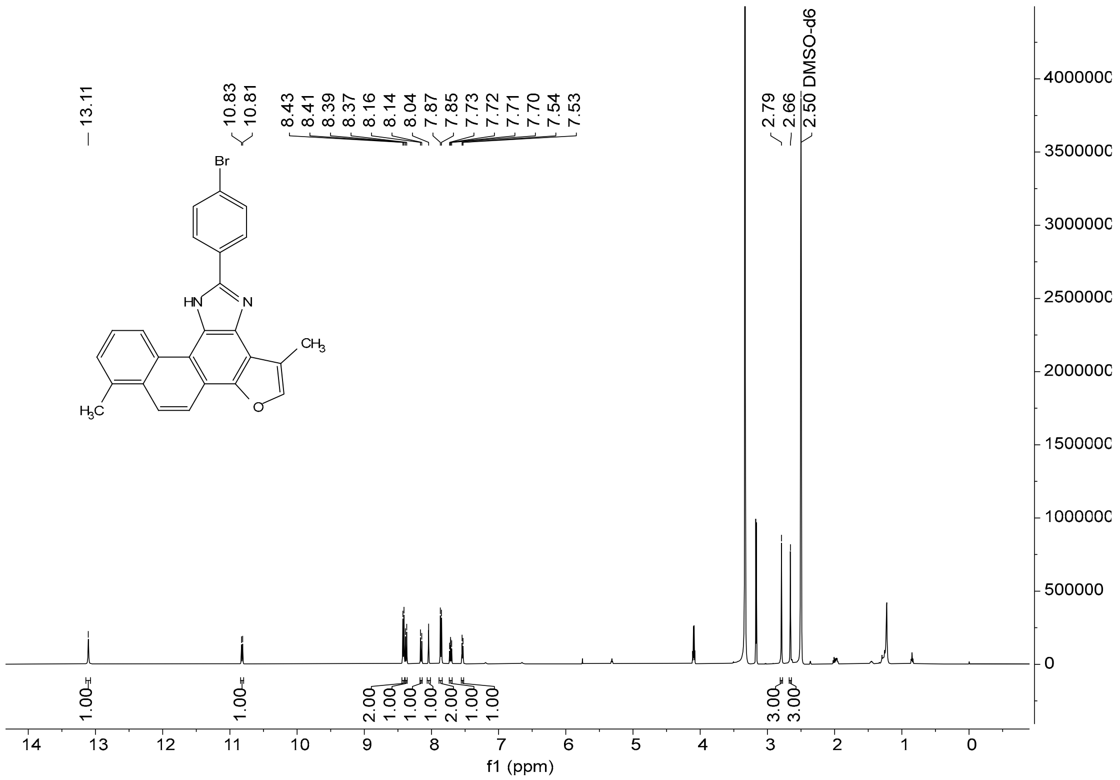

Figure S107. ^{1}H NMR spectrum (500 MHz, DMSO-d_6) of compound **S3-6**.

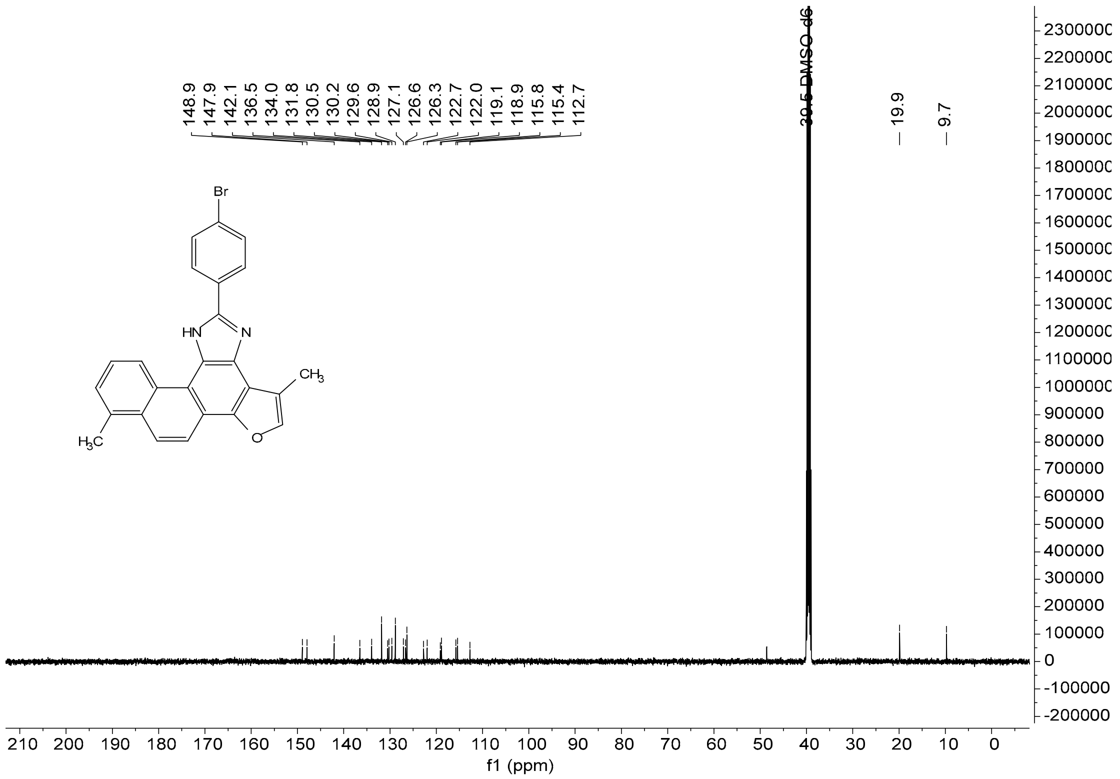

Figure S108. ^{13}C NMR spectrum (500 MHz, DMSO-d_6) of compound **S3-6**.

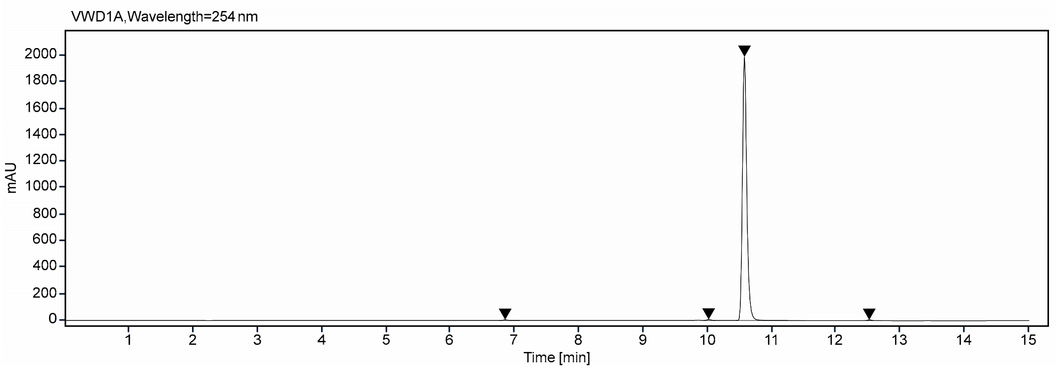

Signal: VWD1A,Wavelength=254 nm

RT [min]	Width [min]	Area	Height	Area%
6.847	0.33	23.16	5.17	0.24
10.013	0.18	31.84	7.31	0.33
10.570	1.08	9433.02	1993.42	99.13
12.514	0.29	28.17	6.25	0.30
	**Sum**	**9516.18**		

Figure S109. HPLC spectrum of compound **S3-6**.

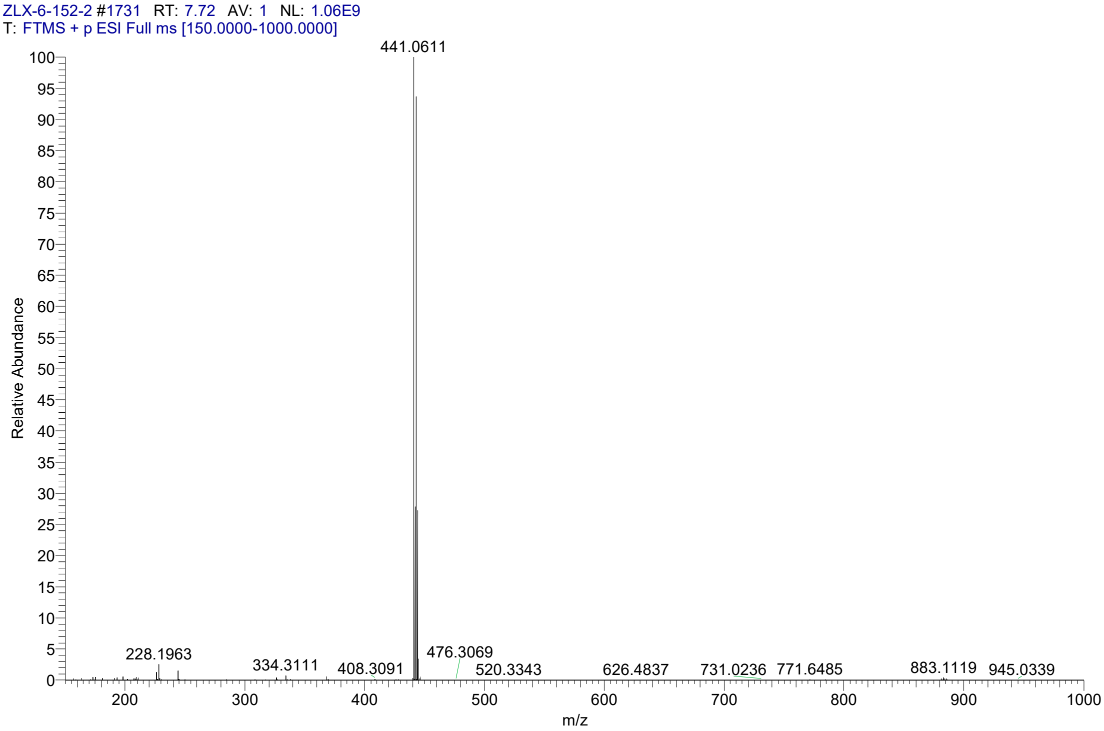

Figure S110. HRMS of compound **S3-6**.

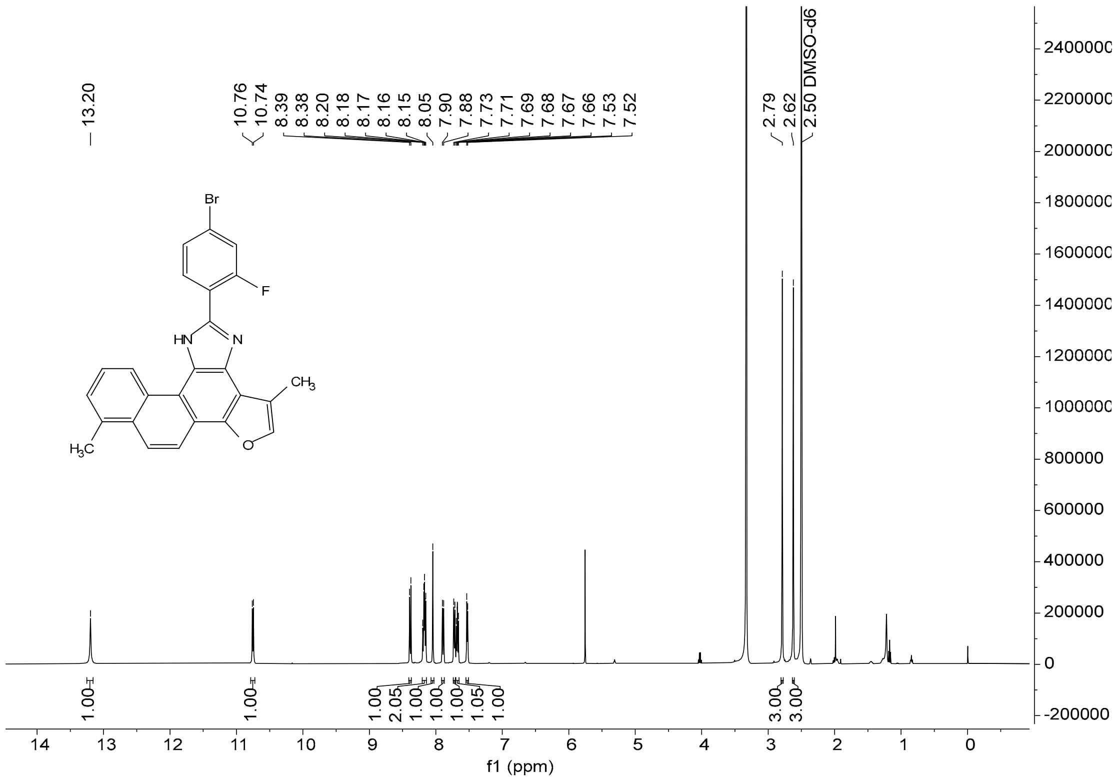

Figure S111. ^{1}H NMR spectrum (500 MHz, DMSO-d_6) of compound **S3-7**.

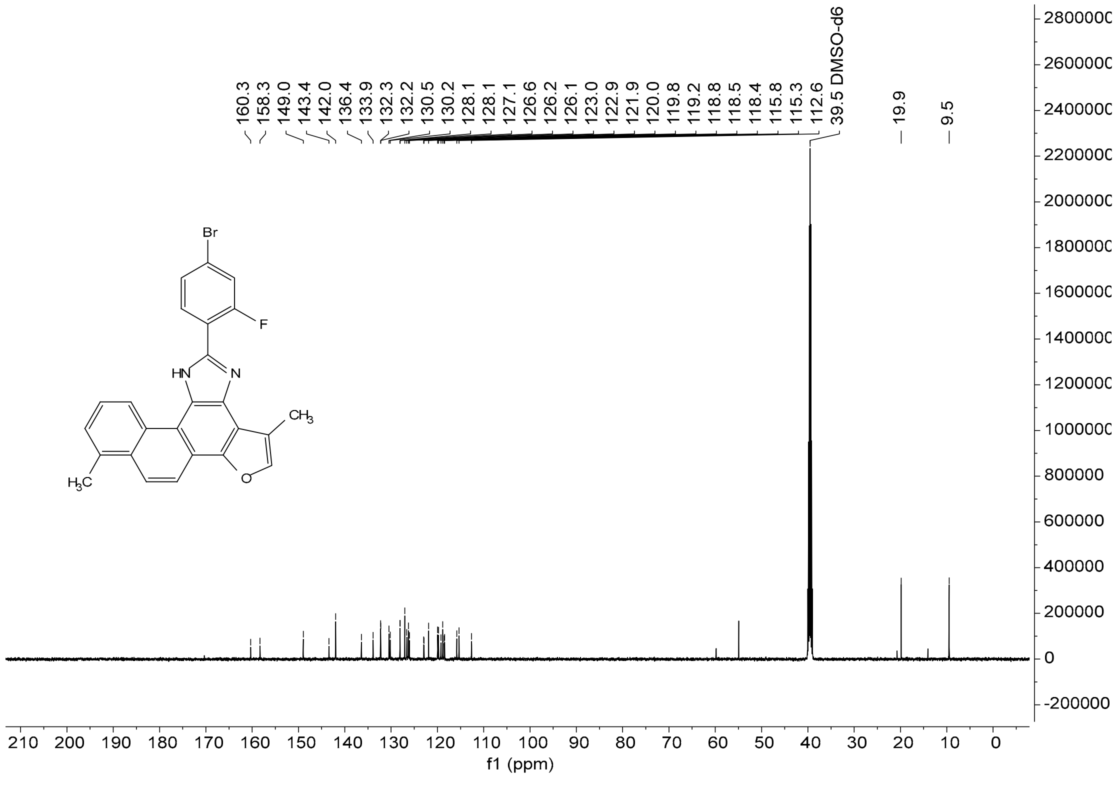

Figure S112. ^{13}C NMR spectrum (500 MHz, DMSO-d_6) of compound **S3-7**.

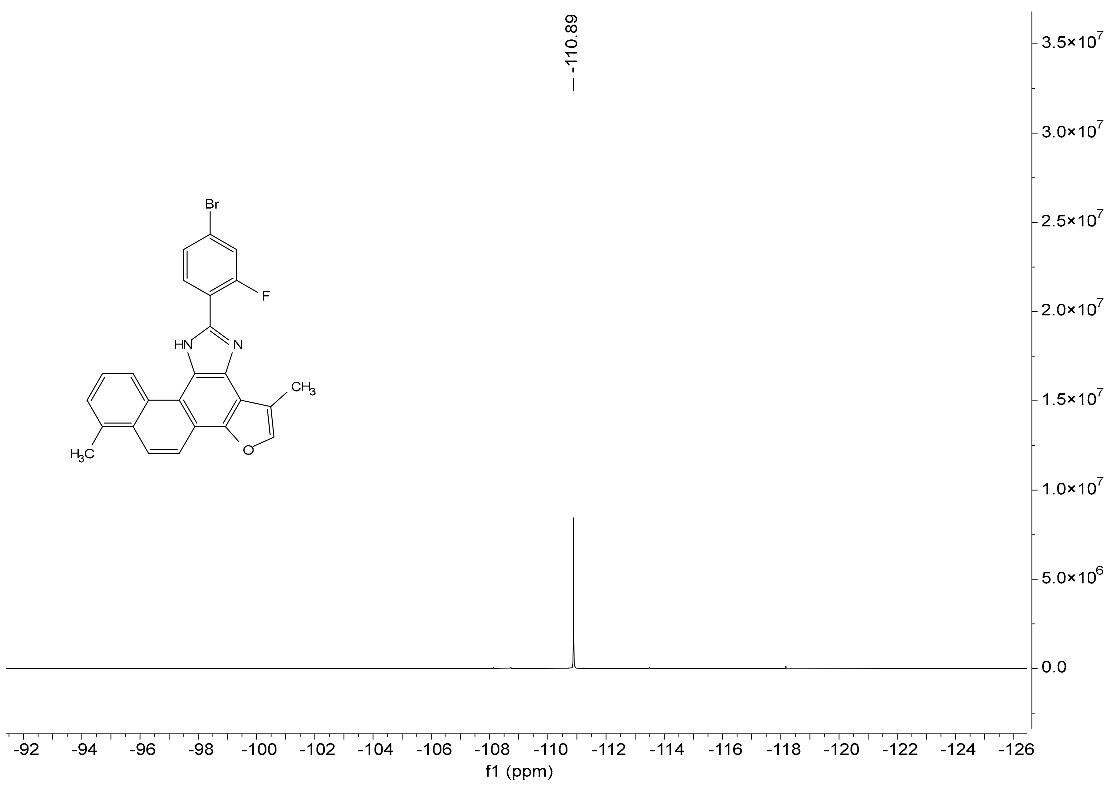

Figure S113. ^{19}F NMR spectrum (500 MHz, DMSO-d_6) of compound **S3-7**.

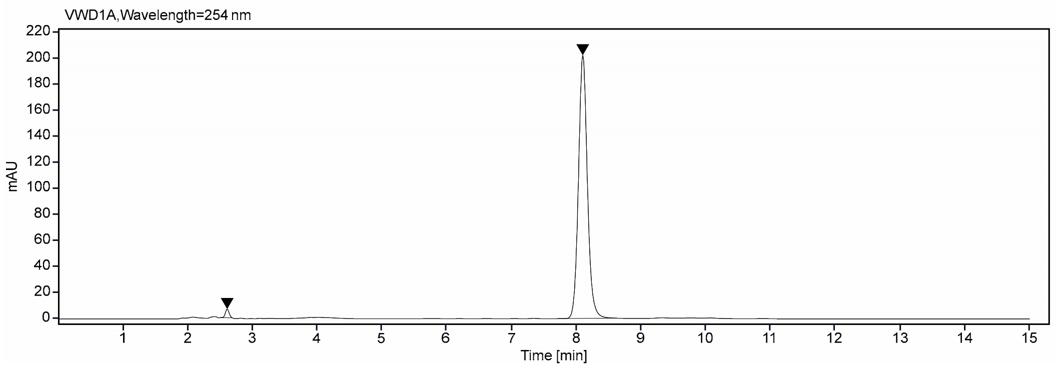

Signal: VWD1A,Wavelength=254 nm

RT [min]	Width [min]	Area	Height	Area%
2.601	0.18	26.86	6.65	1.37
8.094	0.90	1936.92	201.98	98.63
	**Sum**	**1963.78**		

Figure S114. HPLC spectrum of compound **S3-7**.

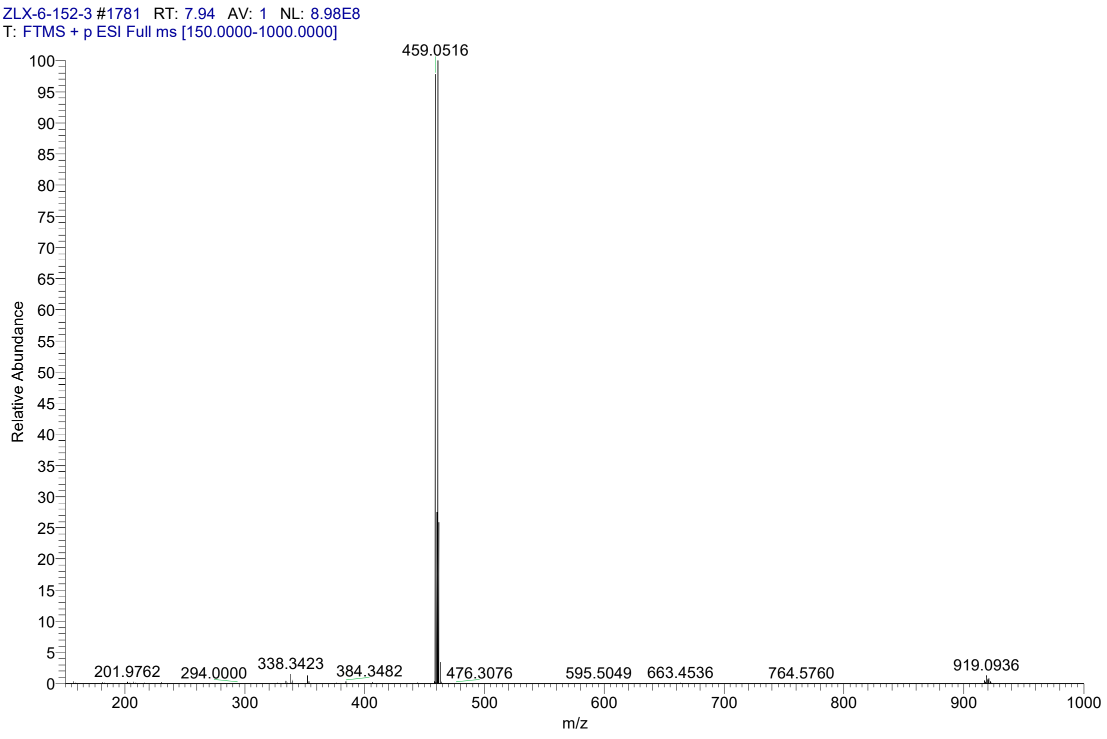

Figure S115. HRMS of compound **S3-7**.

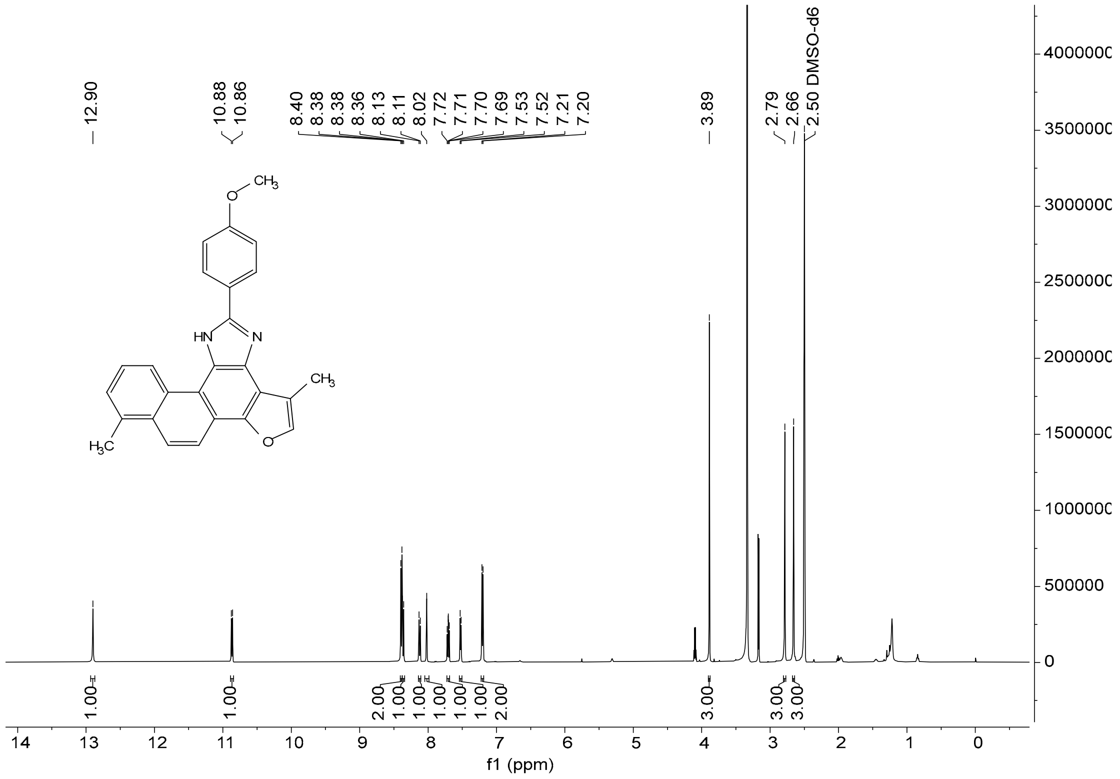

Figure S116. ^{1}H NMR spectrum (500 MHz, DMSO-d_6) of compound **S3-8**.

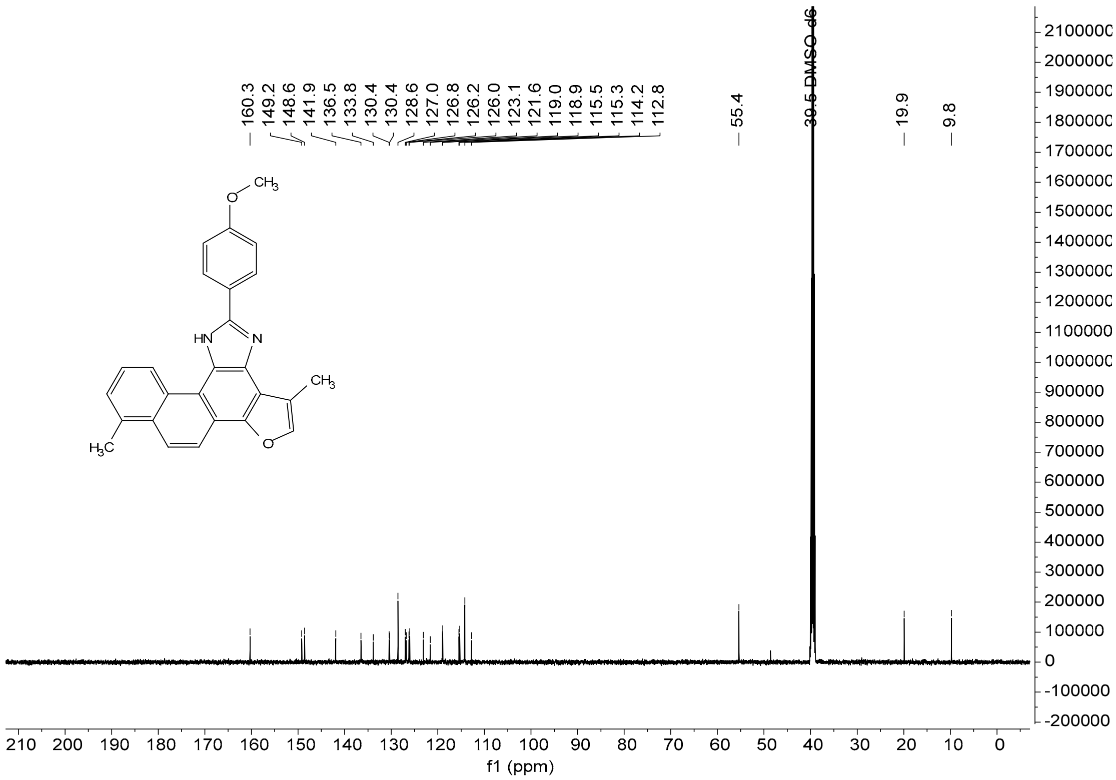

Figure S117. ^{13}C NMR spectrum (500 MHz, DMSO-d_6) of compound **S3-8**.

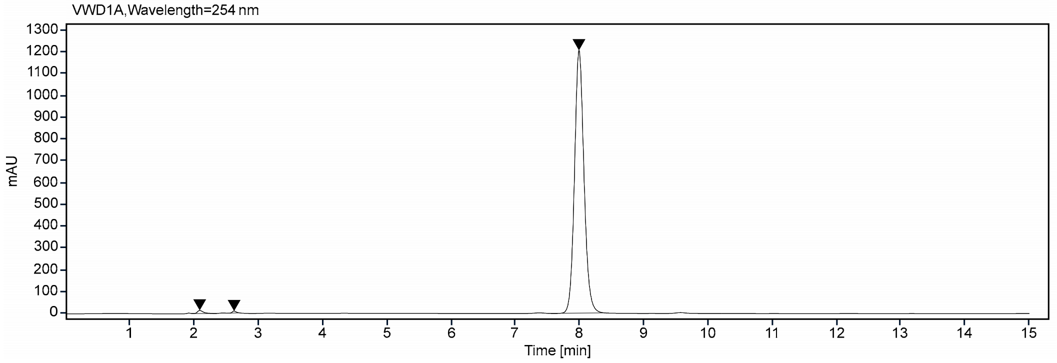

Signal: VWD1A,Wavelength=254 nm

RT [min]	Width [min]	Area	Height	Area%
2.076	0.39	87.46	14.03	0.70
2.611	0.20	32.94	8.81	0.26
7.983	0.89	12374.48	1205.88	99.04
	**Sum**	**12494.87**		

Figure S118. HPLC spectrum of compound **S3-8**.

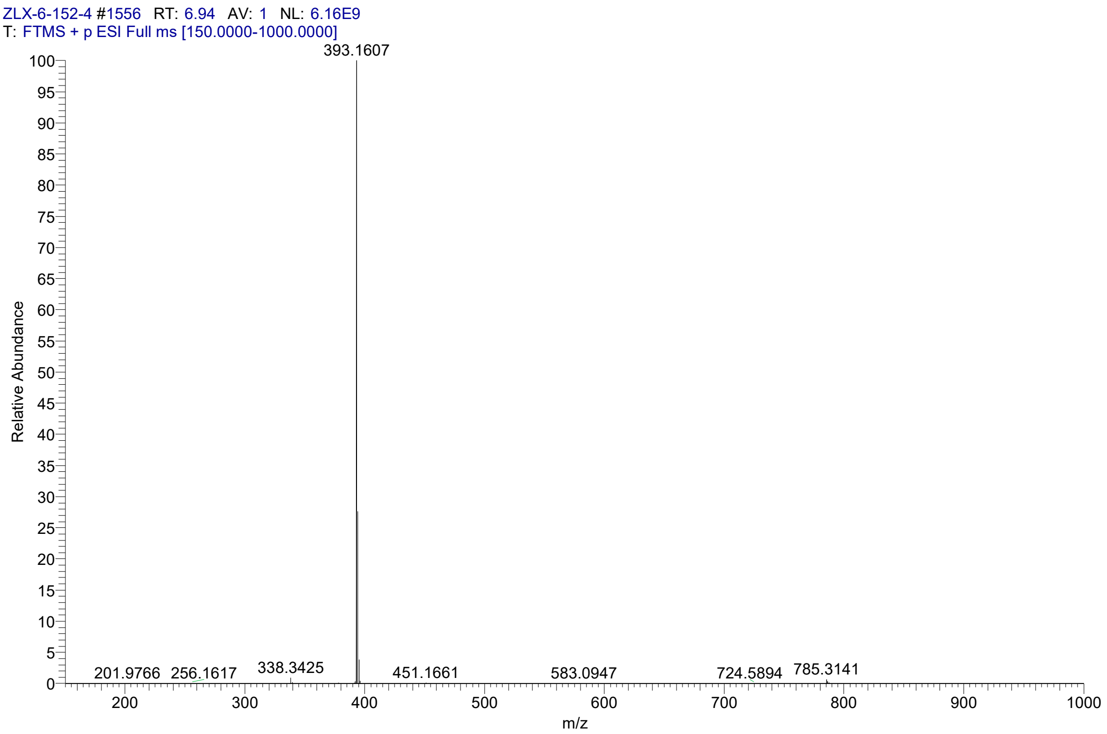

Figure S119. HRMS of compound **S3-8**.

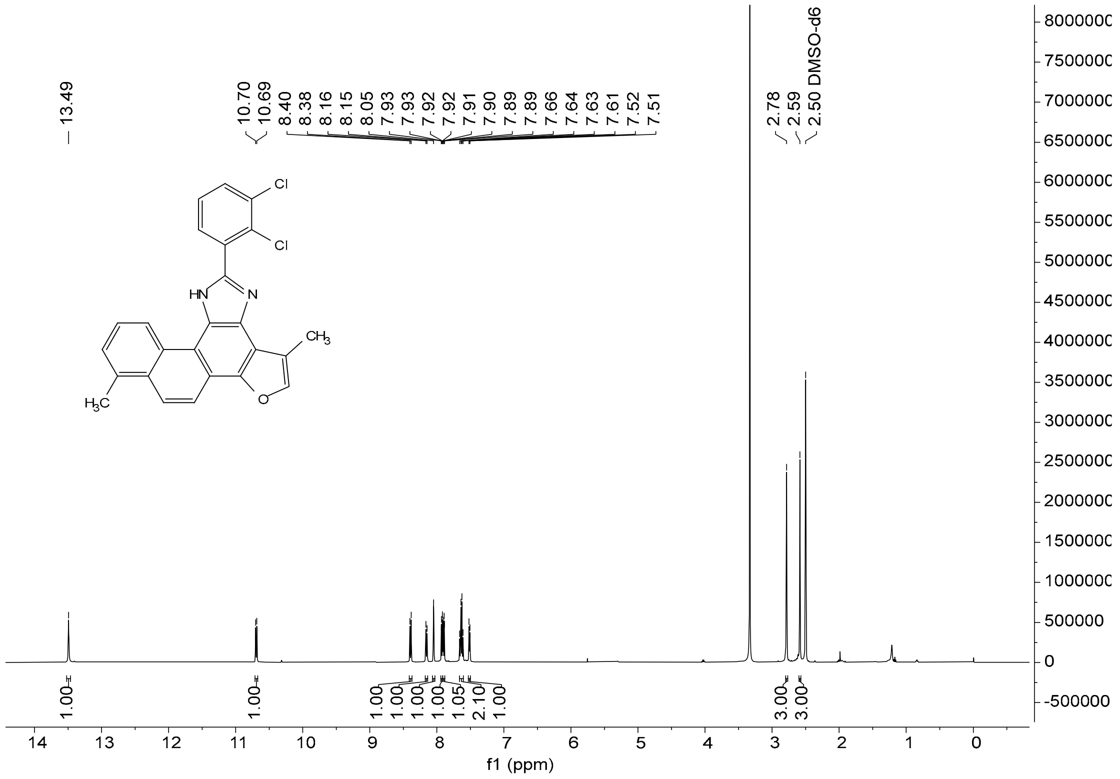

Figure S120. ^{1}H NMR spectrum (500 MHz, DMSO-d_6) of compound **S3-9**.

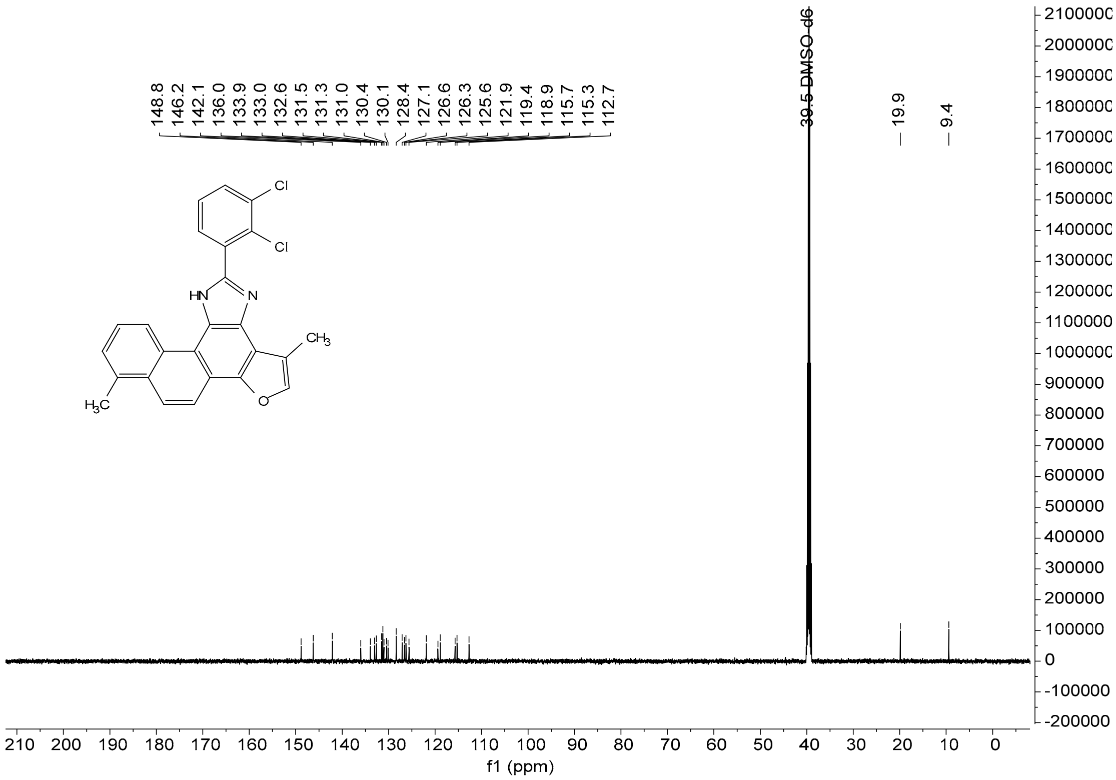

Figure S121. ^{13}C NMR spectrum (500 MHz, DMSO-d_6) of compound **S3-9**.

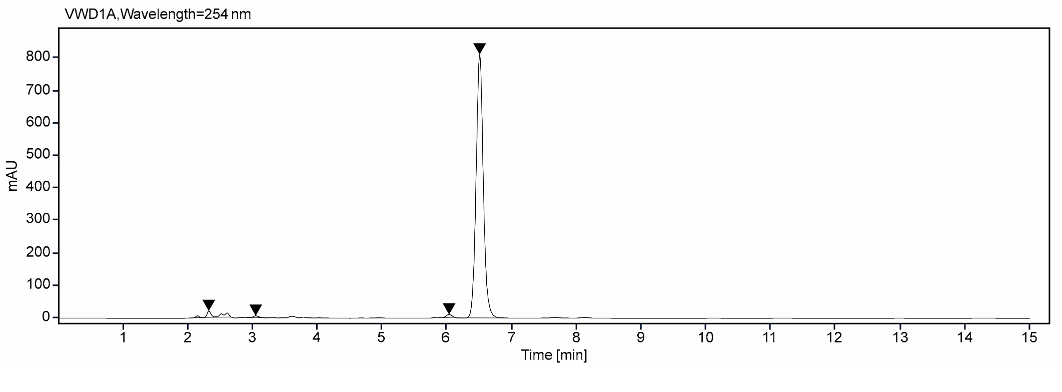

Signal: VWD1A,Wavelength=254 nm

RT [min]	Width [min]	Area	Height	Area%
2.318	0.57	194.88	19.82	2.97
3.043	0.31	27.25	5.44	0.42
6.029	0.18	53.07	9.24	0.81
6.501	0.74	6291.30	812.10	95.81
	**Sum**	**6566.50**		

Figure S122. HPLC spectrum of compound **S3-9**.

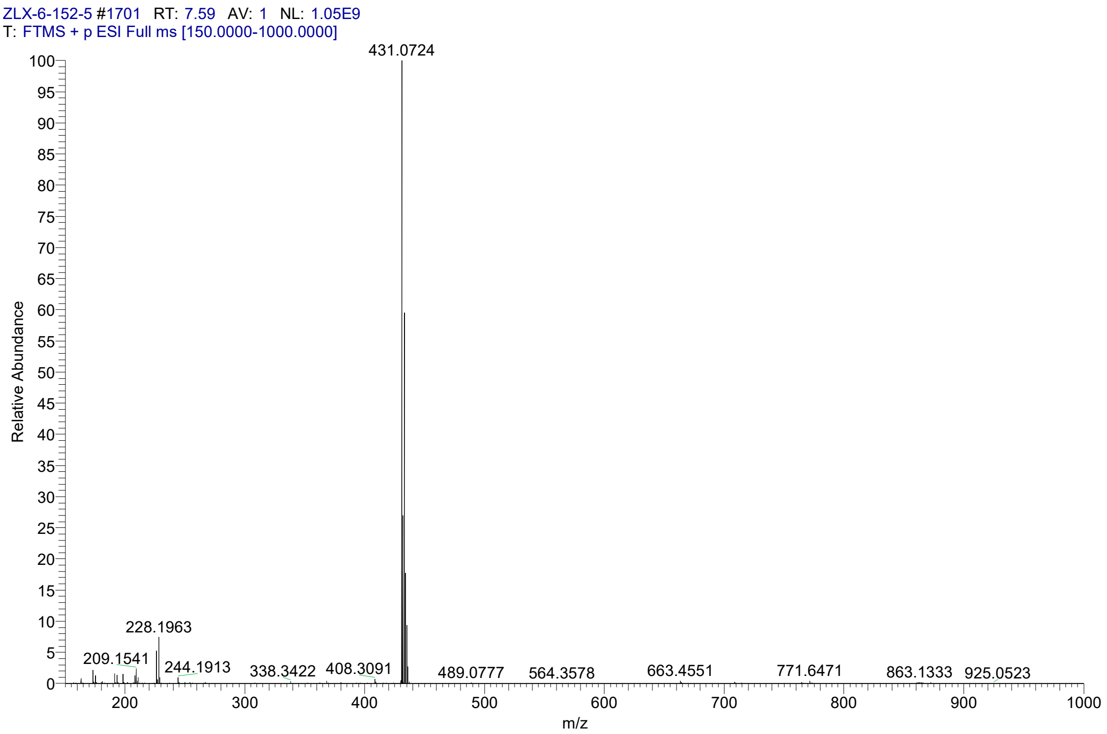

Figure S123. HRMS of compound **S3-9**.

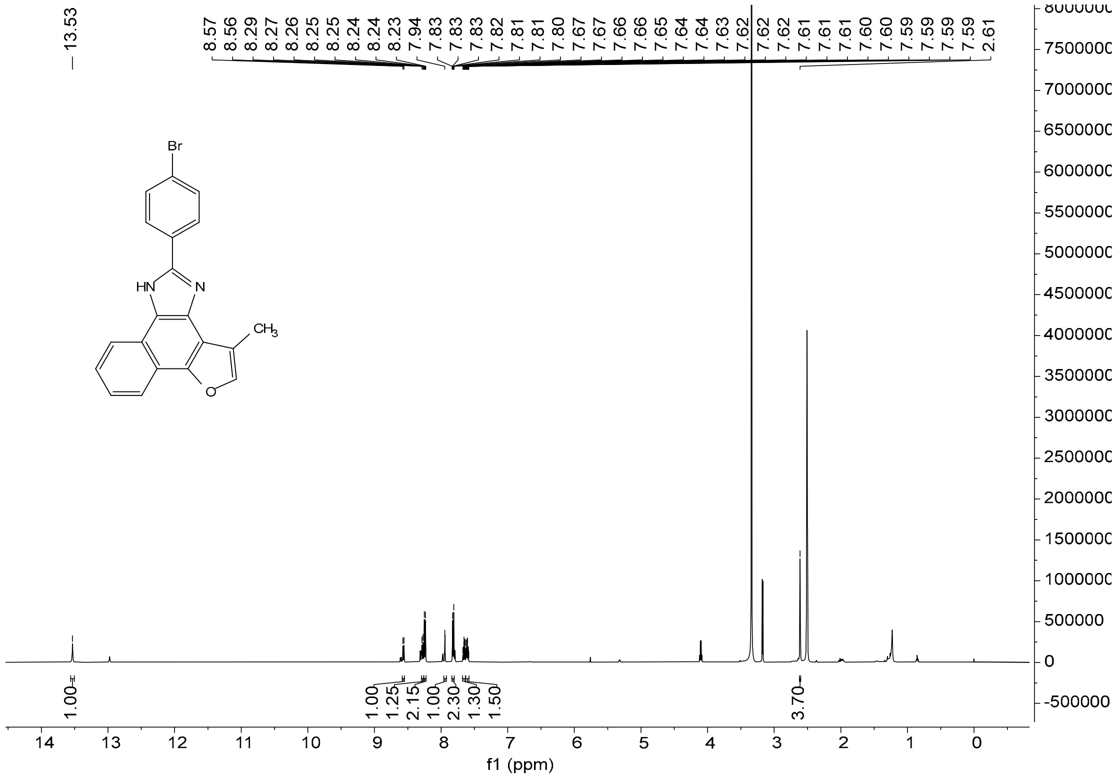

Figure S124. ¹H NMR spectrum (500 MHz, DMSO-d_6) of compound **S3-10**.

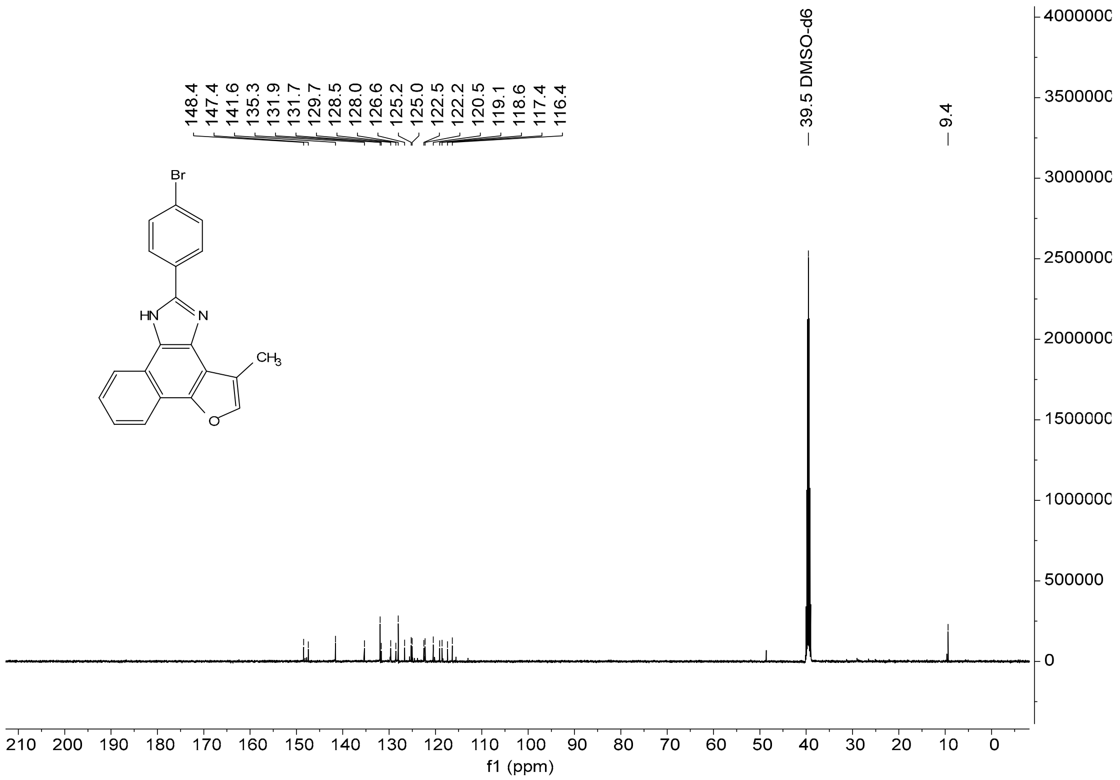

Figure S125. ^{13}C NMR spectrum (500 MHz, DMSO-d_6) of compound **S3-10**.

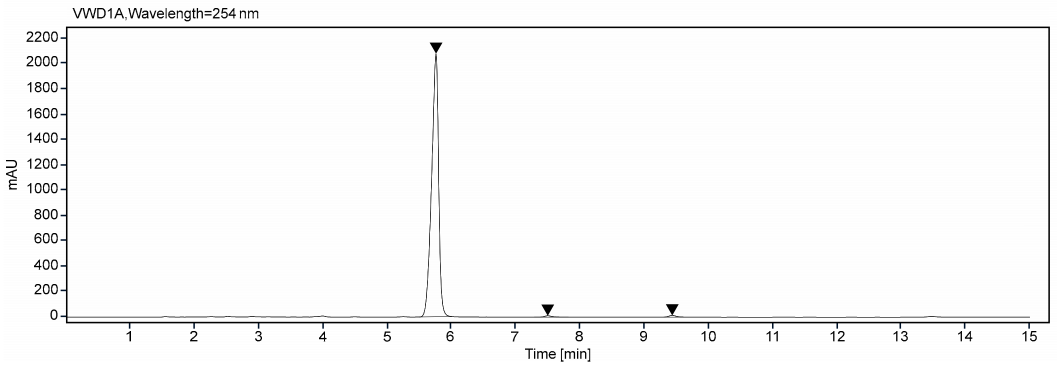

Signal: VWD1A,Wavelength=254 nm

RT [min]	Width [min]	Area	Height	Area%
5.752	0.50	15792.07	2077.92	98.76
7.492	0.54	93.50	11.33	0.58
9.430	0.31	104.09	13.39	0.65
	**Sum**	**15989.67**		

Figure S126. HPLC spectrum of compound **S3-10**.

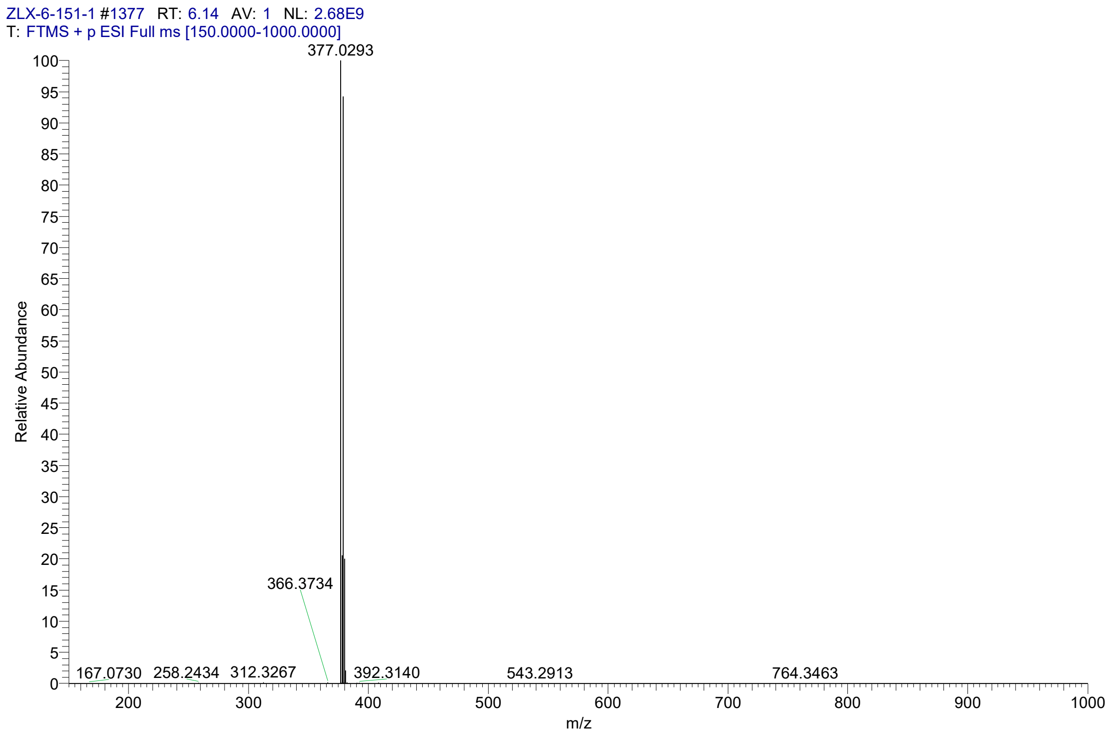

Figure S127. HRMS of compound **S3-10**.

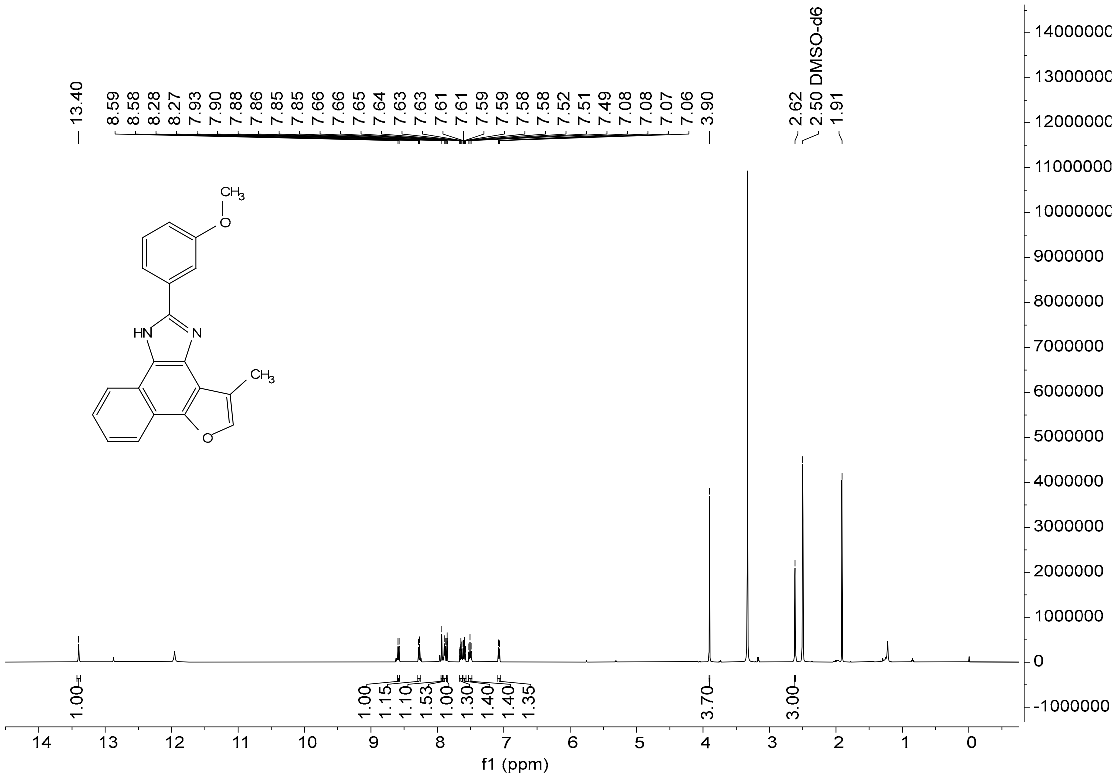

Figure S128. ^{1}H NMR spectrum (500 MHz, DMSO-d_6) of compound **S3-11**.

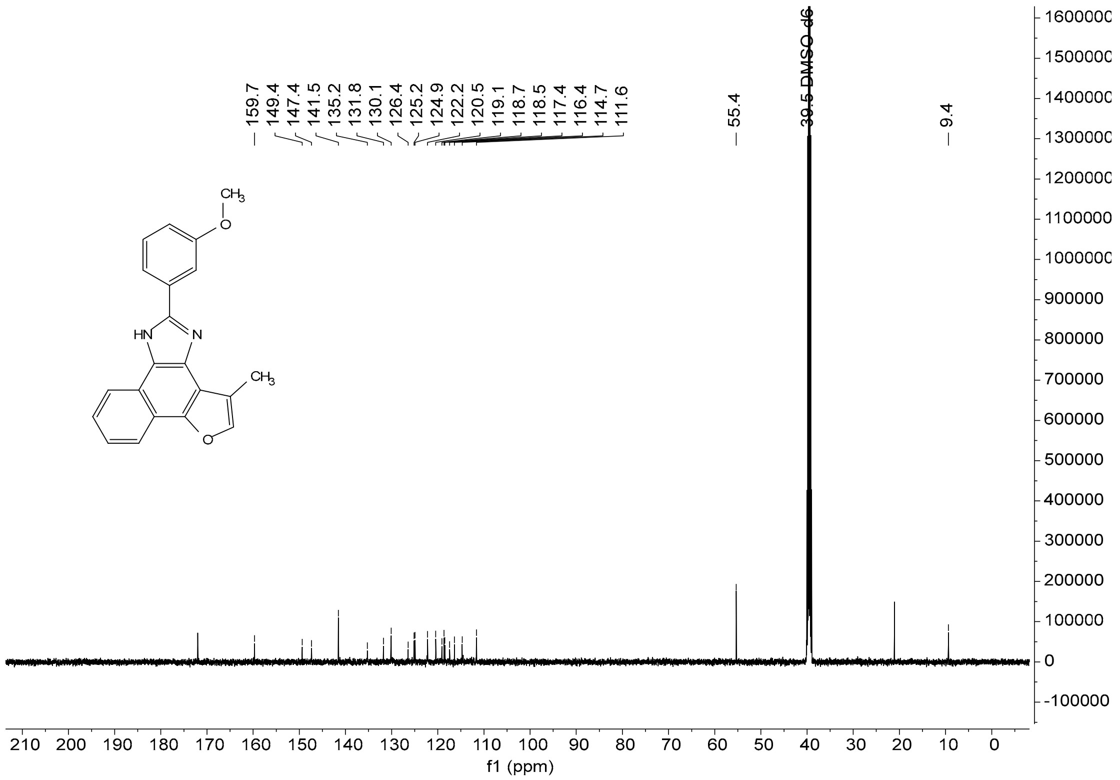

Figure S129. ^{13}C NMR spectrum (500 MHz, DMSO-d_6) of compound **S3-11**.

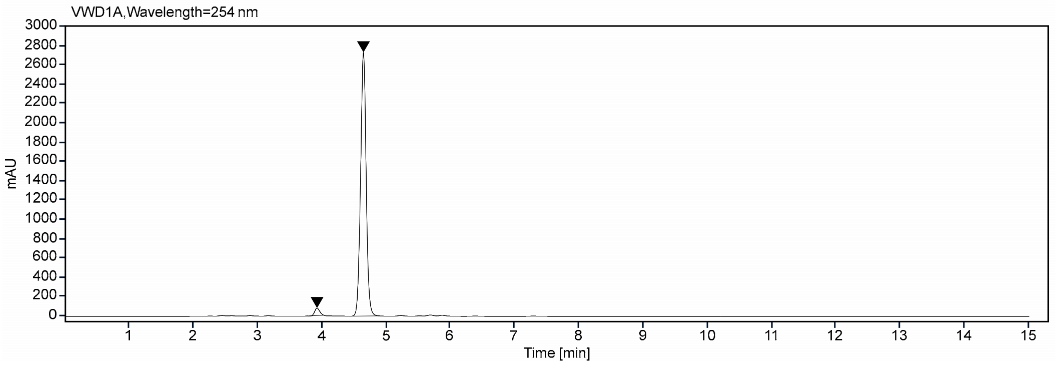

Signal: VWD1A,Wavelength=254 nm

RT [min]	Width [min]	Area	Height	Area%
3.919	0.27	404.91	76.80	2.40
4.642	0.44	16445.80	2730.60	97.60
	**Sum**	**16850.71**		

Figure S130. HPLC spectrum of compound **S3-11**.

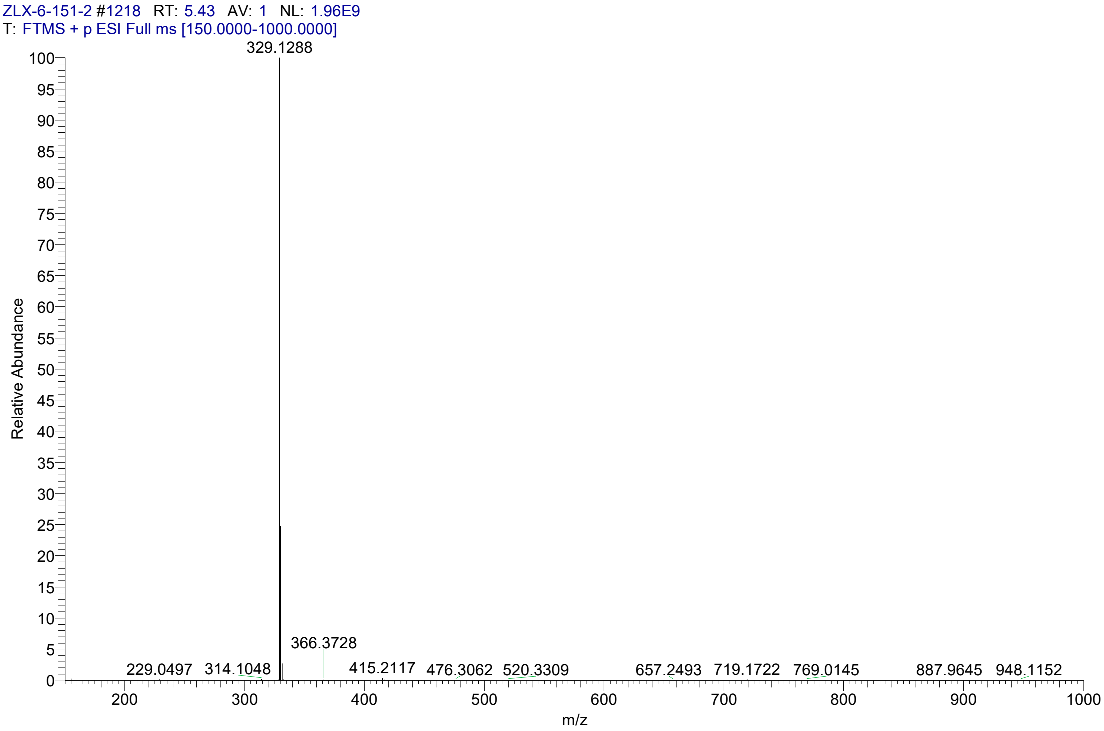

Figure S131. HRMS of compound **S3-11**.

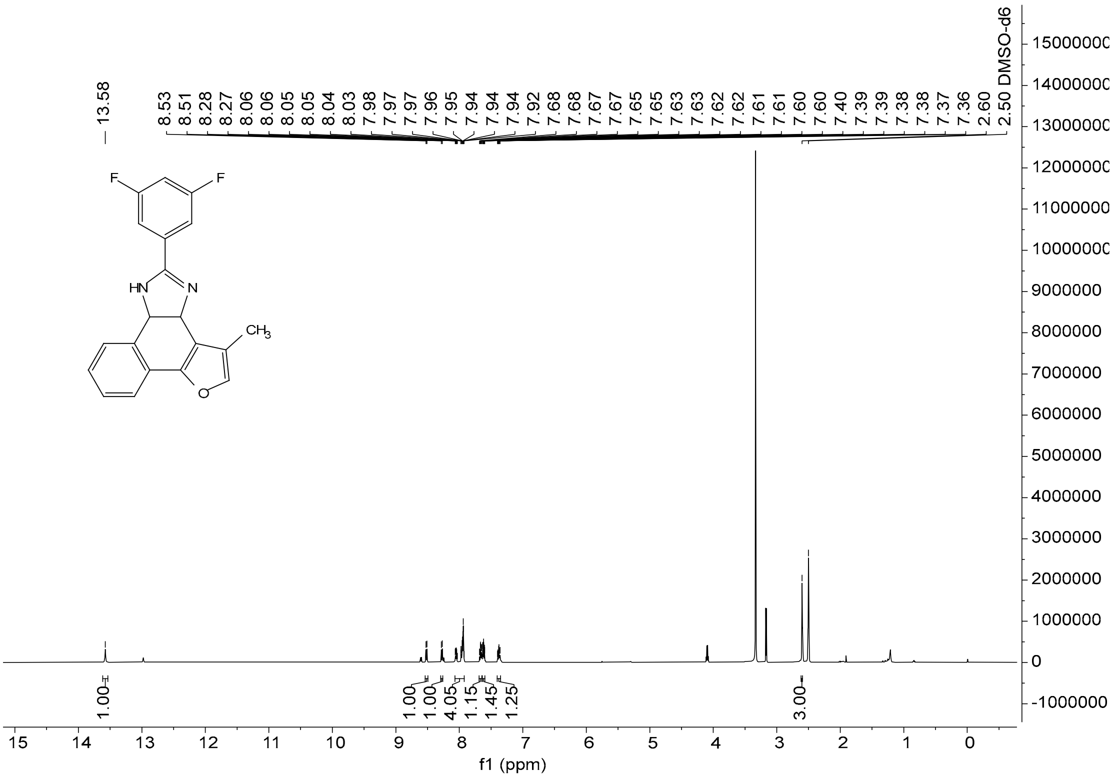

Figure S132. ^{1}H NMR spectrum (500 MHz, DMSO-d_6) of compound **S3-12**.

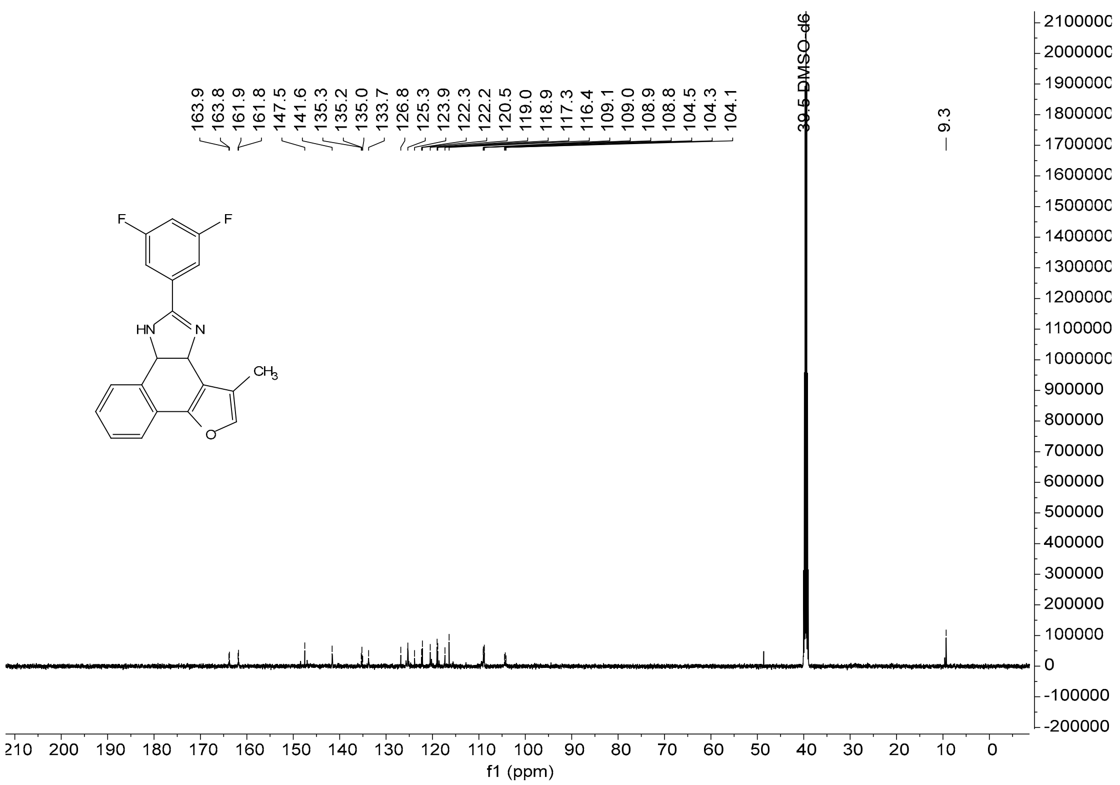

Figure S133. ^{13}C NMR spectrum (500 MHz, DMSO-d_6) of compound **S3-12**.

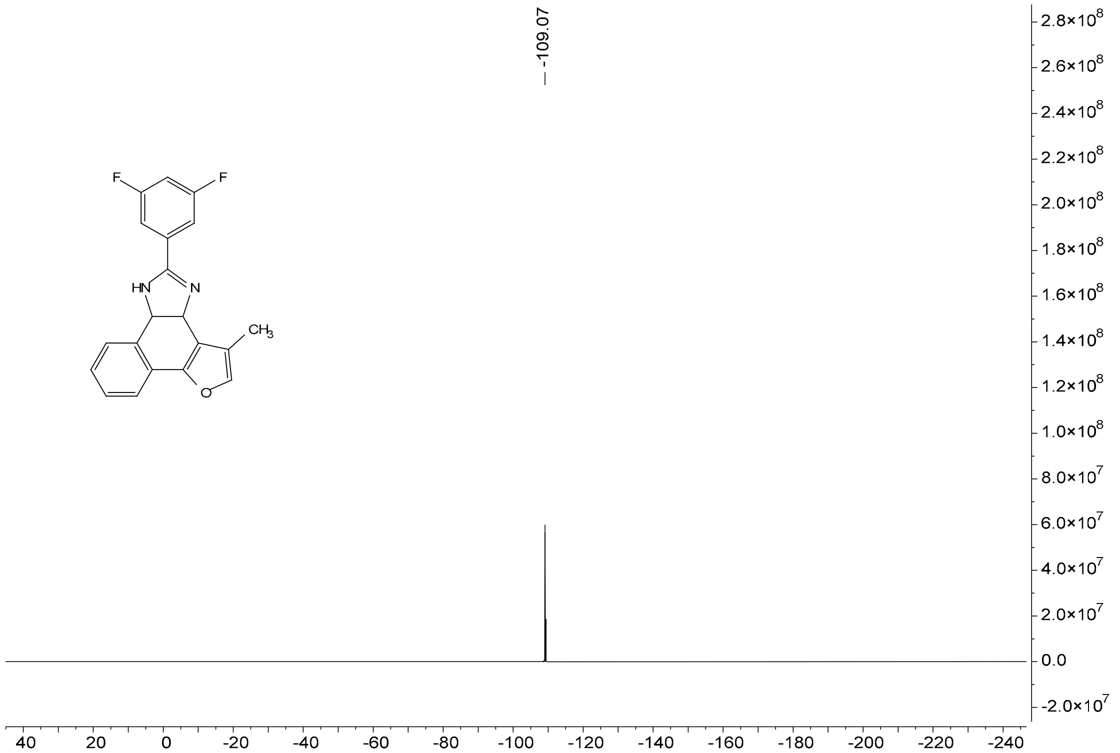

Figure S134. ^{19}F NMR spectrum (500 MHz, DMSO-d_6) of compound **S3-12**.

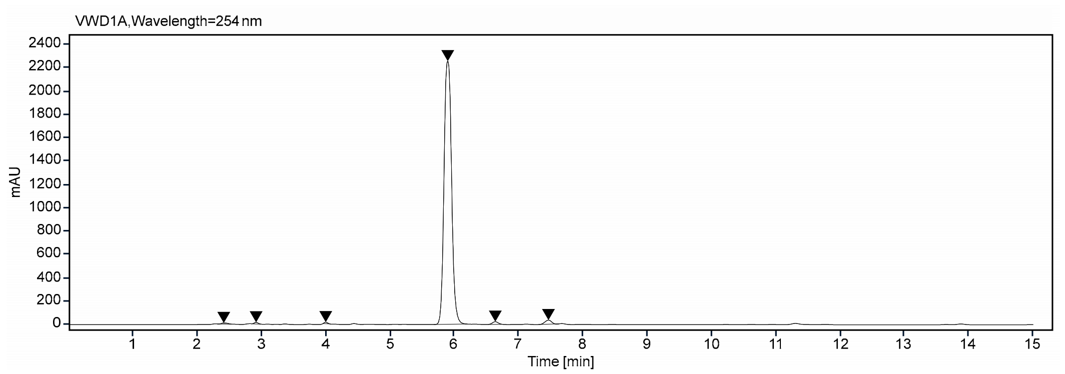

Signal: VWD1A,Wavelength=254 nm

RT [min]	Width [min]	Area	Height	Area%
2.402	0.23	60.66	9.12	0.32
2.905	0.15	55.82	13.82	0.29
3.990	0.12	50.49	13.45	0.27
5.888	0.51	18418.61	2256.55	97.10
6.628	0.21	138.08	22.81	0.73
7.456	0.31	245.36	35.03	1.29
	**Sum**	**18969.02**		

Figure S135. HPLC spectrum of compound **S3-12**.

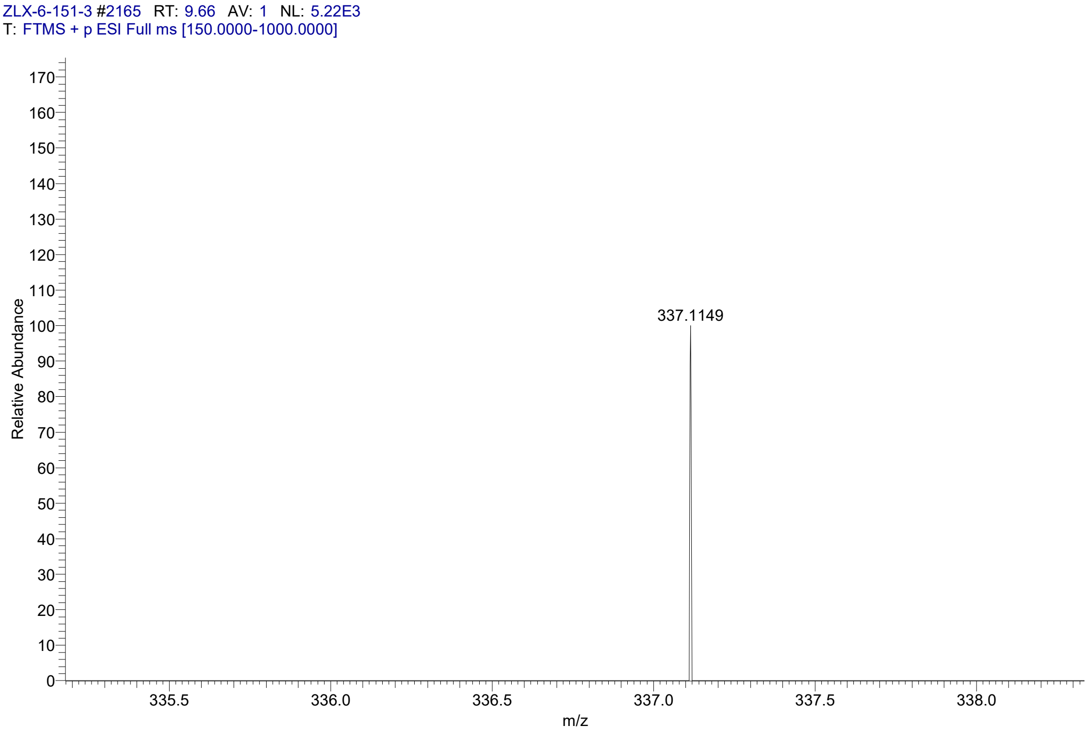

Figure S136. HRMS of compound **S3-12**.

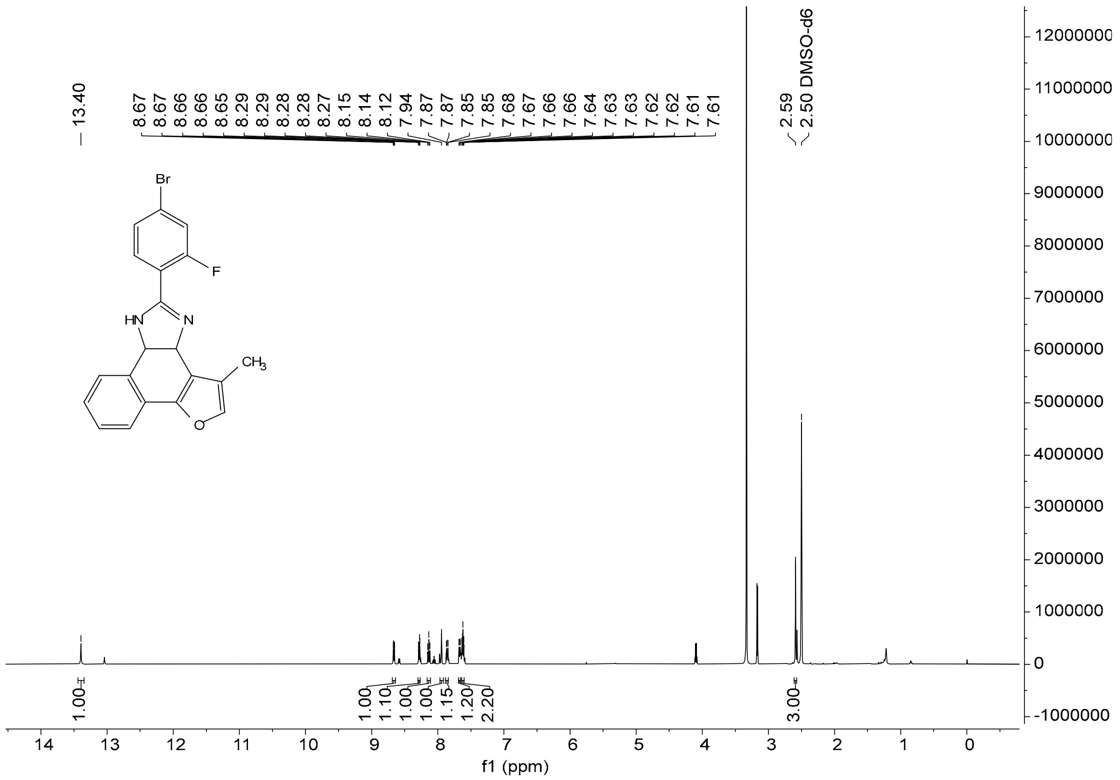

Figure S137. ^{1}H NMR spectrum (500 MHz, DMSO-d_6) of compound **S3-13**.

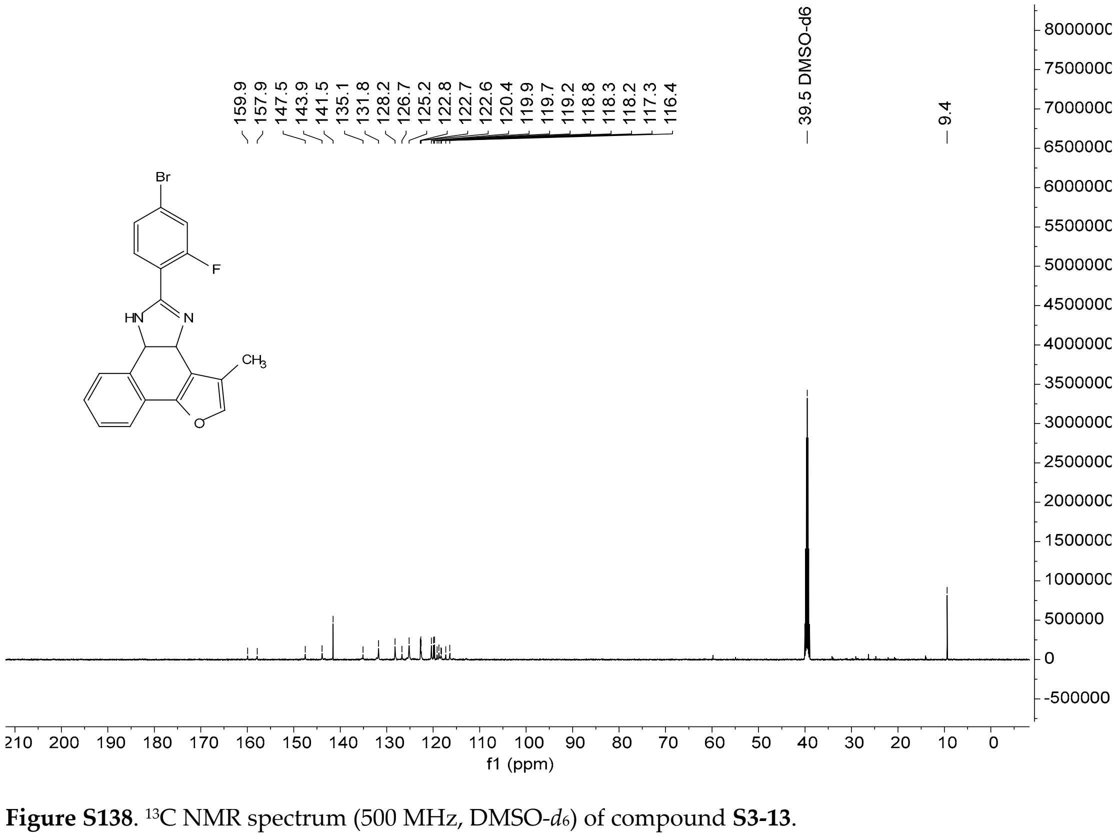

Figure S138. ^{13}C NMR spectrum (500 MHz, DMSO-d_6) of compound **S3-13**.

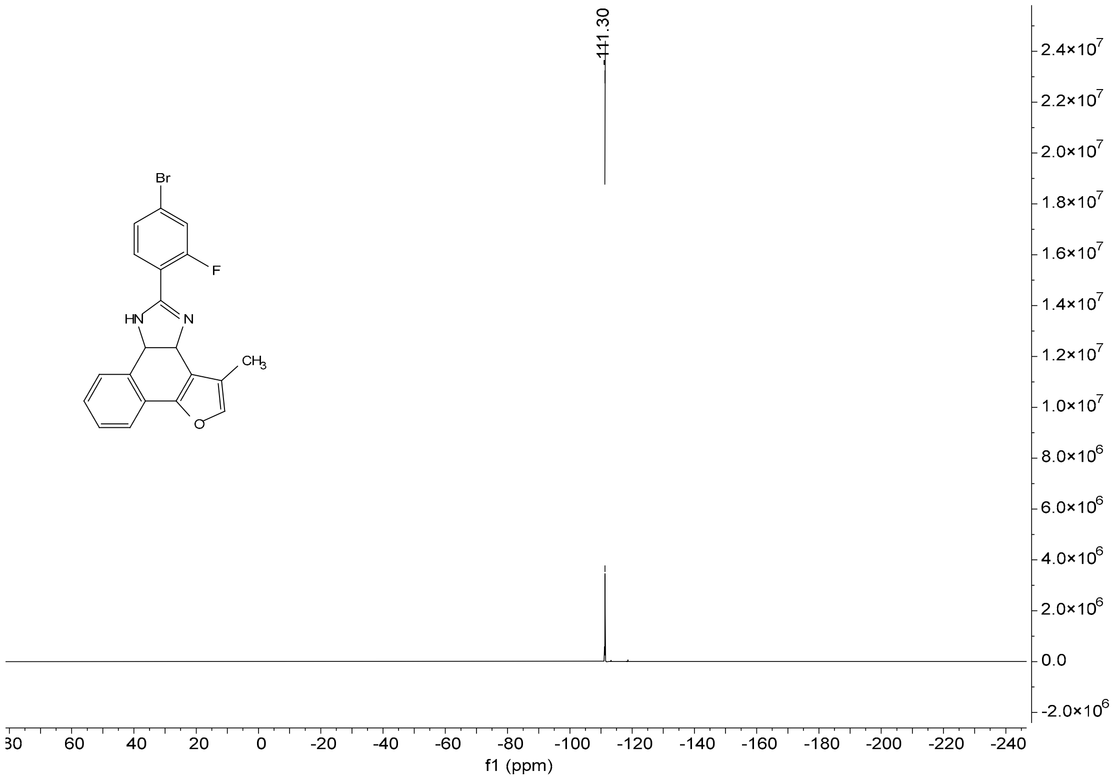

Figure S139. ^{19}F NMR spectrum (500 MHz, DMSO-d_6) of compound **S3-13**.

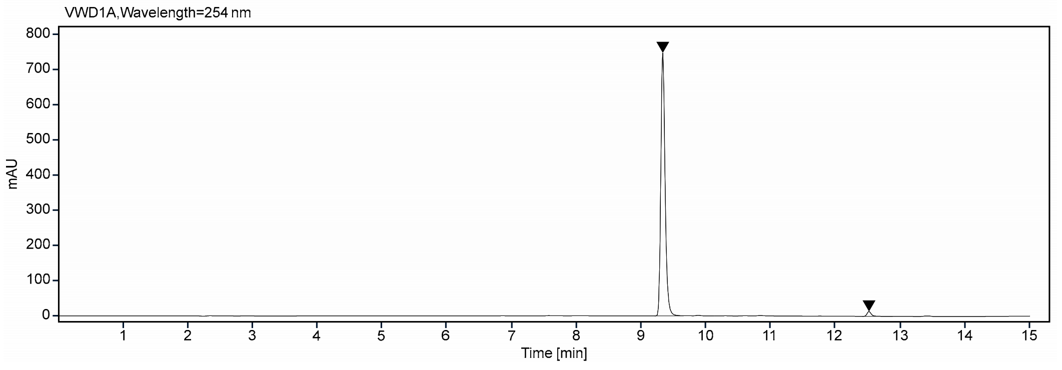

Signal: VWD1A,Wavelength=254 nm

RT [min]	Width [min]	Area	Height	Area%
9.328	0.45	3445.40	747.51	98.13
12.511	0.47	65.80	13.69	1.87
	**Sum**	**3511.20**		

Figure S140. HPLC spectrum of compound **S3-13**.

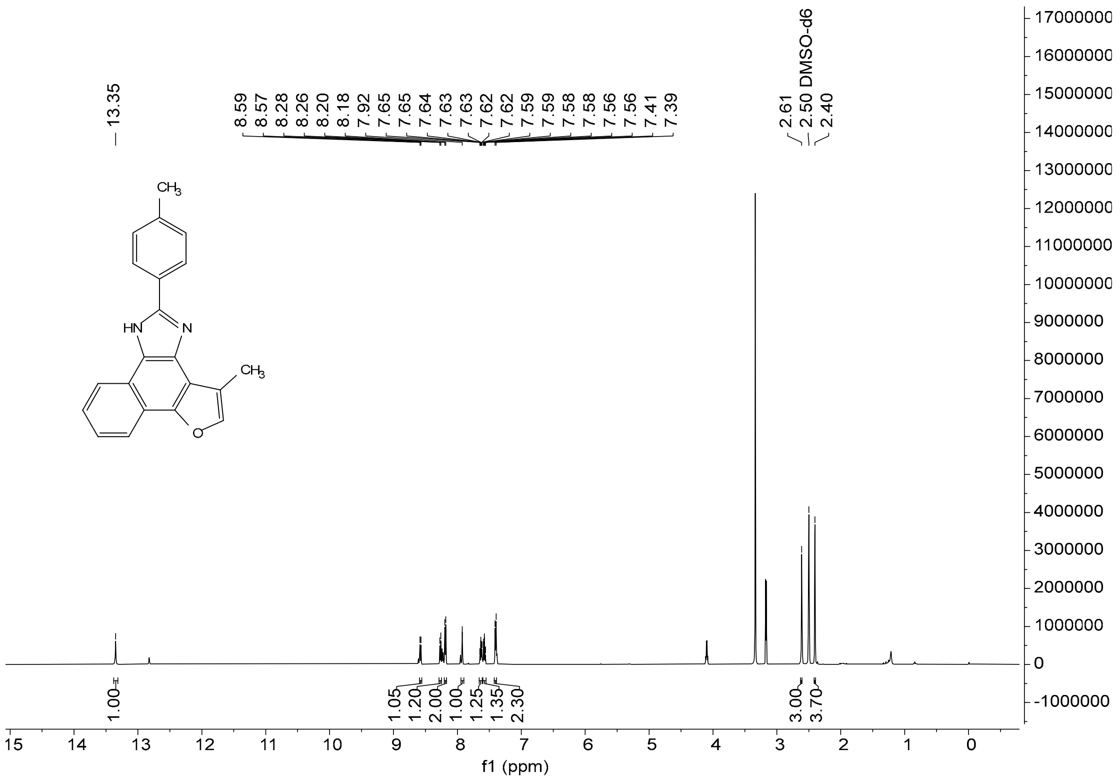

Figure S141. ^{1}H NMR spectrum (500 MHz, DMSO-d_6) of compound **S3-14**.

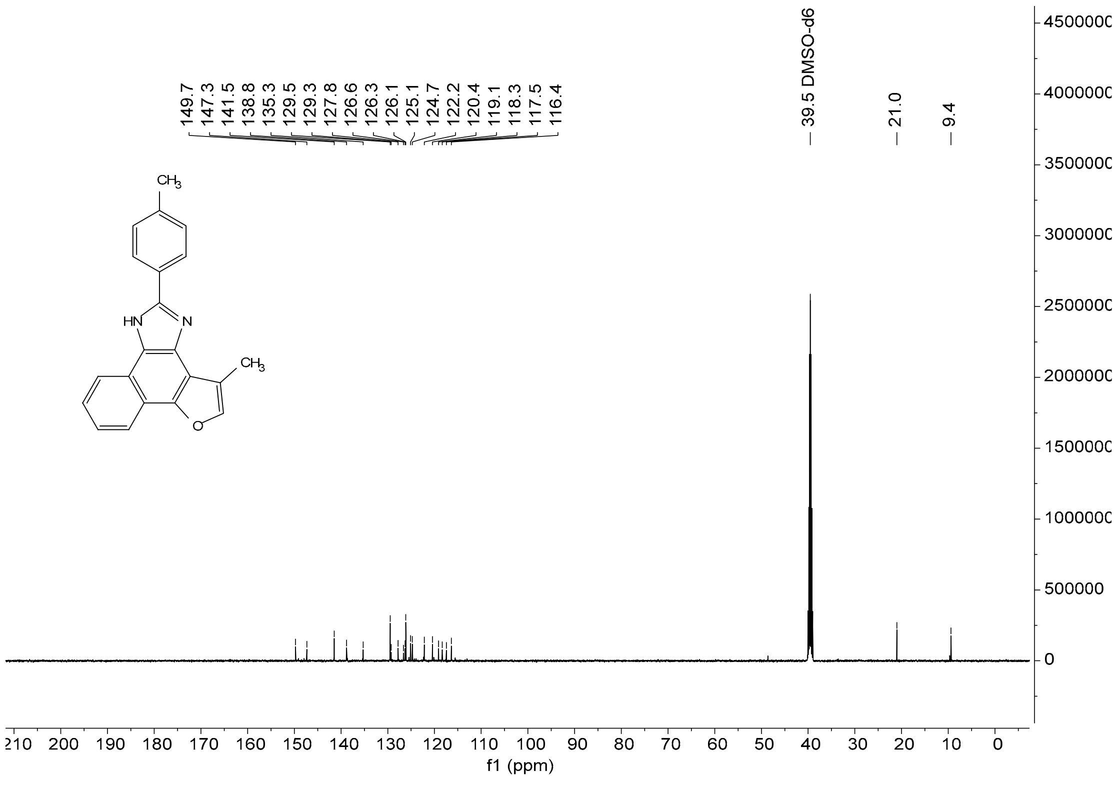

Figure S142. ^{13}C NMR spectrum (500 MHz, DMSO-d_6) of compound **S3-14**.

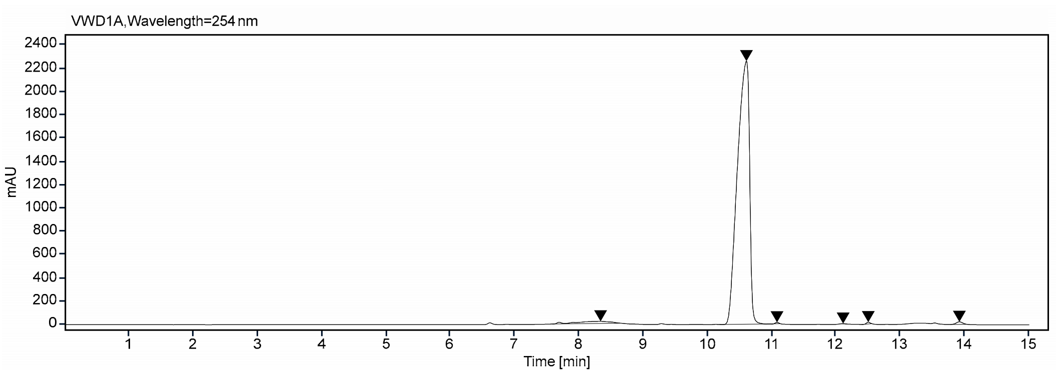

Signal: VWD1A,Wavelength=254 nm

RT [min]	Width [min]	Area	Height	Area%
8.331	1.07	730.70	18.46	2.39
10.601	0.73	29614.66	2261.44	96.76
11.078	0.21	43.66	12.52	0.14
12.107	0.10	9.72	2.81	0.03
12.498	0.16	57.46	15.62	0.19
13.915	0.26	150.91	22.04	0.49
	**Sum**	**30607.11**		

Figure S143. HPLC spectrum of compound **S3-14**.

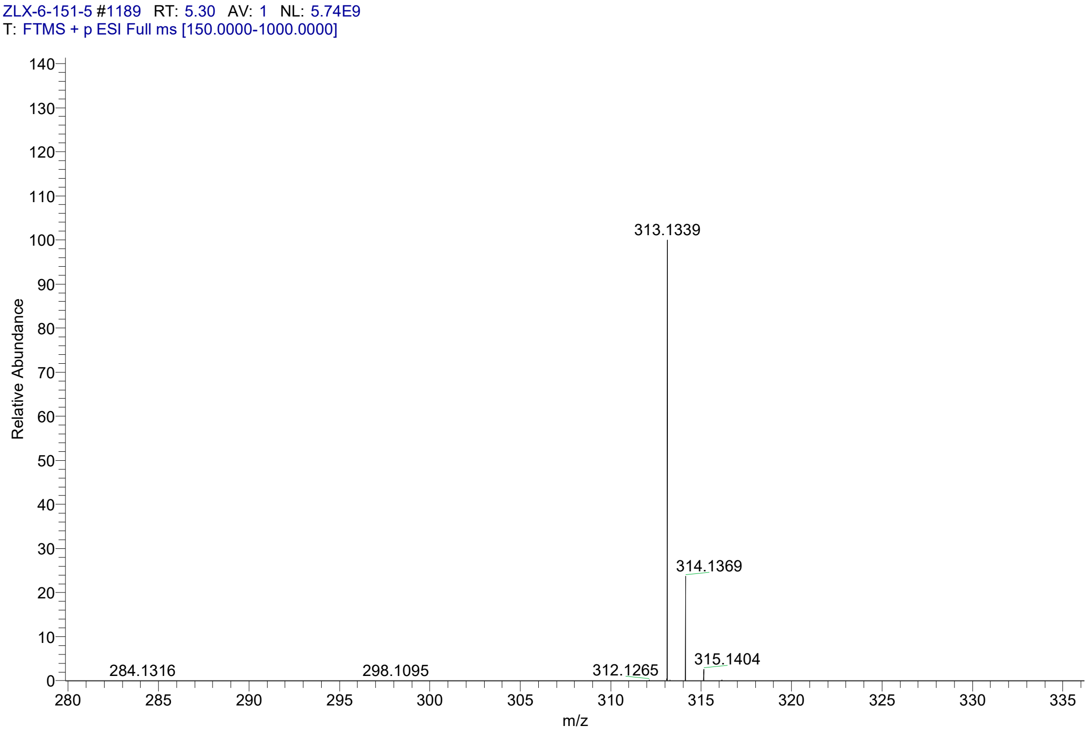

Figure S144. HRMS of compound **S3-14**.
